# Radical Sampling Enabled Saturated N‑Heterocycle Cyclization

**DOI:** 10.1021/jacs.6c01294

**Published:** 2026-04-08

**Authors:** Qinyan Cai, Noah B. Bissonnette, Saegun Kim, Thomas Knauber, Gary M. Chinigo, David C. Blakemore, David W. C. MacMillan

**Affiliations:** † Merck Center for Catalysis at Princeton University, Princeton, New Jersey 08544, United States; ‡ Pfizer Research and Development, Groton, Connecticut 06340, United States

## Abstract

Nitrogen-containing saturated heterocycles are essential structural motifs in many small molecule drugs, and the development of methods to rapidly access these scaffolds remains highly desirable. A modular and streamlined strategy for accessing these motifs involves *in situ* condensation of an aldehyde and an amine, followed by radical generation and cyclization to afford the desired heterocycle. Current applications of this strategy are limited in scope and efficiency, requiring amines that are prefunctionalized with the radical progenitor. Expansion of this approach to unfunctionalized amines, by using native C–H bonds as radical precursors, would greatly expand the scope, modularity, and expediency toward heterocycles. However, the selective C–H bond activation required to effect cyclization is challenging. Radical sampling, characterized by net-reversible hydrogen atom transfer (HAT) processes, addresses this issue by deploying cyclization as the product-determining step. This strategy leverages kinetic differences among competing cyclization pathways to selectively quench undesired radical intermediates with slower rates of cyclization while allowing the rapidly cyclizing radical to form the target six-membered ring. Herein, we report a general cyclization strategy that constructs versatile saturated heterocycles directly from aldehydes and amines through a radical sampling mechanism.

## Graphical abstract

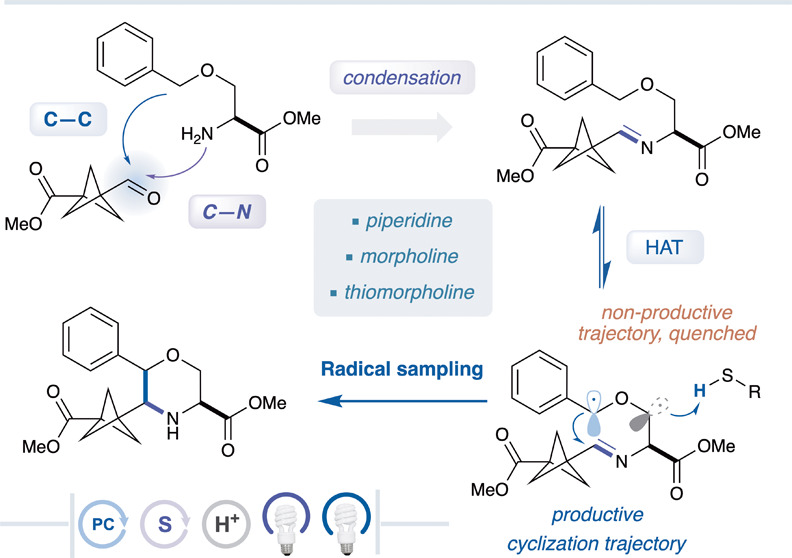

Nitrogen-containing heterocycles are among the most prevalent structural motifs in pharmaceutical compounds.^1^ Accordingly, approximately 82% of new FDA-approved small molecule therapeutics (2013–2023) incorporate at least one nitrogen-containing heterocycle. Among the diverse heterocyclic scaffolds, six-membered, nonaromatic nitrogen ring systems have attracted substantial interest in modern medicinal chemistry. Their topologically flexible, *sp* ^*3*^ character can imbue desirable properties, such as increased solubility, relative to their planar congeners.^2^ Given their utility, piperidine (ranked second) and morpholine (ranked ninth) are among the most common heterocycles found in FDA-approved drugs (Figure 1).^1^

**Figure 1:**
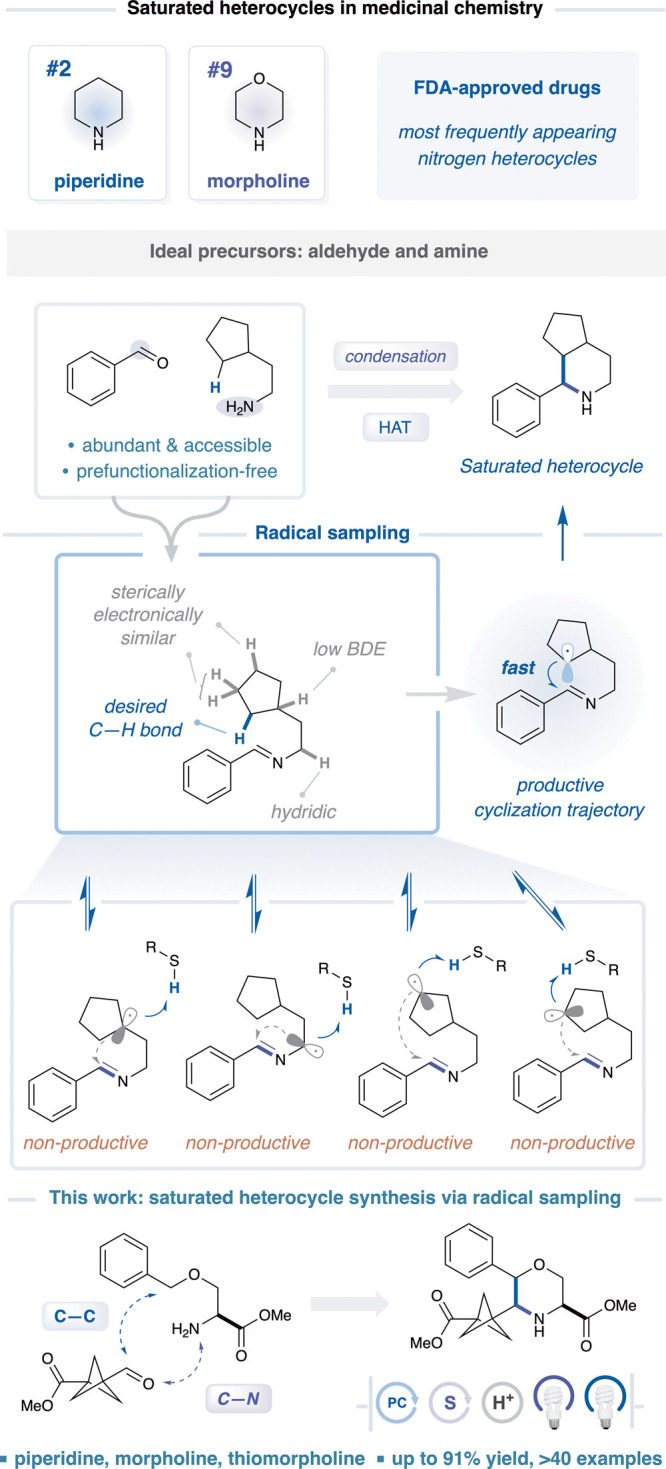
Saturated heterocycle cyclization via radical sampling and HAT.

Traditionally, these motifs are accessed through nucleophilic cyclization and metal-mediated cascade cyclization, which afford versatile cyclized products but require prefunctionalized building blocks prepared through multistep synthesis.^3–23^ One attractive modern strategy for *de novo* ring synthesis employs an aldehyde and an amine bearing a radical precursor. Mechanistically, the two partners are joined by condensation to assemble the core skeleton, followed by functional-group-directed carbon-centered radical generation and subsequent 6-endo-trig ring closure.^24^ Although this approach is quite modular, such methods still require amines bearing either preinstalled substituents,^24–29^ prepared via multistep synthesis, or oxidizable functionalities.^30,31^ These constraints limit the ability to access different heterocycle classes with versatile substitution patterns in an expedited fashion. Consequently, the development of highly modular methods to access a diverse range of saturated heterocyclic scaffolds from native functionalities without prefunctionalization remains a high priority for medicinal chemists.

We questioned whether piperidines, morpholines, and thiomorpholines could be assembled directly from native, widely available, unfunctionalized amines (Figure 1).^32^ Following condensation to form the imine intermediate, hydrogen-atom abstraction (HAA) would generate the key radical species required for cyclization (Figure 1).^33^ Appealingly, this approach exploits C–H bonds as native functional handles, yet several challenges complicate the use of HAA for heterocycle construction. Given that HAA selectivity is generally dictated by intrinsic properties of C–H bonds—such as hydricity, sterics, and bond dissociation energy (BDE)—the site of C–H abstraction is typically substrate-dependent (Figure 1).^34^ According to our design plan, however, the target site of C–H abstraction for the desired cyclization often lacks such differentiating characteristics. We thus require an HAA-independent strategy to achieve selectivity.

To this end, we contemplated an alternative design principle that would enable substrate-agnostic access to the desired product by kinetically “sorting” the stochastically generated radicals via rates of cyclization in conjunction with a catalytic hydrogen atom donation (HAD) step. Our computational studies (see SI Table S11 for additional discussion) along with experimental measurements for related structures in literature^35,36^ show that 6-endo radical cyclization which forms the desired six-membered heterocyclic product is kinetically faster than undesired 4-endo, 5-endo, 7-endo radical cyclizations. To exploit these kinetic differences, we envisioned incorporating a HAD step operating at rates of 10^7–8^ M^–1^ s^–1^ that could return slower-cyclizing radicals (<10^8^ s^–1^) to starting materials while allowing faster-cyclizing radicals (>10^8^ s^–1^) to undergo product formation.^37^ This approach, which relies on (1) reversible generation of radicals through paired HAA/HAD steps and (2) a discrete chemical step that dictates product formation, is known as *radical sampling* (Figure 1).^38^ This emerging mechanistic paradigm has enabled functional group migration^39,40^ and site-selective borylation of unactivated alkanes.^38^ Herein, we report the successful application of radical-sampling to generate versatile saturated heterocycles directly from native, unfunctionalized aldehydes and amines in an expedited fashion.

For optimal versatility, the HAA catalyst must be capable of abstracting hydrogen atoms from both electronically activated sites (e.g., α-oxy and α-thio C–H bonds, BDE ≈ 92 kcal/mol)^33^ and unactivated sites (methyl or methylene C–H bonds along unsubstituted alkyl chains, BDE ≈ 100 kcal/mol).^33^ Radicals centered on electronegative atoms—such as oxygen and chlorine—are highly electrophilic and therefore capable of abstracting strong C–H bonds at high rates.^41,42^ To minimize reaction components and streamline the system, photoinitiated direct HAA is preferred over indirect HAA pathways.^34^ In particular, decatungstate anion and FeCl_3_—which generates chlorine radicals via ligand-to-metal charge transfer (LMCT)^43^—have been applied in photoredox cross-coupling and radical-sampling chemistry and thus represent ideal HAA catalysts.^33,34,40,43–46^

For the HAD step, thiols and disulfides are well established to operate at the desired rates of 10^7–8^ M^–1^ s^–1^.^37^ Finally, although HAD renders the overall HAT process net reversible, α-amino C–H bonds remain electronically predisposed toward HAA. To suppress potential product inhibition arising from undesired HAA at these sites, we reasoned that employing excess acid to protonate the imine is necessary.^47–49^ Moreover, protonation enhances polarity matching, enabling the nucleophilic carbon-centered radical to cyclize more efficiently onto the electrophilic iminium acceptor.^48^

Figure 2 outlines a plausible mechanism for the transformation. Aldehyde **I** and amine **II** undergo condensation followed by protonation to generate iminium ion **III**. With light irradiation, photocatalyst **IV** is excited and generates species **V** via LMCT.^43^ The generated chlorine radical unselectively abstracts a hydrogen atom from one of multiple positions on the iminium ion, generating a mixture of radical species (**VII** and **VIII**). LMCT and HAA events simultaneously reduce the photocatalyst and produce HCl, affording **VI**.^43^ Slower cyclizing radical intermediates (**VII**) undergo HAD with thiol reagent **X** to regenerate iminium **III**, whereas radical intermediate **VIII**, bearing the appropriately positioned radical, rapidly undergoes the favored six-endo cyclization to selectively furnish the six-membered ring, **XI**.^35,36^ Subsequent oxidation of photocatalyst **VI** [*E*(Fe^2+^/Fe^3+^) = – 0.53 V vs SCE^52^] by thiol radical **IX** ^53^ or aminium radical cation **XI** ^47,49^ regenerates the active catalyst and furnishes thiol **X** or the saturated heterocycle **XII**.

**Figure 2:**
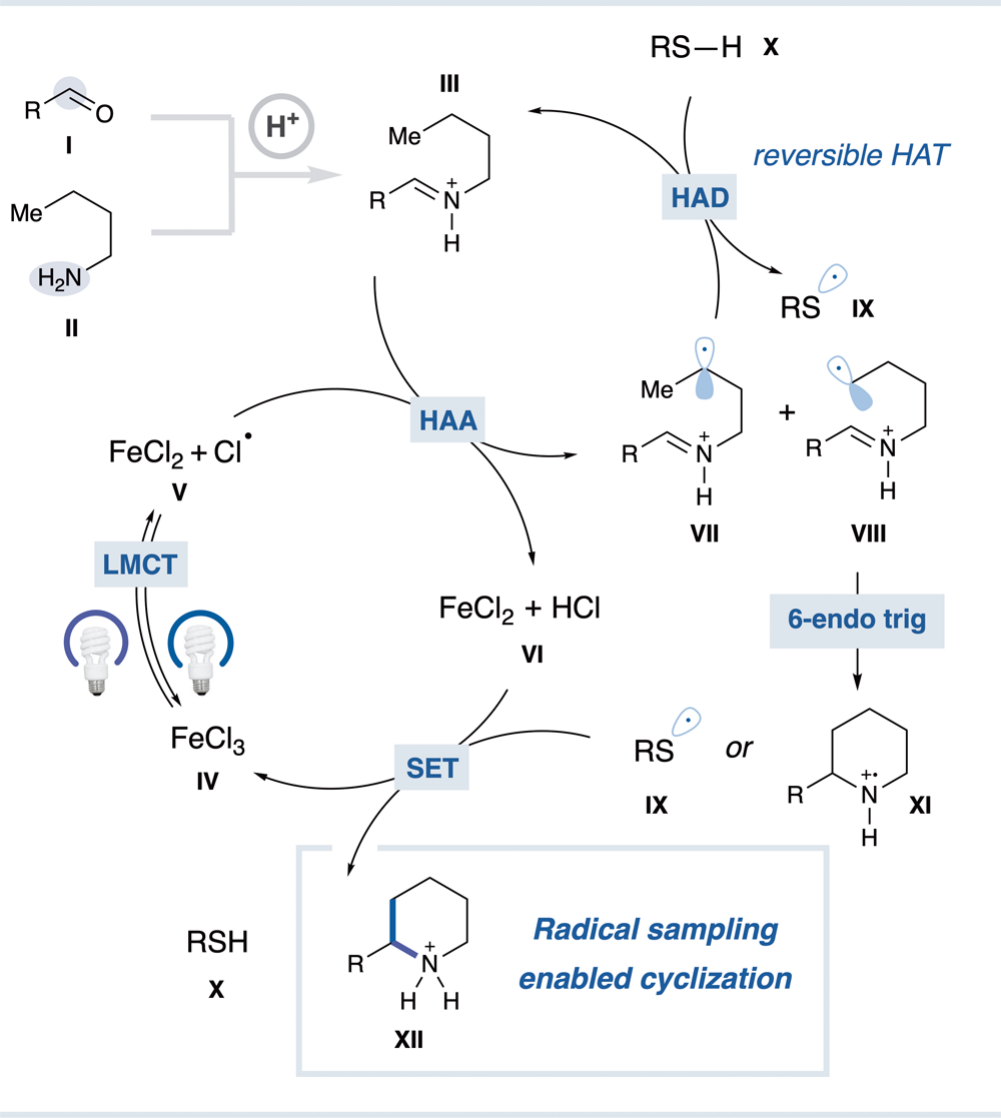
Plausible mechanism for saturated heterocycle cyclization via radical sampling and HAT.

We targeted piperidine cyclization as the model reaction, which gives access to a rarely precedented class of substrates in previous radical cyclization methodologies due to the challenge of selectively generating desired carbon-centered radicals along the unsubstituted alkyl chains. The reaction was first evaluated using methyl 4-formylbenzoate **1** and 3,3-dimethylbutan-1-amine **2** (Table 1). Encouragingly, piperidine **3** was obtained in 82% yield using FeCl_3_ as a photoinduced HAA catalyst under standardized 365 nm Integrated Photoreactor (IPR) irradiation (entry 1, see SI section 5 for UV–vis studies). The optimal conditions employed 4,4′-dimethoxyphenyl disulfide as the hydrogen atom donor, benzenesulfonic acid (p*K*_a_ = – 2.8 in water)^54^ as the acid, and acetonitrile as the solvent. As summarized in Table 1, careful selection of the HAA catalyst and acid proved critical for reactivity (see SI Tables S2–4 for additional optimization data). Substituting tetra-*n*-butylammonium decatungstate (TBADT) for FeCl_3_ resulted in a significantly lower cyclization efficiency (entry 2). Similarly, replacing benzenesulfonic acid with the weaker trifluoroacetic acid (TFA, p*K*_a_ = 0.2–0.5 in water)^55^ led to greatly diminished reactivity (entry 3). Importantly, in the absence of light, HAA catalyst, or acid, the reaction did not proceed due to a lack of radical generation or preferential abstraction at α-amino sites (entries 4–6). The disulfide also plays a key role in facilitating product formation. In its absence, cyclized product can still form, as multiple abstractable C–H bonds (nine total, including three methyl groups) are available. However, the reaction efficiency is greatly reduced, leading primarily to the noncyclized byproduct, methyl 4-(((3,3-dimethylbutyl)­amino)­methyl)­benzoate (entry 7).

**Table 1:**
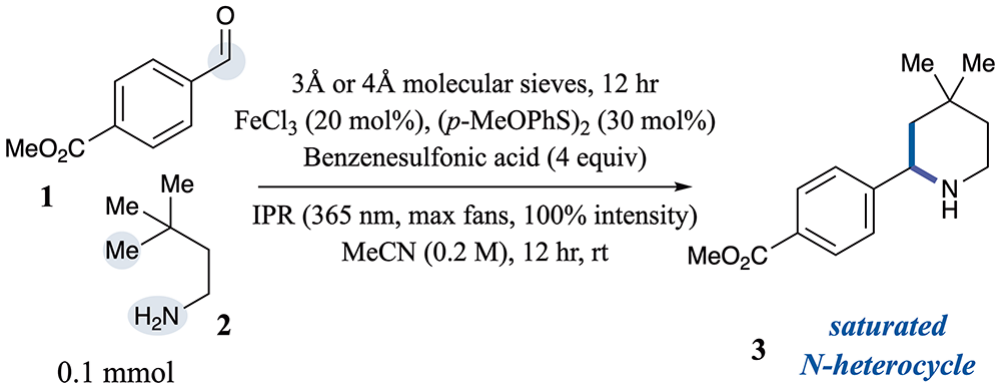
Optimization and Control Reactions^a^

entry	deviation	yield^b^
1	none	82%
2	TBADT instead of FeCl_3_	44%
3	TFA instead of benzenesulfonic acid	35%
4	no light	0%
5	no FeCl_3_	0%
6	no benzenesulfonic acid	0%
7	no (*p*-MeOPhS)_2_	45%

a See SI for experimental details.

b Yield determined by ^1^H NMR analysis versus 1,4-dinitrobenzene as an internal standard.

With the optimized conditions in hand, we next explored the scope of piperidine formation. As shown in Table 2, a variety of alkyl amines can be employed directly as coupling partners to generate piperidines in a one-pot fashion. Various substitutions on the amine are well tolerated, affording excellent yields (**3**–**5**, 72–91% yield). Substrates bearing a distal phenyl group exhibit selective HAA, favoring six-membered ring formation (**6**, 52% yield). Linear and cyclopentyl-bearing alkyl amines are also competent cyclization partners, providing the corresponding products in 50–86% yields (**7**–**10**). To bias the cyclization pathway toward six-membered ring formation, increasing both the disulfide loading and the reaction concentration proved effective, and these modified conditions delivered up to 82% selectivity for the six-membered product over the five- and seven-membered analogues (**10**, 82% yield). Beyond alkyl amines, aldehydes bearing diverse electronic properties and functionalities are compatible (**11**–**20**, 53–85% yield), including heterocyclic motifs, such as pyrazole (**19**, 54% yield). Notably, installing a *gem*-dimethyl group at C6 to block 6-endo cyclization allowed access to the 7-membered azepane scaffold (**21**, 48% yield, 95% selectivity).

**Table 2:**
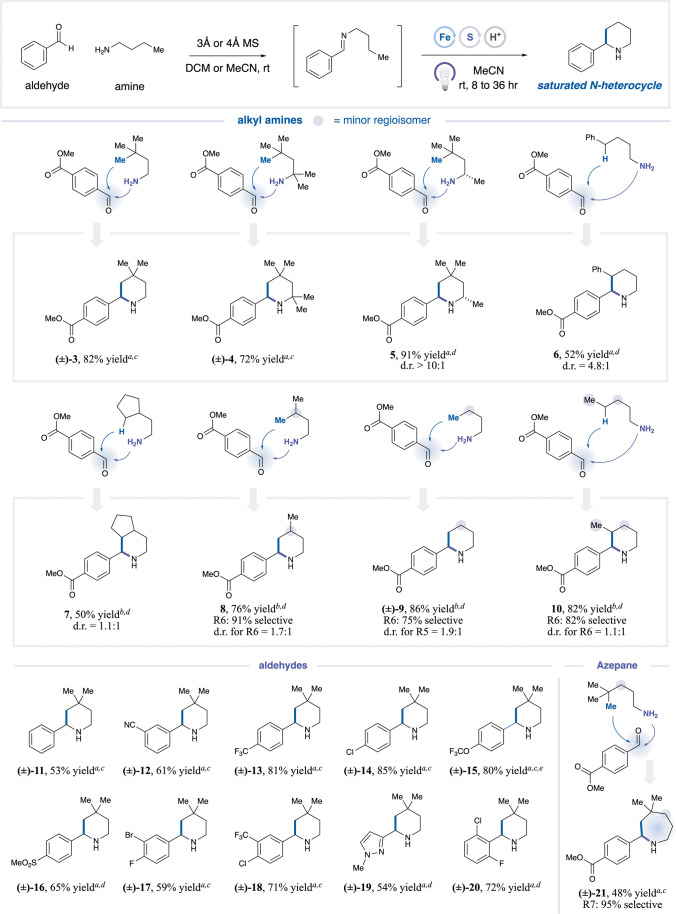
Piperidine Scope

a Isolated yields reported.

b Due to coelution of isomers, reported total assay yields for all cyclized regioisomers, R6 is the proportion for the desired six-membered regioisomer.

c General procedure A-1.

d General procedure A-2.

e Isolated as Boc-derivatized product.

We next examined the formation of morpholines and thiomorpholines, two widely represented scaffolds in medicinal chemistry (Table 3). In general, FeCl_3_ functions effectively as the HAA catalyst under standardized 420 nm IPR irradiation (see SI Section 4, **Part II**). However, for benzylic α-oxy C–H bond abstraction, TBADT [*E*([W_10_O_32_]^4–^/[W_10_O_32_]^5–^) = – 0.97 V vs SCE^42,50,51^ ] proved more effective, likely due to its steric bulk, which mitigates product inhibition at the tertiary α-oxy benzylic sites of the formed morpholines. As shown in Table 3, diverse substitution patterns are well tolerated on both methoxy- and thiol-functionalized amines. C–H bonds at methyl, methylene, methylthiol, ethylthiol, and α-oxy benzylic sites can be abstracted to exclusively generate the six-membered cyclized products. Versatile benzaldehydes with electron-donating or electron-withdrawing substituents are compatible (**22**–**33**, 48–65% yield), and heterocycles, such as pyridine, are also tolerated, albeit with a slight decrease in yield (**34**, 45% yield). Even sterically demanding aldehydes, including benzaldehydes bearing two ortho substituents, undergo efficient cyclization (**35**, 55% yield).

**Table 3:**
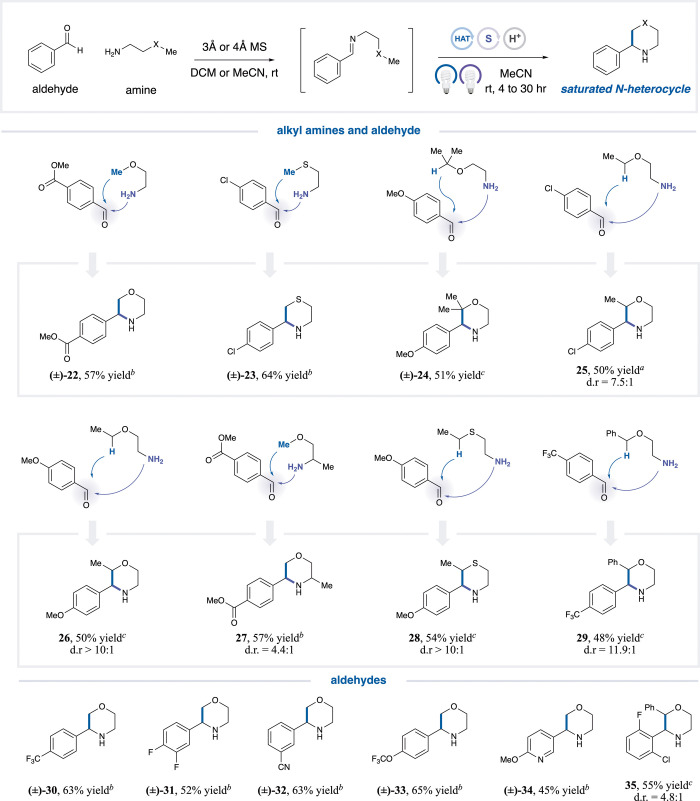
Morpholine and Thiomorpholine Scope^a^

a General procedure B-1.

b General procedure B-2.

c General procedure C-2. See SI for full experimental details.

d All yields are isolated.

To further demonstrate the versatility of this strategy, we expanded the scope beyond aryl aldehydes. Cyclopropanecarbaldehyde, cyclobutanecarbaldehyde, and pivalaldehyde were well tolerated (Table 4, **36**–**39**, 33–60% yield) to afford diverse poly substituted piperidines. Meanwhile, we employed bicyclo[1.1.1]­pentane (BCP)-derived aldehyde as a coupling partner, achieving facile incorporation of this valuable phenyl bioisostere^56^ into a saturated heterocyclic framework in good yield (**40**, 63% yield). Additionally, the use of serine-derived amines allowed introduction of chirality and rapid buildup of molecular complexity (**42**–**43**, 44–48% yield). Merging these motifs, we assembled a complex morpholine bearing BCP ester, phenyl, and derivatized serine in one flask with a 43% yield (**41**) [see SI section 16 for supplemental substrate scopes]. In addition to enabling access to a variety of saturated heterocycles with high regioselectivity, the examination of representative substrates revealed that the *trans* diastereomer was formed as the major product in all assigned cases. Furthermore, the use of bulkier TBADT as the HAA catalyst generally afforded higher diastereomeric ratios compared to FeCl_3_, likely due to the disfavored hydrogen atom abstraction after cyclization, particularly at the newly formed tertiary (benzylic/α-oxy) C–H bonds (see SI Section 14 for a detailed discussion of diastereoselectivity). Collectively, these examples demonstrate a modular and efficient strategy for the construction of diverse saturated heterocycles—piperidines, morpholines, and thiomorpholines—from native and readily available aldehydes and amines.

**Table 4:**
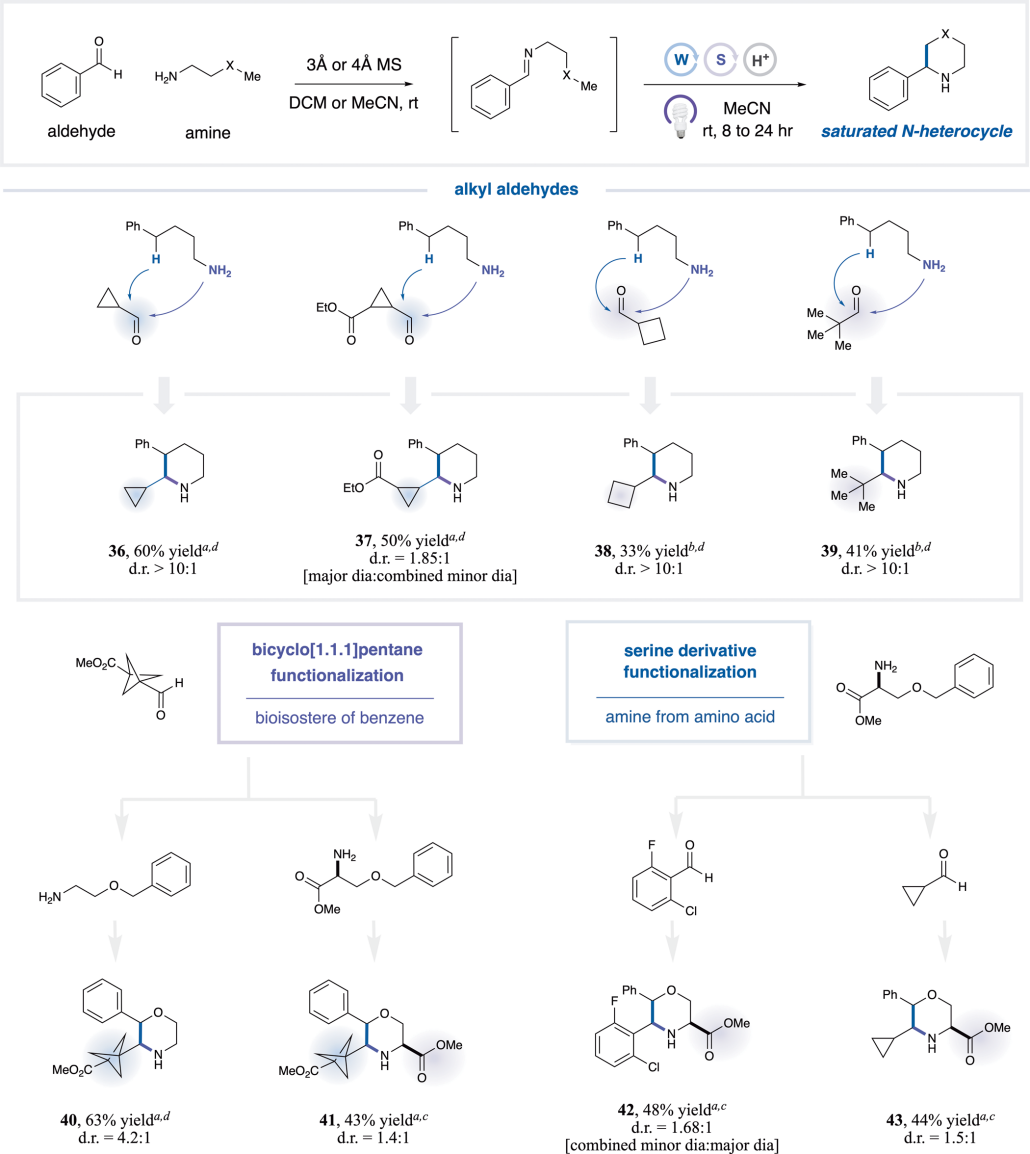
Saturated Heterocycle Formation with Bioactive Motifs

a Isolated yields reported.

b Assay yield due to the >10% yield loss in isolations.

c General procedure C-1.

d General procedure C-2. See SI for full experimental details.

We next sought to probe the mechanism—particularly the net reversibility of the paired HAA/HAD process and the role of acid in preventing product inhibition. Through deuterium-labeling, as shown in Table 5, the use of deuterated benzenesulfonic acid (62% deuteration, SI Figure S4) resulted in deuterium incorporation into model piperidine substrate **44**. Notably, deuterium incorporation was primarily observed at the terminal methyl groups (∼30% incorporation) with minor deuteration at other non-α-amino positions (<5% incorporation, SI Figure S5). High-resolution mass spectrometry (HRMS) further confirmed the presence of one to three deuterium atoms in the product (SI Figure S6). Collectively, these results implicate a reversible hydrogen atom transfer (HAT), consistent with a radical sampling process during the reaction. In the morpholine system, complete erosion of stereochemistry at the internal α-oxy methine site of **45** suggests reversible HAT at multiple positions. Together, these observations support the hypothesis that net reversibility is derived from HAA/HAD steps during the reaction. In contrast, strong acid protonation is known to deactivate α-amino C–H bonds toward HAA,^57–59^ which should preserve stereochemistry at nitrogen-adjacent centers. Indeed, chiral centers situated α- to nitrogen in both piperidine **5** and morpholine **46** retain >99% ee after the reaction, suggesting that protonation mitigates product inhibition and favors generation of the desired radical intermediates (see SI Section 7).

**Table 5:**
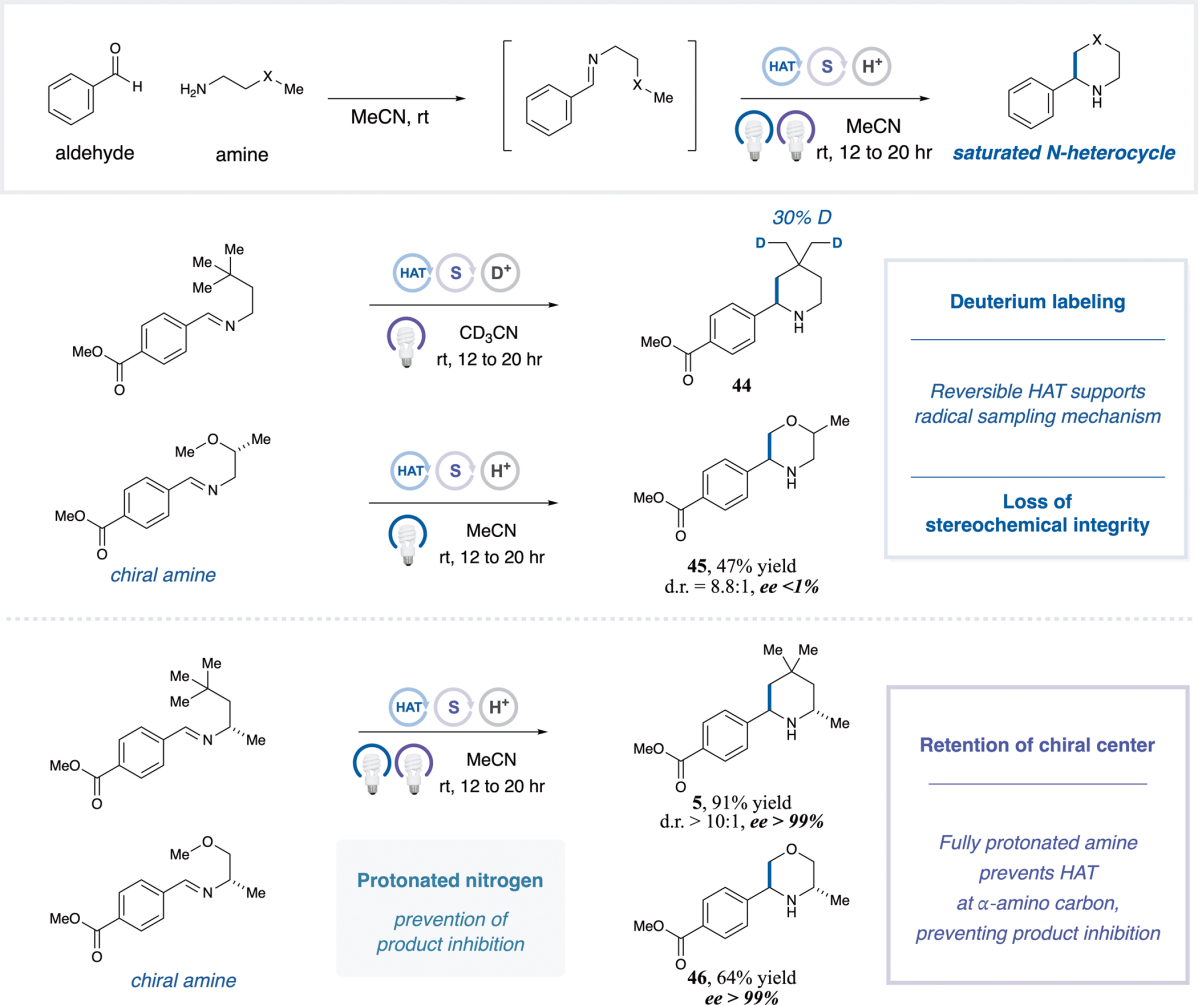
Mechanistic Investigation^a^

a Isolated yields reported. See SI for experimental details.

In conclusion, we describe a radical sampling-based strategy that enables the rapid and modular construction of piperidines, morpholines, and thiomorpholines directly from native and abundant aldehydes and amines using commercially available, inexpensive catalysts and reagents under an exceptionally straightforward reaction setup. Given the value of saturated heterocycles in medicinal chemistry, we anticipate that this transformation will find broad utility across the synthetic community.

## Supplementary Material



## Notes

Experimental details, expanded substrate table, compound characterization data, and spectra (PDF)
