# Supplementary material for: Radical Sampling Enabled Saturated N‑Heterocycle Cyclization
Source: J Am Chem Soc. 2026 Apr 8;148(15):15354–63. doi: 10.1021/jacs.6c01294 (PMC13107455; doi:10.1021/jacs.6c01294)
Supplement: Supplementary file 1 [file ja6c01294_si_001.pdf]

Supporting Information

Radical Sampling Enabled Saturated N-Heterocycle Cyclization

Qinyan Cai,[1]* Noah B. Bissonnette,[1]* Saegun Kim,[1] Thomas Knauber,[2] Gary M. Chinigo,[2] David C. Blakemore,[2] and David W. C. MacMillan[1†]

**These authors contributed equally to the work disclosed herein.*

†Corresponding author. Email: dmacmill@princeton.edu

1. Merck Center for Catalysis at Princeton University, Princeton, New Jersey 08544, United States
2. Pfizer Research and Development, Groton, CT 06340, United States

This PDF file includes:

Materials and Methods

Supplementary Text

Supplementary Substrate Scopes

Figures S1 to S15

Tables S1 to S11

References

Spectral Data

Table of Contents

1) General information S4

2) General considerations for N-heterocycle cyclization via radical sampling S5

3) Standard procedures used for reaction optimization S7

4) Reaction optimization/discussion and control experiments S8

5) UV-Vis study for $FeCl_3$ in the presence of reaction components S14

6) General procedures for N-heterocycle cyclization via radical sampling S15

7) Evidence for reversible HAT of radical sampling S21

8) Computations (DFT) S30

9) Proposed mechanism for TBADT mediated cyclization S63

10) Piperidine scope via radical sampling S64

11) (Thio)morpholine scope via radical sampling S83

12) Complex saturated heterocycle scope via radical sampling S97

13) Mechanistic substrate characterization S111

14) Diastereoselectivity and major diastereomer discussion S115

15) Structure elucidation of the side product from deuterium labeling studies S117

16) Supplemental substrate scopes **S124**

17) References **S125**

18) Spectral data **S127**

1) General information

All reagents purchased from commercial vendors were either used directly or purified following the procedures of Armarego and Perrin.[1] Solvents employed for starting material syntheses or photochemical reactions were purified using methods previously established by Grubbs.[2] All reagents in liquid states were transferred via syringe under either nitrogen or air. Photochemical reactions were typically irradiated using a 420 and 365 nm LED module housed within the PennOC/Penn PhD Integrated Photoreactor (a standardized reactor available for purchase through Sigma Aldrich).[3] Solutions containing organic solvents were concentrated *in vacuo* using a Büchi rotary evaporator (with attached 25-40 °C water bath). Purification of products via flash chromatography was generally achieved using automated Teledyne ISCO CombiFlash™ NextGen300+ or Biotage Isolera™ Spektra systems. Chromatography involving these systems featured Silicycle SiliaSep™, RediSep® Silver and Biotage® Sfär KP-Amino (KP-NH / KP-Amino) flash cartridges for normal-phase purifications or SiliaSep™ C18 cartridges for reverse-phase procedures. Alternatively, reverse-phase purifications could be performed via preparative High Performance Liquid Chromatography (prepHPLC) using a Teledyne ISCO ACCQPrep® HP150 system. These procedures employed either a Waters XBridge BEH C18 OBD Prep Column (30 mm x 150 mm, 130 Å, 5 μm) or a RediSep Prep C18 Column (30 mm x 250 mm, 100 Å, 5 μm), and compounds were eluted using H_2O/MeCN solutions buffered with 0.1% NH_4OH or formic acid modifier, respectively. ^1H and ^{13}C NMR spectra were collected using 300, 400 and 500 MHz Bruker NMR spectrometers and are referenced internally against residual signals from proteo-solvent [(1) acetone-d_6: 2.05 ppm, multiplicity 5, 2.84 ppm and 206.26 ppm, 29.84 ppm, multiplicity 7, respectively (2) acetonitrile-d_3: 1.94 ppm, multiplicity 5, 2.13 ppm and 118.26 ppm, 1.32 ppm, multiplicity 7, respectively (3) chloroform-*d*: 7.26 ppm and 77.16 ppm, multiplicity 3, respectively (4) methanol-d_4: 3.31 ppm, multiplicity 5, 4.87 ppm and 49.00 ppm, multiplicity 7, respectively (5) DMSO-d_6: 2.50 ppm, multiplicity 5, 3.30 ppm and 39.52 ppm, multiplicity 7]. ^{19}F NMR spectra were collected using a 400 MHz Bruker NanoBay Avance III HD NMR spectrometer and are not internally referenced. For ^{13}C NMR, data are typically reported in terms of chemical shift only (multiplicity and coupling constants are only utilized when showcasing coupling with relevant ^{19}F nuclei). For ^1H and ^{19}F NMR, all reported data includes the following parameters: chemical shift (δ ppm), multiplicity (s = singlet, d = doublet, t = triplet, q = quartet, m = multiplet, br = broad), coupling constant (Hz), and integration. Agilent 1290 Infinity II LC systems were employed for compound analysis via liquid chromatography (LC). IR spectra are reported in wavenumbers (cm^{-1}) based on measurements recorded with a Perkin Elmer Spectrum 100 FTIR spectrometer. For mass spectrometry (MS) analysis, high resolution mass spectra were obtained using Agilent 6220 ESI-TOF LC/MS or Agilent GC-QTOF instrumentation available in the Princeton University Mass Spectral Facility.

2) General considerations for N-heterocycle cyclization via radical sampling

1: **Photoreactor setup** – All cyclizations via radical sampling were conducted according to optimal procedures and irradiated using a PennOC/PennPhD Integrated Photoreactor (IPR).[3] For these experiments, 365 and 420 nm UV-blue light LED modules were utilized, with preference for M2 photoreactors/LED plates. Across all examples, 100% LED intensity, 200-500 rpm stir rate and 6800 rpm fan speed were used as default settings. N-heterocycle cyclizations were typically complete after 12-20 hours of irradiation. However, deviations to these irradiation times were determined based on specific aldehyde and amine substrate pairings and are reported in the procedural descriptions accompanying each example. When performing reactions according to previously standardized IPR guidelines,[3] each reaction vessel is typically maintained near room temperature for the duration of irradiation.

**Note: For small-scale reactions (0.1 mmol), four reactions can be performed simultaneously within the IPR using customized "multi-vial" holders. For on-scale reactions (0.3 mmol to 0.5 mmol), the cyclization is performed with a single reaction vial per photoreactor.*

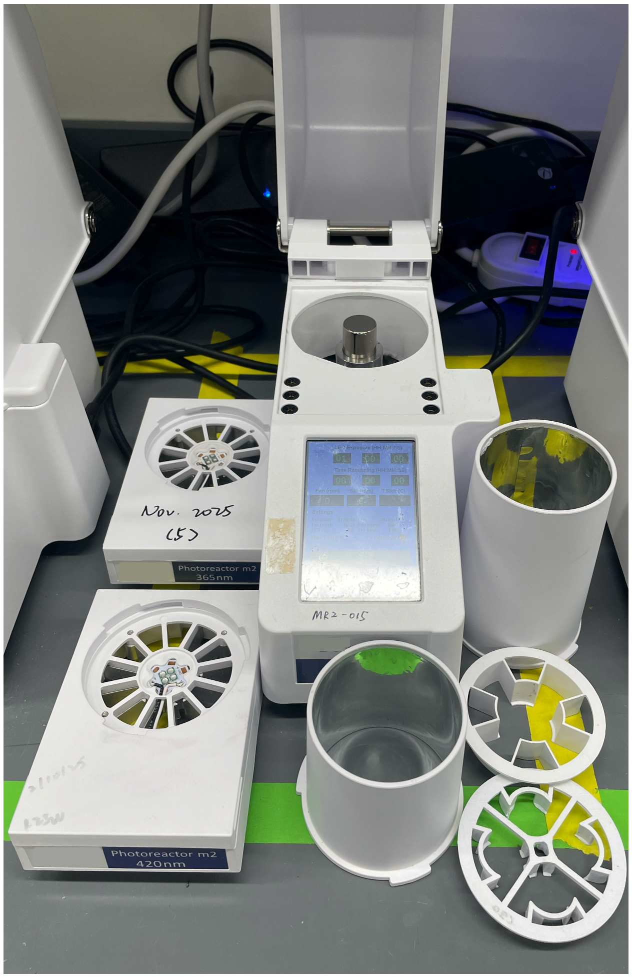

Figure S1. Standard Integrated Photoreactor assembly used in N-heterocycle cyclization.

2: **Imine condensation consideration** – The cyclization can be carried out with preformed, isolated imine or *in-situ* generated imine. There is ***little to no difference*** in yields for the cyclization across both protocols. For both preformed and *in-situ* generated imine, the aldehyde is typically the limiting reagent, and 1.10 equiv. of amine is added. Molecular sieves (3Å or 4Å) were used for both conditions. Molecular sieves in *ball-form* are preferred over mesh- or pellet-form to avoid the light filtering effect during the irradiation. Preformed imine, condensed in dichloromethane (DCM), was generally used for optimization and some reaction scope substrates. *In-situ* generated imine, condensed in acetonitrile (MeCN, reaction solvent), was only used for reaction scope.

3: **Reaction setup, degassing considerations** – Depends on the substrate classes, the optimal N-heterocycle cyclization protocol employs both air free and open-to-air setups. For all piperidine formation as well as (thiol)morpholine formation utilizing tetra-*n*-butylammonium decatungstate (TBADT) as the catalyst, air-free setup is preferred. Therefore, using standard inlet needle/vent needle sparging procedures previously developed for photoredox applications,[5] five-minute N_2 in-solution sparge times are recommended for optimal performance (during the sparging, placing vials in the ice bath can effectively prevent solvent evaporation). For the remaining (thiol)morpholine cyclization, air is tolerated and makes no difference. All the reagents apart from benzenesulfonic acid can be weighed under air. Benzenesulfonic acid was stored and weighed inside of the glovebox (quite hygroscopic). For iron(III) chloride, $FeCl_3$, the reagent bottle may contain both black solids and yellow solids due its hygroscopicity. Only black solids are used and directly weighing $FeCl_3$ into vials is recommended. For some cases using trifluoroacetic acid (TFA) instead of benzenesulfonic acid, TFA is added *after* the sparging to avoid the evaporation of the liquid acid during the sparging.

3: **Assay considerations** – For cyclization utilizing $FeCl_3$ as the HAT catalyst and benzenesulfonic acid as the acid, the resulting reaction mixture normally contains precipitates after irradiation. Therefore, adding water dropwise until the reaction mixture is homogenous is crucial for accurate assay yields.

4: **Common observations of mass balance** – In general, heterocycle cyclization is efficient to form the desired 6-member rings. However, in addition to desired product, several additional compounds may be detectable following standard heterocycle cyclization procedures. First, remaining imine or the hydrolyzed aldehyde (especially with addition of water) can be detected in certain cases. Second, the reduced, linear byproduct without successful cyclization can be detected in certain cases especially for piperidine formation. This byproduct may potentially arise by (i) excitation of imine C–N double bond (highly conjugated imines) and subsequent HAT from a thiol/solvent or (ii) ground state reduction of the imine C–N double bond and HAT/proton transfer steps. Third, chlorinated cyclized product can be detected when the irradiation time exceeds the ideal length. Intermediates may be generated via HAT at C—H bonds of cyclized products and quenched by chlorine radical.

3) Standard procedures used for reaction optimization

Typical procedure for optimization/control experiments (on 0.1 mmol scale):

Imine condensation: To a 100-mL round-bottom flask (RBF), 6 mmol of aldehyde was added and dissolved in ~35 mL of DCM. Then, 1.5 g molecular sieves (3Å or 4Å) were added into the flask. Lastly, 1.1 equiv of amine was added. After 12 hours, the mixture was filtered through celite and concentrated *in vacuo* to afford pure imine with quantitative yield.

Reagent vial: An oven-dried 8-mL vial was charged with benzenesulfonic acid (4 equiv) in the glovebox. Then, the reagent vial was capped tightly and transferred outside of the glovebox. $FeCl_3$ (20 mol%) was then directly weighed into the reagent vial, followed by addition of pre-weighed bis(4-methoxyphenyl) disulfide (30 mol%) under air. All transfers and weighing were completed under ~1 min to minimize absorption of exogenous water in the atmosphere.

Reaction vial: (*E*)-4-(((3,3-dimethylbutyl)imino)methyl)benzoate (0.1 mmol) was directly weighed into a second 8-mL vial via syringe.

The reagent vial was then added 0.3 mL of MeCN to fully dissolve the reagents. The mixture was then transferred to the reaction vial. Then, 0.2 mL of MeCN was added to rinse the reagent vial. The mixture was again transferred to the reaction vial. The reaction was sparged in solution with a vent needle under N_2 for 3 minutes on ice (to minimize evaporation of the solvent). Then, the reaction mixture was sealed with parafilm and subjected to IPR irradiation using 365 nm LED modules at 100% light intensity with maximum fan speed (6800 rpm) and 500 rpm stir rate for 12 hours. After this time, water was added dropwise to fully solubilize the crude mixture into a homogeneous solution. Then, 1,4-dinitrobenzene was added as an internal standard, and an aliquot was removed for NMR analysis.

4) Reaction optimization/discussion and control experiments

Part I: Reaction optimization and control experiments

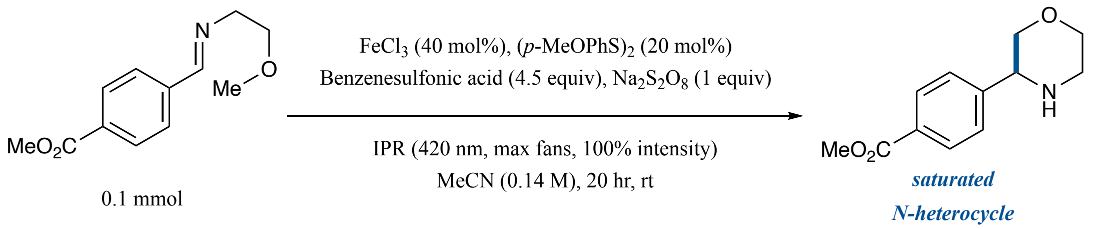

entry	deviation	yield
1	none	55%
2	no $FeCl_3$	0%
3	no (*p*-MeOPhS)$_2$	40%
4	no benzenesulfonic acid	0%
5	0.5 mmol without $Na_2S_2O_8$	43%
6	0.5 mmol with $Na_2S_2O_8$	64%
7	no light	0%

Table S1. Control experiments for (thio)morpholine cyclization

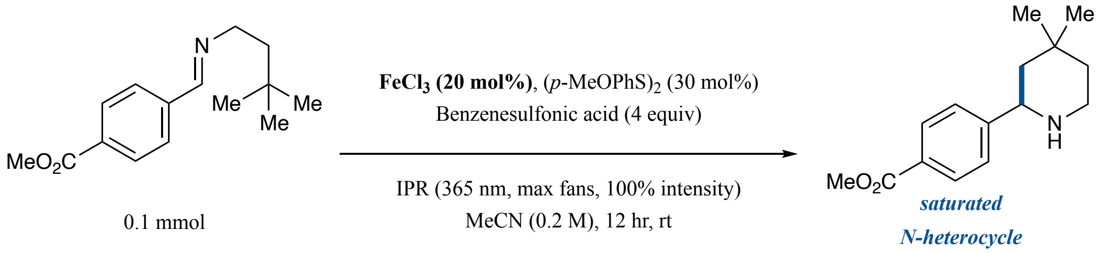

entry	deviation	yield^b
1	**$FeCl_3$**	71%
2	$CeCl_3$, TBACl	<1%
3	$CuCl_2$	21%
4	TBADT	44%
5	Benzophenone	<1%

Table S2. Evaluation of HAT catalysts for piperidine cyclization

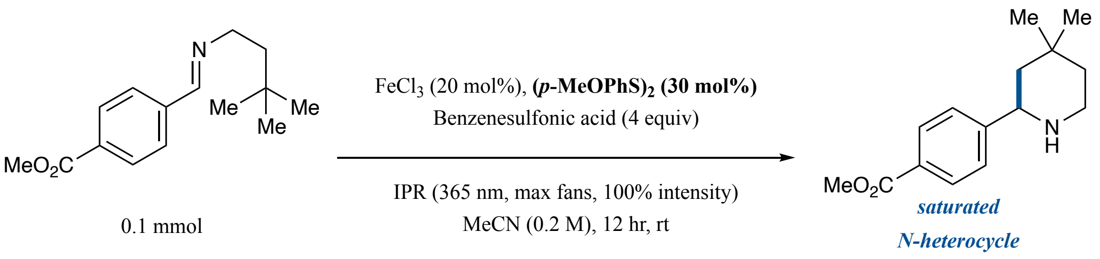

entry	deviation	yield[b]
1	**$(p\text{-MeOPhS})_2$**	71%
2	$(PhS)_2$	48%
3	(p-ClPhS)$_2$	39%
4	(p-MePhS)$_2$	38%
5	(iPrPhS)$_2$	53%

Table S3. Evaluation of disulfide sources for piperidine cyclization

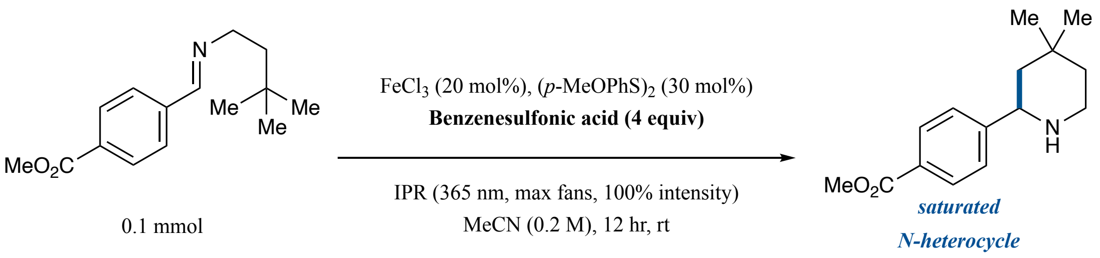

entry	deviation	yield[b]
1	**Benzenesulfonic acid**	71%
2	TFA	35%
3	Triflic acid	0%
4	4-Chlorobenzenesulfonic acid	22%
5	4-Methoxybenzenesulfonic acid	33%

Table S4. Evaluation of acids for piperidine cyclization

Part II: Reaction optimization discussion

[1] Loading of $FeCl_3$

For morpholine/thiomorpholine substrate class (40 mo% $FeCl_3$), a higher loading of $FeCl_3$ is employed compared to piperidine substrate class (20 mol% $FeCl_3$) for two potential reasons. (1) Based on the reaction condition, (thio)morpholine scope employs 420 nm lights instead of the 365 nm lights. According to the UV-Vis study (**Figure S3**), $FeCl_3$ has a weaker absorption at 420 nm compared to 365 nm. Therefore, higher loading of $FeCl_3$ may allow higher concentration of generated chlorine radical at a given time. (2) The activated α-oxy/α-thiol secondary C–H bonds are present in both starting iminium and cyclized products across all of the morpholine and thiomorpholine substrates. Therefore, higher loading of the $FeCl_3$ is necessary to enable sufficient abstracting at the desired terminal α-oxy/α-thiol primary C–H bonds.

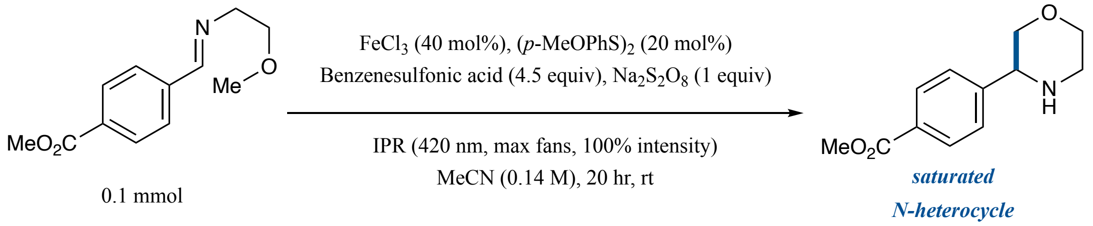

Plates/$FeCl_3$	10 mol%	15 mol%	20 mol%	30 mol%	40 mol%
420 nm	**31% cyclized**	**35% cyclized**	**33% cyclized**	**40% cyclized**	**47% cyclized**

Plates/$FeCl_3$	40 mol%	50 mol%	60 mol%	80 mol%	1 equiv
420 nm	**45% cyclized**	**44% cyclized**	**45% cyclized**	**46% cyclized**	**44% cyclized**

Table S5. $FeCl_3$ loading optimization *(during optimization campaign, not with the optimal conditions).*

[2] 420 nm light *vs* 365 nm light for (thio)morpholine scope

For (thio)morpholine scope, 420 nm light is utilized rather than 365 nm. As shown by **Figure S2**, during the optimization campaign, cyclization was demonstrated to be successful under both wavelengths. In particular, employing General Procedure A-1/A-2 for (thio)morpholine scope can obtain cyclized product with 35-45% yield (10-20% lower compared to standard General Procedure B-1/B-2). However, the mass balance was improved greatly with 420 nm light, which has a lower energy compared to 365 nm light. The potential decomposition of the starting iminium

ion under 365 nm may result from the presence of the activated α-oxy/α-thiol C–H bonds. The decomposition of the starting iminium under 365 nm light is further supported by the studies in **Figure S2.**

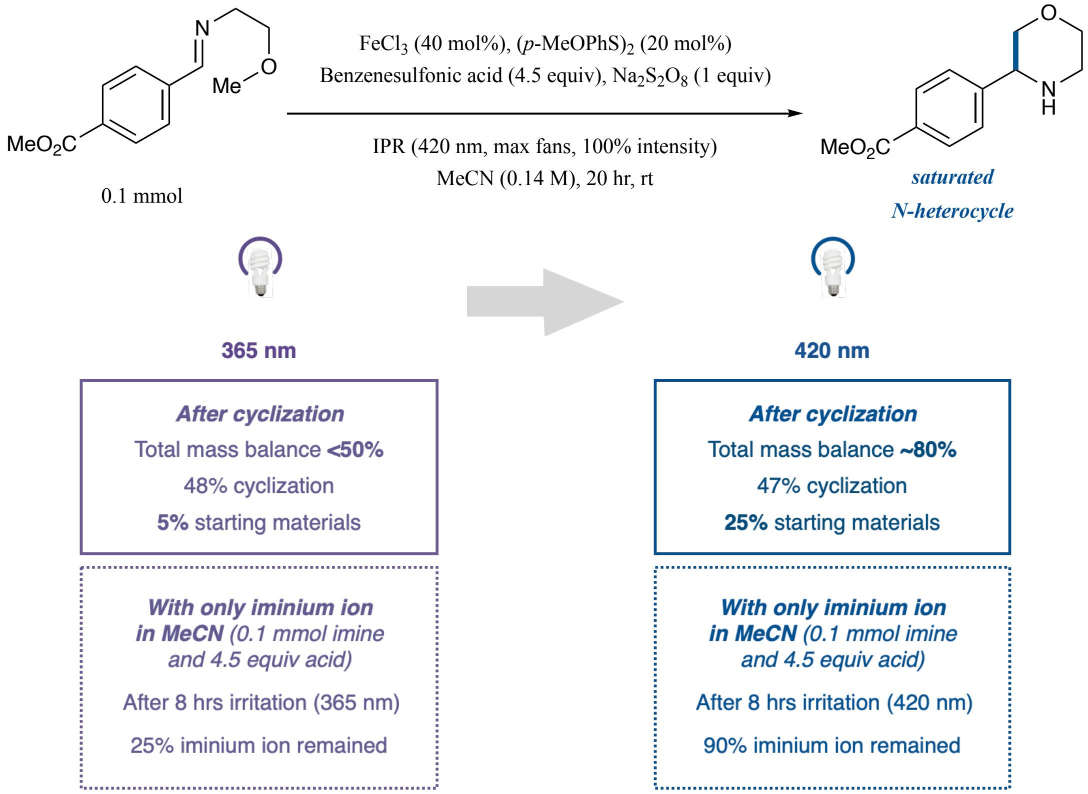

Figure S2. Wavelength optimization *(during optimization campaign, not with the optimal conditions).*

[3] $Na_2S_2O_8$ as the additive for morpholine scope

For *on-scale* reactions (0.5 mmol), morpholine scope employs $Na_2S_2O_8$ as the additive. For small scale reactions (0.1 mmol), the addition of the oxidant does not make a huge difference in product yield. The oxidant in the on-scale reactions may function in two different ways: (1) it may facilitate the oxidation of the Fe^{II} to Fe^{III} after LMCT to turn over the catalytic cycle more efficiently. (2) oxyl radical from the oxidant may also involve in hydrogen atom abstraction. The employment of the oxidant in $FeCl_3$ chemistry is also precedented. The following publication also employs a similar combination of $FeCl_3$ and oxidant (^{t}BuOO^{t}Bu) to complete the catalytic cycle.[15]

[4] Potential chloride anion-containing additive for $FeCl_3$ system

Versatile chloride anion additives were tested during the optimization campaign to potentially facilitate the LMCT of $FeCl_3$. In particular, under 365 nm light, different additives were tried on (thio)morpholine scope to improve the yield with a lower $FeCl_3$ loading (**Table S6**). However, none of conditions with additional chloride anion source demonstrated better reaction performance than the condition without chloride anion additive during the optimization campaign (**Table S6**).

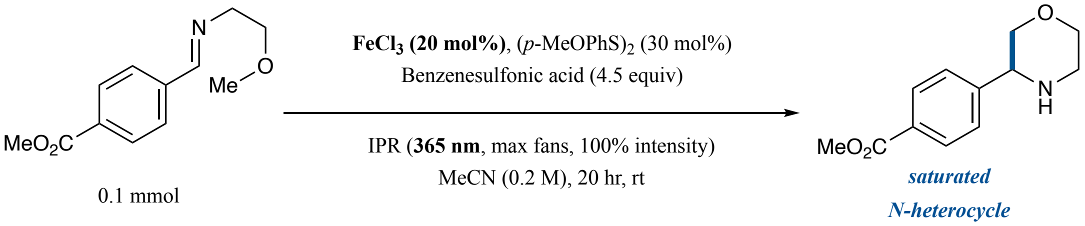

Additive	Assay
None	**44% cyclized**<br>1% starting materials
HCl	**40% cyclized**<br>2% starting materials
LiCl	**30% cyclized**<br>2% starting materials
TBACl	**<10% cyclized**<br><5% starting materials

Table S6. Additive optimization under 365 nm *(during optimization campaign, not with the optimal conditions).*

Additionally, after switching to 420 nm light for (thio)morpholine, TBACl and other chloride anion containing additives were tested again for possibility of lowering the loading of $FeCl_3$. However, the results were consistent with previous findings that no improved catalytic reactivities were observed with addition of additives.

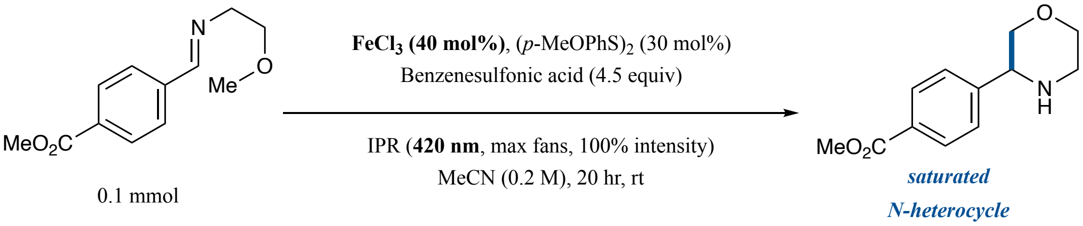

Additive	Assay
None	**47% cyclized**<br>>20% starting materials
0.5 equiv TBACl	**19% cyclized**<br>34% starting materials

0.8 equiv TBACl	**17% cyclized**<br>47% starting materials
1.5 equiv TBACl	**9% cyclized**<br>45% starting materials
0.8 equiv LiCl	**44% cyclized**<br>20% starting materials

Table S7. Additive optimization under 420 nm light *(during optimization campaign, not with the optimal conditions)*.

5) UV-Vis study for $FeCl_3$ in the presence of reaction components

UV-Vis study was carried out with four different combinations of reaction components in reaction solvent, MeCN. For each combination, the stock solutions were employed and prepared based on actual reaction conditions to add different components, ensuring the consistent concentrations of different reagents across different combinations. Four solutions were further diluted by the same factor for UV-Vis scans. Four combinations include: (1) $FeCl_3$ only; (2) $FeCl_3$ and standard imine; (3) $FeCl_3$ and benzenesulfonic acid; (4) $FeCl_3$, benzenesulfonic acid and standard imine.

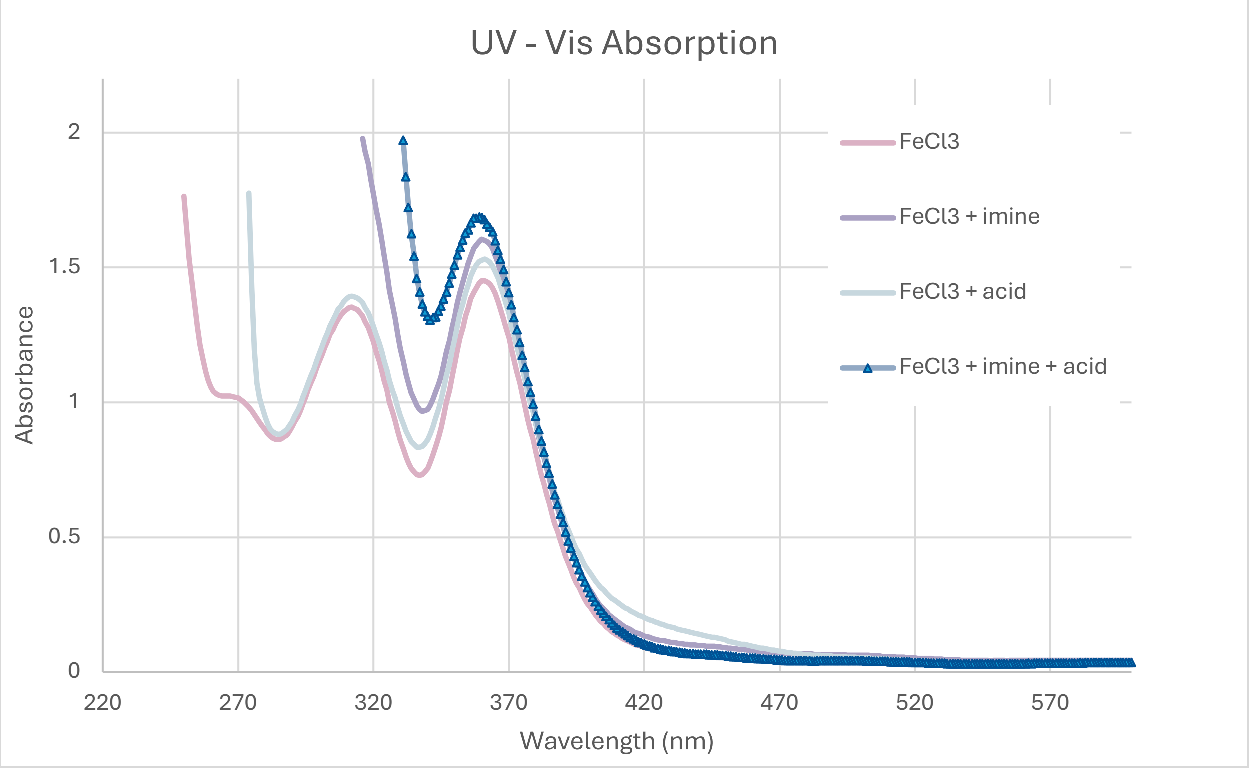

Figure S3. UV-Vis studies.

Based on the UV-Vis (**Figure S3**), the addition of acid or/and imine didn't change the strong absorption at 365 nm of $FeCl_3$. In the presence of both acid and imine, the absorbance at 365 nm is even improved slightly but without a major difference. Based on the mechanistic proposal, the primary role of acid is to (i) increase the imine's electrophilicity to enable a more favorable cyclization from the nucleophilic carbon-centered radical and (ii) "protect" the thermodynamically weak but polarity-mismatched alpha ammonium C–H bonds from abstraction.

6) General procedures for N-heterocycle cyclization via radical sampling

General Procedure A-1 (*in-situ* imine): piperidine cyclization via HAT and radical sampling (0.5 mmol scale)

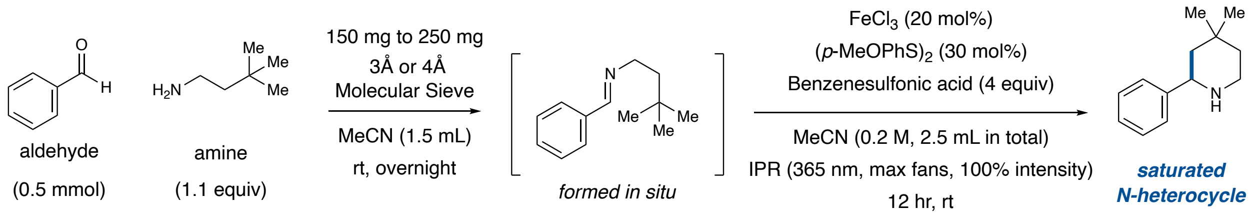

<u>**Reaction vial** (*In-situ* imine condensation):</u> To an oven-dried 40-mL vial, aldehyde (0.5 mmol, 1 equiv) was added, followed by addition of 150-250 mg molecular sieves (3Å or 4Å). The mixture was then added 1.5 mL of anhydrous MeCN to fully dissolve the aldehyde. Amine (0.55 mmol, 1.1 equiv) was added last to the reaction mixture. After capping the vial, the reaction was stirred at 100 rpm (to avoid breaking the molecular sieves) overnight under air.

<u>**Reagent vial**:</u> An oven-dried 8-mL vial was charged with benzenesulfonic acid (316.4 mg, 2.0 mmol, 4 equiv) in the glovebox. Then, the <u>reagent vial</u> was capped tightly and transferred outside of the glovebox. $FeCl_3$ (16.2 mg, 0.1 mmol, 20 mol%) was then directly weighed into the <u>reagent vial</u>, followed by addition of pre-weighed bis(4-methoxyphenyl) disulfide (41.8 mg, 0.15 mmol, 30 mol%) under air. All transfers and weighing were completed under ~1 min to minimize absorption of exogenous water in the atmosphere.

After overnight imine condensation, the <u>reagent vial</u> was added 0.5 mL of MeCN to fully dissolve the reagents. The mixture was transferred to the <u>reaction vial</u>. Then, 0.5 mL of MeCN was added to rinse the <u>reagent vial</u>. The mixture was again transferred to the reaction vial. The reaction was sparged in solution with a vent needle under N_2 for 5 minutes on ice. Then, the reaction mixture was subjected to IPR irradiation using 365 nm LED modules at 100% light intensity with maximum fan speed (6800 rpm) and 500 rpm stir rate for 12-36 hours. After this time, water was added dropwise to fully solubilize the crude mixture into a homogeneous solution. Then, 1,4-dinitrobenzene was added as an internal standard, and an aliquot was removed for NMR analysis. Subsequently, the reaction mixture was basified by saturated $NaHCO_3$ solution and poured into 20 mL of saturated EDTA solution and transferred to a separatory funnel. The reaction vial was rinsed into the separatory funnel with 1:1 EtOAc/water mixture (3 x 10 mL), and the layers separated. After extracting the aqueous layer three times with EtOAc (3 x 15 mL), the aqueous layer was added 1M NaOH solution and the pH was checked by pH indicators to be greater than 10. The aqueous solution was then extracted one last time with EtOAc (15 mL), the combined extracts were dried over Na_2SO_4, filtered, and concentrated *in vacuo*. Depending on the identity of the cyclized product, the residue was subjected to different purification methods [(1) Biotage® Sfär KP-Amino (KP-NH / KP-Amino) Flash Cartridges; (2) acid/base wash; (3) prepHPLC or reverse phase chromatography] to afford pure cyclized products.

General Procedure A-2 (preformed imine): piperidine cyclization via HAT and radical sampling (0.5 mmol scale)

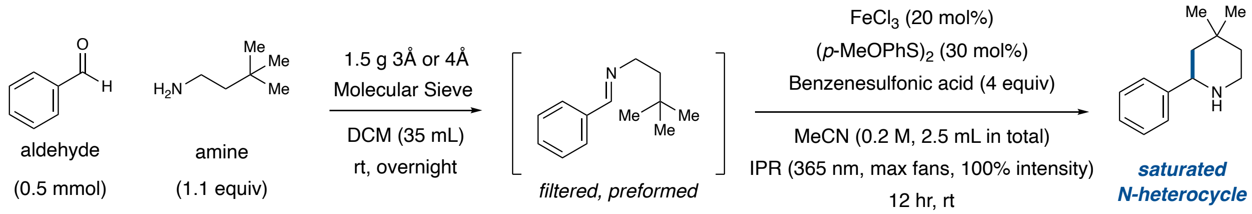

Preformed imine: To a 100-mL round-bottom flask (RBF), aldehyde (6 mmol, 1 equiv) was added and dissolved in ~35 mL of DCM. Then, 1.5 g molecular sieves (3Å or 4Å) were added into the flask. Lastly, amine (6.6 mmol, 1.1 equiv) was added into the RBF. After 12 hours, the mixture was filtered through celite and concentrated *in vacuo* to afford pure imine in quantitative yield.

Reagent vial: An oven-dried 8-mL vial was charged with benzenesulfonic acid (316.4 mg, 2.0 mmol, 4 equiv) in the glovebox. Then, the reagent vial was capped tightly and transferred outside of the glovebox. $FeCl_3$ (16.2 mg, 0.1 mmol, 20 mol%) was then directly weighed into the reagent vial, followed by addition of pre-weighed bis(4-methoxyphenyl) disulfide (41.8 mg, 0.15 mmol, 30 mol%) under air. All transfers and weighing were completed under ~1 min to minimize absorption of exogenous water in the atmosphere.

Reaction vial: An oven-dried 40-mL vial was charged with imine (0.5 mmol, 1 equiv) under air.

The reagent vial was then added 1 mL of MeCN to fully dissolve the reagents. The mixture was then transferred to the reaction vial. Then, 1.5 mL of MeCN was added to rinse the reagent vial. The mixture was again transferred to the reaction vial. The reaction was sparged in solution with a vent needle under N_2 for 5 minutes on ice (preventing the evaporation of the solvent). Then, the reaction mixture was subjected to IPR irradiation using 365 nm LED modules at 100% light intensity with maximum fan speed (6800 rpm) and 500 rpm stir rate for 12-36 hours. After this time, water was added dropwise to fully solubilize the crude mixture into a homogeneous solution. Then, 1,4-dinitrobenzene was added as an internal standard, and an aliquot was removed for NMR analysis. Subsequently, the reaction mixture was basified by saturated $NaHCO_3$ solution and poured into 20 mL of saturated EDTA solution and transferred to a separatory funnel. The reaction vial was rinsed into the separatory funnel with 1:1 EtOAc/water mixture (3 x 10 mL), and the layers separated. After extracting the aqueous layer three times with EtOAc (3 x 15 mL), the aqueous layer was added 1M NaOH solution and the pH was checked by pH indicators to be greater than 10. The aqueous solution was then extracted one last time with EtOAc (15 mL), the combined extracts were dried over Na_2SO_4, filtered, and concentrated *in vacuo*. Depending on the identity of the cyclized product, the residue was subjected to different purification methods [(1) Biotage® Sfär KP-Amino (KP-NH / KP-Amino) Flash Cartridges; (2) acid/base wash; (3) prepHPLC or reverse phase chromatography] to afford pure cyclized products.

General Procedure B-1 (*in-situ* imine): (thio)morpholine cyclization via HAT and radical sampling (0.5 mmol scale)

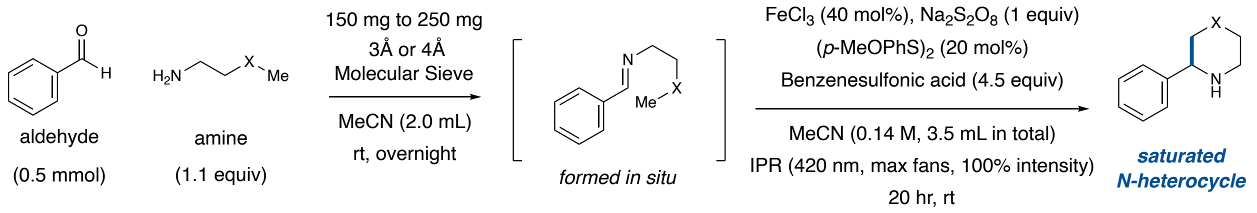

<u>Reaction vial</u> <u>(*In-situ* imine condensation):</u> To a 40-mL vial, aldehyde (0.5 mmol, 1 equiv) was added, followed by addition of 150-250 mg molecular sieves (3Å or 4Å). The mixture was then added 2 mL of anhydrous MeCN to fully dissolve the aldehyde. Amine (0.55 mmol, 1.1 equiv) was added last to the reaction mixture. After capping the vial, the reaction was stirred at 100 rpm (to avoid breaking the molecular sieves) overnight under air.

<u>Reagent vial</u>: An 8-mL vial was charged with benzenesulfonic acid (355.9 mg, 2.25 mmol, 4.5 equiv) in the glovebox. Then, the <u>reagent vial</u> was capped tightly and transferred outside of the glovebox. $FeCl_3$ (32.4 mg, 0.2 mmol, 40 mol%) was then directly weighed into the <u>reagent vial</u>, followed by addition of pre-weighed bis(4-methoxyphenyl) disulfide (27.8 mg, 0.1 mmol, 20 mol%) under air. All transfers and weighing were completed under ~1 min to minimize absorption of exogenous water in the atmosphere.

<u>Reaction vial</u> <u>(after imine condensation):</u> After overnight condensation, the <u>reaction vial</u> was added sodium persulfate $Na_2S_2O_8$ (0.5 mmol, 119.1 mg, 1 equiv) under air.

Then, the <u>reagent vial</u> was then added 1 mL of MeCN to fully dissolve the reagents. The mixture was then transferred to the <u>reaction vial</u>. Then, 0.5 mL of MeCN was added to rinse the <u>reagent vial</u>. The mixture was again transferred to the reaction vial. Then, the reaction mixture was subjected to IPR irradiation using 420 nm LED modules at 100% light intensity with maximum fan speed (6800 rpm) and 500 rpm stir rate for 20 hours. After this time, water was added dropwise to fully solubilize the crude mixture into a homogeneous solution. Then, 1,4-dinitrobenzene was added as an internal standard, and an aliquot was removed for NMR analysis. Subsequently, the reaction mixture was basified by saturated $NaHCO_3$ solution and poured into 20 mL of saturated EDTA solution and transferred to a separatory funnel. The reaction vial was rinsed into the separatory funnel with 1:1 EtOAc/water mixture (3 x 10 mL), and the layers separated. After extracting the aqueous layer three times with EtOAc (3 x 15 mL), the aqueous layer was added 1M NaOH solution and the pH was checked by pH indicators to be greater than 10. The aqueous solution was then extracted one last time with EtOAc (15 mL), the combined extracts were dried over Na_2SO_4, filtered, and concentrated *in vacuo.* Depending on the identity of the cyclized product, the residue was subjected to different purification methods [(1) Biotage® Sfär KP-Amino (KP-NH / KP-Amino) Flash Cartridges; (2) acid/base wash; (3) prepHPLC or reverse phase chromatography] to afford pure cyclized products.

General Procedure B-2 (preformed imine): (thio)morpholine cyclization via HAT and radical sampling (0.5 mmol scale)

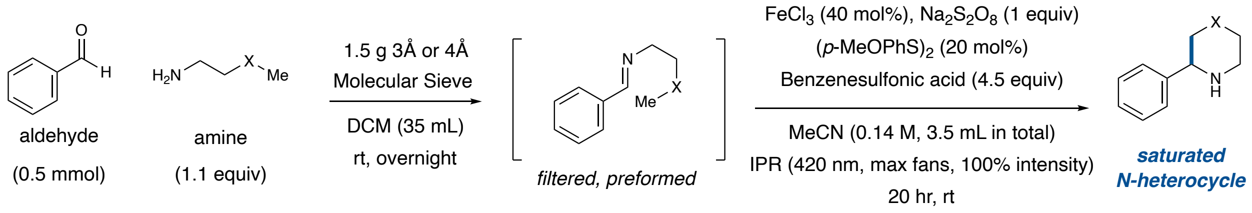

<u>**Preformed imine**</u>: To a 100-mL round-bottom flask (RBF), aldehyde (6 mmol, 1 equiv) was added and dissolved in ~35 mL of DCM. Then, 1.5 g molecular sieves (3Å or 4Å) were added into the flask. Lastly, amine (6.6 mmol, 1.1 equiv) was added into the RBF. After 12 hours, the mixture was filtered through celite and concentrated *in vacuo* to afford pure imine with quantitative yield.

<u>**Reagent vial**</u>: An 8-mL vial was charged with benzenesulfonic acid (355.9 mg, 2.25 mmol, 4.5 equiv) in the glovebox. Then, the <u>reagent vial</u> was capped tightly and transferred outside of the glovebox. $FeCl_3$ (32.4 mg, 0.2 mmol, 40 mol%) was then directly weighed into the <u>reagent vial</u>, followed by addition of pre-weighed bis(4-methoxyphenyl) disulfide (27.8 mg, 0.1 mmol, 20 mol%) under air. All transfers and weighing were completed under ~1 min to minimize absorption of exogenous water in the atmosphere.

<u>**Reaction vial**</u>: The <u>reaction vial</u> was added imine (0.5 mmol, 1 equiv) and sodium persulfate $Na_2S_2O_8$ (0.5 mmol, 119.1 mg, 1 equiv) under air.

The <u>reagent vial</u> was then added 2 mL of MeCN to fully dissolve the reagents. The mixture was then transferred to the <u>reaction vial</u>. Then, 1.5 mL of MeCN was added to rinse the <u>reagent vial</u>. The mixture was again transferred to the reaction vial. Then, the reaction mixture was subjected to IPR irradiation using 420 nm LED modules at 100% light intensity with maximum fan speed (6800 rpm) and 500 rpm stir rate for 20 hours. After this time, water was added dropwise to fully solubilize the crude mixture into a homogeneous solution. Then, 1,4-dinitrobenzene was added as an internal standard, and an aliquot was removed for NMR analysis. Subsequently, the reaction mixture was basified by saturated $NaHCO_3$ solution and poured into 20 mL of saturated EDTA solution and transferred to a separatory funnel. The reaction vial was rinsed into the separatory funnel with 1:1 EtOAc/water mixture (3 x 10 mL), and the layers separated. After extracting the aqueous layer three times with EtOAc (3 x 15 mL), the aqueous layer was added 1M NaOH solution and the pH was checked by pH indicators to be greater than 10. The aqueous solution was then extracted one last time with EtOAc (15 mL), the combined extracts were dried over Na_2SO_4, filtered, and concentrated *in vacuo*. Depending on the identity of the cyclized product, the residue was subjected to different purification methods [(1) Biotage® Sfär KP-Amino (KP-NH / KP-Amino) Flash Cartridges; (2) acid/base wash; (3) prepHPLC or reverse phase chromatography] to afford pure cyclized products.

General Procedure C-1 (*in-situ* imine): piperidine/morpholine cyclization from activated amine/electron-rich aldehyde via HAT and radical sampling (0.5 mmol scale)

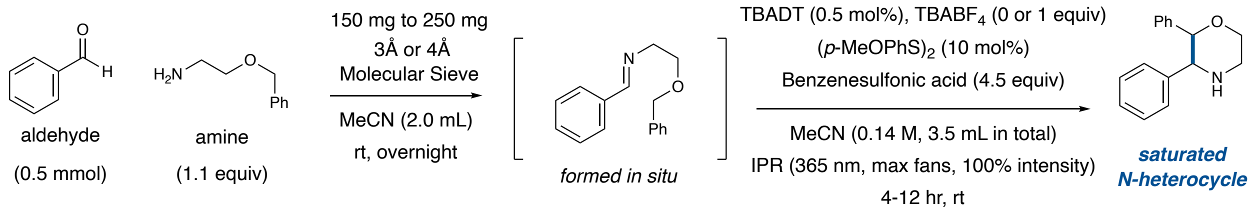

<u>**Reaction vial**</u> <u>(*In-situ* imine condensation):</u> To a 40-mL vial, aldehyde (0.5 mmol, 1 equiv) was added, followed by addition of 150-250 mg molecular sieves (3Å or 4Å). The mixture was then added 2 mL of anhydrous MeCN to fully dissolve the aldehyde. Amine (0.55 mmol, 1.1 equiv) was added last to the reaction mixture. After capping the vial, the reaction was stirred at 100 rpm (to avoid breaking the molecular sieves) overnight under air.

<u>**Reagent vial**:</u> An 8-mL vial was charged with benzenesulfonic acid (355.9 mg, 2.25 mmol, 4.5 equiv) in the glovebox. Then, the <u>reagent vial</u> was capped tightly and transferred outside of the glovebox. TBADT (3.7 mg, 0.0025 mmol, 0.5 mol%), bis(4-methoxyphenyl) disulfide (13.9 mg, 0.05 mmol, 10 mol%) and tetrabutylammonium tetrafluoroborate $TBABF_4$ (164.6 mg, 0.5 mmol, 1 equiv) were weighed into the <u>reagent vial</u> under air.

After overnight imine condensation, the <u>reagent vial</u> was then added 1 mL of MeCN to fully dissolve the reagents. The mixture was then transferred to the <u>reaction vial</u>. Then, 0.5 mL of MeCN was added to rinse the <u>reagent vial</u>. The mixture was again transferred to the reaction vial. The reaction was sparged in solution with a vent needle under N_2 for 5 minutes on ice (preventing the evaporation of the solvent). Then, the reaction mixture was subjected to IPR irradiation using 365 nm LED modules at 100% light intensity with maximum fan speed (6800 rpm) and 500 rpm stir rate for 8 to 24 hours. After this time, 1,4-dinitrobenzene was added as an internal standard, and an aliquot was removed for NMR analysis. Subsequently, the reaction mixture was basified by saturated $NaHCO_3$ solution and transferred to a separatory funnel. The reaction vial was rinsed into the separatory funnel with 1:1 EtOAc/water mixture (3 x 10 mL), and the layers separated. After extracting the aqueous layer three times with EtOAc (3 x 15 mL), the aqueous layer was added 1M NaOH solution and the pH was checked by pH indicators to be greater than 10. The aqueous solution was then extracted one last time with EtOAc (15 mL), the combined extracts were dried over Na_2SO_4, filtered, and concentrated *in vacuo.* The residue was subjected to automated column chromatography (prepHPLC or reverse phase chromatography) to afford the pure cyclized products.

General Procedure C-2 (preformed imine): piperidine/morpholine cyclization from activated amine/electron-rich aldehyde via HAT and radical sampling (0.5 mmol scale)

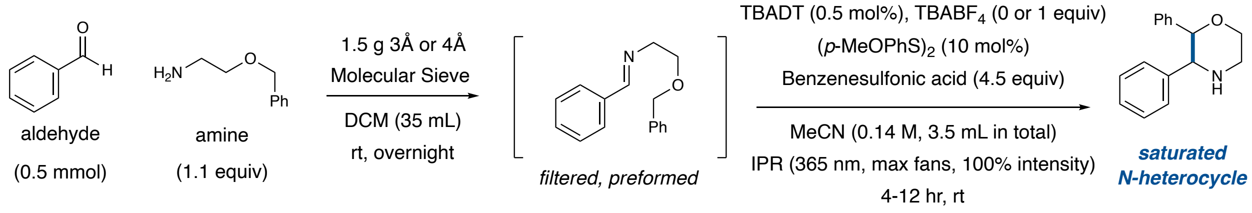

Preformed imine: To a 100-mL round-bottom flask (RBF), aldehyde (6 mmol, 1 equiv) was added and dissolved in ~35 mL of DCM. Then, 1.5 g molecular sieves (3Å or 4Å) were added into the flask. Lastly, amine (6.6 mmol, 1.1 equiv) was added into the RBF. After 12 hours, the mixture was filtered through celite and concentrated *in vacuo* to afford pure imine with quantitative yield.

Reagent vial: An 8-mL vial was charged with benzenesulfonic acid (355.9 mg, 2.25 mmol, 4.5 equiv) in the glovebox. Then, the reagent vial was capped tightly and transferred outside of the glovebox. TBADT (3.7 mg, 0.0025 mmol, 0.5 mol%), bis(4-methoxyphenyl) disulfide (13.9 mg, 0.05 mmol, 10 mol%) and tetrabutylammonium tetrafluoroborate $TBABF_4$ (164.6 mg, 0.5 mmol, 1 equiv) were weighed into the reagent vial under air.

Reaction vial: the reaction vial was added imine (0.5 mmol, 1 equiv).

Then, the reagent vial was then added 2 mL of MeCN to fully dissolve the reagents. The mixture was then transferred to the reaction vial. Then, 1.5 mL of MeCN was added to rinse the reagent vial. The mixture was again transferred to the reaction vial. The reaction was sparged in solution with a vent needle under N_2 for 5 minutes on ice (preventing the evaporation of the solvent). Then, the reaction mixture was subjected to IPR irradiation using 365 nm LED modules at 100% light intensity with maximum fan speed (6800 rpm) and 500 rpm stir rate for 8 to 24 hours. After this time, 1,4-dinitrobenzene was added as an internal standard, and an aliquot was removed for NMR analysis. Subsequently, the reaction mixture was basified by saturated $NaHCO_3$ solution and transferred to a separatory funnel. The reaction vial was rinsed into the separatory funnel with 1:1 EtOAc/water mixture (3 x 10 mL), and the layers separated. After extracting the aqueous layer three times with EtOAc (3 x 15 mL), the aqueous layer was added 1M NaOH solution and the pH was checked by pH indicators to be greater than 10. The aqueous solution was then extracted one last time with EtOAc (15 mL), the combined extracts were dried over Na_2SO_4, filtered, and concentrated *in vacuo.* The residue was subjected to automated column chromatography (prepHPLC or reverse phase chromatography) to afford the pure cyclized products.

7) Evidence for reversible HAT of radical sampling

Part I: Deuterium labeling study

[1] Synthesis of deuterated benzenesulfonic acid

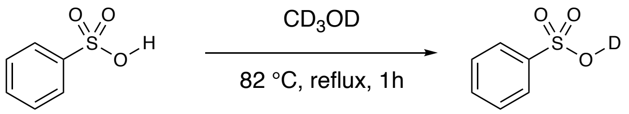

To an oven-dried 100-mL round-bottom flask (RBF), benzenesulfonic acid (2g, 126.4 mmol, 1 equiv) was added and dissolved in ~5 to 7 mL of anhydrous CD_3OD from glass ampoule. Then, the mixture was allowed to heat up to 82 °C and refluxed for 1 hour. The mixture was then immediately concentrated down and re-dissolved in another ~5 to 7 mL of anhydrous CD_3OD from glass ampoule, followed by refluxing at 82 °C for 1 hour. The process was repeated 3 to 5 times. Lastly, the mixture was immediately concentrated down and transferred to the glovebox for storage. To monitor the deuteration, CD_2Cl_2:DMSO-d_6 (v:v = 95:5) was used as the NMR solvent and the NMR relaxation time was set to 10s. Due to the slight changes of the ratio between CD_2Cl_2 and DMSO-d_6, the OH peak might be shifted. After evaluation of various conditions, the best result was 62% deuteration of the benzenesulfonic acid.

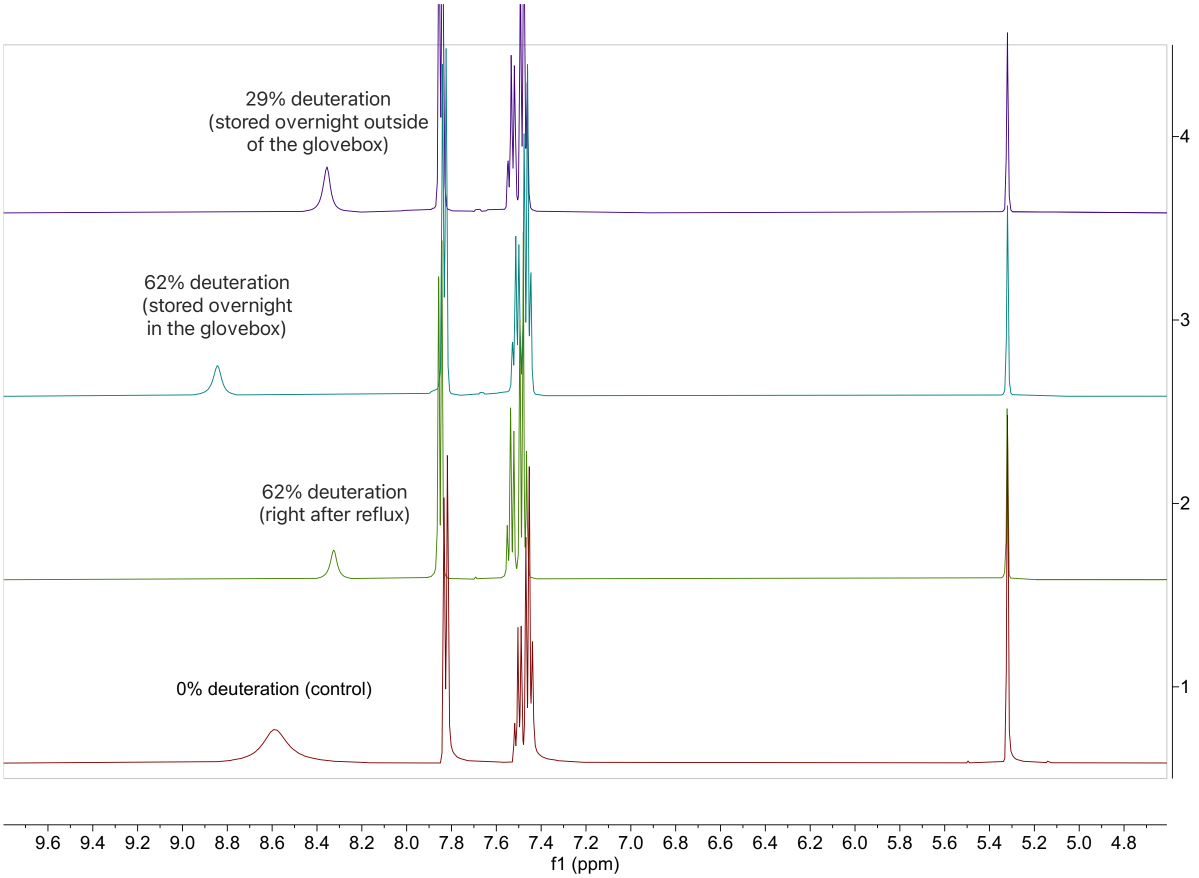

Figure S4. Deuteration monitor of the benzenesulfonic acid.

[2] Deuterium labeling study – with model substrate

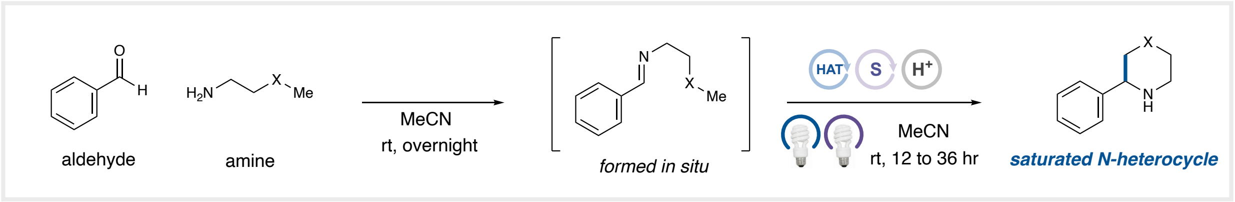

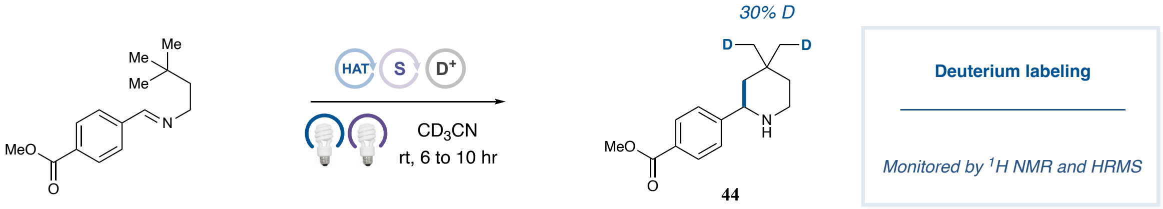

Reaction vial: *In the glovebox*, an oven-dried 40-mL vial was charged methyl (*E*)-4-(((3,3-dimethylbutyl)imino)methyl)benzoate (61.8 mg, 0.25 mmol), $FeCl_3$ (8.1 mg, 0.05 mmol, 20 mol%), bis(4-methoxyphenyl) disulfide (20.9 mg, 0.075 mmol, 30 mol%) and *d*-benzenesulfonic acid (159.2 mg, 1.0 mmol, 4 equiv, 62% deuteration). Then, 1.25 mL of anhydrous CD_3CN was added to dissolve the mixture. The reaction mixture was brought out from the glovebox and sealed with parafilm. The reaction was then irradiated under 365 nm light with 100% light intensity for 12 hours.

After working up according to **General Procedure A-2**, the basified, dry organic mixture was subjected to further purification by preparative reverse phase HPLC with 45% to 65% MeCN in water (0.1% NH_4OH modifier). Product containing fractions were concentrated to afford desired product as an off-white solid.

NMR results: The deuteration is mainly at two terminal methyl groups with 27% and 30% deuteration.

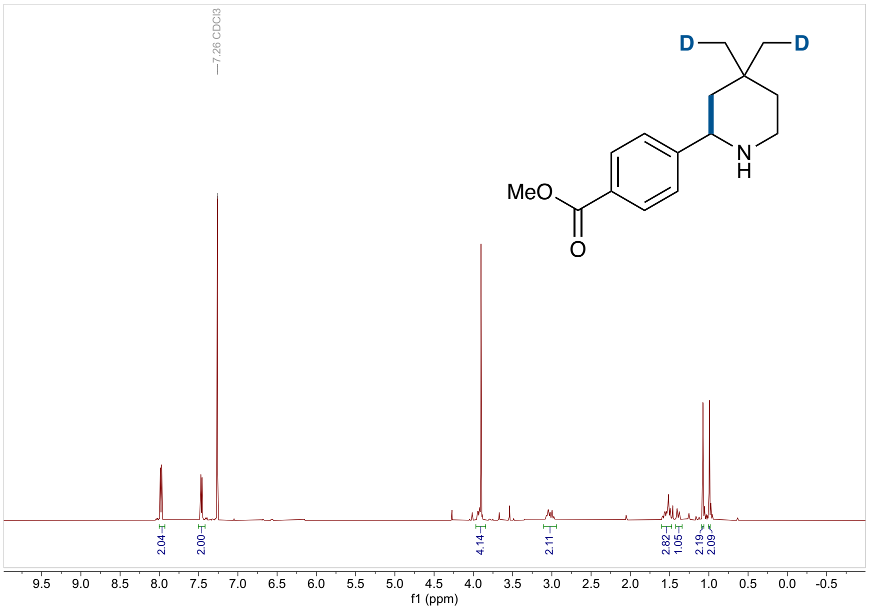

Figure S5. NMR analysis of deuterated piperidine cyclization product, compound **44**.

HRMS results: Though the NMR mainly demonstrates two positions with deuterium labeling, the HRMS found four different masses corresponding to no-deuteration, D_1, D_2, D_3 incorporation.

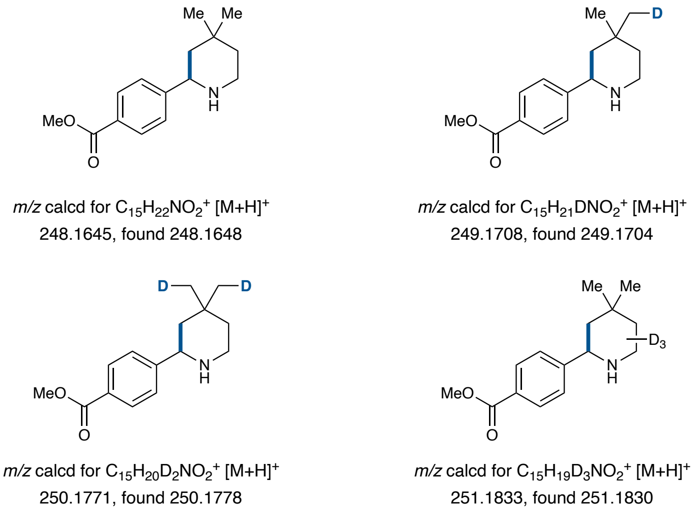

Figure S6. HRMS analysis of deuterated piperidine cyclization product.

[3] Deuterium labeling study – with 3-methylbutan-1-amine

Apart from the model substrate, a similar deuterium labeling study is carried out with 3-methylbutan-1-amine following the same protocol. As mentioned in Part I – [1], the deuterated benzenesulfonic acid was prepared by refluxing in CD_3OD instead of D_2O due to its hygroscopicity (with D_2O, imine hydrolyzes during the reaction). Though the utilization of CD_3OD avoids the presence of deuterated water, CD_3OD was difficult to be completely removed from deuterated benzenesulfonic acid. Therefore, during the deuterium labeling study, it produced a side product following the mechanism shown in **Figure S7** as the major product. It worths noticing that the radical generated from deuterated methanol is nucleophilic, rendering a highly polarity matched addition into iminium. Please refer to ***15) Structure elucidation of the side product from deuterium labeling studies*** for more details.

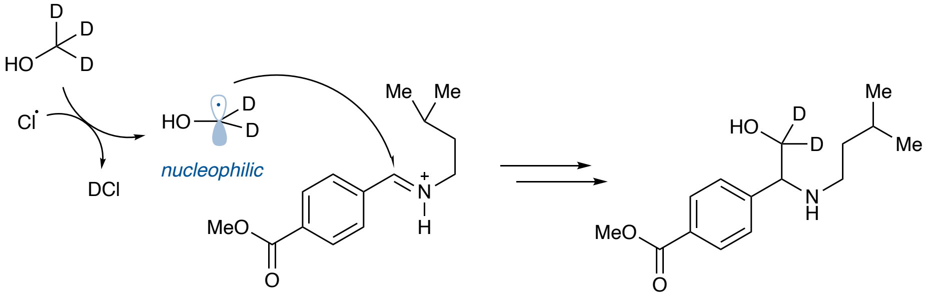

Figure S7. Mechanism for byproduct generation in deuterium labeling study.

For model substate (Part I – [2]), though the side product still formed, there was only one desired regioisomer (no diastereomer) as the deuterium labeled cyclized product to allow successful isolation and structure assignment. With 3-methylbutan-1-amine, two regioisomers are generated.

For the desired regioisomer, two possible diastereomers can form, making isolation and characterization challenging despite multiple purification protocols being attempted. Though unable to obtain the characterizable deuterated cyclized product, the deuterium incorporation to the cyclized product was supported by HRMS (**Figure S8**). In the meantime, the side product demonstrated successful deuterium incorporation (**Figure S8**) at various C–H bonds. Furthermore, the primary site of deuterium incorporation was at the tertiary position (24% deuterium incorporation), suggesting significant radical formation at this site. This finding provides further evidence that "incorrect," yet thermodynamically favored radical isomers, are funneled towards "correct," thermodynamically unfavorable radicals, that furnish the 6-membered products. Additionally, no incorporation was found at the α-amino C–H bonds which is also in agreement with our mechanistic design that the protonation of amine prevents α-amino hydrogen atom abstraction.

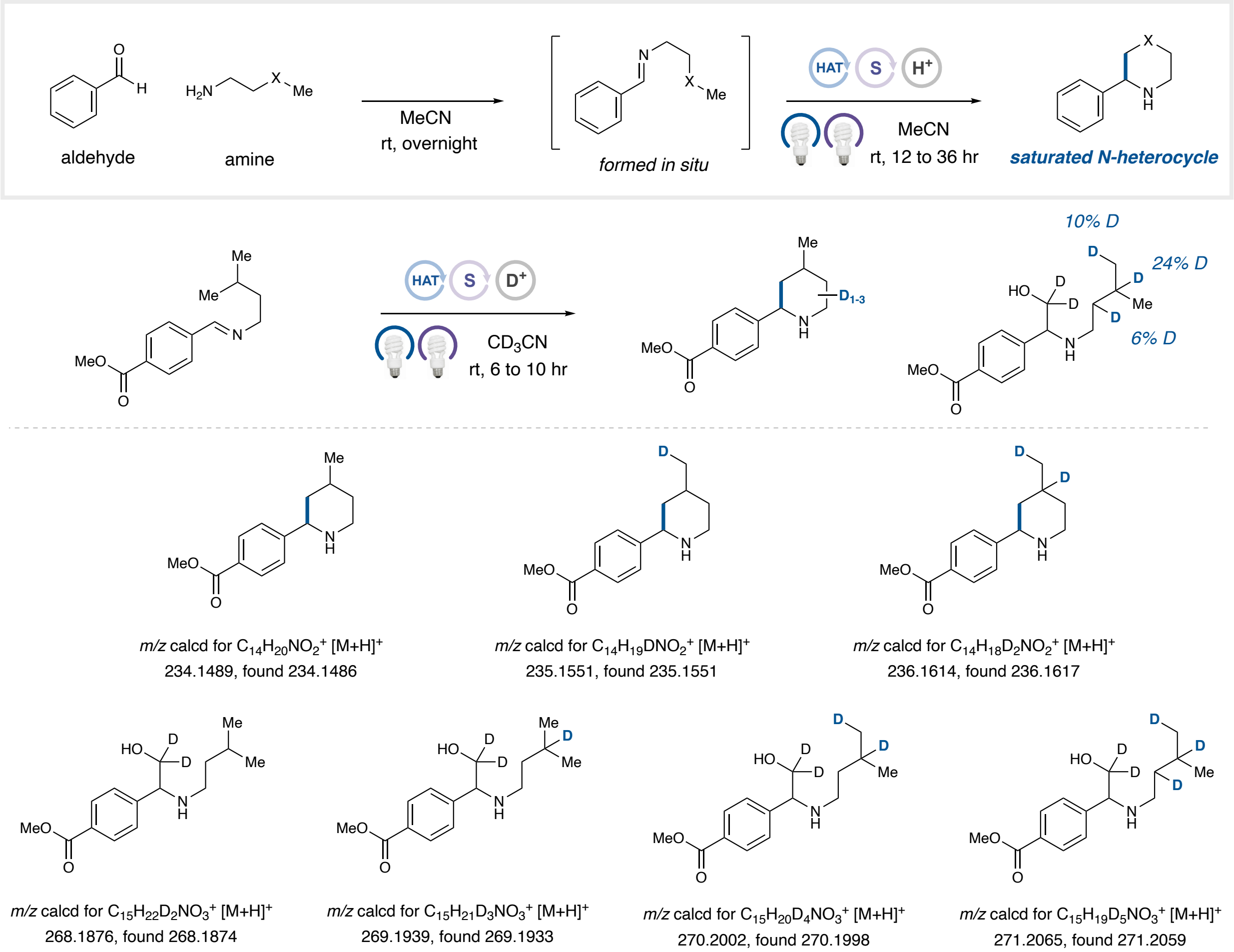

Figure S8. Deuterium labeling study with 3-methylbutan-1-amine.

Part II: Retention and loss of chirality study

[1] Loss of chirality: racemization of the chiral center

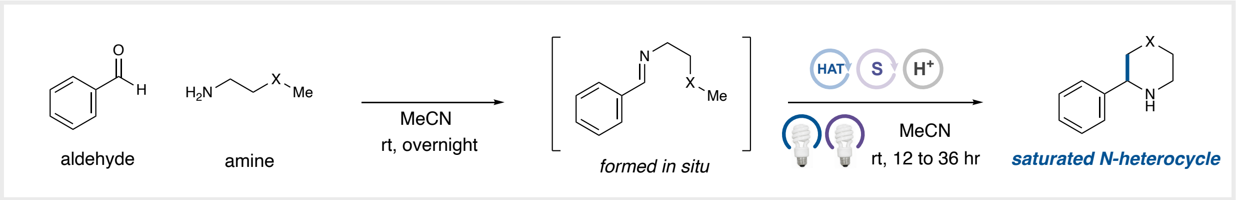

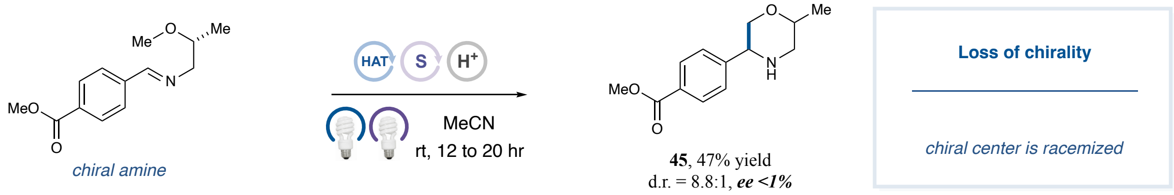

<u>Reaction setup:</u>

[1] Racemic (**compound S1**): racemic product were prepared according to **General Procedure B-2** with methyl (*E*)-4-(((2-methoxypropyl)imino)methyl)benzoate (117.6 mg, 0.5 mmol), sodium persulfate (119.1 mg, 0.5 mmol, 1 equiv), benzenesulfonic acid (355.9 mg, 2.25 mmol, 4.5 equiv), $FeCl_3$ (32.4 mg, 0.2 mmol, 40 mol%), bis(4-methoxyphenyl) disulfide (27.8 mg, 0.1 mmol, 20 mol%), and anhydrous MeCN (3.5 mL). The reaction was irradiated for 20 hrs under 100% light intensity, 420 nm plate.

After working up according to **General Procedure B-2**, the basified, dry organic mixture was subjected to further purification by preparative reverse phase HPLC with 35% to 50% MeCN in water (0.1% NH_4OH modifier). Product containing fractions were concentrated to afford desired product as an off white solid (43.7 mg, 0.19 mmol, 37% yield).

[2] Chiral (**compound 45**): chiral product were prepared according to **General Procedure B-2** with methyl (*R*,*E*)-4-(((2-methoxypropyl)imino)methyl)benzoate (76.2 mg, 0.3 mmol, 93% purity), sodium persulfate (71.4 mg, 0.3 mmol, 1 equiv), benzenesulfonic acid (213.5 mg, 1.35 mmol, 4.5 equiv), $FeCl_3$ (19.5 mg, 0.12 mmol, 40 mol%), bis(4-methoxyphenyl) disulfide (16.7 mg, 0.06 mmol, 20 mol%), and anhydrous MeCN (1.75 mL). The reaction was irradiated for 20 hrs under 100% light intensity, 420 nm plate.

After working up according to **General Procedure B-2**, the basified, dry organic mixture was subjected to further purification by preparative reverse phase HPLC with 35% to 50% MeCN in water (0.1% NH_4OH modifier). Product containing fractions were concentrated to afford desired product as an off white solid (33 mg, 0.14 mmol, 47% yield).

<u>ee analysis</u>: The results is analyzed by Chiral HPLC PrepI20-M7-30%_C2_2mL min_40C [ColumnTek Enantiocel® A3-5 (4.6 x 250 mm); 2.0 mL/min @ 30% isopropanol (0.1% diethylamine) / 70% CO2 (100 bar), 220 nm]

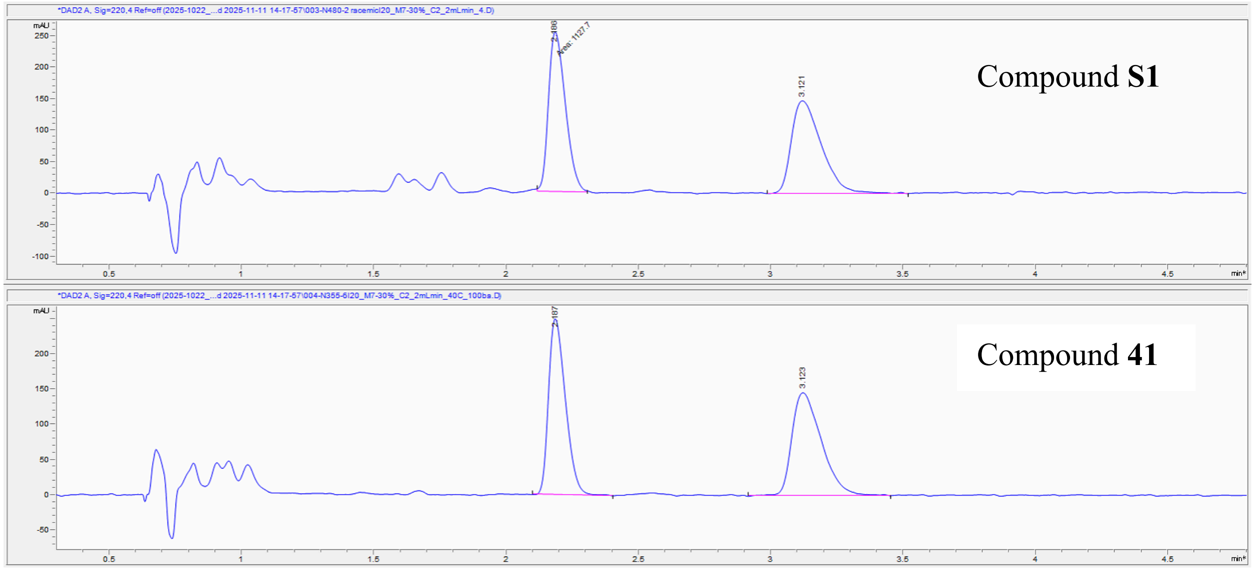

Figure S9. Chiral HPLC analysis of methyl 4-(6-methylmorpholin-3-yl)benzoate pair.

[2] Retention of chirality

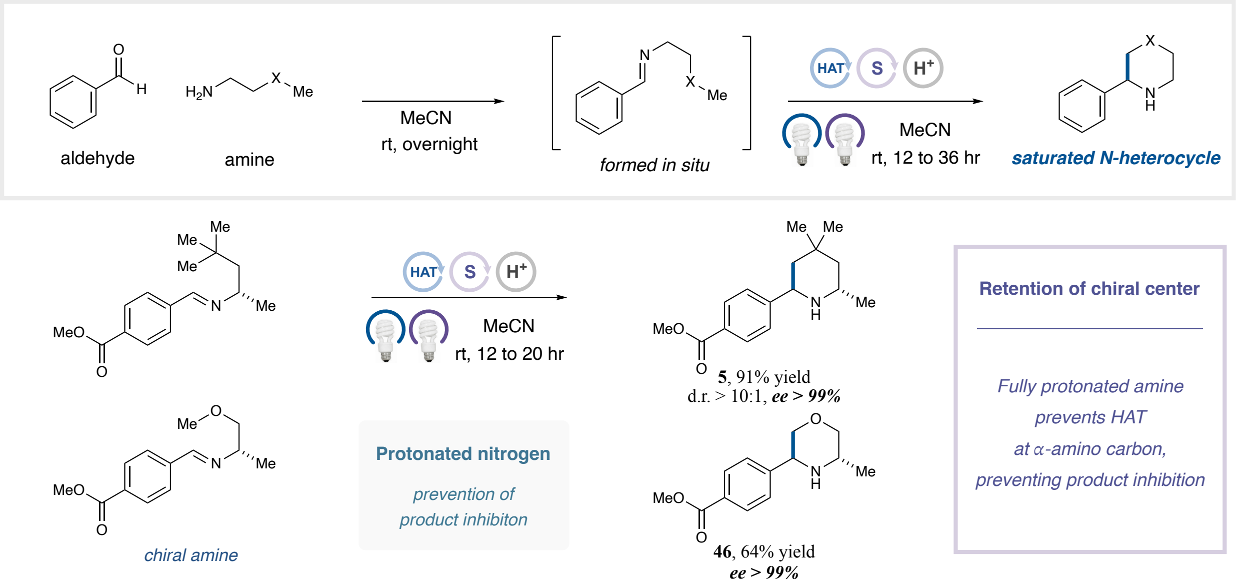

Piperidine - reaction setup:

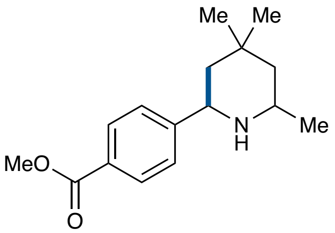

Compound S2: Prepared according to **General Procedure A-2** with methyl (*E*)-4-(((4,4-dimethylpentan-2-yl)imino)methyl)benzoate (78.4 mg, 0.3 mmol), benzenesulfonic acid (189.8 mg, 1.2 mmol, 4 equiv), $FeCl_3$ (9.7 mg, 0.06 mmol, 20 mol%), bis(4-methoxyphenyl) disulfide (25.1 mg, 0.09 mmol, 30 mol%), and anhydrous MeCN (1.5 mL). The reaction was irradiated for 12 hrs under 100% light intensity, 365 nm plate.

After working up according to **General Procedure A-2**, the basified, dry organic mixture was subjected to further purification by preparative reverse phase HPLC with 45% to 65% MeCN in water (0.1% NH_4OH modifier). Product containing fractions were concentrated to afford desired product as an off white solid (53.7 mg, 0.21 mmol, 68% yield).

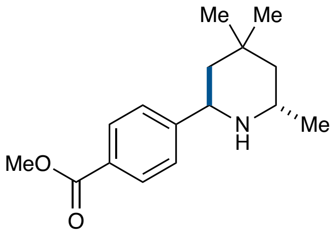

Preparation referred to piperidine cyclization scope, substrate **5**.

ee analysis: The results is analyzed by Chiral HPLC PrepI20-M7-15%_C2_2mL min_40C [ColumnTek Enantiocel® A3-5 (4.6 x 250 mm); 2.0 mL/min @ 15% isopropanol (0.1% diethylamine) / 85% CO2 (100 bar), 220 nm]

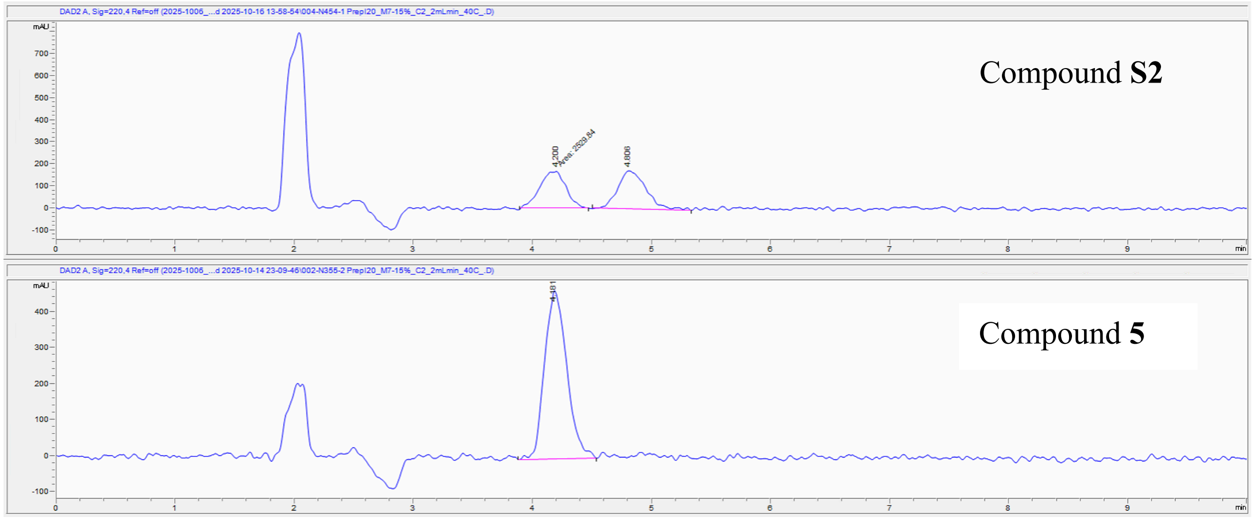

Figure S10. Chiral HPLC analysis of methyl 4-(4,4,6-trimethylpiperidin-2-yl)benzoate pair.

Morpholine - reaction setup:

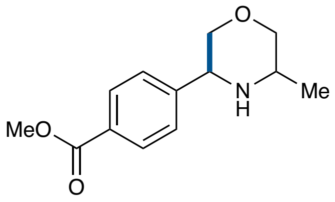

Preparation referred to morpholine/thiomorpholine cyclization scope, substrate **27**.

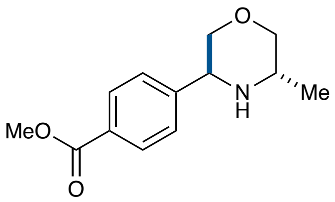

Compound 46: Prepared according to **General Procedure B-2** with methyl (*R*,*E*)-4-(((1-methoxypropan-2-yl)imino)methyl)benzoate (117.6 mg, 0.5 mmol), sodium persulfate (119.1 mg, 0.5 mmol, 1 equiv), benzenesulfonic acid (355.9 mg, 2.25 mmol, 4.5 equiv), $FeCl_3$ (32.4 mg, 0.2 mmol, 40 mol%), bis(4-methoxyphenyl) disulfide (27.8 mg, 0.1 mmol, 20 mol%), and anhydrous MeCN (3.5 mL). The reaction was irradiated for 20 hrs under 100% light intensity, 420 nm plate.

After working up according to **General Procedure B-2**, the mixture was purified by purification ***method (2) acid/base wash***. With the basified, dry organic mixture, Et_2O (10 mL) was added to partially dissolve the mixture, followed by addition of water (10 mL). Then, concentrated HCl was directly added into the mixture. After mixing, the mixture was transferred to separatory funnel. The layer separated and the aqueous layer was collected. Additional water and concentrated HCl were added into the Et_2O layer to allow the second mixing and layer separation. The process was repeated 3 to 4 times. The collected aqueous layer was basified by saturated $NaHCO_3$ solution and 1M NaOH solution. After the pH of the solution was checked by pH indicators to be greater than 10. The aqueous layer was extracted by EtOAc (4 x 20 mL) to afford light yellow/off-white products (75.8 mg, 0.32 mmol, 64% yield). With assistance of preparative reverse phase HPLC with 30% to 55% MeCN in water (0.1% NH_4OH modifier), the two diastereomer was further separated.

ee analysis: The results is analyzed by Chiral HPLC PrepI20-M7-20%_C3_2mL min_40C [ColumnTek Enantiocel® A8-5 (4.6 x 250 mm); 2.0 mL/min @ 20% isopropanol (0.1% diethylamine) / 80% CO2 (100 bar), 220 nm]

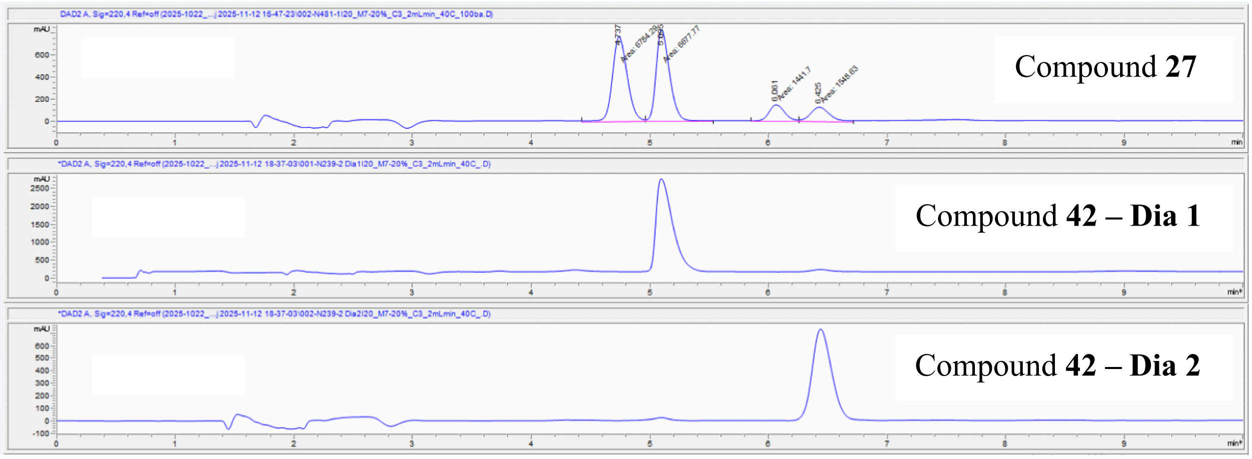

Figure S11. Chiral HPLC analysis of methyl 4-(5-methylmorpholin-3-yl)benzoate pair.

8) Computations (DFT)

Computational Methods: All computations were performed in Gaussian 09.[6] All molecular geometries were optimized at the M06-2x level of theory,[7] in conjunction with the def2-SVP atomic basis set. The density fitting W06 approach for the Coulomb integrals was used to accelerate the geometry optimization and frequencies calculations.[8] Frequency calculations were carried out at the same level of theory as for geometry optimization to confirm the stationary points as either minimum (no imaginary frequencies) or saddle points (one imaginary frequency) on the potential energy surface and to obtain thermal corrections to the Gibbs energies at 298 K and 1 atm pressure. Accurate electronic energies were obtained from single-point calculations at the M06-2x level on the optimized structures, with the Gaussian def2-TZVP atomic basis set. The density-fitting W06 approach for the Coulomb integrals was used to accelerate the calculations. The SMD solvation model[9] was used to compute the solvation Gibbs energies; acetonitrile was used as the solvent. The Gibbs energies are thus calculated for 1 atm standard state, while the reference state in solution is 1 M state for all compounds except the solvent. To account for this, correcting term − RT ln(1/(24.5 L/mol×1 mol/L)) = 1.9 kcal/mol was added to all species.

Benchmarking Functionals and Basis Sets:

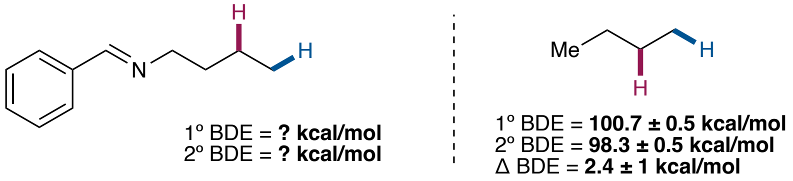

Functional	*Basis Set*	*Dispersion Correction*	*1° BDE (kcal/mol)*	*2° BDE (kcal/mol)*	*Δ BDE (kcal/mol)*
B3LYP	*def2-SZVP*	*D3BJ*	*99.4*	*95.5*	*3.9*
PBE	*def2-SZVP*	*D3BJ*	*98.0*	*93.5*	*4.5*
TPSS	*def2-SZVP*	*D3BJ*	*99.4*	*95.3*	*4.1*
M06-2x	***def2-SZVP***	***none***	***100.1***	***96.5***	***3.8***
WB97XD	*def2-SZVP*	*none*	*100.3*	*96.4*	*4.0*
M06-2x	*6-311G*	*none*	*100.5*	*97.0*	*3.5*
M06-2x	***def2-TZVP***	***none***	***100.2***	***97.0***	***3.2***
M06-2x	*def2-QZVP*	*none*	*100.6*	*97.3*	*3.3*
M06-2x	*cc-PVDZ*	*none*	*99.8*	*96.4*	*3.4*
M06-2x	*cc-PVTZ*	*none*	*100.9*	*97.6*	*3.3*
M06-2x	*cc-PVQZ*	*none*	*100.4*	*97.1*	*3.3*

Table S8: Benchmarking computational methods against known bond dissociation energies

Structures for benchmarking were optimized using B3LYP/def2-SVP with D3BJ dispersion correction and computed in the gas phase. BDE was calculated using the following equation with the enthalpies of the optimized species.

$BDE_{R\text{–}H} = \Delta H_{H\text{-}atom} + \Delta H_{free\text{-}radical} - \Delta H_{R\text{–}H}$

Literature values for C–H BDEs for butane can be found in "*The Comprehensive Handbook of Chemical Bond Energies*" by Yu-Ran Luo.[10] When evaluating a range of functionals, M06-2x performed best giving the closest ΔBDE to the literature. Next a few basis sets were evaluated using M06-2x. def2-TZVP was selected for all future energy calculations as this functional gave absolute BDEs and ΔBDE close to literature values while maintaining computational efficiency relative to the quadruple zeta functionals tested.

Computational Results:

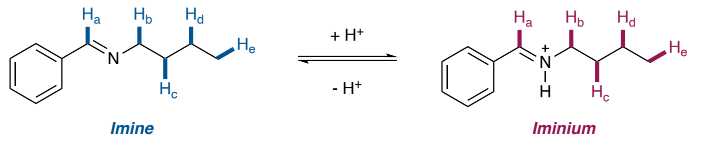

Position	BDE Imine (kcal/mol)	BDE Iminium (kcal/mol)	ΔBDE (kcal/mol)
H_a	97.2	110.3	+13.1
H_b	79.5	84.8	+5.3
H_c	98.2	97.6	-0.6
H_d	97.1	97.6	+0.5
H_e	100.2	101.0	+0.8

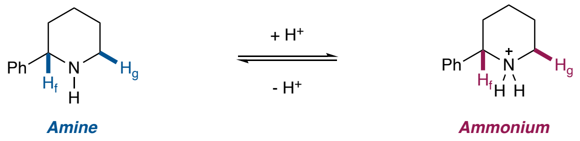

Position	BDE Amine (kcal/mol)	BDE Ammonium (kcal/mol)	ΔBDE (kcal/mol)
H_f	81.0	92.5	+11.5
H_g	90.7	101.7	+11.0

Table S9: Effect of protonation on imine and product BDEs

With a benchmarked computational system in hand, we modeled the bond dissociation energies for the imine and cyclized product (both neutral and iminium/ammonium species). Consistent with the literature, protonation of the nitrogen resulted in a ~5 to ~13 kcal/mol increase in α-nitrogen C–H bond strengths while having minimal effects distal to the site of protonation. The strengthening of the α-amino C–H bonds can be attributed to the ablation of the hyperconjugative interaction between the C–H σ* and nitrogen lone pair (furthermore, destabilizing the resultant carbon centered radical). Despite this strengthening, the α-amino C–H bonds (H_b = 84.8 kcal/mol and H_f = 92.5 kcal/mol) are still much weaker than the desired site of abstraction (H_e =101.0

kcal/mol). This effect can also be seen in the thermodynamics of chlorine radical abstraction computed in MeCN where α-amino abstraction is the thermodynamically favored HAT. Despite this, abstraction does not occur from these positions (see deuteration experiment) as they are kinetically disfavored due to polar effects in the HAT transition state.

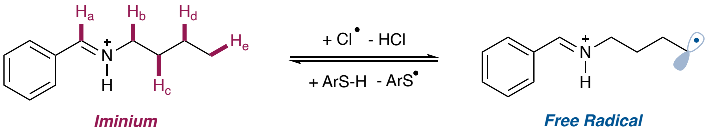

HAT site	ΔG HAT (kcal/mol)	ΔG back HAT (kcal/mol)	Comment
H_a	5.2	-33.8	uphill HAT
H_b	-20.7	-7.8	thermodynamically favored HAT position
H_c	-8.5	-20.0	–
H_d	-9.6	-19.0	–
H_e	-5.9	-21.5	–

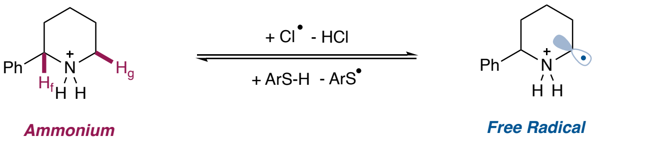

HAT site	ΔG HAT (kcal/mol)	ΔG back HAT (kcal/mol)	Comment
H_f	-16.6	-12.0	may cause pdt inhibition
H_g	-7.0	-21.5	–

Table S10: Thermodynamics of HAT by Cl radical and back HAT with thiol (*p*OMePhSH)

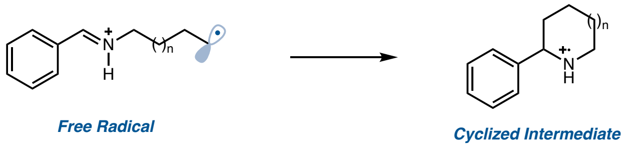

Ring size	ΔG HAT (kcal/mol)	ΔG‡ (kcal/mol)	k (sec^{-1})
4 membered	12.9	26.8	1.4×10^{-7}
5 membered	-9.3	10.7	8.6×10^{4}
6 membered	-10.7	2.8	5.6×10^{10}
7 membered	-8.1	5.2	9.6×10^{8}
8 membered	-1.4	10.8	7.4×10^{4}

Table S11: Effects of ring size on rates and energetics of cyclization

Computationally, it is thermodynamically downhill to make rings of 5 to 8 members. However, the relative rates of cyclization differ significantly between our observed favored product (6 membered ring, $k = 5.6 \times 10^{10}$) and other observed minor products (5 and 7 membered rings, k =

8.6 x 10^4 and 9.6 x 10^8). In conjunction with experimental rates for cyclization these computational results provide preliminary evidence to support our observed regioselectivity (given the ~100x faster cyclization rate versus other ring sizes). Furthermore, HAT between carbon centered radicals and aryl thiols is approximately k = 10^7 to 10^8 sec^{-1} thus,[11] the back HAT pathway is competitive or favored when compared with any non 6 membered ring transition state.

Optimized Geometries and Energies:

H Atom

M06-2x/def2svp, gas phase, correction to enthalpy = 0.00236 Hartree

M06-2x/def2tzvp, gas phase, E = -0.498139 Hartree

H	0.00000000	0.00000000	0.00000000

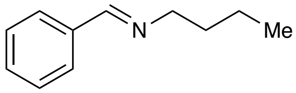

M06-2x/def2svp, gas phase, correction to enthalpy = 0.250027 Hartree

M06-2x/def2tzvp, gas phase, E = -482.896256 Hartree

C	-4.33829800	0.50967000	0.31850900
C	-3.98016500	-0.82307300	0.51255900
C	-2.65960800	-1.22282200	0.31143700
C	-1.69165200	-0.29456900	-0.08388900
C	-2.05784700	1.04432500	-0.27745500
C	-3.37448400	1.44212700	-0.07678200
H	-5.37100600	0.82548600	0.47495000
H	-4.73034200	-1.55270100	0.82086600
H	-2.37327400	-2.26660900	0.46201700
H	-1.28530000	1.75002600	-0.58587600
H	-3.65679700	2.48519100	-0.22828500
C	-0.29595100	-0.73381300	-0.29294200
H	-0.10118000	-1.81161400	-0.11634000
N	0.62513700	0.05592500	-0.65121100
C	1.96024900	-0.46659800	-0.81412800
H	2.27123500	-0.28496600	-1.85698900
H	2.00398700	-1.56178200	-0.64279400
C	2.93984100	0.24391500	0.11585200
H	2.85470400	1.32972300	-0.05116100
H	2.63199900	0.06540500	1.15989500
C	4.38209200	-0.20920700	-0.08431300
H	4.44635400	-1.30008200	0.06508300
C	5.35595200	0.49551800	0.85300500
H	5.09840800	0.30225400	1.90510800
H	6.38923000	0.15823000	0.69211600
H	5.32902500	1.58476600	0.70040700
H	4.67623000	-0.02763500	-1.13163200

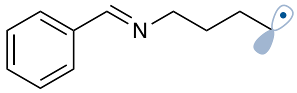

M06-2x/def2svp, gas phase, correction to enthalpy = 0.235197 Hartree
M06-2x/def2tzvp, gas phase, E = -482.225898 Hartree

C	-4.28063800	0.49146300	0.30718900
C	-3.91539700	-0.84069500	0.49141500
C	-2.59106600	-1.23025300	0.29549900
C	-1.62650100	-0.29227000	-0.08478800
C	-1.99984200	1.04600800	-0.26853800
C	-3.32023600	1.43360500	-0.07304300
H	-5.31633600	0.79927900	0.45962200
H	-4.66295400	-1.57778700	0.78802500
H	-2.29912200	-2.27355500	0.43840600
H	-1.22984200	1.75945700	-0.56527600
H	-3.60820000	2.47619100	-0.21680200
C	-0.22680800	-0.72065700	-0.28838400
H	-0.02622000	-1.79875000	-0.12053000
N	0.69154500	0.07825400	-0.63337100
C	2.03063300	-0.43417800	-0.79286500
H	2.34559600	-0.24454100	-1.83317000
H	2.08155800	-1.53003800	-0.62725800
C	2.99942000	0.27836300	0.14615900
H	2.91448200	1.36404700	-0.01451300
H	2.68960600	0.09052800	1.18763000
C	4.44660400	-0.16972500	-0.04884600
H	4.49769200	-1.27197100	0.07601700
C	5.39761500	0.49606700	0.88294500
H	5.06905100	0.79157500	1.88103400
H	6.46726000	0.51004400	0.67358900
H	4.75390700	0.01137500	-1.09363900

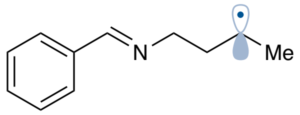

M06-2x/def2svp, gas phase, correction to enthalpy = 0.235363 Hartree
M06-2x/def2tzvp, gas phase, E = -482.23107 Hartree

C	-4.29994100	0.52090100	0.29250900
C	-3.95247900	-0.81624500	0.47506000
C	-2.63161700	-1.22142800	0.28775500
C	-1.65272200	-0.29418500	-0.08209300
C	-2.00824900	1.04914500	-0.26437200
C	-3.32521100	1.45234000	-0.07750400
H	-5.33290000	0.84097300	0.43814000
H	-4.71123700	-1.54503300	0.76363600
H	-2.35359800	-2.26870100	0.42934600
H	-1.22735900	1.75390500	-0.55330600
H	-3.59929900	2.49880600	-0.22022000
C	-0.25691100	-0.73936700	-0.27621100
H	-0.07168500	-1.82087700	-0.11283400
N	0.67473300	0.04925700	-0.60896100

C	2.00752500	-0.47991500	-0.76258800
H	2.33363200	-0.29786300	-1.79995000
H	2.04635300	-1.57488000	-0.58992400
C	2.98386400	0.22479500	0.17822500
H	2.90940600	1.31411500	0.01646000
H	2.63925400	0.06276700	1.22098800
C	4.39160500	-0.23387100	0.00768400
H	4.56245600	-1.25892000	-0.33406900
C	5.51584600	0.50690300	0.64304300
H	5.54538200	0.35090100	1.73939600
H	6.49127800	0.19225400	0.24772700
H	5.41492700	1.59330800	0.48828100

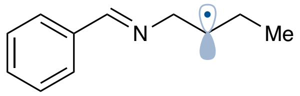

M06-2x/def2svp, gas phase, correction to enthalpy = 0.235342 Hartree
M06-2x/def2tzvp, gas phase, E = -482.229358 Hartree

C	-4.27574800	0.50632700	0.35870500
C	-3.90357500	-0.81587200	0.59366100
C	-2.58601100	-1.21517400	0.37323200
C	-1.63520800	-0.29697000	-0.08225400
C	-2.01549500	1.03127400	-0.31704100
C	-3.32910100	1.42866500	-0.09695200
H	-5.30614900	0.82178800	0.53021000
H	-4.64038500	-1.53755800	0.94916600
H	-2.28862700	-2.25065300	0.55612200
H	-1.25635400	1.72942100	-0.67231100
H	-3.62245100	2.46348800	-0.28043800
C	-0.24240800	-0.73594400	-0.31024800
H	-0.03629300	-1.80491600	-0.09856300
N	0.66339500	0.04594800	-0.71947000
C	1.99612200	-0.47998900	-0.90588700
H	2.24346300	-0.37957800	-1.98417500
H	2.05894400	-1.56692000	-0.67946300
C	2.99863300	0.27052700	-0.09457300
H	2.75424400	1.30455100	0.16330400
C	4.41305100	-0.19329000	-0.01942000
H	4.43418500	-1.28499300	0.14893700
C	5.21919200	0.51893300	1.06323400
H	4.77212200	0.35235700	2.05365300
H	6.25846200	0.16434300	1.09162300
H	5.23656400	1.60394600	0.88380000
H	4.91180900	-0.04784100	-1.00032500

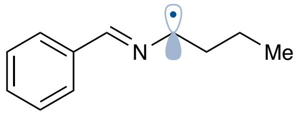

M06-2x/def2svp, gas phase, correction to enthalpy = 0.235775 Hartree
M06-2x/def2tzvp, gas phase, E = -482.259548 Hartree

C	-4.25715600	0.72618300	0.16109200
C	-4.07009000	-0.65382300	0.27018400
C	-2.79381600	-1.19453400	0.17578100
C	-1.67318100	-0.36310600	-0.03049600
C	-1.87601100	1.03031700	-0.13902700
C	-3.15397300	1.56205500	-0.04365500
H	-5.25953600	1.15017800	0.23516100
H	-4.92722700	-1.30979900	0.43000300
H	-2.64774500	-2.27380500	0.26107000
H	-1.00502700	1.66622400	-0.29864000
H	-3.29875600	2.64032100	-0.12915000
C	-0.35720300	-0.93580100	-0.12609900
H	-0.26066300	-2.02998900	-0.03485900
N	0.73175100	-0.17942000	-0.31954900
C	1.90148400	-0.73646700	-0.40054500
H	2.00594900	-1.83447000	-0.30351200
C	3.14912500	0.05752900	-0.59459000
H	3.65358600	-0.27439300	-1.52032600
H	2.86611900	1.11149400	-0.73111700
C	4.13032700	-0.08827000	0.57552800
H	3.63381100	0.25003900	1.49876900
C	5.41749100	0.69708600	0.35548500
H	5.20602900	1.76918200	0.22919400
H	6.10626900	0.58710200	1.20430500
H	5.93946600	0.35258800	-0.54983700
H	4.36348500	-1.15574700	0.72383000

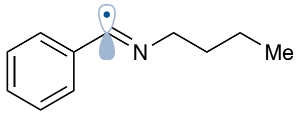

M06-2x/def2svp, gas phase, correction to enthalpy = 0.237592 Hartree
M06-2x/def2tzvp, gas phase, E = -482.23311 Hartree

C	-4.35960800	0.49854500	0.31317100
C	-3.99503500	-0.82831100	0.53740600
C	-2.67490900	-1.22915400	0.33997000
C	-1.71491400	-0.30414900	-0.08265500
C	-2.08690800	1.03083400	-0.30723200
C	-3.40466500	1.42744500	-0.10914600
H	-5.39304800	0.81307300	0.46757200
H	-4.74139700	-1.55245400	0.86685000
H	-2.37023900	-2.26313300	0.51029400
H	-1.32214300	1.73604100	-0.63650200
H	-3.69324400	2.46519500	-0.28382200
C	-0.32425300	-0.73751400	-0.28769500
N	0.64985800	-0.08049400	-0.65867300
C	2.01347200	-0.53621600	-0.83070000

H	2.31012600	-0.32679200	-1.87103400
H	2.08301600	-1.62620000	-0.67279400
C	2.95545000	0.19955200	0.11604300
H	2.83187800	1.28436000	-0.03457400
H	2.64797500	-0.00867800	1.15420500
C	4.41454300	-0.19593600	-0.08414800
H	4.51849100	-1.28540900	0.05114400
C	5.35666400	0.53223900	0.86748800
H	5.10167100	0.31511600	1.91546200
H	6.40236900	0.23570700	0.70717400
H	5.29004600	1.62167200	0.72910100
H	4.70647400	0.01095400	-1.12736600

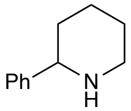

M06-2x/def2svp, gas phase, correction to enthalpy = 0.252691 Hartree
M06-2x/def2tzvp, gas phase, E = -482.915116 Hartree

C	2.68255200	0.92600000	-0.81661900
C	3.36936300	0.24656200	0.36241500
C	2.74123100	-1.12230800	0.61897100
C	1.22846000	-0.99197200	0.78708200
C	0.60080900	-0.25855300	-0.40732100
H	4.44820800	0.15336100	0.16799800
H	2.90245600	0.34315800	-1.73883000
H	3.08930200	1.93664500	-0.96640500
H	0.79543500	1.56389900	-1.29778200
H	2.95384300	-1.78345300	-0.23933000
H	3.18729600	-1.59860800	1.50464200
H	0.75365500	-1.97856400	0.89652200
H	0.99725300	-0.41146700	1.69529800
H	0.75604400	-0.90252100	-1.30320500
H	3.24335000	0.88245900	1.25328000
C	-0.89058900	-0.08840700	-0.21517000
C	-1.77938200	-1.05968500	-0.68598000
C	-1.39980500	1.01281300	0.48185500
C	-3.15017700	-0.93825700	-0.46430600
H	-1.39023600	-1.91967600	-1.23720700
C	-2.77094200	1.13769400	0.70107600
H	-0.70226600	1.77034500	0.84286100
C	-3.65006500	0.16279000	0.23032900
H	-3.83131200	-1.70333600	-0.84055500
H	-3.15614000	2.00258800	1.24401800
H	-4.72307200	0.26214300	0.40145500
N	1.25535100	1.03185400	-0.56239200

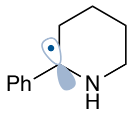

M06-2x/def2svp, gas phase, correction to enthalpy = 0.239648 Hartree
M06-2x/def2tzvp, gas phase, E = -482.277189 Hartree

C	-2.64597200	-1.30639400	-0.06695100
C	-3.46221900	-0.04393000	0.16910600
C	-2.76592800	1.14118400	-0.49118500
C	-1.35911000	1.32532300	0.08433700
C	-0.56958700	0.04564700	0.09859900
H	-4.47809700	-0.18225400	-0.22836300
H	-2.69454200	-1.56884900	-1.14302800
H	-3.06523000	-2.15227700	0.49567300
H	-0.72582400	-1.94488500	0.50059700
H	-2.69418800	0.95672200	-1.57625300
H	-3.34908400	2.06345900	-0.35760200
H	-0.81814300	2.08706700	-0.49188600
H	-1.44770500	1.71476600	1.11844100
H	-3.54904200	0.13340600	1.25365000
C	0.85724000	0.01652100	0.03249900
C	1.62951500	1.20680900	0.14378200
C	1.58197000	-1.19736300	-0.13593200
C	3.01438100	1.17647800	0.09581500
H	1.12832200	2.16280300	0.29656900
C	2.96792900	-1.21319000	-0.18029700
H	1.05011900	-2.13979600	-0.28013000
C	3.70394600	-0.03051400	-0.06323600
H	3.57060900	2.11084800	0.19285700
H	3.48560200	-2.16404400	-0.32135800
H	4.79340100	-0.04818600	-0.09856800
N	-1.27417000	-1.10745900	0.36002500

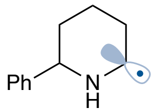

M06-2x/def2svp, gas phase, correction to enthalpy = 0.239121 Hartree
M06-2x/def2tzvp, gas phase, E = -482.261208 Hartree

C	-2.67130400	-1.01628700	-0.74190800
C	-3.40744700	-0.26973500	0.32398000
C	-2.78934800	1.11241300	0.54609600
C	-1.28305600	0.97782100	0.75584700
C	-0.62240200	0.29551400	-0.44870600
H	-4.47083400	-0.18770400	0.05596200
H	-3.09483000	-1.93741700	-1.14676600
H	-0.82450600	-1.51670100	-1.42839500
H	-2.97564000	1.74064300	-0.34086600
H	-3.25454100	1.61374600	1.40739100
H	-0.80729600	1.95742200	0.91077400

H	-1.07850200	0.36727100	1.65168900
H	-0.75572900	0.97254400	-1.31853200
H	-3.36840200	-0.82432800	1.28573300
C	0.86239600	0.10261500	-0.22913000
C	1.76776600	1.07938700	-0.65477000
C	1.34879600	-1.02100200	0.44753400
C	3.13249300	0.94091000	-0.40795300
H	1.39633500	1.95681400	-1.19035100
C	2.71404100	-1.16343300	0.69157200
H	0.64134900	-1.78467800	0.77583100
C	3.60959200	-0.18285200	0.26608000
H	3.82708100	1.71035300	-0.74885100
H	3.08103700	-2.04591000	1.21841600
H	4.67799800	-0.29535700	0.45644700
N	-1.28038200	-0.97982800	-0.69719100

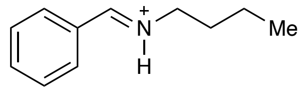

M06-2x/def2svp, gas phase, correction to enthalpy = 0.26434 Hartree
M06-2x/def2tzvp, gas phase, E = -483.275476 Hartree

C	-4.37192600	0.44840200	0.35114100
C	-3.98431900	-0.88268600	0.50430100
C	-2.66001500	-1.24123000	0.28333500
C	-1.71831700	-0.26478700	-0.09220600
C	-2.12078800	1.07828500	-0.24454900
C	-3.44207100	1.42817200	-0.02273200
H	-5.41122200	0.73216700	0.52372600
H	-4.71411100	-1.63817400	0.79499600
H	-2.34741300	-2.28100800	0.40037100
H	-1.41300800	1.85760100	-0.53632000
H	-3.75853200	2.46466100	-0.13936700
C	-0.36369600	-0.70881600	-0.30553200
H	-0.14427100	-1.77386000	-0.16719600
N	0.63886400	0.02792200	-0.65224800
C	2.02105100	-0.43071300	-0.84126300
H	2.30509800	-0.20268800	-1.87951600
H	2.02135000	-1.52308400	-0.72382300
C	2.97606800	0.23645100	0.14106800
H	2.91594800	1.33224200	0.02683000
H	2.65975300	0.00338700	1.17052900
C	4.41749700	-0.22013100	-0.08045500
H	4.46964200	-1.31615300	0.02229000
C	5.38575200	0.43809300	0.89500500
H	5.12472900	0.19764000	1.93562300
H	6.41309600	0.09507000	0.71912700
H	5.37412400	1.53224900	0.78713400
H	4.71491900	0.00899800	-1.11653600
H	0.48244600	1.02926800	-0.78081800

M06-2x/def2svp, SMD = MeCN, correction to Gibbs free energy = 0.211719 Hartree
M06-2x/def2tzvp, SMD = MeCN, E = -483.370434 Hartree

C	4.36673100	-0.41513100	0.37853900
C	3.95895800	0.91325400	0.49921400
C	2.63083900	1.25111900	0.25721800
C	1.70748900	0.25685800	-0.10514500
C	2.12506800	-1.08095300	-0.22683500
C	3.45144800	-1.41010600	0.01580400
H	5.40791100	-0.68239600	0.56746600
H	4.67544300	1.68531100	0.78145600
H	2.29644000	2.28648900	0.34714300
H	1.42304300	-1.86743700	-0.50981300
H	3.77869600	-2.44619600	-0.07735200
C	0.34047700	0.68559200	-0.34105600
H	0.11134200	1.75252300	-0.24114600
N	-0.63917200	-0.07962200	-0.66457400
C	-2.01570900	0.36080900	-0.88716300
H	-2.30252900	0.02986800	-1.89556600
H	-2.01913100	1.45803800	-0.86311100
C	-2.95672300	-0.22223900	0.15801700
H	-2.87715900	-1.32182700	0.14638700
H	-2.63656400	0.11678100	1.15641800
C	-4.40155500	0.19479400	-0.09856600
H	-4.46252100	1.29536500	-0.11048200
C	-5.36023000	-0.36466800	0.94431200
H	-5.09370100	-0.01445600	1.95315700
H	-6.39527500	-0.05425400	0.74246100
H	-5.33403700	-1.46500900	0.95420500
H	-4.70170900	-0.14417800	-1.10355900
H	-0.47679700	-1.08724500	-0.76167300

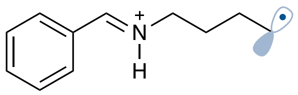

M06-2x/def2svp, gas phase, correction to enthalpy = 0.249531 Hartree
M06-2x/def2tzvp, gas phase, E = -482.603897 Hartree

C	4.31247900	0.43352400	-0.34163700
C	3.91892800	-0.89651800	-0.48870000
C	2.59179500	-1.24706900	-0.27211000
C	1.65314300	-0.26355000	0.09283600
C	2.06173700	1.07846300	0.23913500
C	3.38580200	1.42028300	0.02179900
H	5.35402800	0.71101500	-0.51083400
H	4.64629600	-1.65737600	-0.77133600
H	2.27451100	-2.28593500	-0.38455100
H	1.35658700	1.86311600	0.52286700

H	3.70701800	2.45581600	0.13382200
C	0.29549200	-0.69934700	0.30151500
H	0.07137800	-1.76417100	0.16917600
N	-0.70552600	0.04505500	0.63750400
C	-2.09041300	-0.40558400	0.82026500
H	-2.38529000	-0.15831500	1.85113800
H	-2.09336700	-1.49987900	0.72215000
C	-3.03123200	0.24714700	-0.18519600
H	-2.97042400	1.34438600	-0.08932200
H	-2.71042900	-0.00581700	-1.20760300
C	-4.48002500	-0.19963800	0.03050200
H	-4.54583600	-1.29702700	-0.05006400
C	-5.41808300	0.44045200	-0.93231000
H	-6.35652300	-0.04563000	-1.19475000
H	-5.29101600	1.48986500	-1.20343400
H	-4.77141700	0.03910900	1.07404300
H	-0.54457000	1.04647200	0.75958000

M06-2x/def2svp, SMD = MeCN, correction to Gibbs free energy = 0.195596 Hartree
M06-2x/def2tzvp, SMD = MeCN, E = -482.699652 Hartree

C	4.30953800	0.41373100	-0.35314500
C	3.90170700	-0.91347300	-0.48577600
C	2.57063900	-1.25120600	-0.26039700
C	1.64435500	-0.25808400	0.09782600
C	2.06200600	1.07874000	0.23081100
C	3.39126200	1.40775300	0.00468300
H	5.35302800	0.68092300	-0.52899600
H	4.62043300	-1.68473100	-0.76447600
H	2.23626700	-2.28572200	-0.35985500
H	1.35761800	1.86466500	0.50933900
H	3.71847300	2.44301200	0.10678600
C	0.27478300	-0.68706700	0.31713500
H	0.04436500	-1.75216800	0.20183200
N	-0.70616400	0.07578700	0.64315100
C	-2.08487900	-0.36493300	0.84735300
H	-2.38206800	-0.04555900	1.85650700
H	-2.09074500	-1.46173000	0.80984600
C	-3.01291100	0.23358000	-0.20031800
H	-2.93396400	1.33258500	-0.17321100
H	-2.68806500	-0.09375000	-1.20027900
C	-4.46342000	-0.18294000	0.04026700
H	-4.53941500	-1.28339500	0.04298100
C	-5.40185500	0.39237300	-0.96015100
H	-6.38254800	-0.05775000	-1.12196900
H	-5.22141900	1.38914000	-1.36977000
H	-4.75337900	0.13139100	1.06422700
H	-0.54278600	1.08173000	0.75524800

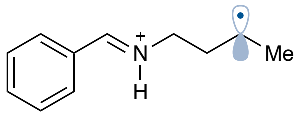

M06-2x/def2svp, gas phase, correction to enthalpy = 0.249684 Hartree
M06-2x/def2tzvp, gas phase, E = -482.609535 Hartree

C	-4.33492900	0.32065300	0.40943300
C	-3.90667900	-1.00656600	0.38450900
C	-2.57346500	-1.29204800	0.11612900
C	-1.66348000	-0.24609500	-0.12763000
C	-2.10719300	1.09250300	-0.10107400
C	-3.43716300	1.36929800	0.16675600
H	-5.38134400	0.54729500	0.61948600
H	-4.61178700	-1.81581100	0.57364200
H	-2.22903400	-2.32809800	0.09403800
H	-1.42503600	1.92470800	-0.28961100
H	-3.78558300	2.40186700	0.18797200
C	-0.29733800	-0.61709300	-0.39744400
H	-0.04665600	-1.68421100	-0.40624400
N	0.68342700	0.18881000	-0.63721500
C	2.07548700	-0.20315700	-0.88848800
H	2.34888700	0.16747300	-1.88765000
H	2.11231600	-1.30036600	-0.91227700
C	3.02331600	0.35767300	0.16985800
H	2.89548800	1.45986200	0.21710300
H	2.73587100	-0.02674600	1.16238400
C	4.43970000	-0.00431300	-0.13560700
C	5.46578100	-0.05915700	0.93869200
H	5.08435100	-0.56738400	1.83776200
H	6.37020200	-0.58214000	0.60248300
H	5.78035300	0.95346700	1.25531500
H	4.76501600	0.06465700	-1.17757400
H	0.49873200	1.19356800	-0.62712600

M06-2x/def2svp, SMD = MeCN, correction to Gibbs free energy = 0.194901 Hartree
M06-2x/def2tzvp, SMD = MeCN, E = -482.704775 Hartree

C	-4.32802300	0.34953300	0.40340400
C	-3.90494800	-0.97954600	0.41715800
C	-2.57206400	-1.28124800	0.15488100
C	-1.65930200	-0.24982400	-0.12045800
C	-2.09245000	1.08865000	-0.13532100
C	-3.42349200	1.38146000	0.12722400
H	-5.37305600	0.58829100	0.60853600
H	-4.61343800	-1.78028300	0.63147200
H	-2.22585100	-2.31659400	0.16103600
H	-1.39902800	1.90365000	-0.35126500
H	-3.76291600	2.41769800	0.11689200
C	-0.28628100	-0.64088100	-0.38395500

H	-0.04287300	-1.70894400	-0.36758700
N	0.68360700	0.16073100	-0.64249200
C	2.06440000	-0.24232400	-0.90150800
H	2.34100000	0.15999100	-1.88652600
H	2.08951000	-1.33808300	-0.94826700
C	3.00482100	0.29081700	0.17395900
H	2.86741500	1.38927400	0.25122400
H	2.71226700	-0.12200000	1.15416900
C	4.42678100	-0.04271200	-0.12702600
C	5.46254200	0.04538100	0.93670500
H	5.12652600	-0.44349200	1.86581700
H	6.40952500	-0.41598100	0.62364700
H	5.68698600	1.09747900	1.20236400
H	4.73893200	-0.04273100	-1.17570500
H	0.50783600	1.17077900	-0.65874000

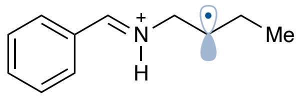

M06-2x/def2svp, gas phase, correction to enthalpy = 0.249834 Hartree
M06-2x/def2tzvp, gas phase, E = -482.609619 Hartree

C	-3.92145800	-0.73647800	0.09782200
C	-3.52610000	-0.14245300	-1.10072400
C	-2.25189000	0.40123200	-1.20934700
C	-1.36835600	0.35167900	-0.11480100
C	-1.77810600	-0.25076000	1.09261700
C	-3.04914700	-0.79113500	1.19330900
H	-4.92146800	-1.16394300	0.18539000
H	-4.21097500	-0.10403800	-1.94767600
H	-1.93387100	0.86860000	-2.14366900
H	-1.11441200	-0.30287100	1.95867400
H	-3.37073600	-1.25796500	2.12428900
C	-0.06367100	0.93723800	-0.29817400
H	0.16850000	1.37979400	-1.27414500
N	0.87893500	1.00290100	0.58265700
C	2.24273100	1.53542400	0.34902100
H	2.42532800	2.30719300	1.10792800
H	2.21205200	2.02467600	-0.63888000
C	3.24907600	0.43806300	0.40484900
H	4.00503200	0.45951900	1.19207800
C	3.33900200	-0.57071500	-0.68591200
H	2.33560600	-0.76083500	-1.11057700
C	3.98510000	-1.88286100	-0.24707800
H	3.39988900	-2.36629600	0.54747200
H	4.06356700	-2.58260100	-1.08829000
H	4.99844700	-1.70551100	0.13927100
H	3.92418500	-0.13638700	-1.52154000
H	0.72322700	0.58495100	1.50159300

M06-2x/def2svp, SMD = MeCN, correction to Gibbs free energy = 0.195651 Hartree
M06-2x/def2tzvp, SMD = MeCN, E = -482.703789 Hartree

C	-3.91777200	0.71078100	-0.13091100
C	-3.50671700	0.19453300	1.09795300
C	-2.22360100	-0.32719500	1.23159300
C	-1.34997000	-0.33539100	0.13214000
C	-1.76898100	0.18991000	-1.10401700
C	-3.04982200	0.70975100	-1.22906700
H	-4.92380200	1.12004600	-0.23847600
H	-4.18562300	0.19886300	1.95136100
H	-1.88639500	-0.73402900	2.18685700
H	-1.10201900	0.19829400	-1.96838200
H	-3.37820500	1.11768500	-2.18561800
C	-0.03197200	-0.90474400	0.34938100
H	0.21336400	-1.25985900	1.35674400
N	0.88409700	-1.03943800	-0.54175500
C	2.24374400	-1.55406600	-0.28751300
H	2.43095000	-2.34375400	-1.02475200
H	2.22215900	-2.00149500	0.71860400
C	3.24100800	-0.45129500	-0.38746200
H	3.96478800	-0.46513300	-1.20485700
C	3.32077200	0.59872500	0.66424800
H	2.31701500	0.77619800	1.09060900
C	3.92148700	1.90859300	0.16393100
H	3.30838800	2.33864200	-0.64193300
H	3.99019000	2.64849700	0.97341800
H	4.93429500	1.74602900	-0.23404200
H	3.92700600	0.21601100	1.51008100
H	0.71015200	-0.71755600	-1.49898500

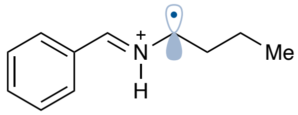

M06-2x/def2svp, gas phase, correction to enthalpy = 0.250044 Hartree
M06-2x/def2tzvp, gas phase, E = -482.630202 Hartree

C	-4.33824100	0.24266000	0.00025900
C	-3.58474800	1.41942200	-0.00011300
C	-2.20034400	1.35231100	-0.00030100
C	-1.54215300	0.09869600	-0.00010900
C	-2.32166400	-1.08484300	0.00031700
C	-3.70290100	-1.00692900	0.00047700
H	-5.42770100	0.29450000	0.00040000
H	-4.08271100	2.38903800	-0.00025500
H	-1.60758300	2.26902400	-0.00062200
H	-1.86151700	-2.07600400	0.00060800
H	-4.29782100	-1.92035500	0.00079700
C	-0.11804500	0.10755700	-0.00034700

H	0.42265900	1.05400300	-0.00047100
N	0.63950400	-1.01075300	-0.00052300
C	1.96804800	-1.08946000	-0.00036900
H	2.38004400	-2.10067500	-0.00062400
C	2.84224000	0.10405200	0.00008200
H	2.59460100	0.72964800	-0.88038400
H	2.59449600	0.72911500	0.88088300
C	4.33154600	-0.24593400	0.00005100
H	4.55255700	-0.86517100	0.88328700
C	5.20891200	0.99841800	0.00048500
H	5.02153600	1.61641800	0.89047000
H	6.27140300	0.72562500	0.00043900
H	5.02161400	1.61698600	-0.88912200
H	4.55265300	-0.86462900	-0.88354100
H	0.13333200	-1.89795800	-0.00079900

M06-2x/def2svp, SMD = MeCN, correction to Gibbs free energy = 0.198025 Hartree
M06-2x/def2tzvp, SMD = MeCN, E = -482.725644 Hartree

C	-4.33977800	0.24678500	0.00521800
C	-3.57927800	1.41946700	-0.00319800
C	-2.19333200	1.34718900	-0.00649000
C	-1.53880300	0.09350400	-0.00177000
C	-2.32028100	-1.08563100	0.00761600
C	-3.70531200	-1.00059000	0.01081400
H	-5.42945600	0.30265400	0.00777700
H	-4.07203000	2.39256700	-0.00720000
H	-1.59168500	2.25813100	-0.01311400
H	-1.85696400	-2.07425600	0.01345700
H	-4.30003200	-1.91494500	0.01804400
C	-0.10810600	0.10566200	-0.00626400
H	0.42701600	1.05489200	-0.00728500
N	0.64700000	-1.01546900	-0.01015800
C	1.96663400	-1.08776000	-0.00978500
H	2.37657900	-2.09961200	-0.01451100
C	2.83685400	0.10594300	-0.00233500
H	2.58251700	0.73117400	-0.87990000
H	2.57683800	0.72371200	0.87885300
C	4.32256200	-0.24370000	0.00105500
H	4.54016800	-0.86877800	0.88106900
C	5.19902900	0.99987000	0.01277600
H	5.00159300	1.61281800	0.90517800
H	6.26464100	0.73237600	0.01505900
H	5.00839200	1.62489700	-0.87269500
H	4.54703600	-0.85716200	-0.88537700
H	0.15525000	-1.91461300	-0.01407000

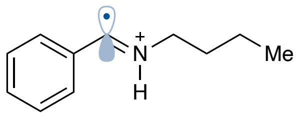

M06-2x/def2svp, gas phase, correction to enthalpy = 0.250572 Hartree
M06-2x/def2tzvp, gas phase, E = -482.590075 Hartree

C	4.44828100	0.08926100	0.38284900
C	3.80747600	1.29981500	0.10611400
C	2.45388700	1.31014300	-0.19940400
C	1.73708500	0.09525600	-0.20036100
C	2.39147200	-1.12992800	0.06824900
C	3.74590400	-1.12189300	0.35915600
H	5.51502900	0.08544700	0.61222300
H	4.36977900	2.23339900	0.12052500
H	1.93904000	2.24346300	-0.43127300
H	1.84360500	-2.07348500	0.02867700
H	4.26374200	-2.05912300	0.56378800
C	0.35009600	0.11461800	-0.49987000
N	-0.67093300	-0.59264500	-0.23387900
C	-2.02368900	-0.38017700	-0.80211800
H	-2.28601700	-1.29558100	-1.35241700
H	-1.93630600	0.44316000	-1.52339400
C	-3.04014000	-0.07723800	0.28876500
H	-3.05371100	-0.90306300	1.02055600
H	-2.73147500	0.83008700	0.83211100
C	-4.44116300	0.11224700	-0.29227000
H	-4.41859400	0.92977200	-1.03075000
C	-5.47351800	0.41686900	0.78664100
H	-5.21826200	1.33774500	1.33033800
H	-6.47020500	0.55165200	0.34796400
H	-5.53628600	-0.40197100	1.51771100
H	-4.73133800	-0.79680700	-0.84341800
H	-0.58661400	-1.35001300	0.45800600

M06-2x/def2svp, SMD = MeCN, correction to Gibbs free energy = 0.195839 Hartree
M06-2x/def2tzvp, SMD = MeCN, E = -482.682102 Hartree

C	-4.43448200	0.08765500	-0.39764100
C	-3.79931300	1.29600800	-0.10138200
C	-2.44573600	1.30757800	0.21281900
C	-1.72759900	0.09813100	0.20699600
C	-2.36965500	-1.12356200	-0.08358600
C	-3.72468000	-1.11758100	-0.38504900
H	-5.49947100	0.08225000	-0.63600400
H	-4.36413600	2.22864400	-0.10926100
H	-1.93103000	2.23811200	0.45670000
H	-1.80676200	-2.05868700	-0.06309400
H	-4.23378100	-2.05546800	-0.61004700
C	-0.33398900	0.12095800	0.52142300

N	0.67134800	-0.60138700	0.26897000
C	2.02159500	-0.39276100	0.82301000
H	2.29274200	-1.32120400	1.34455400
H	1.94761800	0.41984000	1.55681300
C	3.01523100	-0.07081000	-0.28170900
H	3.00543900	-0.88350600	-1.02659100
H	2.69401200	0.84861500	-0.79665900
C	4.42506200	0.10503700	0.27452300
H	4.41694500	0.90595800	1.03166900
C	5.43859900	0.43286500	-0.81402400
H	5.17103000	1.36527900	-1.33403800
H	6.44721100	0.55802500	-0.39524400
H	5.48266600	-0.36929200	-1.56636100
H	4.72361100	-0.81701000	0.79942700
H	0.56426500	-1.39295300	-0.38693700

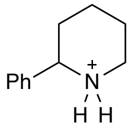

M06-2x/def2svp, gas phase, correction to enthalpy = 0.26748 Hartree
M06-2x/def2tzvp, gas phase, E = -483.293759 Hartree

C	2.63178900	-1.09639500	-0.74937900
C	3.38567000	0.16833300	-0.37451000
C	2.83727700	0.77771800	0.91514700
C	1.33704800	1.04681800	0.79429600
C	0.56075500	-0.21564000	0.43690400
H	4.44952400	-0.08610100	-0.27493600
H	2.74104200	-1.87454900	0.01905700
H	2.94657200	-1.51065000	-1.71555900
H	0.98196700	-0.13396800	-1.60214400
H	0.63005600	-1.64680900	-1.08635600
H	3.02371400	0.09142200	1.75713000
H	3.36767500	1.71037700	1.14659500
H	0.92084800	1.43203500	1.73522400
H	1.15332700	1.82268800	0.03262100
H	0.74551700	-0.98841100	1.19885500
H	3.31615400	0.89563800	-1.20103600
C	-0.92422600	-0.06131600	0.22563900
C	-1.79938000	-0.99971700	0.78321400
C	-1.43856200	0.98060100	-0.55778700
C	-3.17223200	-0.89862300	0.56385000
H	-1.40872700	-1.80808700	1.40644500
C	-2.81012400	1.07613100	-0.78109100
H	-0.77882900	1.73727500	-0.99025100
C	-3.67703100	0.13755200	-0.21997700
H	-3.84839400	-1.62835200	1.01024900
H	-3.20498500	1.89152000	-1.38794300
H	-4.75109200	0.21851600	-0.39086700

N	1.16138900	-0.80218800	-0.83927500

M06-2x/def2svp, SMD = MeCN, correction to Gibbs free energy = 0.211828 Hartree
M06-2x/def2tzvp, SMD = MeCN, E = -483.383954 Hartree

C	2.62823000	-1.23979900	-0.53965000
C	3.40500500	0.06034700	-0.43110200
C	2.87095700	0.92713800	0.70726100
C	1.37452700	1.19652800	0.54346600
C	0.54845400	-0.08500100	0.49403300
H	4.46715700	-0.17926400	-0.28201100
H	2.73116400	-1.84080900	0.37479500
H	2.94522500	-1.84633600	-1.39728600
H	1.00548200	-0.42800300	-1.56150300
H	0.63571300	-1.80851000	-0.75572500
H	3.04649600	0.41507300	1.66795900
H	3.42077500	1.87866100	0.74391600
H	0.99625400	1.80891000	1.37447600
H	1.20755600	1.77189400	-0.38247000
H	0.79305600	-0.72243500	1.36083500
H	3.32620400	0.60694200	-1.38582000
C	-0.90565400	0.04329100	0.32465900
C	-1.77290700	-1.05307500	0.70655800
C	-1.53167900	1.12789700	-0.40041000
C	-3.12156800	-1.03885200	0.43199300
H	-1.33826400	-1.90484300	1.24219000
C	-2.88315900	1.12776800	-0.66623600
H	-0.92888800	1.97762300	-0.73351500
C	-3.73182700	0.05170300	-0.25899100
H	-3.73850300	-1.88250100	0.75814400
H	-3.31505400	1.97893100	-1.20251200
H	-4.79988200	0.05727500	-0.47939200
N	1.18046400	-0.94146900	-0.68736600

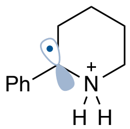

M06-2x/def2svp, gas phase, correction to enthalpy = 0.253566 Hartree
M06-2x/def2tzvp, gas phase, E = -482.63661 Hartree

C	2.36458800	-1.30410600	0.38972900
C	3.30685900	-0.11258600	0.41233500
C	2.54487100	1.20130100	0.58076500
C	1.45311200	1.34871900	-0.49757300
C	0.55895900	0.15485300	-0.50112100
H	4.02554100	-0.26110800	1.22996000
H	1.79481700	-1.39860800	1.32417400
H	2.87494300	-2.25162000	0.17375400
H	1.82061400	-1.03338600	-1.60239100

H	0.69839600	-1.89101400	-0.76424800
H	2.07183900	1.23138600	1.57434800
H	3.23973200	2.04942800	0.52492000
H	0.87177000	2.26075400	-0.33145400
H	1.93462900	1.46095400	-1.48815900
H	3.89760800	-0.09729300	-0.51972100
C	-0.82852600	0.05565700	-0.22312700
C	-1.49816900	-1.18816500	-0.03879300
C	-1.61179400	1.23982700	-0.12042300
C	-2.85762600	-1.23680800	0.21998100
H	-0.96921200	-2.14476900	-0.06457400
C	-2.97007600	1.17649000	0.13761100
H	-1.14719800	2.21413700	-0.26480800
C	-3.60512900	-0.05798100	0.30827500
H	-3.34294700	-2.20275700	0.36206800
H	-3.54636700	2.09973900	0.20315600
H	-4.67526100	-0.10265500	0.51097300
N	1.32981200	-1.08519900	-0.69628000

M06-2x/def2svp, SMD = MeCN, correction to Gibbs free energy = 0.205644 Hartree
M06-2x/def2tzvp, SMD = MeCN, E = -482.740075 Hartree

C	-2.36367500	-1.28978500	-0.41165200
C	-3.29132300	-0.08958200	-0.44185200
C	-2.51133800	1.21592700	-0.57608200
C	-1.44687300	1.33111800	0.53245000
C	-0.56791400	0.12788900	0.52169600
H	-3.98992600	-0.21980400	-1.27978500
H	-1.78500800	-1.39025900	-1.33897300
H	-2.88776700	-2.22849300	-0.19848200
H	-1.85426100	-1.04966100	1.58889100
H	-0.75331300	-1.93263400	0.76161400
H	-2.01009800	1.25028200	-1.55614100
H	-3.19394500	2.07479300	-0.51994400
H	-0.86176600	2.24788700	0.41324100
H	-1.95923700	1.39078100	1.51039800
H	-3.89044800	-0.07644300	0.48347300
C	0.82387900	0.04454400	0.23252500
C	1.50173400	-1.19246300	0.04805700
C	1.59626700	1.23396800	0.12011200
C	2.86276200	-1.22880400	-0.21914800
H	0.97196900	-2.14512000	0.09465000
C	2.95587500	1.18137800	-0.14587100
H	1.12259600	2.20532600	0.25620400
C	3.60328200	-0.04701800	-0.31599000
H	3.35250900	-2.19403000	-0.35749800
H	3.52166300	2.11158100	-0.21956400
H	4.67346000	-0.08306200	-0.52391300
N	-1.34749900	-1.09773900	0.68862300

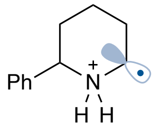

M06-2x/def2svp, gas phase, correction to enthalpy = 0.252568 Hartree
M06-2x/def2tzvp, gas phase, E = -482.620931 Hartree

C	2.61583400	-1.08350200	-0.78005600
C	3.44265700	0.07264500	-0.36089600
C	2.87808600	0.73833600	0.90290400
C	1.38870600	1.04021500	0.74114500
C	0.58890800	-0.21924100	0.43448100
H	4.47616500	-0.26620800	-0.21269400
H	2.97474200	-1.95809000	-1.32080700
H	0.92797400	-0.21373600	-1.62445100
H	0.66289400	-1.71948500	-1.03777700
H	3.02723500	0.07008400	1.76469200
H	3.43106400	1.66250000	1.11306300
H	0.96948200	1.47676900	1.65824600
H	1.23460700	1.78550800	-0.05723800
H	0.78357000	-0.97667800	1.20827200
H	3.48026300	0.81887000	-1.18130800
C	-0.89404400	-0.06020800	0.22774800
C	-1.76902700	-0.99804800	0.78710600
C	-1.40798500	0.98373000	-0.55353700
C	-3.14211200	-0.89434700	0.57122200
H	-1.37769900	-1.80711100	1.40907500
C	-2.78005800	1.08167500	-0.77285100
H	-0.74802100	1.74040100	-0.98541900
C	-3.64683300	0.14362400	-0.21052000
H	-3.81858000	-1.62301200	1.01886300
H	-3.17540600	1.89873600	-1.37711400
H	-4.72121500	0.22666400	-0.37846400
N	1.17544800	-0.84921200	-0.84534600

M06-2x/def2svp, SMD = MeCN, correction to Gibbs free energy = 0.204429 Hartree
M06-2x/def2tzvp, SMD = MeCN, E = -482.723593 Hartree

C	2.66659900	-0.75497400	-1.04189000
C	3.44764100	0.21353800	-0.23792800
C	2.82567600	0.41605500	1.14875700
C	1.33568500	0.72776600	1.03071900
C	0.59567100	-0.37273500	0.27738500
H	4.48646700	-0.13459700	-0.16919300
H	3.06025700	-1.35617600	-1.85941800
H	1.02936700	0.33787000	-1.64356000
H	0.76363100	-1.28446500	-1.60342600
H	2.95915700	-0.50031300	1.74446100
H	3.34161200	1.23103400	1.67361100

H	0.87169100	0.81809700	2.02256900
H	1.18682800	1.68798500	0.50892200
H	0.77912800	-1.34061300	0.76442100
H	3.47068800	1.18744500	-0.76791600
C	-0.89123000	-0.15728800	0.12707000
C	-1.78041700	-1.13747500	0.57916700
C	-1.39799200	1.01454800	-0.45036900
C	-3.15794000	-0.94958000	0.46093300
H	-1.38790100	-2.05189700	1.02961100
C	-2.77389100	1.19799500	-0.57606100
H	-0.71581800	1.79047900	-0.80786200
C	-3.65601800	0.21742200	-0.11794300
H	-3.84301200	-1.71893600	0.82096300
H	-3.15910400	2.11257200	-1.02986400
H	-4.73327300	0.36470700	-0.21292800
N	1.22965500	-0.51926100	-1.09563200

HCl
M06-2x/def2svp, SMD = MeCN, correction to Gibbs free energy = -0.011224 Hartree
M06-2x/def2tzvp, SMD = MeCN, E = -460.803466 Hartree

Cl	0.00000000	0.00000000	0.07180600
H	0.00000000	0.00000000	-1.22070000

Chlorine radical
M06-2x/def2svp, SMD = MeCN, correction to Gibbs free energy = -0.015677 Hartree
M06-2x/def2tzvp, SMD = MeCN, E = -460.134897 Hartree

Cl	0.00000000	0.00000000	0.00000000

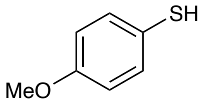

M06-2x/def2svp, SMD = MeCN, correction to Gibbs free energy = 0.099564 Hartree
M06-2x/def2tzvp, SMD = MeCN, E = -744.943398 Hartree

C	-0.15160500	0.71823100	1.21108300
C	-0.15160500	-0.67505100	1.20919400
C	-0.15816100	-1.37822600	0.00000000
C	-0.15160500	-0.67505100	-1.20919400
C	-0.15160500	0.71823100	-1.21108300
C	-0.15566700	1.41517700	0.00000000
H	-0.15490600	1.27906800	2.14751300
H	-0.15074200	-1.22305700	2.15295300
H	-0.15074200	-1.22305700	-2.15295300
H	-0.15490600	1.27906800	-2.14751300
S	-0.11131500	-3.17039800	0.00000000
H	-1.44679600	-3.37242900	0.00000000

O	-0.18508000	2.77828200	0.00000000
C	1.09413200	3.38906700	0.00000000
H	1.66920000	3.10583700	-0.89697900
H	0.93808000	4.47456900	0.00000000
H	1.66920000	3.10583700	0.89697900

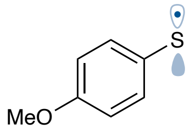

M06-2x/def2svp, SMD = MeCN, correction to Gibbs free energy = 0.090136 Hartree
M06-2x/def2tzvp, SMD = MeCN, E = -744.315338 Hartree

C	-0.16329600	0.65825400	1.21886700
C	-0.16329600	-0.72596100	1.21901500
C	-0.16161000	-1.45241900	0.00000000
C	-0.16329600	-0.72596100	-1.21901500
C	-0.16329600	0.65825400	-1.21886700
C	-0.16571800	1.35449200	0.00000000
H	-0.16964000	1.22535800	2.15124300
H	-0.16641200	-1.27798000	2.16034400
H	-0.16641200	-1.27798000	-2.16034400
H	-0.16964000	1.22535800	-2.15124300
S	-0.15997700	-3.16879000	0.00000000
O	-0.19720200	2.70885700	0.00000000
C	1.07936400	3.33449600	0.00000000
H	1.65389400	3.05547200	-0.89781800
H	0.90845800	4.41716500	0.00000000
H	1.65389400	3.05547200	0.89781800

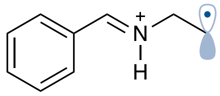

M06-2x/def2svp, SMD = MeCN, correction to Gibbs free energy = 0.142975 Hartree
M06-2x/def2tzvp, SMD = MeCN, E = -404.081301 Hartree

C	-3.25436500	0.33813200	0.12042200
C	-2.82889400	-0.98933500	0.16707300
C	-1.47139200	-1.28138600	0.07587400
C	-0.53654100	-0.24277400	-0.06625100
C	-0.97194000	1.09461600	-0.10953800
C	-2.32727900	1.37794600	-0.01610800
H	-4.31850500	0.56978800	0.19222100
H	-3.55444900	-1.79620000	0.27529400
H	-1.12291700	-2.31532100	0.11204800
H	-0.26083100	1.91631000	-0.21223200
H	-2.66803100	2.41328500	-0.04892200
C	0.85968400	-0.62856300	-0.16087700
H	1.10396800	-1.69375800	-0.08060200

N	1.84987200	0.17162600	-0.33604100
C	3.26071900	-0.23017000	-0.40134300
H	3.65224100	0.10174900	-1.37259300
H	3.27689200	-1.33328100	-0.39102500
C	4.03156700	0.35832300	0.72240400
H	5.06486000	0.66560600	0.56643100
H	3.62855100	0.31011900	1.73522700
H	1.67976600	1.17958800	-0.41349400

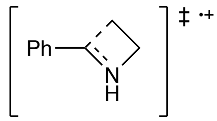

M06-2x/def2svp, SMD = MeCN, correction to Gibbs free energy = 0.145054 Hartree
M06-2x/def2tzvp, SMD = MeCN, E = -404.040659 Hartree

C	1.10683100	-0.43007200	-0.36572100
C	2.60849300	-0.45347100	1.09586200
C	3.24560000	0.20245000	-0.10122500
H	1.41300700	-1.47128600	-0.52917700
H	2.85570000	-1.47673100	1.38640200
H	2.17710200	0.21010600	1.84914300
H	3.86426500	-0.47801300	-0.70093700
H	3.80510600	1.12299100	0.10957100
C	-0.31265700	-0.18237600	-0.17619000
C	-1.19430200	-1.27599700	-0.21251200
C	-0.80859300	1.11288000	0.05438500
C	-2.55942600	-1.07288000	-0.04304500
H	-0.79803500	-2.27930000	-0.38165600
C	-2.17449200	1.30885100	0.21956500
H	-0.12700600	1.96360600	0.12023900
C	-3.04815700	0.21837500	0.16901900
H	-3.24544500	-1.92016800	-0.07607700
H	-2.56191400	2.31274600	0.39758200
H	-4.11955500	0.37728100	0.30373900
N	1.98201400	0.51278500	-0.77492400
H	1.68289600	1.48270800	-0.89519300

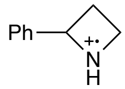

M06-2x/def2svp, SMD = MeCN, correction to Gibbs free energy = 0.14485 Hartree
M06-2x/def2tzvp, SMD = MeCN, E = -404.062655 Hartree

C	1.21767700	0.67741200	0.04161100
C	2.19779500	0.19492600	-1.06850200
C	3.04240600	-0.43467800	0.05385200
H	1.33222700	1.75538300	0.26452900

H	2.67692600	0.99212100	-1.64370600
H	1.74303300	-0.54969100	-1.73079500
H	3.99883100	0.06029000	0.30276700
H	3.21798500	-1.52390100	0.03678400
C	-0.24171700	0.30590500	0.02517900
C	-1.22356600	1.29913900	0.06678600
C	-0.61390200	-1.04291800	-0.04500600
C	-2.57245800	0.94623800	0.02938000
H	-0.92928000	2.34885500	0.12631600
C	-1.96223400	-1.39192600	-0.07247900
H	0.15306200	-1.82187300	-0.08144800
C	-2.94216000	-0.39739700	-0.03749600
H	-3.33693100	1.72445100	0.05651800
H	-2.24951300	-2.44330900	-0.12383600
H	-3.99817500	-0.67167300	-0.06264400
N	2.01831100	-0.06916500	0.98896800
H	1.85261800	-0.32670400	1.97279300

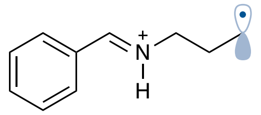

M06-2x/def2svp, SMD = MeCN, correction to Gibbs free energy = 0.169527 Hartree
M06-2x/def2tzvp, SMD = MeCN, E = -443.391043 Hartree

C	-3.78959700	-0.31173400	-0.23716600
C	-3.35157600	1.01247700	-0.23049100
C	-1.99919700	1.28973700	-0.05578100
C	-1.08248000	0.23949600	0.11486200
C	-1.53017500	-1.09464100	0.10631300
C	-2.88043500	-1.36291300	-0.06968100
H	-4.84970000	-0.53189600	-0.37469100
H	-4.06339400	1.82810500	-0.36167800
H	-1.64038500	2.32072600	-0.04856500
H	-0.83293000	-1.92492300	0.23354500
H	-3.23138500	-2.39532800	-0.07790100
C	0.31010200	0.60911000	0.29183000
H	0.56734100	1.67363200	0.26074000
N	1.28263500	-0.20773900	0.48724000
C	2.68828600	0.16920300	0.61980600
H	3.06725500	-0.29517700	1.54013600
H	2.73611100	1.26037200	0.72351300
C	3.49862200	-0.31003400	-0.59565700
H	3.35956200	-1.39871600	-0.69336900
H	3.07503300	0.16573600	-1.49359900
C	4.94115500	0.02371500	-0.44808300
H	5.31275700	1.00683000	-0.74196300

H	5.60138600	-0.62711600	0.12783400
H	1.09168300	-1.21456100	0.51960300

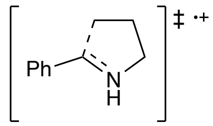

M06-2x/def2svp, SMD = MeCN, correction to Gibbs free energy = 0.172651 Hartree
M06-2x/def2tzvp, SMD = MeCN, E = -443.377056 Hartree

C	3.03280500	0.67905200	-0.45914600
C	3.20991300	-0.59792200	0.38652500
C	1.96958600	-0.82618300	1.18854700
C	0.79185800	-0.17779500	-0.50395000
H	3.38619900	1.57226500	0.07027400
H	3.56683100	0.61268100	-1.41658400
H	3.40220100	-1.46624200	-0.25709900
H	4.08074800	-0.48009800	1.05094100
H	1.64911400	-0.05622100	1.89609400
H	1.13798000	-1.14125600	-0.89435600
C	-0.64343500	-0.04983200	-0.25606200
C	-1.45202700	-1.16963200	-0.50600900
C	-1.21929800	1.12932900	0.24603100
C	-2.82308100	-1.10682500	-0.27647700
H	-0.99460900	-2.08585200	-0.88540700
C	-2.58996800	1.18516700	0.47387000
H	-0.60354200	2.00229200	0.47440900
C	-3.39201400	0.07052200	0.21185300
H	-3.44902100	-1.97738800	-0.47688200
H	-3.03666600	2.09988700	0.86572400
H	-4.46649200	0.11995800	0.39657700
N	1.59595700	0.86091000	-0.66962500
H	1.21878500	1.80247400	-0.55998700
H	1.63073900	-1.84416300	1.39257200

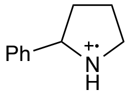

M06-2x/def2svp, SMD = MeCN, correction to Gibbs free energy = 0.174097 Hartree
M06-2x/def2tzvp, SMD = MeCN, E = -443.410358 Hartree

C	2.89608000	0.73624600	-0.50538700
C	3.13097600	-0.30276000	0.59047700
C	1.71192200	-0.61463300	1.07007000
C	0.88707700	-0.57507900	-0.23014500
H	2.81342200	1.76653600	-0.09783900
H	3.62878400	0.78108200	-1.32565200
H	3.59555000	-1.19749200	0.15272500

H	3.78104400	0.08162900	1.38395100
H	1.34655500	0.17421400	1.74460600
H	1.00843000	-1.53776700	-0.77141900
C	-0.58231000	-0.25100300	-0.12304100
C	-1.54185200	-1.24249700	-0.34381200
C	-0.98607000	1.04161800	0.23515800
C	-2.89723900	-0.94545600	-0.20253700
H	-1.22421200	-2.24852100	-0.62617400
C	-2.34150500	1.33779500	0.36574100
H	-0.23813000	1.81839300	0.41792100
C	-3.29805700	0.34401700	0.14940300
H	-3.64295400	-1.72390000	-0.37173700
H	-2.65170000	2.34707100	0.64121700
H	-4.35916700	0.57593000	0.25583400
N	1.60571300	0.39559300	-1.01918200
H	1.21365500	0.80824400	-1.87575600
H	1.61459600	-1.58404500	1.57104000

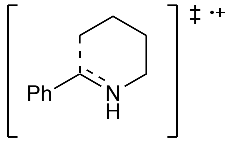

M06-2x/def2svp, SMD = MeCN, correction to Gibbs free energy = 0.199397 Hartree
M06-2x/def2tzvp, SMD = MeCN, E = -482.698948 Hartree

C	-2.69137000	-0.98949400	-0.76918500
C	-3.36747700	-0.23338300	0.37471900
C	-2.86166300	1.20487600	0.52335300
C	-1.44466300	1.32080800	0.97129100
C	-0.44632300	-0.09540700	-0.71661500
H	-4.44735700	-0.22595100	0.17077100
H	-2.83073900	-0.45576900	-1.71967800
H	-3.09239900	-2.00412100	-0.86989500
H	-0.93539900	-1.90022200	0.02033200
H	-3.00800600	1.75489700	-0.42074500
H	-3.49713700	1.72015400	1.27058000
H	-0.89308800	2.24571000	0.78866400
H	-1.09578300	0.69452500	1.79715100
H	-0.80156800	0.70073500	-1.37878000
H	-3.21650400	-0.78577700	1.31604900
C	0.96195200	-0.08955800	-0.33227500
C	1.77439200	0.92154500	-0.86744600
C	1.51155700	-1.04115900	0.54253300
C	3.12762600	0.97146900	-0.54642100
H	1.33514300	1.66222800	-1.53901500
C	2.86344900	-0.98465200	0.86041900
H	0.88963200	-1.82143300	0.98609800

C	3.67153800	0.01813100	0.31548700
H	3.75888700	1.75481400	-0.96771600
H	3.29115900	-1.72292400	1.53988000
H	4.73212600	0.05715000	0.56978700
N	-1.25472500	-1.09901100	-0.52837800

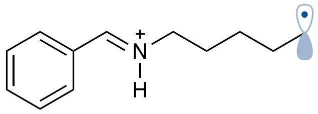

M06-2x/def2svp, SMD = MeCN, correction to Gibbs free energy = 0.222982 Hartree
M06-2x/def2tzvp, SMD = MeCN, E = -522.007564 Hartree

C	4.86856000	0.39349100	-0.45211300
C	4.45459100	-0.93636800	-0.52727300
C	3.13104900	-1.26284100	-0.24772200
C	2.21866500	-0.25568800	0.10667600
C	2.64233900	1.08357500	0.18266500
C	3.96414100	1.40126400	-0.09749000
H	5.90621600	0.65181600	-0.67055300
H	5.16269300	-1.71838100	-0.80317500
H	2.79161500	-2.29902400	-0.30188600
H	1.94920500	1.88005700	0.45973500
H	4.29640000	2.43831000	-0.03982900
C	0.85612000	-0.67264000	0.38461100
H	0.62150400	-1.74105100	0.32042700
N	-0.11345600	0.10549500	0.70732900
C	-1.48586200	-0.32323000	0.97428900
H	-1.74669100	0.03397300	1.98077000
H	-1.49390500	-1.42067600	0.97715400
C	-2.44934100	0.23865900	-0.06202000
H	-2.36574200	1.33761600	-0.07947300
H	-2.15664200	-0.12732800	-1.05902700
C	-3.88732400	-0.16632800	0.24381200
H	-3.96184000	-1.26578000	0.28189400
C	-4.88302200	0.37268500	-0.79839600
H	-4.78951600	1.47100600	-0.82928400
H	-4.17114100	0.20366200	1.24294000
H	0.05431800	1.11485100	0.77108300
H	-4.58106800	-0.00328300	-1.79017400
C	-6.29053600	-0.01606500	-0.50743900
H	-6.89067700	0.56338300	0.19755600
H	-6.66682000	-0.99670500	-0.80706200

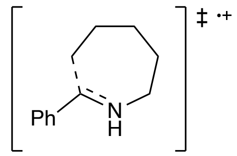

M06-2x/def2svp, SMD = MeCN, correction to Gibbs free energy = 0.226573 Hartree
M06-2x/def2tzvp, SMD = MeCN, E = -522.002857 Hartree

C	3.36369100	0.63388800	-0.37317700
C	2.32016400	1.75590300	-0.40908800
C	0.99238500	1.40719400	-0.97641200
C	3.16859500	-0.44596500	0.70200700
C	0.23354700	-0.37723600	0.47830900
C	2.33548100	-1.65508300	0.30392000
H	3.42827800	0.15733700	-1.36609900
H	2.19954300	2.19003100	0.59799300
H	0.94065100	0.76420600	-1.85973100
H	0.70252600	0.25629700	1.23535500
H	2.79344200	0.00131100	1.63678400
H	2.37970500	-2.42526400	1.08892700
H	4.34031100	1.10587700	-0.19471200
H	2.72702400	2.57922000	-1.03033800
H	0.15365700	2.08891100	-0.81957200
H	4.15229500	-0.86652300	0.95656600
H	2.73801800	-2.10240700	-0.61503500
H	0.45658900	-2.05861200	-0.57858700
N	0.91993700	-1.38658800	0.03600700
C	-1.20107100	-0.20697500	0.24013600
C	-1.89267700	0.69144500	1.06667500
C	-1.88739500	-0.89656100	-0.77170300
C	-3.26105400	0.88200800	0.89880800
H	-1.34947200	1.23415900	1.84339800
C	-3.25379000	-0.69715200	-0.93774700
H	-1.36261000	-1.57766000	-1.44551800
C	-3.94169400	0.18753800	-0.10247600
H	-3.79699800	1.57559200	1.54804300
H	-3.78586000	-1.23034100	-1.72665300
H	-5.01375600	0.33995400	-0.23837900

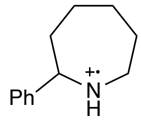

M06-2x/def2svp, SMD = MeCN, correction to Gibbs free energy = 0.229362 Hartree
M06-2x/def2tzvp, SMD = MeCN, E = -522.026882 Hartree

C	-1.50728300	1.92706600	-0.40339600
C	-2.14890800	-1.49871300	0.91634900
C	-2.84191600	1.37787900	0.13984300

C	-3.37210600	-1.15272200	0.06029800
C	-3.36266000	0.18798300	-0.68668500
H	-1.65150200	2.45860400	-1.35021300
H	-2.71754300	1.10172400	1.19680200
H	-2.13767500	-0.88506800	1.83141000
H	-4.24781200	-1.17939900	0.72842000
H	-0.27601800	0.67460700	-1.60732900
H	-1.05017600	2.60423600	0.33944000
H	-2.28485700	-2.53383400	1.26410400
H	-3.53701400	2.22773800	0.12015000
H	-3.52989400	-1.95303000	-0.68021300
H	-4.38708600	0.39271800	-1.02810200
H	-2.76765100	0.09053500	-1.60956400
C	-0.07522300	-0.03427300	0.36634600
H	-0.31496400	0.41320900	1.34139000
C	-0.76615000	-1.42031500	0.26351000
H	-0.79467600	-1.74481800	-0.78876500
H	-0.08292400	-2.10254400	0.78971100
C	1.43675900	-0.07050000	0.18090700
C	2.21005400	0.90615900	0.81908300
C	2.05614600	-1.01747000	-0.64217100
C	3.59432600	0.92239500	0.65488800
H	1.72266600	1.64883600	1.45538200
C	3.44070500	-0.99583500	-0.80739400
H	1.46277200	-1.77831100	-1.15229300
C	4.21079500	-0.02881600	-0.15912800
H	4.19224500	1.67984100	1.16427600
H	3.92026500	-1.74042800	-1.44484700
H	5.29427300	-0.01679000	-0.28946200
N	-0.57995200	0.86073500	-0.64214300

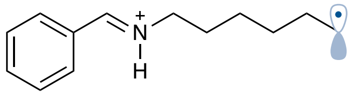

M06-2x/def2svp, SMD = MeCN, correction to Gibbs free energy = 0.248605 Hartree
M06-2x/def2tzvp, SMD = MeCN, E = -561.315595 Hartree

C	-5.39138300	0.62278500	0.45582900
C	-5.02312400	-0.66951300	0.82979100
C	-3.72185900	-1.10733700	0.60346100
C	-2.78743800	-0.25225900	-0.00334000
C	-3.16362100	1.05174600	-0.37517200
C	-4.46281300	1.48185800	-0.14342700
H	-6.41104300	0.96928900	0.63309300
H	-5.74904700	-1.33490400	1.29825800
H	-3.41709900	-2.11547700	0.89151400
H	-2.44963200	1.73668000	-0.83742500
H	-4.75781900	2.49235500	-0.42828800
C	-1.45387400	-0.78732500	-0.21259500

H	-1.24174800	-1.79488600	0.16196600
N	-0.48962900	-0.18528300	-0.81016700
C	0.85952800	-0.72029100	-0.98633600
H	1.08202800	-0.69382500	-2.06242800
H	0.84570400	-1.76613300	-0.65470900
C	1.87741500	0.10052800	-0.20571400
H	1.81334400	1.15413800	-0.52346000
H	1.61772800	0.06671200	0.86440200
C	3.29547000	-0.41831900	-0.41789300
H	3.34765500	-1.47701800	-0.11202900
C	4.33518500	0.38498700	0.35621200
H	4.28674800	1.44368100	0.05015800
H	3.53761000	-0.39167200	-1.49389400
H	-0.64193300	0.75802300	-1.18070800
H	4.09564400	0.35965200	1.43267100
C	5.76825600	-0.13593000	0.14603200
H	5.98954800	-0.10957900	-0.93422200
H	5.79844900	-1.19494900	0.45289600
C	6.78781600	0.64601500	0.89839500
H	7.18327800	1.57845900	0.48929500
H	6.98064300	0.43475900	1.95262000

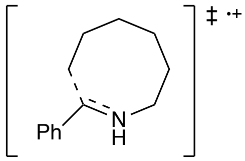

M06-2x/def2svp, SMD = MeCN, correction to Gibbs free energy = 0.256264 Hartree
M06-2x/def2tzvp, SMD = MeCN, E = -561.306075 Hartree

C	-1.90720000	-1.97649900	-0.47076400
C	-1.96063500	1.91873300	-0.16352800
C	-2.98104900	-0.91074500	-0.62940100
C	-3.23020400	1.38073600	0.49816000
C	-3.23849600	-0.13669500	0.67405800
H	-2.21307300	-2.72688500	0.26835900
H	-2.69149500	-0.22922700	-1.44380400
H	-1.96324600	1.74316400	-1.25319800
H	-4.09623300	1.69531900	-0.10514000
H	-0.19317900	-1.93591500	0.78080000
H	-1.72346000	-2.49465400	-1.42464900
H	-1.94243300	3.02195400	-0.07501600
H	-3.89139600	-1.41773900	-0.97880900
H	-3.34785200	1.85247300	1.48633300
H	-4.19699300	-0.44535500	1.11595200
H	-2.46983700	-0.40636200	1.41768100
C	0.01750200	-0.41145200	-0.51475600
H	-0.35789200	-0.03815200	-1.47382800

C	-0.66937900	1.44388600	0.40551000
H	-0.63604800	1.07308100	1.43510000
H	0.22397100	1.98000100	0.07964600
C	1.44910600	-0.20323500	-0.21146800
C	2.24345800	0.41988400	-1.18351700
C	2.02264200	-0.60521700	1.00307700
C	3.60359300	0.61356900	-0.95501800
H	1.78934100	0.74465900	-2.12223100
C	3.38179200	-0.40393000	1.22796600
H	1.41358400	-1.06376700	1.78649400
C	4.17438600	0.20136800	0.24994500
H	4.21920500	1.08914900	-1.71994100
H	3.82431400	-0.71705700	2.17469800
H	5.23920500	0.35689500	0.43111200
N	-0.63137000	-1.43771500	0.00555100

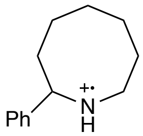

M06-2x/def2svp, SMD = MeCN, correction to Gibbs free energy = 0.257149 Hartree
M06-2x/def2tzvp, SMD = MeCN, E = -561.326401 Hartree

C	-1.50728300	1.92706600	-0.40339600
C	-2.14890800	-1.49871300	0.91634900
C	-2.84191600	1.37787900	0.13984300
C	-3.37210600	-1.15272200	0.06029800
C	-3.36266000	0.18798300	-0.68668500
H	-1.65150200	2.45860400	-1.35021300
H	-2.71754300	1.10172400	1.19680200
H	-2.13767500	-0.88506800	1.83141000
H	-4.24781200	-1.17939900	0.72842000
H	-0.27601800	0.67460700	-1.60732900
H	-1.05017600	2.60423600	0.33944000
H	-2.28485700	-2.53383400	1.26410400
H	-3.53701400	2.22773800	0.12015000
H	-3.52989400	-1.95303000	-0.68021300
H	-4.38708600	0.39271800	-1.02810200
H	-2.76765100	0.09053500	-1.60956400
C	-0.07522300	-0.03427300	0.36634600
H	-0.31496400	0.41320900	1.34139000
C	-0.76615000	-1.42031500	0.26351000
H	-0.79467600	-1.74481800	-0.78876500
H	-0.08292400	-2.10254400	0.78971100
C	1.43675900	-0.07050000	0.18090700
C	2.21005400	0.90615900	0.81908300
C	2.05614600	-1.01747000	-0.64217100
C	3.59432600	0.92239500	0.65488800

H	1.72266600	1.64883600	1.45538200
C	3.44070500	-0.99583500	-0.80739400
H	1.46277200	-1.77831100	-1.15229300
C	4.21079500	-0.02881600	-0.15912800
H	4.19224500	1.67984100	1.16427600
H	3.92026500	-1.74042800	-1.44484700
H	5.29427300	-0.01679000	-0.28946200
N	-0.57995200	0.86073500	-0.64214300

9) Proposed mechanism for TBADT mediated cyclization

Figure S12 outlines a plausible mechanism for the cyclization mediated by TBADT. Aldehyde **I** and amine **II** undergo condensation followed by protonation to generate iminium ion **III**. Light irradiation converts the photocatalyst **IV** to its excited state, **V**,[12] which then unselectively abstracts a hydrogen atom from one of multiple positions on the iminium ion, generating a mixture of radical species (**VII** and **VIII**). This HAA event simultaneously reduces the photocatalyst to **VI**. More slowly cyclizing radical intermediates (**VII**) undergo HAD with thiol reagent **X** to regenerate iminium **III**, whereas radical intermediate **VIII**, bearing the appropriately positioned radical, rapidly undergoes the favored six-endo cyclization to selectively furnish the six-membered ring, **XI**. Subsequent oxidation of photocatalyst **VI** [$E([W_{10}O_{32}]^{4-}/[W_{10}O_{32}]^{5-}) = -0.97$ V vs SCE[13,14]] by thiol radical **IX** or aminium radical cation **XI** regenerates the active catalyst and furnishes thiol anion **XII** and the saturated heterocycle **XIII**.

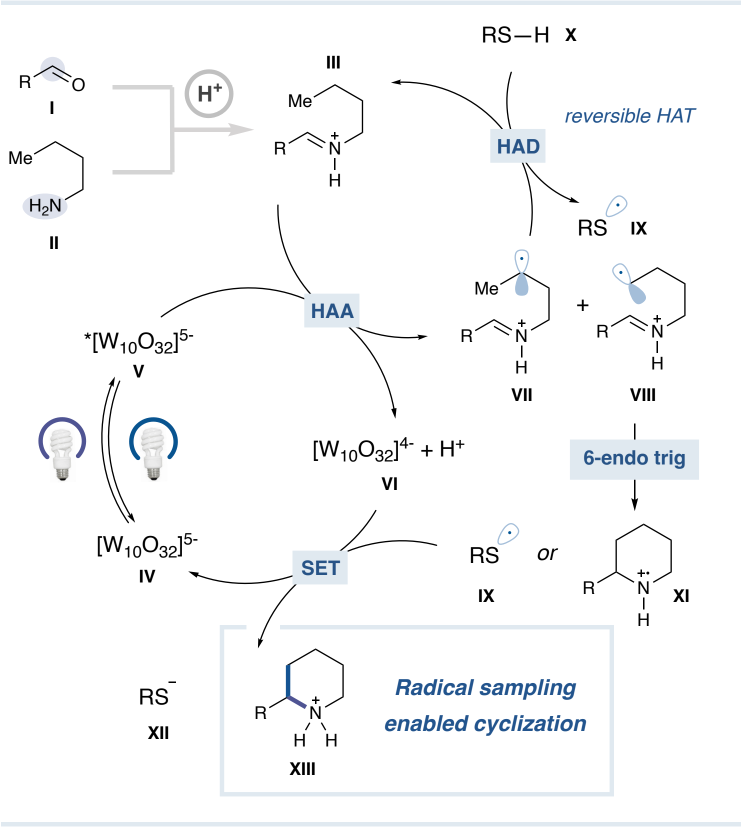

Figure S12. Proposed mechanism for TBADT mediated cyclization.

10) Piperidine scope via radical sampling

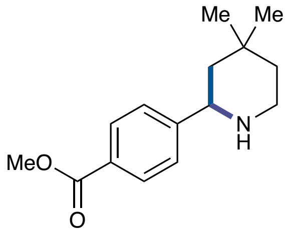

(±)-Methyl 4-(4,4-dimethylpiperidin-2-yl)benzoate (3)

Prepared according to **General Procedure A-1** with methyl 4-formylbenzoate (83.8 mg, 0.5 mmol, 1 eq, 98% purity) and 3,3-dimethyl-butylamine (58.6 mg, 77.9 µL, 0.55 mmol, 1.1 eq, 95% purity), benzenesulfonic acid (316.4 mg, 2.0 mmol, 4 equiv), $FeCl_3$ (16.2 mg, 0.1 mmol, 20 mol%), bis(4-methoxyphenyl) disulfide (41.8 mg, 0.15 mmol, 30 mol%) and anhydrous MeCN (2.5 mL). The reaction was irradiated for 12 hrs under 100% light intensity, 365 nm plate.

After working up according to **General Procedure A-1**, the mixture was purified by purification ***method (3) reverse phase chromatography***. The basified, dry organic mixture was loaded on 30 g reverse phase column with 30 to 65% MeCN in water, 0.1 % NH_4OH modifier. Pure fractions were combined concentrated yielding the title compound as an off-white solid after extensive drying (101.9 mg, 0.412 mmol, 82% yield).

^{1}H NMR (500 MHz, $CDCl_3$) δ 7.98 (d, J = 8.4 Hz, 2H), 7.43 (d, J = 8.3 Hz, 2H), 3.90 (s, 3H), 3.85 (dd, J = 11.8, 2.7 Hz, 1H), 3.07 – 2.93 (m, 2H), 1.53 – 1.43 (m, 2H), 1.42 – 1.31 (m, 2H), 1.07 (s, 3H), 0.97 (s, 3H).

^{13}C NMR (126 MHz, $CDCl_3$) δ 167.2, 150.8, 129.9, 129.0, 126.7, 57.4, 52.2, 48.1, 43.3, 38.6, 33.4, 30.4, 24.4.

HRMS (ESI-TOF) *m/z* calcd. for $C_{15}H_{22}NO_2^+$ ($[M+H]^+$) 248.1645, found 248.1641.

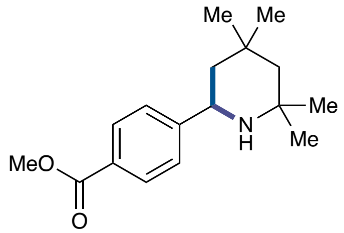

(±)-Methyl 4-(4,4,6,6-tetramethylpiperidin-2-yl)benzoate (4)

Prepared according to **General Procedure A-1** with methyl 4-formylbenzoate (83.8 mg, 0.5 mmol, 1 eq, 98% purity) and *tert*-octylamine (72.5 mg, 90 µL, 0.55 mmol, 1.1 eq, 98% purity), benzenesulfonic acid (316.4 mg, 2.0 mmol, 4 equiv), $FeCl_3$ (16.2 mg, 0.1 mmol, 20 mol%), bis(4-methoxyphenyl) disulfide (41.8 mg, 0.15 mmol, 30 mol%) and anhydrous MeCN (2.5 mL). The reaction was irradiated for 12 hrs under 100% light intensity, 365 nm plate.

After working up according to **General Procedure A-1**, the mixture was purified by purification ***method (3) reverse phase chromatography***. The basified, dry organic mixture was loaded on 30 g reverse phase column with 25 to 65% MeCN in water, 0.1 % NH_4OH modifier. Pure fractions were combined, and the acetonitrile was removed under reduced pressure. The resulting solution was further basified with 10 mL of saturated sodium bicarbonate, transferred to a separatory funnel where the aqueous layer was extracted with DCM (3 x 75 mL). The combined organics were dried over sodium sulfate, filtered and concentrated yielding the title compound as a white solid (99.3 mg, 0.361 mmol, 72% yield).

^{1}H NMR (500 MHz, $CDCl_3$) δ 7.98 (d, J = 8.3 Hz, 2H), 7.45 (d, J = 8.3 Hz, 2H), 4.04 (dd, J = 11.8, 2.4 Hz, 1H), 3.90 (s, 3H), 1.53 (dt, J = 12.9, 2.2 Hz, 1H), 1.40 (dd, J = 13.8, 2.0 Hz, 1H), 1.28 (s, 3H), 1.28 – 1.20 (m, 2H), 1.16 (s, 3H), 1.15 (s, 3H), 0.94 (s, 3H).

^{13}C NMR (126 MHz, $CDCl_3$) δ 167.3, 151.2, 129.9, 128.8, 126.9, 52.9, 52.2, 51.1, 50.3, 48.0, 35.5, 35.3, 31.0, 27.3, 26.7.

HRMS (ESI-TOF) *m*/*z* calcd. for $C_{17}H_{26}NO_2^+$ ($[M+H]^+$) 276.1958, found 276.1957.

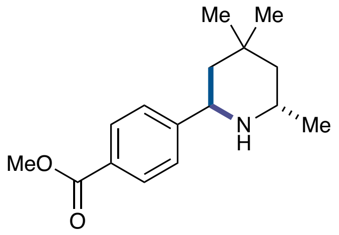

Methyl 4-((6*S*)-4,4,6-trimethylpiperidin-2-yl)benzoate (5)

Prepared according to **General Procedure A-2** with methyl (*S*,*E*)-4-(((4,4-dimethylpentan-2-yl)imino)methyl)benzoate (78.4 mg, 0.3 mmol), benzenesulfonic acid (189.8 mg, 1.2 mmol, 4 equiv), $FeCl_3$ (9.7 mg, 0.06 mmol, 20 mol%), bis(4-methoxyphenyl) disulfide (25.1 mg, 0.09 mmol, 30 mol%), and anhydrous MeCN (1.5 mL). The reaction was irradiated for 12 hrs under 100% light intensity, 365 nm plate.

After working up according to **General Procedure A-2**, the mixture was purified by purification ***method (2) acid/base wash***. With the basified, dry organic mixture, Et_2O (10 mL) was added to partially dissolve the mixture, followed by addition of water (10 mL). Then, concentrated HCl was directly added into the mixture. After mixing, the mixture was transferred to separatory funnel. The layer separated and the aqueous layer was collected. Additional water and concentrated HCl were added into the Et_2O layer to allow the second mixing and layer separation. The process was repeated 3 to 4 times. The collected aqueous layer was basified by saturated $NaHCO_3$ solution and 1M NaOH solution. After the pH of the solution was checked by pH indicators to be greater than 10, the aqueous layer was extracted by EtOAc (4 x 20 mL) to afford off-white solids (70.6 mg, 0.27 mmol, 91% yield, d.r > 10:1 [determined by NMR]).

^{1}H NMR (500 MHz, $CDCl_3$) δ 7.97 (d, *J* = 8.4 Hz, 2H), 7.44 (d, *J* = 8.2 Hz, 2H), [3.98 (dd, *J* = 12.3, 3.2 Hz), 3.92 (dd, *J* = 12.1, 2.9 Hz), 1H], 3.90 (s, 3H), 3.01 (dqd, *J* = 12.3, 6.1, 2.5 Hz, 1H), 1.62 – 1.52 (m, 1H), 1.47 (dt, *J* = 13.0, 2.4 Hz, 1H), 1.38 (dt, *J* = 13.1, 2.4 Hz, 1H), 1.30 (t, *J* = 12.4 Hz, 1H), 1.23 – 1.08 (m, 4H), 1.06 (s, 3H), 0.95 (s, 3H). **[reported mixture of diastereomers]**

^{13}C NMR (126 MHz, $CDCl_3$) δ δ 167.2, 150.9, 129.9, 128.9, 126.8, 57.7, 52.1, 48.3, 47.7, 47.2, 33.3, 31.0, 25.3, 23.2. **[reported mixture of diastereomers]**

HRMS (ESI-TOF) *m*/*z* calcd. for $C_{16}H_{24}NO_2^+$ ($[M+H]^+$) 262.1802, found 262.1804.

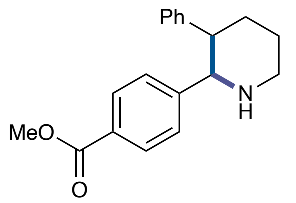

Methyl 4-(3-phenylpiperidin-2-yl)benzoate (6)

Prepared according to **General Procedure A-2** with methyl (*E*)-4-(((4-phenylbutyl)imino)methyl)benzoate (147.7 mg, 0.5 mmol), benzenesulfonic acid (316.4 mg, 2.0 mmol, 4 equiv), $FeCl_3$ (16.2 mg, 0.1 mmol, 20 mol%), bis(4-methoxyphenyl) disulfide (41.8 mg, 0.15 mmol, 30 mol%), and anhydrous MeCN (2.5 mL). The reaction was irradiated for 12 hrs under 100% light intensity, 365 nm plate.

After working up according to **General Procedure A-2**, the mixture was purified by purification method ***(1) Biotage® Sfär KP-Amino (KP-NH / KP-Amino) Flash Cartridges***. The basified, dry organic mixture was dissolved in minimal EtOAc and loaded on a 28 g Biotage® Sfär KP-Amino (KP-NH / KP-Amino) flash cartridges. It is purified with a slow gradient from 0% to 40% EtOAc in hexanes. Then the column was washed with 40%-100% EtOAc in hexanes. The resulting product fractions were directly concentrated down to afford off-white products (76.1 mg, 0.26 mmol, 52% yield, d.r. as determined by uHPLC area ratio analysis: d.r. = 4.8:1).

^{1}H NMR (500 MHz, $CDCl_3$) δ 7.78 (d, *J* = 8.3 Hz, 2H), 7.21 – 7.15 (m, 2H), 7.13 – 7.06 (m, 2H), 7.06 – 7.00 (m, 1H), 6.97 – 6.90 (m, 2H), 3.84 (s, 3H), 3.76 (d, *J* = 10.1 Hz, 1H), 3.31 – 3.13 (m, 1H), 2.92 (td, *J* = 11.3, 3.6 Hz, 1H), 2.74 (tdd, *J* = 9.8, 8.6, 3.6 Hz, 1H), 2.47 (s, 1H), 2.14 – 2.00 (m, 1H), 1.91 – 1.67 (m, 3H). **[reported major diastereomers]**

^{13}C NMR (126 MHz, $CDCl_3$) δ 167.2, 148.4, 143.2, 129.4, 128.9, 128.2, 128.0, 127.9, 126.3, 68.2, 52.1, 51.0, 47.6, 33.4, 26.6. **[reported major diastereomers]**

HRMS (ESI-TOF) *m*/*z* calcd. for $C_{19}H_{22}NO_2^+$ ($[M+H]^+$) 296.1645, found 296.1648.

Major diastereomer assignment based on ^{1}H NMR and COSY

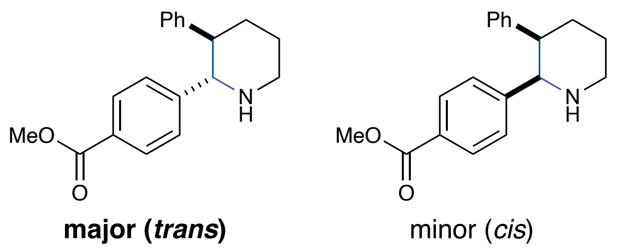

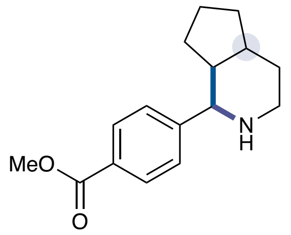

Methyl 4-(octahydro-1*H*-cyclopenta[*c*]pyridin-1-yl)benzoate (7)

Prepared according to **General Procedure A-2** with methyl (*E*)-4-(((2-cyclopentylethyl)imino)methyl)benzoate (129.7 mg, 0.5 mmol), benzenesulfonic acid (316.4 mg, 2.0 mmol, 4 equiv), $FeCl_3$ (16.2 mg, 0.1 mmol, 20 mol%), bis(4-methoxyphenyl) disulfide (41.8 mg, 0.15 mmol, 30 mol%), and anhydrous MeCN (2.5 mL). The reaction was irradiated for 12 hrs under 100% light intensity, 365 nm plate.

After working up according to **General Procedure A-2**, the mixture was purified by purification method ***(3) prepHPLC***. The basified, dry organic mixture was subjected to further purification by preparative reverse phase HPLC with 30% to 60% MeCN in water (0.1% NH_4OH modifier). The clean fractions of the major regioisomer were collected and concentrated down for product characterization. Due to the difficulty of isolating each regioisomer and diastereomer with highly similar polarity, based on the similarity of the chromatography absorbance of all the isomers, the yield is reported for all cyclized product as an assay yield based on chromatography from ultra performance liquid chromatography (uHPLC). Based on UV absorbance at 250 nm, the calibration curve has a slope of 3.9227 with R^2 = 0.9999. (average yields across two runs as determined by uHPLC analysis vs. 1,4-dinitrobenzene: 50% yield; d.r. as determined by uHPLC area ratio analysis: d.r. = 1.14:1 [*the yield only accounts for* ***isolatable and identifiable*** *products, please refer to spectral section for more details*]).

^{1}H NMR (500 MHz, $CDCl_3$) δ 7.98 (d, *J* = 8.0 Hz, 2H), 7.45 (d, *J* = 8.1 Hz, 2H), 3.90 (s, 3H), 3.39 (d, *J* = 10.7 Hz, 1H), 2.93 (d, *J* = 8.8 Hz, 2H), 2.46 – 1.87 (m, 4H), 1.85 – 1.50 (m, 5H), 1.50 – 1.35 (m, 1H), 1.26 (ddd, *J* = 12.9, 7.9, 3.3 Hz, 1H). **[reported major diastereomer of the major regioisomer, please refer to spectral section for more details]**

^{13}C NMR (126 MHz, $CDCl_3$) δ 167.1, 149.0, 129.8, 129.4, 128.3, 62.1, 52.2, 45.3, 42.4, 38.2, 28.3, 27.1, 22.3. **[reported major diastereomer of the major regioisomer]**

HRMS (ESI-TOF) *m*/*z* calcd. for $C_{16}H_{22}NO_2^+$ ([M+H]$^+$) 260.1645, found 260.1648.

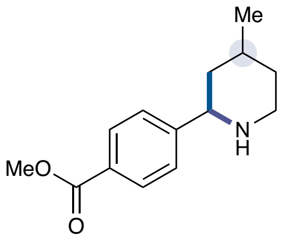

Methyl 4-(4-methylpiperidin-2-yl)benzoate (8)

Prepared according to **General Procedure A-2** with methyl (*E*)-4-((isopentylimino)methyl)benzoate (116.7 mg, 0.5 mmol), benzenesulfonic acid (316.4 mg, 2.0 mmol, 4 equiv), $FeCl_3$ (16.2 mg, 0.1 mmol, 20 mol%), **bis(4-methoxyphenyl) disulfide (83.5 mg, 0.3 mmol, 60 mol%)**, and **anhydrous MeCN (0.65 mL)**. The reaction was irradiated for 12 hrs under 100% light intensity, 365 nm plate. *For linear alkyl substrates, increasing disulfide loading and reaction concentration improves the regioselectivity towards six-member ring formation.*

After working up according to **General Procedure A-2**, the mixture was purified by purification method ***(3) prepHPLC***. The basified, dry organic mixture was subjected to further purification by preparative reverse phase HPLC with 30% to 60% MeCN in water (0.1% NH_4OH modifier). The clean fractions of the major regioisomer were collected and concentrated down for product characterization. Due to the difficulty of isolating each regioisomer and diastereomer with highly similar polarity, based on the similarity of the chromatography absorbance of all the isomers, the yield is reported for all cyclized product as an assay yield based on chromatography from ultra performance liquid chromatography (uHPLC). Based on UV absorbance at 195 nm, the calibration curve has a slope of 1.0387 with R^2 = 0.9992. (average yields across two runs as determined by uHPLC analysis vs. 1,4-dinitrobenzene: 76% yield; r.r as determined by uHPLC area ratio analysis: r.r (6-member : 5-member) = 9.7:1; d.r. between the six-member rings as determined by uHPLC area ratio analysis: d.r. = 1.7:1).

^{1}H NMR (500 MHz, $CDCl_3$) δ 7.98 (d, *J* = 8.2 Hz, 2H), 7.49 (d, *J* = 8.0 Hz, 2H), 3.97 – 3.92 (m, 1H), 3.91 (s, 3H), 3.75 (dd, *J* = 11.5, 2.6 Hz, 1H), 3.23 – 3.11 (m, 1H), 2.80 (td, *J* = 12.3, 2.6 Hz, 1H), 1.83 (dq, *J* = 13.1, 2.7 Hz, 1H), 1.75 – 1.56 (m, 2H), 1.39 – 1.21 (m, 2H), 0.98 (d, *J* = 6.3 Hz, 3H). **[reported major diastereomer of the major regioisomer, please refer to spectral section for more details]**

^{13}C NMR (126 MHz, $CDCl_3$) δ 167.0, 130.0, 129.57, 129.55, 127.1, 61.6, 52.2, 46.8, 42.3, 33.3, 31.7, 22.3. **[reported major diastereomer of the major regioisomer]**

HRMS (ESI-TOF) *m*/*z* calcd. for $C_{14}H_{20}NO_2^+$ ($[M+H]^+$) 234.1489, found 234.1486.

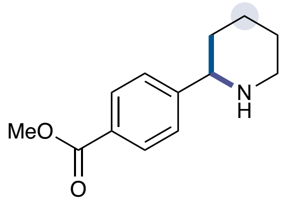

(±)-Methyl 4-(piperidin-2-yl)benzoate (9)

Prepared according to **General Procedure A-2** with methyl (*E*)-4-((butylimino)methyl)benzoate (109.6 mg, 0.5 mmol), benzenesulfonic acid (316.4 mg, 2.0 mmol, 4 equiv), $FeCl_3$ (16.2 mg, 0.1 mmol, 20 mol%), **bis(4-methoxyphenyl) disulfide (83.5 mg, 0.3 mmol, 60 mol%)**, and **anhydrous MeCN (0.65 mL**). The reaction was irradiated for 12 hrs under 100% light intensity, 365 nm plate. *For linear alkyl substrates, increasing disulfide loading and reaction concentration improve the regioselectivity towards six-member ring formation.*

After working up according to **General Procedure A-2**, the mixture was purified by purification method ***(3) prepHPLC***. The basified, dry organic mixture was subjected to further purification by preparative reverse phase HPLC with 30% to 60% MeCN in water (0.1% NH_4OH modifier). The clean fractions of the major regioisomer were collected and concentrated down for product characterization. Due to the difficulty of isolating each regioisomer and diastereomer with highly similar polarity, based on the similarity of the chromatography absorbance of all the isomers, the yield is reported for all cyclized product as an assay yield based on chromatography from ultra performance liquid chromatography (uHPLC). Based on UV absorbance at 195 nm, the calibration curve has a slope of 1.5704 with R^2 = 0.9998. (average yields across two runs as determined by uHPLC analysis vs. 1,4-dinitrobenzene: 86% yield; r.r as determined by uHPLC area ratio analysis: r.r (6-member : 5-member) = 3:1; d.r. between the five-member rings as determined by uHPLC area ratio analysis: d.r. = 1.9:1).

^{1}H NMR (500 MHz, $CDCl_3$) δ 7.98 (d, *J* = 8.0 Hz, 2H), 7.49 (d, *J* = 8.0 Hz, 2H), 3.99 – 3.91 (m, 1H), 3.90 (s, 3H), 3.75 (dd, *J* = 11.5, 2.7 Hz, 1H), 3.19 (d, *J* = 12.3 Hz, 1H), 2.82 – 2.75 (m, 1H), 1.93 – 1.79 (m, 2H), 1.72 – 1.63 (m, 3H), 1.53 (ddt, *J* = 17.0, 8.2, 3.8 Hz, 1H). **[reported major diastereomer of the major regioisomer, please refer to spectral section for more details]**

^{13}C NMR (126 MHz, $CDCl_3$) δ 167.0, 133.2, 130.1, 129.6, 127.1, 61.7, 52.2, 47.0, 33.6, 24.7, 24.6. **[reported major diastereomer of the major regioisomer]**

HRMS (ESI-TOF) *m*/*z* calcd. for $C_{13}H_{18}NO_2^+$ ($[M+H]^+$) 220.1332, found 220.1338.

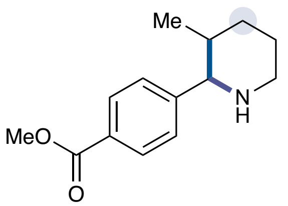

Methyl 4-(3-methylpiperidin-2-yl)benzoate (10)

Prepared according to **General Procedure A-2** with methyl (*E*)-4-((pentylimino)methyl)benzoate (116.7 mg, 0.5 mmol), benzenesulfonic acid (316.4 mg, 2.0 mmol, 4 equiv), $FeCl_3$ (16.2 mg, 0.1 mmol, 20 mol%), **bis(4-methoxyphenyl) disulfide (83.5 mg, 0.3 mmol, 60 mol%)**, and **anhydrous MeCN (0.65 mL)**. The reaction was irradiated for 12 hrs under 100% light intensity, 365 nm plate. *For linear alkyl substrates, increasing disulfide loading and reaction concentration improve the regioselectivity towards six-member ring formation.*

After working up according to **General Procedure A-2**, the mixture was purified by purification method ***(3) prepHPLC***. The basified, dry organic mixture was subjected to further purification by preparative reverse phase HPLC with 30% to 60% MeCN in water (0.1% NH_4OH modifier). The clean fractions of the major regioisomer were collected and concentrated down for product characterization. Due to the difficulty of isolating each regioisomer and diastereomer with highly similar polarity, based on the similarity of the chromatography absorbance of all the isomers, the yield is reported for all cyclized product as an assay yield based on chromatography from ultra performance liquid chromatography (uHPLC). Based on UV absorbance at 195 nm, the calibration curve has a slope of 0.9855 with R^2 = 0.9994. (average yields across two runs as determined by uHPLC analysis vs. 1,4-dinitrobenzene: 82% yield; r.r as determined by uHPLC area ratio analysis: r.r (6-member : 5- and 7-member) = 4.6:1; d.r. between the six-member rings as determined by uHPLC area ratio analysis: d.r. = 1.1:1).

^{1}H NMR (500 MHz, $CDCl_3$) δ 7.99 (d, *J* = 7.9 Hz, 2H), 7.48 (d, *J* = 8.0 Hz, 2H), 3.98 – 3.92 (m, 1H), 3.91 (s, 3H), 3.26 (d, *J* = 10.1 Hz, 1H), 3.10 (d, *J* = 12.0 Hz, 1H), 2.73 (td, *J* = 12.2, 2.7 Hz, 1H), 1.95 – 1.85 (m, 1H), 1.79 (s, 2H, br), 1.70 (d, *J* = 13.5 Hz, 1H), 1.32 – 1.08 (m, 1H), 0.62 (d, *J* = 6.6 Hz, 3H). **[reported major diastereomer of the major regioisomer, please refer to spectral section for more details]**

^{13}C NMR (126 MHz, $CDCl_3$) δ 167.0, 129.9, 129.8, 128.3, 126.6, 69.0, 52.3, 47.1, 36.9, 33.7, 25.4, 18.8. **[reported major diastereomer of the major regioisomer]**

HRMS (ESI-TOF) *m*/*z* calcd. for $C_{14}H_{20}NO_2^+$ ($[M+H]^+$) 234.1489, found 234.1489.

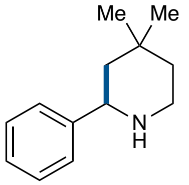

(±)-4,4-Dimethyl-2-phenylpiperidine (11)

Prepared according to **General Procedure A-1** with benzaldehyde (53.3 mg, 51.3 µL, 0.5 mmol, 1 eq, 99% purity) and 3,3-dimethyl-butylamine (58.6 mg, 77.9 µL, 0.55 mmol, 1.1 eq, 95% purity), benzenesulfonic acid (316.4 mg, 2.0 mmol, 4 equiv), $FeCl_3$ (16.2 mg, 0.1 mmol, 20 mol%), bis(4-methoxyphenyl) disulfide (41.8 mg, 0.15 mmol, 30 mol%) and anhydrous MeCN (2.5 mL). The reaction was irradiated for 12 hrs under 100% light intensity, 365 nm plate.

After working up according to **General Procedure A-1**, the mixture was purified by purification ***method (3) reverse phase chromatography***. The basified, dry organic mixture was loaded on 30 g reverse phase column with 5 to 20% MeCN in water, 0.1 % FA modifier. Pure fractions were combined, and the acetonitrile was removed under reduced pressure. The resulting solution was basified with 10 mL of saturated sodium bicarbonate, transferred to a separatory funnel where the aqueous layer was extracted with DCM (3 x 75 mL). The combined organics were dried over sodium sulfate, filtered and concentrated yielding the title compound as a slightly volatile light-yellow oil (50.6 mg, 0.27 mmol, 53% yield).

^{1}H NMR (500 MHz, $CDCl_3$) δ 7.39 (d, J = 6.9 Hz, 2H), 7.31 (t, J = 7.5 Hz, 2H), 7.24 (m, J = 7.4 Hz, 1H), 3.86 – 3.78 (m, 1H), 2.97 (d, J = 10.7 Hz, 2H), 1.59 – 1.45 (m, 2H), 1.36 (d, J = 13.4 Hz, 1H), 1.06 (s, 3H), 0.98 (s, 3H).

Spectral data are consistent with those reported in literature:
Healy, M. A. M.; Smith, S. A.; Stemp, G. *Synth Commn*, **1995**, *25*(23), 3789-3797.

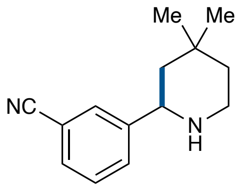

(±)-3-(4,4-Dimethylpiperidin-2-yl)benzonitrile (12)

Prepared according to **General Procedure A-1** with 3-formylbenzonitirle (66.9 mg, 0.5 mmol, 1 eq, 98% purity) and 3,3-dimethyl-butylamine (58.6 mg, 77.9 μL, 0.55 mmol, 1.1 eq, 95% purity), benzenesulfonic acid (316.4 mg, 2.0 mmol, 4 equiv), $FeCl_3$ (16.2 mg, 0.1 mmol, 20 mol%), bis(4-methoxyphenyl) disulfide (41.8 mg, 0.15 mmol, 30 mol%) and anhydrous MeCN (2.5 mL). The reaction was irradiated for 12 hrs under 100% light intensity, 365 nm plate.

After working up according to **General Procedure A-1**, the mixture was purified by purification ***method (3) reverse phase chromatography***. The basified, dry organic mixture was loaded on 30 g reverse phase column with 25 to 65% MeCN in water, 0.1 % NH_4OH modifier. Pure fractions were combined, and the acetonitrile was removed under reduced pressure. The resulting solution was further basified with 10 mL of saturated sodium bicarbonate, transferred to a separatory funnel where the aqueous layer was extracted with DCM (3 x 75 mL). The combined organics were dried over sodium sulfate, filtered and concentrated yielding the title compound as a light-yellow oil (68.5 mg, 0.304 mmol, 61% yield accounting for 95% purity).

^{1}H NMR (500 MHz, $CDCl_3$) δ 7.69 (d, *J* = 2.2 Hz, 1H), 7.61 (d, *J* = 7.8 Hz, 1H), 7.55 – 7.50 (m, 1H), 7.40 (t, *J* = 7.7 Hz, 1H), 3.84 (dd, *J* = 11.8, 2.7 Hz, 1H), 3.07 – 2.93 (m, 2H), 1.77 (s, 1H), 1.47 (tdd, *J* = 11.0, 7.5, 4.8 Hz, 2H), 1.41 – 1.36 (m, 1H), 1.33 (t, *J* = 12.4 Hz, 1H), 1.06 (s, 3H), 0.98 (s, 3H).

^{13}C NMR (126 MHz, $CDCl_3$) δ 147.6, 131.4, 130.9, 130.6, 129.3, 119.1, 112.6, 56.9, 48.1, 43.2, 38.4, 33.3, 30.4, 24.3.

HRMS (ESI-TOF) *m/z* calcd. for $C_{14}H_{19}N_2^+$ ($[M+H]^+$) 215.1543, found 215.1542.

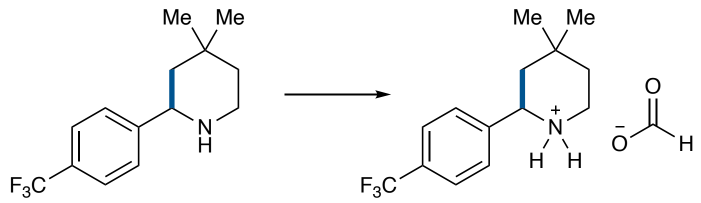

(±)-4,4-Dimethyl-2-(4-(trifluoromethyl)phenyl)piperidine (13)

Prepared according to **General Procedure A-1** with 4-trifluoromethylbenzaldehyde (97.0 mg, 0.5 mmol, 1 eq, 98% purity) and 3,3-dimethyl-butylamine (58.6 mg, 77.9 µL, 0.55 mmol, 1.1 eq, 95% purity), benzenesulfonic acid (316.4 mg, 2.0 mmol, 4 equiv), $FeCl_3$ (16.2 mg, 0.1 mmol, 20 mol%), bis(4-methoxyphenyl) disulfide (41.8 mg, 0.15 mmol, 30 mol%) and anhydrous MeCN (2.5 mL). The reaction was irradiated for 12 hrs under 100% light intensity, 365 nm plate.

After working up according to **General Procedure A-1**, the mixture was purified by purification ***method (3) reverse phase chromatography***. The basified, dry organic mixture was loaded on 30 g reverse phase column with 30 to 80% MeCN in water, 0.1 % NH_4OH modifier). Pure fractions were combined and acidified with 0.5 mL of FA then concentrated yielding the title compound as an off-white solid after extensive drying (125.6 mg, 0.406 mmol, 81% yield accounting for 98% purity).

^{1}H NMR (500 MHz, MeOD) δ 8.48 (s, 1H, formate peak), 7.79 (d, J = 8.1 Hz, 2H), 7.69 (d, J = 8.0 Hz, 2H), 4.51 (dd, J = 12.8, 3.4 Hz, 1H), 3.42 – 3.35 (m, 2H), 1.93 – 1.79 (m, 2H), 1.79 – 1.66 (m, 2H), 1.23 (s, 3H), 1.14 (s, 3H). The hydrogens at $^+NH_2$ was not detected by the NMR due to the use of MeOD.

^{13}C NMR (126 MHz, MeOD) δ 169.5 (formate peak), 142.8, 132.4 (q, J = 32.6 Hz), 129.1, 127.2 (q, J = 3.8 Hz), 125.3 (q, J = 271.4 Hz), 57.4, 44.1, 43.1, 35.8, 32.4, 30.6, 23.8.

^{19}F NMR (282 MHz, MeOD) δ -64.35.

HRMS (ESI-TOF) *m/z* calcd. for $C_{14}H_{19}F_3N^+$ ($[M+H]^+$) 258.1464, found 258.1468.

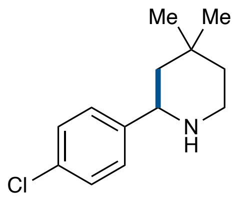

(±)-2-(4-Chlorophenyl)-4,4-dimethylpiperidine (14)

Prepared according to **General Procedure A-1** with 4-chlorobenzaldehyde (71.7 mg, 0.5 mmol, 1 eq, 98% purity) and 3,3-dimethyl-butylamine (58.6 mg, 77.9 µL, 0.55 mmol, 1.1 eq, 95% purity), benzenesulfonic acid (316.4 mg, 2.0 mmol, 4 equiv), $FeCl_3$ (16.2 mg, 0.1 mmol, 20 mol%), bis(4-methoxyphenyl) disulfide (41.8 mg, 0.15 mmol, 30 mol%) and anhydrous MeCN (2.5 mL). The reaction was irradiated for 12 hrs under 100% light intensity, 365 nm plate.

After working up according to **General Procedure A-1**, the mixture was purified by purification ***method (3) reverse phase chromatography***. The basified, dry organic mixture was loaded on 30 g reverse phase column with 25 to 65% MeCN in water, 0.1 % NH_4OH modifier). Pure fractions were combined, and the acetonitrile was removed under reduced pressure. The resulting solution was further basified with 10 mL of saturated sodium bicarbonate, transferred to a separatory funnel where the aqueous layer was extracted with DCM (3 x 75 mL). The combined organics were dried over sodium sulfate, filtered and concentrated yielding the title compound as a light-yellow solid (95.2 mg, 0.425 mmol, 85% yield).

^{1}H NMR (400 MHz, MeOD) δ 7.48 (s, 4H), 4.41 (dd, J = 12.8, 3.2 Hz, 1H), 3.37 – 3.32 (m, 2H), 1.88 (dd, J = 14.4, 12.7 Hz, 1H), 1.81 – 1.64 (m, 3H), 1.21 (s, 3H), 1.12 (s, 3H).

^{13}C NMR (101 MHz, MeOD) δ 168.3, 136.9, 136.5, 130.5, 130.1, 57.2, 43.8, 43.0, 35.6, 32.4, 30.5, 23.8.

HRMS (ESI-TOF) *m/z* calcd. for $C_{13}H_{19}ClN^+$ ($[M+H]^+$) 224.1201, found 224.1205.

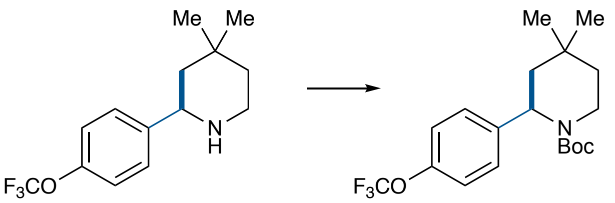

(±)-Tert-butyl 4,4-dimethyl-2-(4-(trifluoromethoxy)phenyl)piperidine-1-carboxylate (15)

Prepared according to **General Procedure A-1** with 4-(tirfluoromethoxy)benzaldehyde (97.0 mg, 0.5 mmol, 1 eq, 98% purity) and 3,3-dimethyl-butylamine (58.6 mg, 77.9 µL, 0.55 mmol, 1.1 eq, 95% purity), benzenesulfonic acid (316.4 mg, 2.0 mmol, 4 equiv), $FeCl_3$ (16.2 mg, 0.1 mmol, 20 mol%), bis(4-methoxyphenyl) disulfide (41.8 mg, 0.15 mmol, 30 mol%) and anhydrous MeCN (2.5 mL). The reaction was irradiated for 24 hrs under 100% light intensity, 365 nm plate.

Due to the isolation difficulty, the product was isolated after Boc protection. After working up according to **General Procedure A-1**, the mixture is redissolved in DCM. The mixture in DCM then was added sodium sulfate, di-*tert*-butyl dicarbonate (218.3 mg, 1.0 mmol, 2 eq), TEA (151.8 mg, 209 µL, 1.5 mmol, 3 eq.) and DMAP (6.1 mg, 0.05 mmol, 10 mol%). The resulting solution was stirred overnight then filtered and concentrated under reduced pressure. The crude residue was purified by preparative HPLC (70 to 100% MeCN in water, 0.1 % NH_4OH modifier) yielding the title compound as a clear oil (148.6 mg, 0.398 mmol, 80% yield).

^{1}H NMR (500 MHz, $CDCl_3$) δ 7.19 (d, J = 8.7 Hz, 2H), 7.15 (d, J = 8.5 Hz, 2H), 5.16 (t, J = 5.9 Hz, 1H), 4.06 (ddd, J = 13.8, 5.2, 3.1 Hz, 1H), 3.12 (ddd, J = 13.8, 12.2, 3.6 Hz, 1H), 1.92 (ddd, J = 14.1, 5.3, 1.4 Hz, 1H), 1.76 (dd, J = 14.1, 6.4 Hz, 1H), 1.62 – 1.42 (m, 2H), 1.37 (s, 9H), 0.98 (s, 3H), 0.64 (s, 3H).

^{13}C NMR (126 MHz, $CDCl_3$) δ 155.7, 147.7 (q, J = 2.2 Hz), 142.1, 126.8, 121.0, 120.6 (q, J = 256.7 Hz), 80.0, 53.1, 42.6, 37.9, 37.8, 31.9, 29.1, 28.5, 27.7.

^{19}F NMR (471 MHz, $CDCl_3$) δ -57.93.

HRMS (ESI-TOF)* *m/z* calcd. for $C_{19}H_{27}F_3NO_3^+$ ([M+H]$^+$) 374.1938, found 274.1418 and 318.1318.

**Note: this HRMS result indicates the desired product, following in situ tert-butyl carbamate decomposition to the corresponding carbamic acid under acidic and the corresponding piperidine ESI-TOF conditions. For carbamic acid analogue: m/z* calcd for $C_{15}H_{19}F_3NO_3^+$ ([M+H]$^+$) 318.1312. *For piperidine analogue: m/z* calcd for $C_{14}H_{19}F_3NO^+$ ([M+H]$^+$) 274.1413.

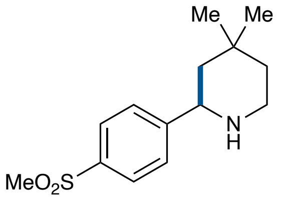

(±)-4,4-Dimethyl-2-(4-(methylsulfonyl)phenyl)piperidine (16)

Prepared according to **General Procedure A-2** with (*E*)-*N*-(3,3-dimethylbutyl)-1-(4-(methylsulfonyl)phenyl)methanimine (133.7 mg, 0.5 mmol), benzenesulfonic acid (316.4 mg, 2.0 mmol, 4 equiv), $FeCl_3$ (16.2 mg, 0.1 mmol, 20 mol%), bis(4-methoxyphenyl) disulfide (41.8 mg, 0.15 mmol, 30 mol%), and anhydrous MeCN (2.5 mL). The reaction was irradiated for 8 hrs under 100% light intensity, 365 nm plate.

After working up according to **General Procedure A-2**, the mixture was purified by purification ***method (2) acid/base wash***. With the basified, dry organic mixture, Et_2O (10 mL) was added to partially dissolve the mixture, followed by addition of water (10 mL). Then, concentrated HCl was directly added into the mixture. After mixing, the mixture was transferred to separatory funnel. The layer separated and the aqueous layer was collected. Additional water and concentrated HCl were added into the Et_2O layer to allow the second mixing and layer separation. The process was repeated 3 to 4 times. The collected aqueous layer was basified by saturated $NaHCO_3$ solution and 1M NaOH solution. After the pH of the solution was checked by pH indicators to be greater than 10. The aqueous layer was extracted by EtOAc (4 x 20 mL) to afford light yellow oil (87.5 mg, 0.33 mmol, 65% yield).

^{1}H NMR (500 MHz, $CDCl_3$) δ 7.87 (d, *J* = 8.4 Hz, 2H), 7.57 (d, *J* = 8.4 Hz, 2H), 3.89 (dd, *J* = 11.8, 2.7 Hz, 1H), 3.07 – 3.02 (m, 1H), 3.03 (s, 3H), 2.98 (td, *J* = 12.1, 2.9 Hz, 1H), 1.68 (s, 1H), 1.55 – 1.43 (m, 2H), 1.39 (dq, *J* = 13.3, 2.6 Hz, 1H), 1.31 (dd, *J* = 13.2, 11.6 Hz, 1H), 1.07 (s, 3H), 0.97 (s, 3H).

^{13}C NMR (126 MHz, $CDCl_3$) δ 152.3, 139.1, 127.7, 127.6, 57.2, 48.4, 44.7, 43.2, 38.6, 33.3, 30.4, 24.4.

HRMS (ESI-TOF) *m*/*z* calcd. for $C_{14}H_{22}NO_2S^+$ ($[M+H]^+$) 268.1366, found 268.1366.

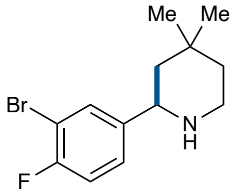

(±)-2-(3-Bromo-4-fluorophenyl)-4,4-dimethylpiperidine (17)

Prepared according to **General Procedure A-1** with 3-bromo-4-fluoro-benzaldehyde (103.6 mg, 0.5 mmol, 98% purity), 3,3-dimethylbutan-1-amine (78 µL, 0.55 mol, 1.1 equiv, 95% purity), benzenesulfonic acid (316.4 mg, 2.0 mmol, 4 equiv), $FeCl_3$ (16.2 mg, 0.1 mmol, 20 mol%), bis(4-methoxyphenyl) disulfide (41.8 mg, 0.15 mmol, 30 mol%), and anhydrous MeCN (2.5 mL). The reaction was irradiated for 36 hrs under 100% light intensity, 365 nm plate.

After working up according to **General Procedure A-1**, the mixture was purified by purification ***method (2) acid/base wash***. With the basified, dry organic mixture, Et_2O (10 mL) was added to partially dissolve the mixture, followed by addition of water (10 mL). Then, concentrated HCl was directly added into the mixture. After mixing, the mixture was transferred to separatory funnel. The layer separated and the aqueous layer was collected. Additional water and concentrated HCl were added into the Et_2O layer to allow the second mixing and layer separation. The process was repeated 3 to 4 times. The collected aqueous layer was basified by saturated $NaHCO_3$ solution and 1M NaOH solution. After the pH of the solution was checked by pH indicators to be greater than 10. The aqueous layer was extracted by EtOAc (4 x 20 mL) to afford light yellow oil (84.2 mg, 0.29 mmol, 59% yield).

^{1}H NMR (500 MHz, $CDCl_3$) δ 7.58 (dd, *J* = 6.8, 2.2 Hz, 1H), 7.26 – 7.21 (m, 1H), 7.04 (t, *J* = 8.4 Hz, 1H), 3.74 (dd, *J* = 11.6, 2.7 Hz, 1H), 3.04 – 2.91 (m, 2H), 1.62 (s, 1H), 1.49 – 1.39 (m, 2H), 1.36 (dq, *J* = 13.5, 2.7 Hz, 1H), 1.29 (t, *J* = 12.4 Hz, 1H), 1.04 (s, 3H), 0.96 (s, 3H).

^{13}C NMR (126 MHz, $CDCl_3$) δ 158.1 (d, *J* = 246.1 Hz), 143.2 (d, *J* = 3.6 Hz), 131.8, 127.2 (d, *J* = 7.0 Hz), 116.3 (d, *J* = 22.1 Hz), 108.9 (d, *J* = 20.8 Hz), 56.5, 48.4, 43.3, 38.6, 33.4, 30.4, 24.4.

^{19}F NMR (471 MHz, $CDCl_3$) δ -110.3 (td, *J* = 7.8, 5.0 Hz).

HRMS (ESI-TOF) *m/z* calcd. for $C_{13}H_{18}BrFN^+$ ($[M+H]^+$) 286.0601, found 286.0603.

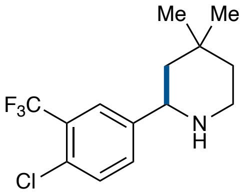

(±)-2-(4-Chloro-3-(trifluoromethyl)phenyl)-4,4-dimethylpiperidine (18)

Prepared according to **General Procedure A-1** with 4-chloro-3-(trifluoromethyl)benzaldehyde (109.8, 0.5 mmol, 95% purity), 3,3-dimethylbutan-1-amine (78 µL, 0.55 mol, 1.1 equiv, 95% purity), benzenesulfonic acid (316.4 mg, 2.0 mmol, 4 equiv), $FeCl_3$ (16.2 mg, 0.1 mmol, 20 mol%), bis(4-methoxyphenyl) disulfide (41.8 mg, 0.15 mmol, 30 mol%), and anhydrous MeCN (2.5 mL). The reaction was irradiated for 24 hrs under 100% light intensity, 365 nm plate.

After working up according to **General Procedure A-1**, the mixture was purified by purification ***method (2) acid/base wash***. With the basified, dry organic mixture, Et_2O (10 mL) was added to partially dissolve the mixture, followed by addition of water (10 mL). Then, concentrated HCl was directly added into the mixture. After mixing, the mixture was transferred to separatory funnel. The layer separated and the aqueous layer was collected. Additional water and concentrated HCl were added into the Et_2O layer to allow the second mixing and layer separation. The process was repeated 3 to 4 times. The collected aqueous layer was basified by saturated $NaHCO_3$ solution and 1M NaOH solution. After the pH of the solution was checked by pH indicators to be greater than 10. The aqueous layer was extracted by EtOAc (4 x 20 mL) to afford light yellow oil (103.7 mg, 0.36 mmol, 71% yield).

^{1}H NMR (500 MHz, MeOD) δ 7.81 (d, J = 2.0 Hz, 1H), 7.64 – 7.52 (m, 2H), 3.91 (dd, J = 12.1, 2.7 Hz, 1H), 3.05 – 2.91 (m, 2H), 1.54 (dt, J = 13.0, 2.4 Hz, 1H), 1.52 – 1.47 (m, 1H), 1.45 – 1.40 (m, 1H), 1.39 – 1.35 (m, 1H), 1.13 (s, 3H), 1.02 (s, 3H). The hydrogen at NH was not detected by the NMR due to the use of MeOD.

^{13}C NMR (126 MHz, MeOD) δ 145.7, 133.0, 132.7, 131.4 (d, J = 2.0 Hz), 129.2 (q, J = 31.0 Hz), 127.1 (q, J = 5.3 Hz), 124.5 (q, J = 273.4 Hz), 57.5, 48.3, 43.8, 39.0, 33.6, 31.1, 24.5.

^{19}F NMR (471 MHz, MeOD) δ -63.78.

HRMS (ESI-TOF) m/z calcd. for $C_{14}H_{18}ClF_3N^+$ ($[M+H]^+$) 292.1074, found 292.1071.

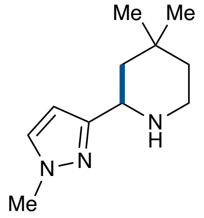

(±)-4,4-Dimethyl-2-(1-methyl-1*H*-pyrazol-3-yl)piperidine (19)

Prepared according to **General Procedure A-2** with (*E*)-*N*-(3,3-dimethylbutyl)-1-(1-methyl-1*H*-pyrazol-3-yl)methanimine (96.6 mg, 0.5 mmol), benzenesulfonic acid (316.4 mg, 2.0 mmol, 4 equiv), $FeCl_3$ (16.2 mg, 0.1 mmol, 20 mol%), bis(4-methoxyphenyl) disulfide (41.8 mg, 0.15 mmol, 30 mol%), and anhydrous MeCN (2.5 mL). The reaction was irradiated for 12 hrs under 100% light intensity, 365 nm plate.

After working up according to **General Procedure A-2**, the mixture was purified by purification ***method (3) prepHPLC (basic mode)***. The basified, dry organic mixture was subjected to further purification by preparative reverse phase HPLC with 20% to 50% MeCN in water (0.1% NH_4OH modifier). Product containing fractions were concentrated to afford desired product as an off-white solid (52 mg, 0.27 mmol, 54% yield).

^{1}H NMR (500 MHz, $CDCl_3$) δ 7.23 (d, *J* = 2.2 Hz, 1H), 6.17 (d, *J* = 2.3 Hz, 1H), 3.92 (dd, *J* = 12.1, 2.8 Hz, 1H), 3.83 (s, 3H), 3.10 – 2.78 (m, 2H), 2.24 (s, 1H), 1.60 (dt, *J* = 13.1, 2.6 Hz, 1H), 1.54 – 1.37 (m, 2H), 1.33 – 1.23 (m, 1H), 1.02 (s, 3H), 0.96 (s, 3H).

^{13}C NMR (126 MHz, $CDCl_3$) δ 155.2, 130.7, 103.0, 51.2, 45.5, 42.8, 38.8, 38.4, 33.2, 29.9, 24.3.

HRMS (ESI-TOF) *m*/*z* calcd. for $C_{11}H_{20}N_3^+$ ($[M+H]^+$) 194.1652, found 194.1655.

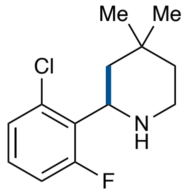

(±)-2-(2-Chloro-6-fluorophenyl)-4,4-dimethylpiperidine (20)

Prepared according to **General Procedure A-2** with (*E*)-1-(2-chloro-6-fluorophenyl)-*N*-(3,3-dimethylbutyl)methanimine (120.9 mg, 0.5 mmol), benzenesulfonic acid (316.4 mg, 2.0 mmol, 4 equiv), $FeCl_3$ (16.2 mg, 0.1 mmol, 20 mol%), bis(4-methoxyphenyl) disulfide (41.8 mg, 0.15 mmol, 30 mol%), and anhydrous MeCN (2.5 mL). The reaction was irradiated for 12 hrs under 100% light intensity, 365 nm plate.

After working up according to **General Procedure A-1**, the mixture was purified by purification ***method (2) acid/base wash***. With the basified, dry organic mixture, Et_2O (10 mL) was added to partially dissolve the mixture, followed by addition of water (10 mL). Then, concentrated HCl was directly added into the mixture. After mixing, the mixture was transferred to separatory funnel. The layer separated and the aqueous layer was collected. Additional water and concentrated HCl were added into the Et_2O layer to allow the second mixing and layer separation. The process was repeated 3 to 4 times. The collected aqueous layer was basified by saturated $NaHCO_3$ solution and 1M NaOH solution. After the pH of the solution was checked by pH indicators to be greater than 10. The aqueous layer was extracted by EtOAc (4 x 20 mL) to afford light yellow oil (86 mg, 0.36 mmol, 72% yield).

^{1}H NMR (500 MHz, $CDCl_3$) δ 7.19 – 7.06 (m, 2H), 7.03 – 6.78 (m, 1H), 4.44 (dd, *J* = 12.2, 2.7 Hz, 1H), 3.08 (ddd, *J* = 13.6, 4.6, 2.3 Hz, 1H), 2.91 (tt, *J* = 13.8, 3.5 Hz, 1H), 2.15 (s, 1H), 1.63 (t, *J* = 12.7 Hz, 1H), 1.45 (dt, *J* = 13.2, 2.4 Hz, 1H), 1.42 – 1.30 (m, 2H), 1.10 (s, 3H), 0.97 (s, 3H).

^{13}C NMR (126 MHz, $CDCl_3$) δ 162.2 (d, *J* = 248.3 Hz), 134.0 (d, *J* = 8.3 Hz), 128.7 (d, *J* = 11.8 Hz), 128.6 (d, *J* = 10.2 Hz), 125.7 (d, *J* = 3.1 Hz), 115.1 (d, *J* = 24.0 Hz), 53.3, 43.7, 43.6, 39.4, 33.3, 30.3, 23.9.

^{19}F NMR (471 MHz, $CDCl_3$) δ -111.76.

HRMS (ESI-TOF) *m*/*z* calcd. for $C_{13}H_{18}ClFN^+$ ($[M+H]^+$) 242.1106, found 242.1102.

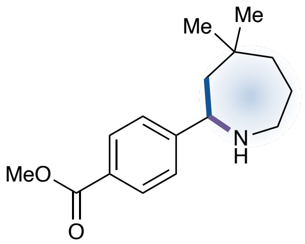

Methyl 4-(4,4-dimethylazepan-2-yl)benzoate (21)

Prepared according to **General Procedure A-1** with methyl 4-formylbenzoate (86.4 mg, 0.5 mmol, 95% purity), 4,4-dimethylpentan-1-amine (66.7 mg, 0.55 mmol, 95% purity), benzenesulfonic acid (316.4 mg, 2.0 mmol, 4 equiv), $FeCl_3$ (16.2 mg, 0.1 mmol, 20 mol%), bis(4-methoxyphenyl) disulfide (41.8 mg, 0.15 mmol, 30 mol%), and anhydrous MeCN (2.5 mL). The reaction was irradiated for 12 hrs under 100% light intensity, 365 nm plate.

After working up according to **General Procedure A-1**, the basified, dry organic mixture was subjected to further purification by preparative reverse phase HPLC with 45% to 55% MeCN in water (0.1% NH_4OH modifier). Product containing fractions were concentrated to afford desired product as an pale yellow oil (63 mg, 0.24 mmol, 48% yield, r.r = 95:5 [determined by NMR, 5% of five-member cyclized product was observed]).

^{1}H NMR (500 MHz, CDCl$_3$) δ 7.96 (d, J = 8.0 Hz, 2H), 7.37 (d, J = 8.0 Hz, 2H), 3.89 (s, 3H), 3.83 (d, J = 10.0 Hz, 1H), 3.17 (dt, J = 13.0, 5.1 Hz, 1H), 2.95 – 2.86 (m, 1H), 2.06 – 1.93 (m, 1H), 1.80 (dd, J = 14.5, 10.2 Hz, 1H), 1.69 (dtt, J = 9.1, 6.1, 2.7 Hz, 2H), 1.65 – 1.49 (m, 3H), 1.05 (s, 3H), 0.95 (s, 3H). **[reported major regioisomer, trace amount of minor regioisomer was observed]**

^{13}C NMR (126 MHz, CDCl$_3$) δ 167.14, 152.83, 130.05, 128.61, 126.26, 59.37, 53.33, 52.13, 49.85, 41.23, 33.51, 32.73, 28.63, 25.99. **[reported major regioisomer, trace amount of minor regioisomer was observed]**

HRMS (ESI-TOF) *m*/*z* calcd. for $C_{14}H_{24}NO_2^+$ ([M+H]$^+$) 262.1802, found 262.1801.

11) (Thio)morpholine scope via radical sampling

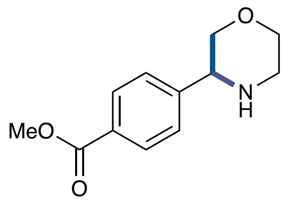

(±)-Methyl 4-(morpholin-3-yl)benzoate (22)

Prepared according to **General Procedure B-2** with methyl (*E*)-4-(((2-methoxyethyl)imino)methyl)benzoate (110.6 mg, 0.5 mmol), sodium persulfate (119.1 mg, 0.5 mmol, 1 equiv), benzenesulfonic acid (355.9 mg, 2.25 mmol, 4.5 equiv), $FeCl_3$ (32.4 mg, 0.2 mmol, 40 mol%), bis(4-methoxyphenyl) disulfide (27.8 mg, 0.1 mmol, 20 mol%), and anhydrous MeCN (3.5 mL). The reaction was irradiated for 20 hrs under 100% light intensity, 420 nm plate.

After working up according to **General Procedure B-2**, the mixture was purified by purification ***method (2) acid/base wash***. With the basified, dry organic mixture, Et_2O (10 mL) was added to partially dissolve the mixture, followed by addition of water (10 mL). Then, concentrated HCl was directly added into the mixture. After mixing, the mixture was transferred to separatory funnel. The layer separated and the aqueous layer was collected. Additional water and concentrated HCl were added into the Et_2O layer to allow the second mixing and layer separation. The process was repeated 3 to 4 times. The collected aqueous layer was basified by saturated $NaHCO_3$ solution and 1M NaOH solution. After the pH of the solution was checked by pH indicators to be greater than 10. The aqueous layer was extracted by EtOAc (4 x 20 mL) to afford light yellow oil (63 mg, 0.28 mmol, 57% yield).

^{1}H NMR (500 MHz, Acetone) δ 7.96 (d, *J* = 8.4 Hz, 2H), 7.57 (d, *J* = 8.2 Hz, 2H), 3.94 (dd, *J* = 10.0, 3.2 Hz, 1H), 3.87 (s, 3H), 3.77 (dt, *J* = 11.2, 2.3 Hz, 1H), 3.73 (dd, *J* = 10.8, 3.2 Hz, 1H), 3.53 (td, *J* = 11.1, 2.8 Hz, 1H), 3.22 (t, *J* = 10.4 Hz, 1H), 3.02 (td, *J* = 11.5, 3.2 Hz, 1H), 2.98 – 2.92 (m, 1H), 2.82 (br s, NH).

^{13}C NMR (126 MHz, Acetone) δ 167.1, 147.6, 130.23, 130.15, 128.2, 74.0, 67.8, 60.9, 52.3, 47.0.

Spectral data are consistent with those reported in literature:
Luescher, M. U.; Vo, C. V. T.; Bode, J. W. *Org. Lett.* **2014**, *16*(4), 1236-1239.

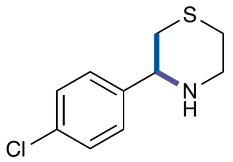

(±)-3-(4-Chlorophenyl)thiomorpholine (23)

Prepared according to **General Procedure B-2** with (*E*)-1-(4-chlorophenyl)-*N*-(2-(methylthio)ethyl)methanimine (107.9 mg, 0.5 mmol, 99% purity), **no oxidant (sodium persulfate) added**, benzenesulfonic acid (355.9 mg, 2.25 mmol, 4.5 equiv), $FeCl_3$ (32.4 mg, 0.2 mmol, 40 mol%), bis(4-methoxyphenyl) disulfide (27.8 mg, 0.1 mmol, 20 mol%), and anhydrous MeCN (3.5 mL). The reaction was irradiated for 20 hrs under 100% light intensity, 420 nm plate.

After working up according to **General Procedure B-2**, the mixture was purified by purification ***method (2) acid/base wash***. With the basified, dry organic mixture, Et_2O (10 mL) was added to partially dissolve the mixture, followed by addition of water (10 mL). Then, concentrated HCl was directly added into the mixture. After mixing, the mixture was transferred to separatory funnel. The layer separated and the aqueous layer was collected. Additional water and concentrated HCl were added into the Et_2O layer to allow the second mixing and layer separation. The process was repeated 3 to 4 times. The collected aqueous layer was basified by saturated $NaHCO_3$ solution and 1M NaOH solution. After the pH of the solution was checked by pH indicators to be greater than 10. The aqueous layer was extracted by EtOAc (4 x 20 mL) to afford light yellow oil (67.9 mg, 0.32 mmol, 64% yield).

^{1}H NMR (500 MHz, $CDCl_3$) δ 7.30 (s, 4H), 3.91 (dd, *J* = 10.6, 2.3 Hz, 1H), 3.44 (dt, *J* = 12.1, 3.1 Hz, 1H), 3.16 (td, *J* = 11.9, 2.3 Hz, 1H), 2.89 (ddd, *J* = 13.3, 11.7, 3.0 Hz, 1H), 2.78 (dd, *J* = 13.2, 10.5 Hz, 1H), 2.51 – 2.32 (m, 2H), 1.75 (s, 1H).

^{13}C NMR (126 MHz, $CDCl_3$) δ 142.8, 133.5, 128.9, 128.1, 62.4, 49.2, 35.0, 27.6.

HRMS (ESI-TOF) *m/z* calcd. for $C_{10}H_{13}ClNS^+$ ($[M+H]^+$) 214.0452, found 214.0457.

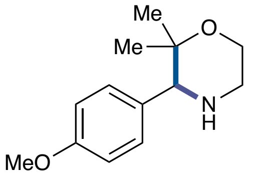

(±)-3-(4-Methoxyphenyl)-2,2-dimethylmorpholine (24)

Prepared according to **General Procedure C-2** with (*E*)-*N*-(2-isopropoxyethyl)-1-(4-methoxyphenyl)methanimine (66.4 mg, 0.3 mmol), trifluoroacetic acid (102.6 mg, 68.9 µL, 0.9 mmol, 3 equiv), TBADT (9.9 mg, 0.003 mmol, 1.0 mol%), bis(4-methoxyphenyl) disulfide (8.4 mg, 0.03 mmol, 10 mol%), $TBABF_4$ (98.8 mg, 0.3 mmol, 1 equiv) and anhydrous MeCN (1.5 mL). The reaction was irradiated for 24 hrs under 100% light intensity, 365 nm plate.

After working up according to **General Procedure C-2**, the basified, dry organic mixture was subjected to further purification by preparative reverse phase HPLC with 35% to 50% MeCN in water (0.1% NH_4OH modifier). Product containing fractions were concentrated to afford desired product as an off-white solid (33.7 mg, 0.15 mmol, 51% yield).

^{1}H NMR (500 MHz, $CDCl_3$) δ 7.29 (d, J = 8.7 Hz, 2H), 6.82 (d, J = 8.7 Hz, 2H), 3.97 (td, J = 11.6, 3.0 Hz, 1H), 3.79 (s, 3H), 3.68 – 3.60 (m, 2H), 3.07 (td, J = 11.6, 3.6 Hz, 1H), 3.01 – 2.94 (m, 1H), 1.63 (s, 1H), 1.20 (s, 3H), 1.08 (s, 3H).

NMR (126 MHz, $CDCl_3$) δ 159.1, 133.6, 129.5, 113.3, 75.3, 69.5, 61.8, 55.4, 48.0, 27.7, 17.4.

HRMS (ESI-TOF) *m*/*z* calcd. for $C_{13}H_{20}NO_2^+$ ($[M+H]^+$) 222.1489, found 222.14494.

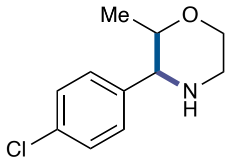

3-(4-Chlorophenyl)-2-methylmorpholine (25)

Prepared according to **General Procedure B-1** with 4-chlorobenzaldehyde (72.5 mg, 0.5 mmol, 97% purity), 2-ethoxyethan-1-amine (44.6 mg, 0.5 mmol, 1 equiv), sodium persulfate (119.1 mg, 0.5 mmol, 1 equiv), benzenesulfonic acid (355.9 mg, 2.25 mmol, 4.5 equiv), $FeCl_3$ (32.4 mg, 0.2 mmol, 40 mol%), bis(4-methoxyphenyl) disulfide (27.8 mg, 0.1 mmol, 20 mol%), and anhydrous MeCN (3.5 mL). The reaction was irradiated for 20 hrs under 100% light intensity, 420 nm plate.

After working up according to **General Procedure B-1**, the mixture was purified by purification ***method (2) acid/base wash***. With the basified, dry organic mixture, Et_2O (10 mL) was added to partially dissolve the mixture, followed by addition of water (10 mL). Then, concentrated HCl was directly added into the mixture. After mixing, the mixture was transferred to separatory funnel. The layer separated and the aqueous layer was collected. Additional water and concentrated HCl were added into the Et_2O layer to allow the second mixing and layer separation. The process was repeated 3 to 4 times. The collected aqueous layer was basified by saturated $NaHCO_3$ solution and 1M NaOH solution. After the pH of the solution was checked by pH indicators to be greater than 10. The aqueous layer was extracted by EtOAc (4 x 20 mL) to afford light yellow oil (52.7 mg, 0.25 mmol, 50% yield, d.r. as determined by uHPLC area ratio analysis: d.r. = 7.5:1).

^{1}H NMR (500 MHz, CD_3CN) δ 7.34 (q, J = 8.3 Hz, 4H), 3.97 – 3.75 (m, 1H), 3.68 – 3.55 (m, 1H), 3.50 – 3.19 (m, 2H), 2.93 (tdd, J = 11.5, 3.3, 1.1 Hz, 1H), 2.87 – 2.72 (m, 1H), 2.23 (s, 1H), 0.98 – 0.24 (m, 3H). **[reported major diastereomers]**

^{13}C NMR (101 MHz, CD_3CN) δ 142.0, 133.5, 130.8, 129.2, 78.3, 68.3, 67.5, 47.2, 18.5. **[reported major diastereomers]**

HRMS (ESI-TOF) *m/z* calcd. for $C_{11}H_{15}ClNO^+$ ($[M+H]^+$) 212.0837, found 212.0839.

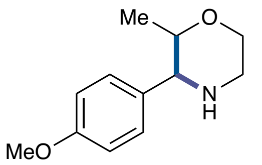

3-(4-methoxyphenyl)-2-methylmorpholine (26)

Prepared according to **General Procedure C-2** with (*E*)-*N*-(2-ethoxyethyl)-1-(4-methoxyphenyl)methanimine (60.4 mg, 0.3 mmol), trifluoroacetic acid (102.6 mg, 68.9 µL, 0.9 mmol, 3 equiv), TBADT (9.9 mg, 0.003 mmol, 1.0 mol%), bis(4-methoxyphenyl) disulfide (8.4 mg, 0.03 mmol, 10 mol%), $TBABF_4$ (98.8 mg, 0.3 mmol, 1 equiv) and anhydrous MeCN (1.5 mL). The reaction was irradiated for 24 hrs under 100% light intensity, 365 nm plate.

After working up according to **General Procedure C-2**, the basified, dry organic mixture was subjected to further purification by preparative reverse phase HPLC with 35% to 50% MeCN in water (0.1% NH_4OH modifier). Product containing fractions were concentrated to afford desired product as an off-white solid (30.8 mg, 0.15 mmol, 50% yield, d.r > 10:1 [determined by NMR]).

^{1}H NMR (500 MHz, $CDCl_3$) δ 7.30 – 7.24 (m, 2H), 6.88 – 6.81 (m, 2H), 3.91 (ddd, *J* = 11.1, 3.3, 1.3 Hz, 1H), 3.83 – 3.74 (m, 4H), 3.50 (dq, *J* = 8.7, 6.2 Hz, 1H), 3.38 (d, *J* = 8.7 Hz, 1H), 3.09 (td, *J* = 11.6, 3.3 Hz, 1H), 2.94 (ddd, *J* = 11.6, 2.7, 1.4 Hz, 1H), 1.78 (s, 1H), 0.92 (d, *J* = 6.2 Hz, 3H). **[reported mixture of diastereomers]**

^{13}C NMR (101 MHz, $CDCl_3$) δ 159.3, 133.7, 129.1, 113.9, 78.0, 67.9, 67.1, 55.4, 46.9, 18.3. **[reported mixture of diastereomers]**

HRMS (ESI-TOF) *m*/*z* calcd. for $C_{12}H_{18}NO_2^+$ ($[M+H]^+$) 208.1332, found 208.1337.

Major diastereomer assignment based on literature precedent for (*trans*)-3-(4-methoxyphenyl)-2-methylmorpholine

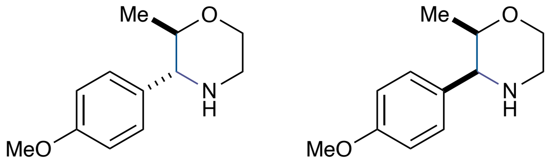

major (*trans*) minor (*cis*)

Wang, Y. Y.; Bode, J. W. *J. Am. Chem. Soc.* **2019**, *141* (24), 9739-9745.

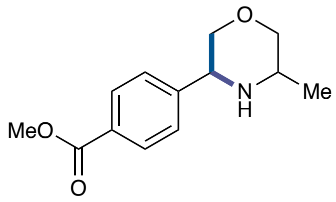

Methyl 4-(5-methylmorpholin-3-yl)benzoate (27)

Prepared according to **General Procedure B-2** with methyl (*E*)-4-(((1-methoxypropan-2-yl)imino)methyl)benzoate (117.6 mg, 0.5 mmol), sodium persulfate (119.1 mg, 0.5 mmol, 1 equiv), benzenesulfonic acid (355.9 mg, 2.25 mmol, 4.5 equiv), $FeCl_3$ (32.4 mg, 0.2 mmol, 40 mol%), bis(4-methoxyphenyl) disulfide (27.8 mg, 0.1 mmol, 20 mol%), and anhydrous MeCN (3.5 mL). The reaction was irradiated for 20 hrs under 100% light intensity, 420 nm plate.

After working up according to **General Procedure B-2**, the mixture was purified by purification ***method (2) acid/base wash***. With the basified, dry organic mixture, Et_2O (10 mL) was added to partially dissolve the mixture, followed by addition of water (10 mL). Then, concentrated HCl was directly added into the mixture. After mixing, the mixture was transferred to separatory funnel. The layer separated and the aqueous layer was collected. Additional water and concentrated HCl were added into the Et_2O layer to allow the second mixing and layer separation. The process was repeated 3 to 4 times. The collected aqueous layer was basified by saturated $NaHCO_3$ solution and 1M NaOH solution. After the pH of the solution was checked by pH indicators to be greater than 10. The aqueous layer was extracted by EtOAc (4 x 20 mL) to afford light yellow oil (67.5 mg, 0.29 mmol, 57% yield, d.r = 4.4:1 [determined by NMR]).

^{1}H NMR (500 MHz, $CDCl_3$) δ 8.03 – 7.93 (m, 2H), 7.51 (dd, *J* = 31.3, 8.1 Hz, 2H), 4.26 – 4.01 (m, 1H), 3.91 (s, 3H), 3.86 – 3.63 (m, 2H), 3.54 – 3.24 (m, 1H), 3.20 – 3.03 (m, 2H), 1.75 (s, 1H), 1.13 (dd, *J* = 87.6, 6.2 Hz, 3H). **[reported mixture of diastereomers]**

^{13}C NMR (126 MHz, $CDCl_3$) δ 167.0, 145.7, 129.9, 129.9, 129.7, 127.7, 127.3, 73.3, 73.0, 72.4, 71.9, 60.7, 54.1, 52.2, 50.9, 46.5, 17.9, 17.8. **[reported mixture of diastereomers]**

HRMS (ESI-TOF) *m/z* calcd. for $C_{13}H_{18}NO_3^+$ ($[M+H]^+$) 236.1281, found 236.1287.

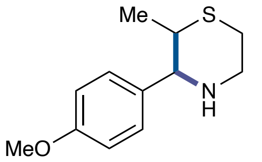

3-(4-Methoxyphenyl)-2-methylthiomorpholine (28)

Prepared according to **General Procedure C-2** with (*E*)-*N*-(2-(ethylthio)ethyl)-1-(4-methoxyphenyl)methanimine (67 mg, 0.3 mmol), trifluoroacetic acid (102.6 mg, 68.9 µL, 0.9 mmol, 3 equiv), TBADT (9.9 mg, 0.003 mmol, 1.0 mol%), bis(4-methoxyphenyl) disulfide (8.4 mg, 0.03 mmol, 10 mol%), $TBABF_4$ (98.8 mg, 0.3 mmol, 1 equiv) and anhydrous MeCN (1.5 mL). The reaction was irradiated for 24 hrs under 100% light intensity, 365 nm plate.

After working up according to **General Procedure C-2**, the basified, dry organic mixture was subjected to further purification by preparative reverse phase HPLC with 35% to 55% MeCN in water (0.1% NH_4OH modifier). Product containing fractions were concentrated to afford desired product as an off-white solid (35.9 mg, 0.16 mmol, 54% yield, d.r > 10:1 [determined by NMR]).

^{1}H NMR (400 MHz, $CDCl_3$) δ 7.25 – 7.21 (m, 2H), 6.89 – 6.80 (m, 2H), 3.80 (s, 3H), 3.44 (d, J = 9.3 Hz, 1H), 3.40 – 3.35 (m, 1H), 3.15 – 3.01 (m, 2H), 2.95 (dq, J = 9.3, 6.8 Hz, 1H), 2.55 – 2.42 (m, 1H), 1.79 (s, 1H), 0.85 (d, J = 6.8 Hz, 3H). **[reported mixture of diastereomers]**

^{13}C NMR (101 MHz, $CDCl_3$) δ 159.3, 135.0, 128.8, 113.9, 70.0, 55.4, 49.2, 40.9, 29.8, 18.1. **[reported mixture of diastereomers]**

HRMS (ESI-TOF) *m/z* calcd. for $C_{12}H_{18}NOS^+$ ($[M+H]^+$) 224.1104, found 224.1109.

Major diastereomer assignment based on ^{1}H NMR and COSY

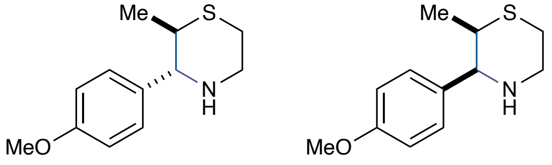

major (*trans*) minor (*cis*)

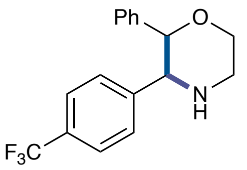

2-Phenyl-3-(4-(trifluoromethyl)phenyl)morpholine (29)

Prepared according to **General Procedure C-2** with (*E*)-*N*-(2-(benzyloxy)ethyl)-1-(4-(trifluoromethyl)phenyl)methanimine (153.7 mg, 0.5 mmol), benzenesulfonic acid (355.9 mg, 2.25 mmol, 4.5 equiv), TBADT (3.7 mg, 0.0025 mmol, 0.5 mol%), bis(4-methoxyphenyl) disulfide (27.8 mg, 0.1 mmol, 20 mol%), $TBABF_4$ (164.6 mg, 0.5 mmol, 1 equiv) and anhydrous MeCN (3.5 mL). The reaction was irradiated for 4 hrs under 100% light intensity, 365 nm plate.

After working up according to **General Procedure C-2**, the basified, dry organic mixture was subjected to further purification by reverse phase chromatography (60 g) with 40% to 55% MeCN in water (0.1% NH_4OH modifier). Product containing fractions were concentrated to afford desired product as an off-white solid (74.1 mg, 0.24 mmol, 48% yield, d.r. as determined by uHPLC area ratio analysis: d.r. = 11.9:1).

^{1}H NMR (500 MHz, $CDCl_3$) δ 7.39 (d, J = 8.0 Hz, 2H), 7.22 – 7.10 (m, 5H), 7.00 (dd, J = 7.1, 2.1 Hz, 2H), 4.32 (d, J = 8.8 Hz, 1H), 4.11 (dd, J = 11.2, 3.3 Hz, 1H), 3.95 (td, J = 11.5, 2.6 Hz, 1H), 3.86 (d, J = 8.8 Hz, 1H), 3.28 (td, J = 11.7, 3.3 Hz, 1H), 3.09 (dd, J = 11.7, 2.5 Hz, 1H), 2.16 (s, 1H). **[reported major diastereomers]**

^{13}C NMR (126 MHz, $CDCl_3$) δ 144.3, 138.7, 129.9, 129.9 (q, J = 32.3 Hz), 128.6, 128.1, 128.0, 127.5, 125.0 (q, J = 3.8 Hz), 85.4, 68.1, 67.3, 46.5. **[reported major diastereomers]**
=

^{19}F NMR (471 MHz, $CDCl_3$) δ -62.50.

HRMS (ESI-TOF) *m*/*z* calcd. for $C_{17}H_{17}F_3NO^+$ ($[M+H]^+$) 308.1257, found 308.1256.

Major diastereomer assignment based on ^{1}H NMR and COSY

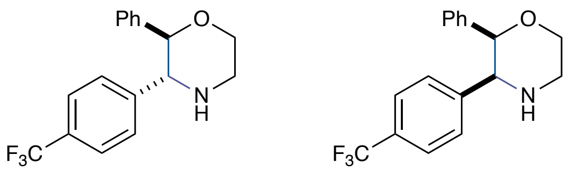

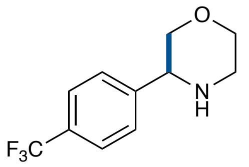

(±)-3-(4-(Trifluoromethyl)phenyl)morpholine (30)

Prepared according to **General Procedure B-2** with (*E*)-*N*-(2-methoxyethyl)-1-(4-(trifluoromethyl)phenyl)methanimine (119.9 mg, 0.5 mmol), sodium persulfate (119.1 mg, 0.5 mmol, 1 equiv), benzenesulfonic acid (355.9 mg, 2.25 mmol, 4.5 equiv), $FeCl_3$ (32.4 mg, 0.2 mmol, 40 mol%), bis(4-methoxyphenyl) disulfide (27.8 mg, 0.1 mmol, 20 mol%), and anhydrous MeCN (3.5 mL). The reaction was irradiated for 30 hrs under 100% light intensity, 420 nm plate.

After working up according to **General Procedure B-2**, the mixture was purified by purification ***method (2) acid/base wash***. With the basified, dry organic mixture, Et_2O (10 mL) was added to partially dissolve the mixture, followed by addition of water (10 mL). Then, concentrated HCl was directly added into the mixture. After mixing, the mixture was transferred to separatory funnel. The layer separated and the aqueous layer was collected. Additional water and concentrated HCl were added into the Et_2O layer to allow the second mixing and layer separation. The process was repeated 3 to 4 times. The collected aqueous layer was basified by saturated $NaHCO_3$ solution and 1M NaOH solution. After the pH of the solution was checked by pH indcators to be greater than 10. The aqueous layer was extracted by EtOAc (4 x 20 mL) to afford light yellow oil (72.4 mg, 0.31 mmol, 63% yield).

^{1}H NMR (500 MHz, MeOD) δ 7.65 (d, *J* = 8.4 Hz, 2H), 7.59 (d, *J* = 8.2 Hz, 2H), 3.99 (dd, *J* = 10.2, 3.2 Hz, 1H), 3.85 (ddt, *J* = 11.1, 5.8, 2.3 Hz, 2H), 3.63 (td, *J* = 11.4, 2.8 Hz, 1H), 3.40 (dd, *J* = 11.2, 10.2 Hz, 1H), 3.06 (ddd, *J* = 12.5, 11.3, 3.4 Hz, 1H), 2.98 (ddd, *J* = 12.5, 2.9, 1.6 Hz, 1H). The hydrogen at NH was not detected by the NMR due to the use of MeOD.

Spectral data are consistent with those reported in literature:
Luescher, M. U.; Bode, J. W. *Synlett*, **2019**, *30*(04), 464-470.

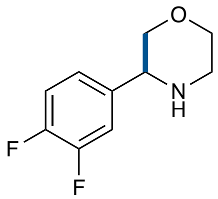

(±)-3-(3,4-Difluorophenyl)morpholine (31)

Prepared according to **General Procedure B-2** with (*E*)-1-(3,4-difluorophenyl)-*N*-(2-methoxyethyl)methanimine (99.6 mg, 0.5 mmol), sodium persulfate (119.1 mg, 0.5 mmol, 1 equiv), benzenesulfonic acid (355.9 mg, 2.25 mmol, 4.5 equiv), $FeCl_3$ (32.4 mg, 0.2 mmol, 40 mol%), bis(4-methoxyphenyl) disulfide (27.8 mg, 0.1 mmol, 20 mol%), and anhydrous MeCN (3.5 mL). The reaction was irradiated for 20 hrs under 100% light intensity, 420 nm plate.

After working up according to **General Procedure B-2**, the mixture was purified by purification ***method (2) acid/base wash***. With the basified, dry organic mixture, Et_2O (10 mL) was added to partially dissolve the mixture, followed by addition of water (10 mL). Then, concentrated HCl was directly added into the mixture. After mixing, the mixture was transferred to separatory funnel. The layer separated and the aqueous layer was collected. Additional water and concentrated HCl were added into the Et_2O layer to allow the second mixing and layer separation. The process was repeated 3 to 4 times. The collected aqueous layer was basified by saturated $NaHCO_3$ solution and 1M NaOH solution. After the pH of the solution was checked by pH indicators to be greater than 10. The aqueous layer was extracted by EtOAc (4 x 20 mL) to afford light yellow oil (51.3 mg, 0.26 mmol, 52% yield).

^{1}H NMR (500 MHz, $CDCl_3$) δ 7.29 – 7.20 (m, 1H), 7.15 – 7.04 (m, 2H), 3.88 (td, *J* = 10.7, 3.2 Hz, 2H), 3.78 (dd, *J* = 11.1, 3.2 Hz, 1H), 3.63 (td, *J* = 11.3, 2.7 Hz, 1H), 3.30 (t, *J* = 10.5 Hz, 1H), 3.11 (td, *J* = 11.5, 3.3 Hz, 1H), 2.99 (dt, *J* = 11.8, 2.1 Hz, 1H), 1.73 (s, 1H).

^{13}C NMR (126 MHz, $CDCl_3$) δ 151.5 (dd, *J* = 248.6, 12.7 Hz), 150.9 (dd, *J* = 248.6, 12.5 Hz), 138.8 – 136.4 (m), 123.2 (dd, *J* = 6.4, 3.6 Hz), 117.3 (d, *J* = 17.3 Hz), 116.2 (d, *J* = 17.8 Hz), 73.7, 67.3, 59.6, 46.5.

^{19}F NMR (471 MHz, $CDCl_3$) δ -137.36, -138.94.

HRMS (ESI-TOF) *m*/*z* calcd. for $C_{10}H_{12}F_2NO^+$ ([M+H]$^+$) 200.0881, found 200.0885.

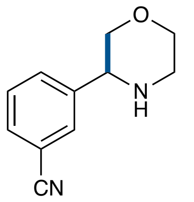

(±)-3-(Morpholin-3-yl)benzonitrile (32)

Prepared according to **General Procedure B-2** with (*E*)-3-(((2-methoxyethyl)imino)methyl)benzonitrile (94.1 mg, 0.5 mmol), sodium persulfate (119.1 mg, 0.5 mmol, 1 equiv), benzenesulfonic acid (355.9 mg, 2.25 mmol, 4.5 equiv), $FeCl_3$ (32.4 mg, 0.2 mmol, 40 mol%), bis(4-methoxyphenyl) disulfide (27.8 mg, 0.1 mmol, 20 mol%), and anhydrous MeCN (3.5 mL). The reaction was irradiated for 20 hrs under 100% light intensity, 420 nm plate.

After working up according to **General Procedure B-2**, the mixture was purified by purification ***method (2) acid/base wash***. With the basified, dry organic mixture, Et_2O (10 mL) was added to partially dissolve the mixture, followed by addition of water (10 mL). Then, concentrated HCl was directly added into the mixture. After mixing, the mixture was transferred to separatory funnel. The layer separated and the aqueous layer was collected. Additional water and concentrated HCl were added into the Et_2O layer to allow the second mixing and layer separation. The process was repeated 3 to 4 times. The collected aqueous layer was basified by saturated $NaHCO_3$ solution and 1M NaOH solution. After the pH of the solution was checked by pH indicators to be greater than 10. The aqueous layer was extracted by EtOAc (4 x 20 mL) to afford light yellow oil (58.3 mg, 0.31 mmol, 63% yield).

^{1}H NMR (500 MHz, $CDCl_3$) δ 7.74 (d, *J* = 1.7 Hz, 1H), 7.63 (dt, *J* = 7.9, 1.6 Hz, 1H), 7.57 (dt, *J* = 7.8, 1.5 Hz, 1H), 7.43 (t, *J* = 7.8 Hz, 1H), 3.97 (dd, *J* = 10.0, 3.2 Hz, 1H), 3.88 (ddt, *J* = 11.1, 3.0, 1.2 Hz, 1H), 3.80 (dd, *J* = 11.1, 3.2 Hz, 1H), 3.64 (td, *J* = 11.3, 2.7 Hz, 1H), 3.33 (dd, *J* = 11.1, 10.0 Hz, 1H), 3.13 (td, *J* = 11.5, 3.3 Hz, 1H), 3.01 (dt, *J* = 11.8, 2.2 Hz, 1H), 1.82 (s, 1H).

^{13}C NMR (126 MHz, $CDCl_3$) δ 142.3, 131.9, 131.60, 131.0, 129.4, 118.8, 112.8, 73.48, 67.33, 59.85, 46.36.

HRMS (ESI-TOF) *m*/*z* calcd. for $C_{11}H_{13}N_2O^+$ ($[M+H]^+$) 189.1022, found 189.1022.

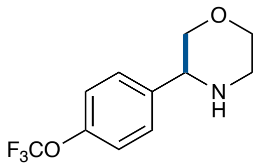

(±)-3-(4-(Trifluoromethoxy)phenyl)morpholine (33)

Prepared according to **General Procedure B-2** with (*E*)-*N*-(2-methoxyethyl)-1-(4-(trifluoromethoxy)phenyl)methanimine (123.6 mg, 0.5 mmol), sodium persulfate (119.1 mg, 0.5 mmol, 1 equiv), benzenesulfonic acid (355.9 mg, 2.25 mmol, 4.5 equiv), $FeCl_3$ (32.4 mg, 0.2 mmol, 40 mol%), bis(4-methoxyphenyl) disulfide (27.8 mg, 0.1 mmol, 20 mol%), and anhydrous MeCN (3.5 mL). The reaction was irradiated for 20 hrs under 100% light intensity, 420 nm plate.

After working up according to **General Procedure B-2**, the mixture was purified by purification ***method (2) acid/base wash***. With the basified, dry organic mixture, Et_2O (10 mL) was added to partially dissolve the mixture, followed by addition of water (10 mL). Then, concentrated HCl was directly added into the mixture. After mixing, the mixture was transferred to separatory funnel. The layer separated and the aqueous layer was collected. Additional water and concentrated HCl were added into the Et_2O layer to allow the second mixing and layer separation. The process was repeated 3 to 4 times. The collected aqueous layer was basified by saturated $NaHCO_3$ solution and 1M NaOH solution. After the pH of the solution was checked by pH indicators to be greater than 10. The aqueous layer was extracted by EtOAc (4 x 20 mL) to afford light yellow oil (80.0 mg, 0.33 mmol, 65% yield).

^{1}H NMR (500 MHz, $CDCl_3$) δ 7.44 (d, *J* = 8.6 Hz, 2H), 7.23 – 6.98 (m, 2H), 4.50 – 4.27 (m, 1H), 3.97 (dd, *J* = 10.1, 3.2 Hz, 1H), 3.89 (ddt, *J* = 11.4, 3.0, 1.2 Hz, 1H), 3.82 (dd, *J* = 11.2, 3.2 Hz, 1H), 3.68 (td, *J* = 11.4, 2.7 Hz, 1H), 3.42 (dd, *J* = 11.3, 10.1 Hz, 1H), 3.13 (td, *J* = 11.6, 3.3 Hz, 1H), 3.03 (dt, *J* = 12.0, 2.2 Hz, 1H).

^{13}C NMR (126 MHz, $CDCl_3$) δ 149.0 – 148.8 (m), 139.3, 128.7, 121.6, 121.2, 73.6, 67.3, 59.9, 46.6.

^{19}F NMR (471 MHz, $CDCl_3$) δ -57.88.

HRMS (ESI-TOF) *m*/*z* calcd. for $C_{11}H_{13}F_3NO_2^+$ ($[M+H]^+$) 248.0893, found 248.0899.

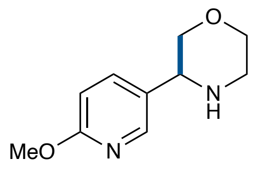

(±)-3-(6-Methoxypyridin-3-yl)morpholine (34)

Prepared according to **General Procedure B-2** with (*E*)-*N*-(2-methoxyethyl)-1-(6-methoxypyridin-3-yl)methanimine (99.1 mg, 0.5 mmol, 98% purity), sodium persulfate (119.1 mg, 0.5 mmol, 1 equiv), benzenesulfonic acid (751.4 mg, 4.75 mmol, 9.5 equiv), $FeCl_3$ (81.1 mg, 0.5 mmol, 1 equiv), bis(4-methoxyphenyl) disulfide (13.9 mg, 0.05 mmol, 10 mol%), and anhydrous MeCN (3.5 mL). The reaction was irradiated for 30 hrs under 100% light intensity, 420 nm plate. *Due to the pyridine coordination to $FeCl_3$, 9.5 equiv of acid and 1 equiv of $FeCl_3$ was used.*

After working up according to **General Procedure B-2** [note: when following the general procedure B-2 to extract from the neutralized crude reaction, ***$CHCl_3$:iPrOH (v:v = 3:1)*** should be used as the organic solvent instead of EtOAc due to the high polarity of the product. Meanwhile, excess EDTA solution as well as NaCl solids were added to capture iron impurities and saturate aqueous layer for better layer separation; careful examination of the aqueous layer is strongly recommended to ensure complete recovery of the product during extraction], the mixture was then purified by purification ***method (3) prepHPLC (basic mode)***. The basified, dry organic mixture was subjected to further purification by preparative reverse phase HPLC with 5% to 40% MeCN in water (0.1% NH_4OH modifier). Product containing fractions were concentrated to afford desired product as an off-white solid (43.5 mg, 0.22 mmol, 45% yield).

^{1}H NMR (500 MHz, $CDCl_3$) δ 8.16 (d, *J* = 2.5 Hz, 1H), 7.67 (dd, *J* = 8.6, 2.4 Hz, 1H), 6.71 (d, *J* = 8.5 Hz, 1H), 3.92 (s, 3H), 3.92 – 3.85 (m, 2H), 3.79 (dd, *J* = 11.5, 3.3 Hz, 1H), 3.69 (td, *J* = 11.4, 2.7 Hz, 1H), 3.54 – 3.39 (m, 1H), 3.11 (td, *J* = 11.6, 3.3 Hz, 1H), 3.03 – 2.96 (m, 1H), 2.80 – 2.67 (m, 1H).

^{13}C NMR (101 MHz, $CDCl_3$) δ 164.2, 146.0, 137.9, 128.2, 111.0, 73.0, 66.9, 57.6, 53.6, 46.3.

HRMS (ESI-TOF) *m*/*z* calcd. for $C_{10}H_{15}N_2O_2^+$ ($[M+H]^+$) 195.1128, found 195.1128.

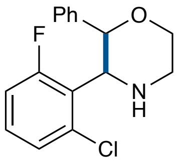

3-(2-Chloro-6-fluorophenyl)-2-phenylmorpholine (35)

Prepared according to **General Procedure C-2** with (*E*)-*N*-(2-(benzyloxy)ethyl)-1-(2-chloro-6-fluorophenyl)methanimine (151.2 mg, 0.5 mmol, 96% purity), benzenesulfonic acid (355.9 mg, 2.25 mmol, 4.5 equiv), TBADT (3.7 mg, 0.0025 mmol, 0.5 mol%), bis(4-methoxyphenyl) disulfide (13.9 mg, 0.05 mmol, 10 mol%), $TBABF_4$ (164.6 mg, 0.5 mmol, 1 equiv) and anhydrous MeCN (3.5 mL). The reaction was irradiated for 6 hrs under 100% light intensity, 365 nm plate.

After working up according to **General Procedure C-2**, the basified, dry organic mixture was subjected to further purification by preparative reverse phase HPLC with 40% to 55% MeCN in water (0.1% NH_4OH modifier). Product containing fractions were concentrated to afford desired product as an off-white solid (35.9 mg, 0.16 mmol, 55% yield, d.r = 4.8:1 [determined by NMR]). **[$TBABF_4$ salt co-eluted with the product after multiple works and chromatography, the 55% isolated yield compensated the mass of TBAfBF$_4$** ($TBABF_4$ NMR: ^{1}H NMR (500 MHz, $CDCl_3$) δ 3.42 – 3.35 (m, 8H), 1.69 (p, *J* = 7.7 Hz, 8H), 1.46 (h, *J* = 7.4 Hz, 8H), 1.01 (t, *J* = 7.3 Hz, 12H). ^{13}C NMR (126 MHz, $CDCl_3$) δ 58.40, 23.73, 19.58, 13.53.)**]**.

^{1}H NMR (500 MHz, $CDCl_3$) δ 7.23 – 7.12 (m, 5H), 7.11 – 6.89 (m, 3H), 5.24 – 4.73 (m, 1H), 4.60 – 4.52 (m, 1H), 4.25 – 4.04 (m, 1H), 3.87 (qd, *J* = 11.5, 3.8 Hz, 1H), 3.25 (tddt, *J* = 16.0, 12.5, 7.0, 2.9 Hz, 1H), 3.14 – 2.82 (m, 1H), 2.19 – 1.98 (m, 1H). **[reported mixtures of diastereomers]**

^{13}C NMR (126 MHz, $CDCl_3$) δ [163.14 (d, *J* = 253.0 Hz), 162.04 (d, *J* = 258.7 Hz)], 138.72, 135.96 (d, *J* = 8.9 Hz), 133.25 (d, *J* = 38.1 Hz), 129.57 (d, *J* = 10.1 Hz), 128.59 (d, *J* = 29.7 Hz), 128.35, 128.13, 128.07, 127.93, 127.34, 127.02, [125.84 (d, *J* = 1.7 Hz), 125.78 (d, *J* = 3.4 Hz)], 125.15 (d, *J* = 16.8 Hz), [115.50 (d, *J* = 25.5 Hz), 114.89 (d, *J* = 24.1 Hz)], 81.73, 68.67, 65.47, 61.84, 56.58, 46.60, 41.79, 39.56, 29.85. **[reported mixtures of diastereomers, pairs of peaks corresponding to two diastereomers have been bracketed]**

^{19}F NMR (471 MHz, $CDCl_3$) δ -99.90, -109.62. **[reported mixtures of diastereomers]**

HRMS (ESI-TOF) *m*/*z* calcd. for $C_{16}H_{16}ClFNO^+$ ($[M+H]^+$) 292.0899, found 292.0902.

12) Complex saturated heterocycle scope via radical sampling

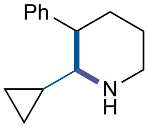

2-Cyclopropyl-3-phenylpiperidine (36)

Prepared according to **General Procedure C-2** with (*E*)-1-cyclopropyl-*N*-(4-phenylbutyl)methanimine (100.7 mg, 0.5 mmol), benzenesulfonic acid (138.4 mg, 0.875 mmol, 1.75 equiv), TBADT (24.9 mg, 0.0075 mmol, 1.5 mol%), bis(4-methoxyphenyl) disulfide (6.96 mg, 0.025 mmol, 5 mol%), and anhydrous MeCN (2.5 mL). The reaction was irradiated for 12 hrs under 100% light intensity, 365 nm plate.

After working up according to **General Procedure C-2**, the basified, dry organic mixture was subjected to further purification by preparative reverse phase HPLC with 45% to 65% MeCN in water (0.1% NH_4OH modifier). Product containing fractions were concentrated to afford desired product as an off-white solid (60.1 mg, 0.30 mmol, 60% yield, d.r > 10:1 [determined by NMR]).

^{1}H NMR (500 MHz, $CDCl_3$) δ 7.27 (d, J = 7.8 Hz, 2H), 7.23 – 7.16 (m, 3H), 3.26 (d, J = 11.8 Hz, 1H), 2.76 (td, J = 11.4, 4.4 Hz, 1H), 2.68 (td, J = 11.5, 3.3 Hz, 1H), 1.98 – 1.91 (m, 2H), 1.83 – 1.71 (m, 2H), 1.65 (qd, J = 12.2, 5.5 Hz, 1H), 1.25 (s, 1H), 0.73 (qt, J = 8.4, 4.9 Hz, 1H), 0.35 – 0.26 (m, 1H), 0.11 (dd, J = 9.6, 5.1 Hz, 1H), -0.00 (tt, J = 8.8, 5.4 Hz, 1H), -0.48 (dq, J = 10.3, 5.2 Hz, 1H). **[reported mixture of diastereomers]**

^{13}C NMR (126 MHz, $CDCl_3$) δ 143.9, 128.3, 128.2, 126.5, 67.2, 50.1, 46.8, 32.6, 25.6, 16.0, 4.8, 1.9. **[reported mixture of diastereomers]**

HRMS (ESI-TOF) *m*/*z* calcd. for $C_{14}H_{20}N^+$ ($[M+H]^+$) 202.1590, found 202.1589.

Major diastereomer assignment based on ^{1}H NMR, COSY and HSQC

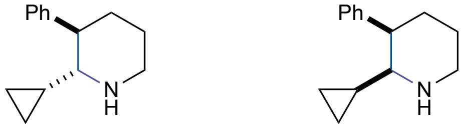

major (*trans*) minor (*cis*)

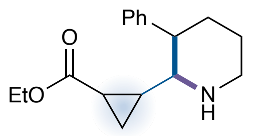

Ethyl 2-(3-phenylpiperidin-2-yl)cyclopropane-1-carboxylate (37)

Prepared according to **General Procedure C-2** with ethyl (*E*)-2-(((4-phenylbutyl)imino)methyl)cyclopropane-1-carboxylate (143.9 mg, 0.5 mmol, 95% purity), benzenesulfonic acid (138.4 mg, 0.875 mmol, 1.75 equiv), TBADT (33.2 mg, 0.01 mmol, 2.0 mol%), bis(4-methoxyphenyl) disulfide (6.96 mg, 0.025 mmol, 5 mol%), and anhydrous MeCN (2.5 mL). The reaction was irradiated for 12 hrs under 100% light intensity, 365 nm plate.

After working up according to **General Procedure C-2**, the basified, dry organic mixture was subjected to further purification by preparative reverse phase HPLC with 45% to 60% MeCN in water (0.1% NH_4OH modifier). Product containing fractions were concentrated to afford desired product as an off-white solid (major diastereomer) and pale yellow oil (mixture of minor diastereomers) (68.5 mg, 0.25 mmol, 50% yield, d.r = 1.85 [major diastereomer] :1 [combination of minor diastereomers], d.r. determined by isolated mass of major and mixture of minor diastereomers).

Major diastereomer characterization
^{1}H NMR (500 MHz, $CDCl_3$) δ 7.28 – 7.23 (m, 2H), 7.22 – 7.16 (m, 1H), 7.16 – 7.11 (m, 1H), 3.89 (dq, *J* = 10.8, 7.2 Hz, 1H), 3.81 (dq, *J* = 10.8, 7.2 Hz, 1H), 3.16 (dp, *J* = 11.6, 1.9 Hz, 1H), 2.70 (td, *J* = 11.7, 2.9 Hz, 1H), 2.55 – 2.47 (m, 1H), 2.03 – 1.52 (m, 7H), 1.32 (tdd, *J* = 9.0, 6.4, 4.2 Hz, 1H), 1.10 (t, *J* = 7.1 Hz, 3H), 0.91 (dt, *J* = 9.0, 4.5 Hz, 1H), 0.77 (dt, *J* = 8.8, 4.5 Hz, 1H), 0.59 (ddd, *J* = 8.5, 6.4, 4.2 Hz, 1H).

^{13}C NMR (126 MHz, $CDCl_3$) δ 173.6, 143.8, 128.5, 127.9, 126.5, 65.9, 60.2, 50.9, 47.3, 32.6, 27.2, 26.6, 20.0, 14.2, 11.9.

HRMS (ESI-TOF) *m*/*z* calcd. for $C_{17}H_{24}NO_2^+$ ($[M+H]^+$) 274.1802, found 274.1804.

Minor diastereomers characterization [reported as a mixture of minor diastereomers]
^{1}H NMR (500 MHz, $CDCl_3$) δ 7.40 – 7.22 (m, 2H), 7.22 – 7.15 (m, 3H), [4.18 – 4.09 (m), 4.04 (q, *J* = 7.1 Hz), 2H], [3.47 (dd, *J* = 10.8, 1.8 Hz), 3.31 – 3.05 (m), 3.09 (d, *J* = 11.2 Hz), 1H], 2.70 (td, *J* = 11.6, 2.7 Hz, 1H), 2.53 (ddd, *J* = 11.6, 9.7, 3.8 Hz, 1H), 2.01 (dd, *J* = 9.8, 8.2 Hz, 1H), 1.96 – 1.41 (m, 5H), [1.38 – 1.23 (m), 1.20 (t, *J* = 7.1 Hz), 5H], [0.76 – 0.68 (m), 0.59 (q, *J* = 4.1 Hz), 1H], [0.33 (d, *J* = 7.3 Hz), 0.12 (ddd, *J* = 8.0, 6.7, 4.7 Hz), 1H]. **[due to generation of the >2 different minor diastereomers, proton counts reported inside the brackets]**

^{13}C NMR (126 MHz, $CDCl_3$) δ 174.0, 143.8, 128.3, 128.2, 126.6, 65.6, 60.5, 50.5, 47.1, 32.8,

28.2, 26.5, 17.6, 14.4, 14.3. **[only reported the *major* minor diastereomer among the minor diastereomer mixtures to avoid messy assignment]**

HRMS (ESI-TOF) *m*/*z* calcd. for $C_{17}H_{24}NO_2^+$ ($[M+H]^+$) 274.1802, found 274.1805.

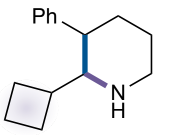

2-Cyclobutyl-3-phenylpiperidine (38)

Prepared according to **General Procedure C-2** with (*E*)-1-cyclopropyl-*N*-(4-phenylbutyl)methanimine (126.7 mg, 0.5 mmol, 85% purity [condensation with lower conversion]), benzenesulfonic acid (138.4 mg, 0.875 mmol, 1.75 equiv), TBADT (33.2 mg, 0.01 mmol, 2.0 mol%), bis(4-methoxyphenyl) disulfide (13.9 mg, 0.05 mmol, 10 mol%), and anhydrous MeCN (5.0 mL). The reaction was irradiated for 12 hrs under 100% light intensity, 365 nm plate.

After working up according to **General Procedure C-2**, the basified, dry organic mixture was subjected to further purification by preparative reverse phase HPLC with 45% to 55% MeCN in water (0.1% NH_4OH modifier). Product containing fractions were concentrated to afford desired product as an off-white solid. (Due to the isolation loss, the yield was determined by assay yield over three replicates using 1,4-nitrobenzene (21 mg, 0.125 mmol, 0.25 equiv, ^{1}H NMR δ 8.42 (s, 4H)) as the internal standard (three replicates: 38%, 31%, 30% yield), the average is 33% yield. d.r > 10:1 [determined by NMR]).

^{1}H NMR (500 MHz, $CDCl_3$) δ 7.25 – 7.22 (m, 2H), 7.17 (dh, *J* = 6.0, 1.6 Hz, 3H), 3.17 (dp, *J* = 11.5, 2.0 Hz, 1H), 2.73 (td, *J* = 11.8, 2.9 Hz, 1H), 2.57 (dd, *J* = 9.7, 7.7 Hz, 1H), 2.25 (ddd, *J* = 11.8, 9.7, 3.7 Hz, 1H), 2.21 – 2.13 (m, 1H), 1.85 (ddt, *J* = 12.9, 5.1, 2.8 Hz, 2H), 1.75 – 1.40 (m, 7H), 1.33 – 1.21 (m, 1H), 1.03 (dddd, *J* = 16.3, 7.5, 4.8, 2.3 Hz, 1H).

^{13}C NMR (126 MHz, $CDCl_3$) δ 144.9, 128.3, 128.1, 126.3, 66.3, 49.9, 47.3, 41.0, 33.9, 27.1, 26.8, 25.0, 18.4. **[trace minor diastereomer was observed]**

HRMS (ESI-TOF) *m*/*z* calcd. for $C_{15}H_{22}N^+$ ([M+H]$^+$) 216.1747, found 216.1747.

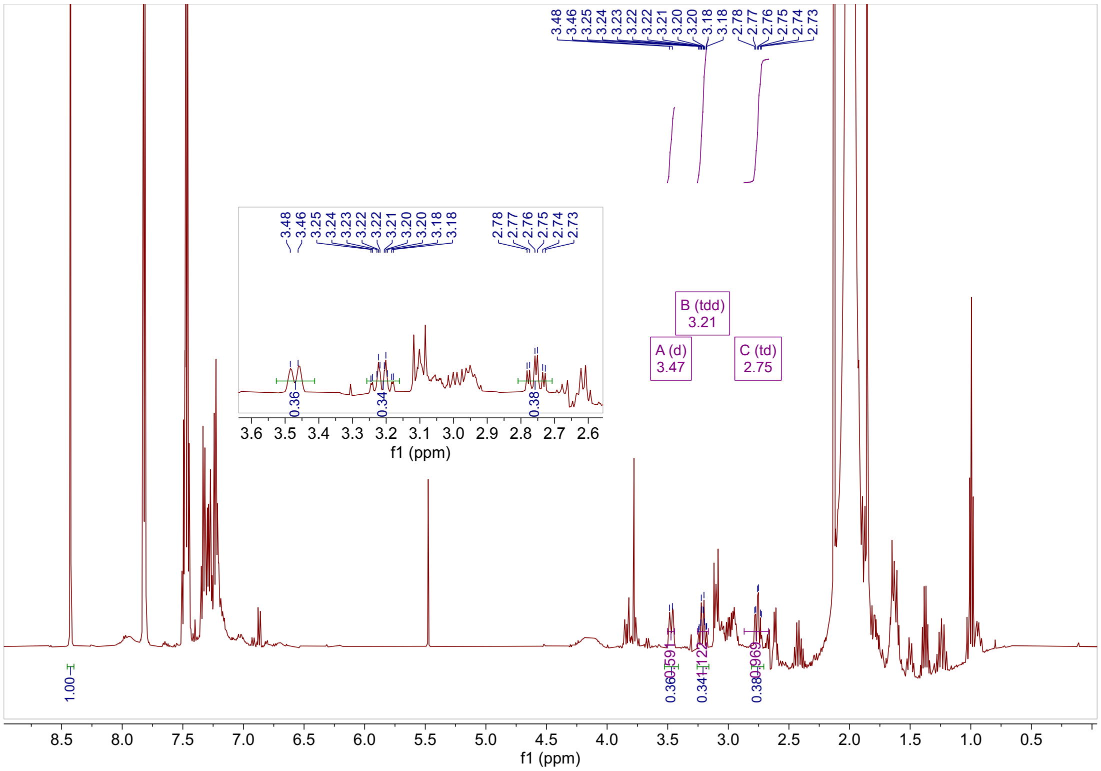

Replicate 1

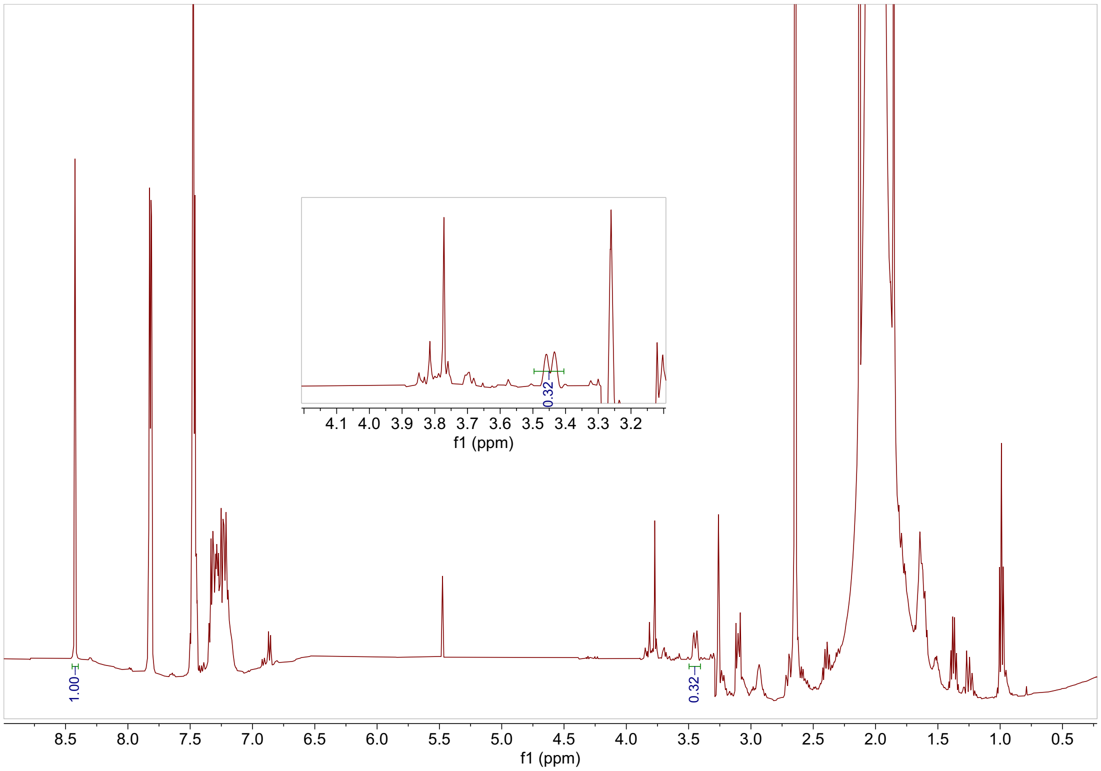

Replicate 2

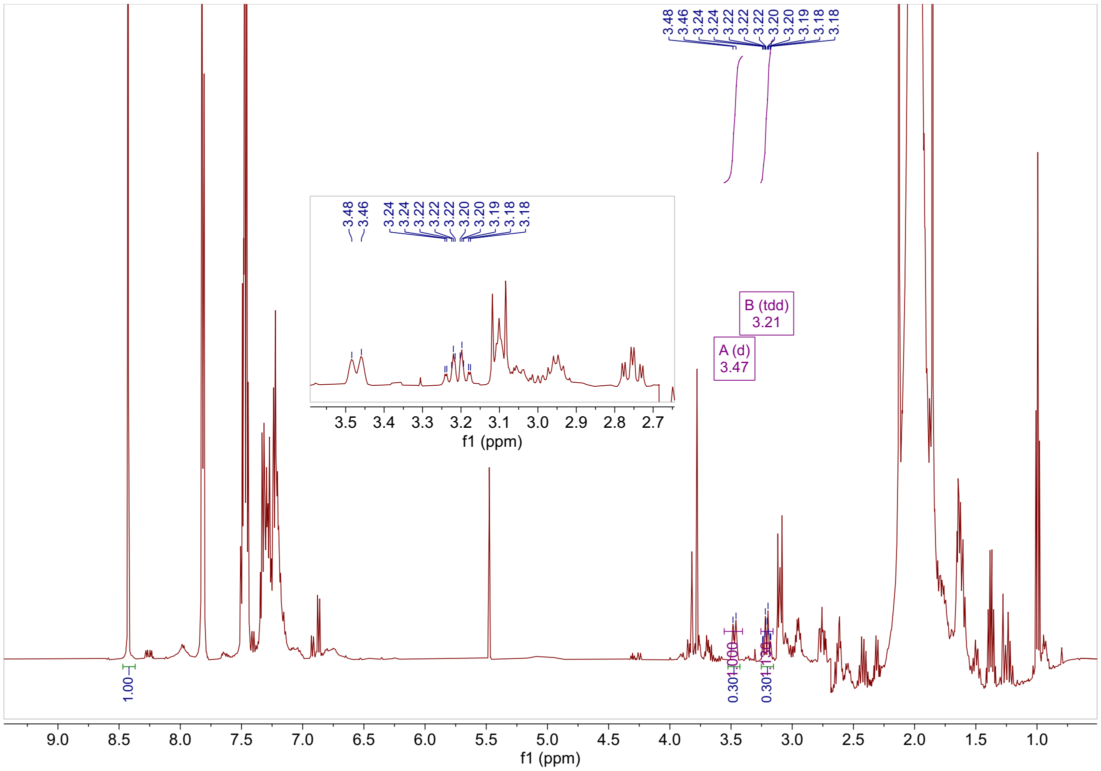
3.48
3.46
3.24
3.24
3.22
3.22
3.22
3.20
3.20
3.19
3.18
3.18
B (tdd)
3.21
A (d)
3.47
3.5 3.4 3.3 3.2 3.1 3.0 2.9 2.8 2.7
f1 (ppm)
1.00
0.301
0.301
9.0 8.5 8.0 7.5 7.0 6.5 6.0 5.5 5.0 4.5 4.0 3.5 3.0 2.5 2.0 1.5 1.0
f1 (ppm)

Replicate 3

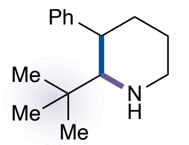

2-(*ert*-Butyl)-3-phenylpiperidine (39)

Prepared according to **General Procedure C-2** with ethyl (*E*)-2,2-dimethyl-*N*-(4-phenylbutyl)propan-1-imine (108.7 mg, 0.5 mmol), benzenesulfonic acid (138.4 mg, 0.875 mmol, 1.75 equiv), TBADT (33.2 mg, 0.01 mmol, 2.0 mol%), bis(4-methoxyphenyl) disulfide (6.96 mg, 0.025 mmol, 5 mol%), and anhydrous MeCN (2.5 mL). The reaction was irradiated for 12 hrs under 100% light intensity, 365 nm plate.

After working up according to **General Procedure C-2**, the basified, dry organic mixture was subjected to further purification by preparative reverse phase HPLC with 45% to 55% MeCN in water (0.1% NH_4OH modifier). Product containing fractions were concentrated to afford desired product as an off-white solid (Due to the isolation loss, the yield was determined by assay yield over three replicates using 1,4-nitrobenzene (21 mg, 0.125 mmol, 0.25 equiv, ^{1}H NMR δ 8.42 (s, 4H) as the internal standard (three replicates: 40%, 42%, 42% yield), the average is 41% yield. d.r > 10:1 [determined by NMR]).

^{1}H NMR (500 MHz, $CDCl_3$) δ 7.24 (d, *J* = 7.4 Hz, 2H), 7.21 – 7.18 (m, 2H), 7.17 – 7.11 (m, 1H), 3.18 (ddt, *J* = 11.7, 4.3, 2.3 Hz, 1H), 2.71 (td, *J* = 11.9, 2.7 Hz, 1H), 2.61 (d, *J* = 9.9 Hz, 1H), 2.53 (ddd, *J* = 11.5, 9.8, 3.7 Hz, 1H), 1.89 – 1.80 (m, 1H), 1.70 – 1.39 (m, 4H), 0.68 (s, 9H).

^{13}C NMR (126 MHz, $CDCl_3$) δ 147.5, 128.3, 128.3, 126.0, 69.6, 47.9, 47.2, 37.0, 35.4, 28.3, 27.2.

HRMS (ESI-TOF) *m*/*z* calcd. for $C_{15}H_{24}N^+$ ($[M+H]^+$) 218.1903, found 218.1907.

Major diastereomer assignment based on ^{1}H NMR and COSY

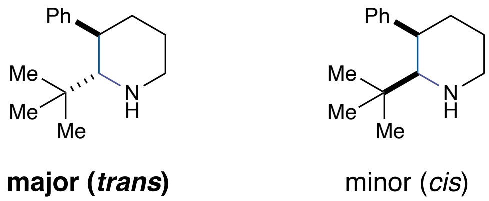

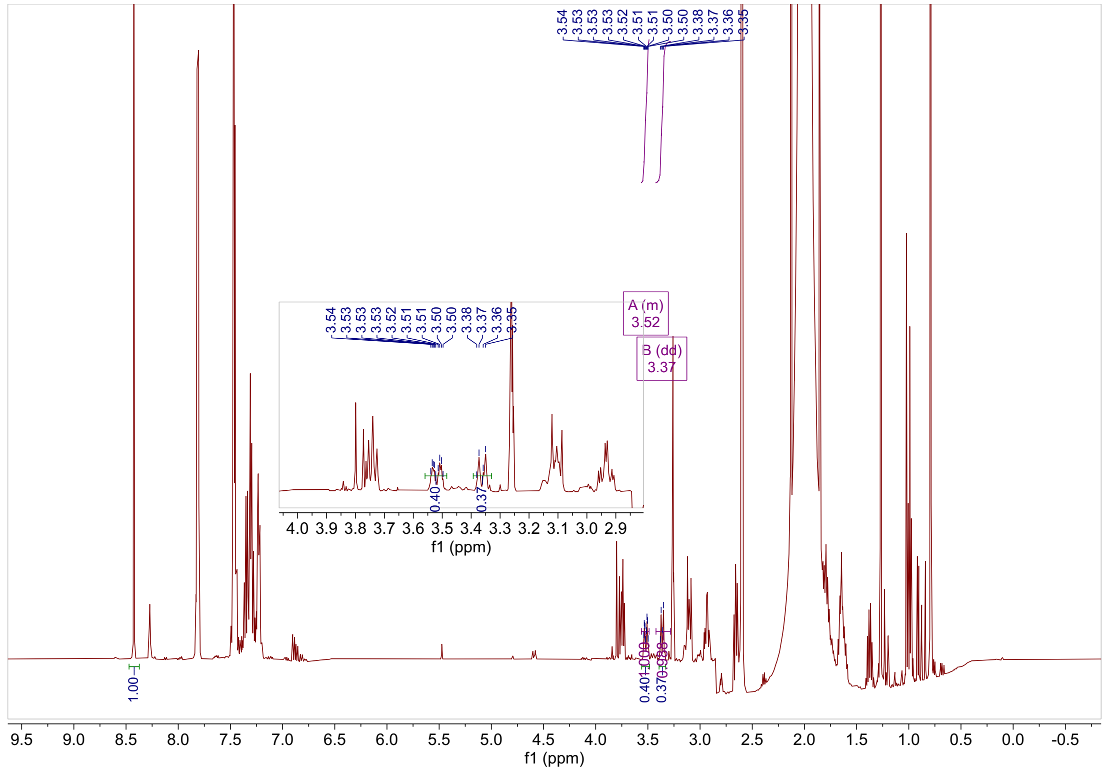

Replicate 1

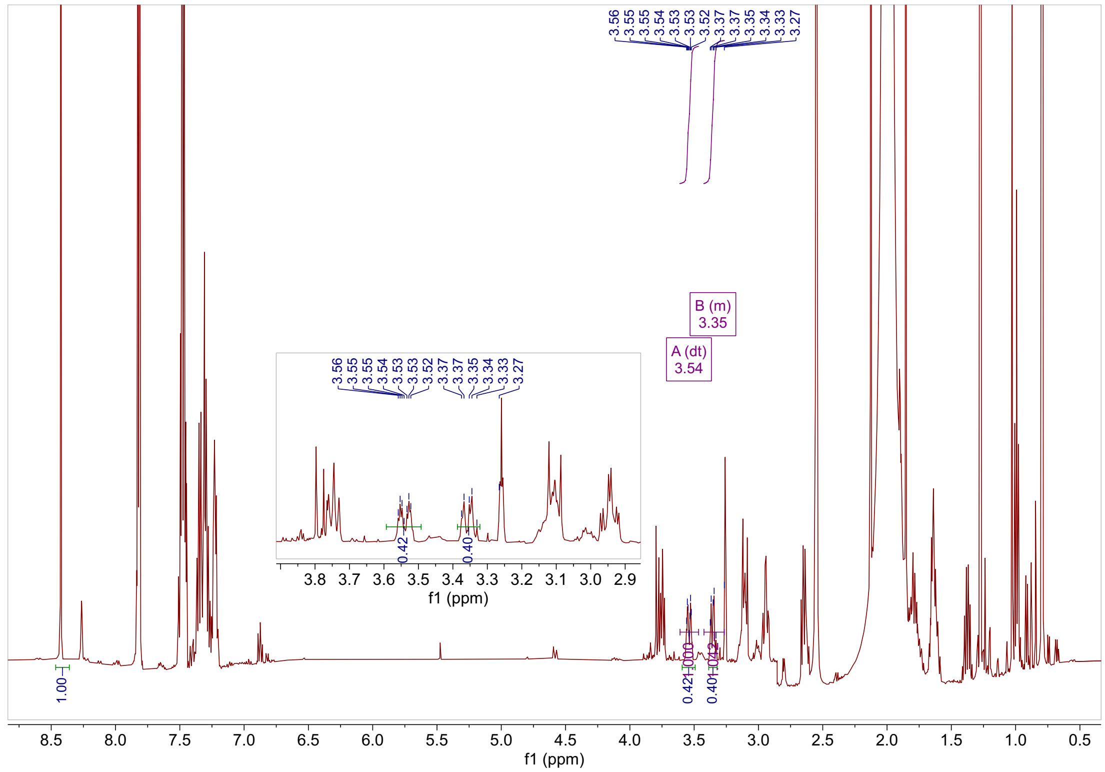

Replicate 2

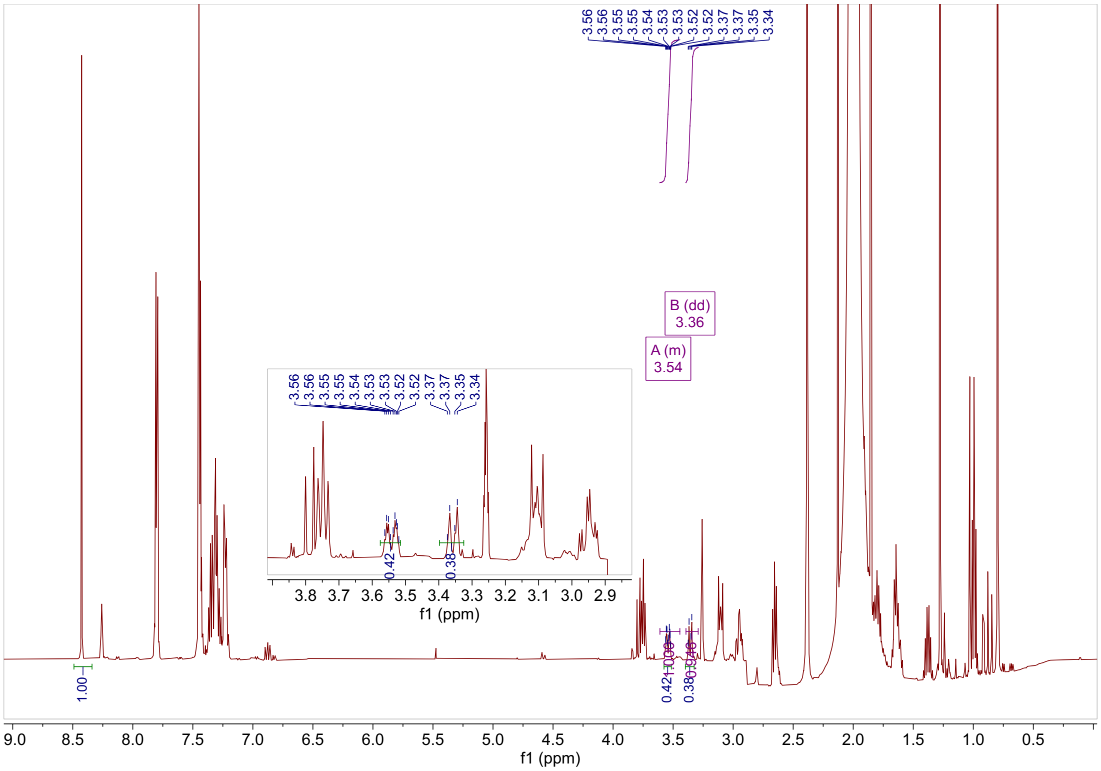
3.56
3.56
3.55
3.55
3.54
3.53
3.53
3.52
3.52
3.37
3.37
3.35
3.34
B (dd)
3.36
A (m)
3.54
1.00
0.42
0.38
f1 (ppm)

Replicate 3

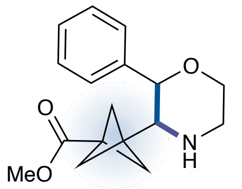

Methyl 3-(2-phenylmorpholin-3-yl)bicyclo[1.1.1]pentane-1-carboxylate (40)

Prepared according to **General Procedure C-2** with methyl (*E*)-3-(((2-(benzyloxy)ethyl)imino)methyl)bicyclo[1.1.1]pentane-1-carboxylate (0.5 mmol, 143.7 mg), benzenesulfonic acid (355.9 mg, 2.25 mmol, 4.5 equiv), TBADT (24.9 mg, 0.0075 mmol, 1.5 mol%), $TBABF_4$ (164.6 mg, 0.5 mmol, 1 equiv) and anhydrous MeCN (3.0 mL). The reaction was irradiated for 12 hrs under 100% light intensity, 365 nm plate **[Due to the activated C–H bond, disulfide was not needed]**.

After working up according to **General Procedure C-2**, the basified, dry organic mixture was subjected to further purification by preparative reverse phase HPLC with 30% to 45% MeCN in water (0.1% NH_4OH modifier). Product containing fractions were concentrated to afford desired product as an off-white solid (90.7 mg, 0.32 mmol, 63% yield, d.r = 4.2:1 [determined by NMR]). **[$TBABF_4$ salt co-eluted with the product after multiple works and chromatography, the 63% isolated yield compensated the mass of $TBABF_4$** ($TBABF_4$ NMR: ^{1}H NMR (500 MHz, DMSO) δ 3.19 – 3.13 (m, 8H), 1.60 – 1.53 (m, 8H), 1.34 – 1.28 (m, 8H), 0.93 (t, *J* = 7.3 Hz, 12H). ^{13}C NMR (101 MHz, DMSO) δ 57.53, 23.05, 19.20, 13.48.)**]**.

^{1}H NMR (500 MHz, DMSO) δ 7.37 – 7.26 (m, 5H), 3.89 (t, *J* = 8.5 Hz, 1H), 3.77 – 3.69 (m, 1H), 3.49 (s, 3H), 3.46 (dd, *J* = 11.0, 3.2 Hz, 1H), 2.89 – 2.77 (m, 3H), 1.63 (dd, *J* = 9.4, 1.8 Hz, 2.38H), 1.50 (d, *J* = 9.5 Hz, 0.57H), 1.39 (dd, *J* = 9.4, 1.7 Hz, 2.39H), 1.29 – 1.24 (m, 0.58H). **[reported mixtures of diastereomers]**

^{13}C NMR (101 MHz, DMSO) δ 169.5, 140.3, 140.2, 128.4, 128.1, 128.1, 128.0, 82.6, 82.4, 67.1, 58.4, 58.2, 51.2, 49.8, 49.5, 45.5, 45.5, 37.5. **[reported mixtures of diastereomers]**

HRMS (ESI-TOF) *m*/*z* calcd. for $C_{17}H_{22}NO_3^+$ ($[M+H]^+$) 288.1594, found 288.1592.

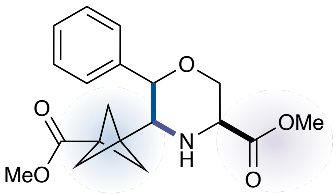

Methyl (3*S*)-5-(3-(methoxycarbonyl)bicyclo[1.1.1]pentan-1-yl)-6-phenylmorpholine-3-carboxylate (41)

Prepared according to **General Procedure C-1** with methyl 3-formylbicyclo[1.1.1]pentane-1-carboxylate (40.0 mg, 0.25 mmol, 96% purity), methyl *O*-benzyl-*L*-serinate (51.3 mg, 0.245 mmol, 0.98 equiv), benzenesulfonic acid (415.2 mg, 2.63 mmol, 10.5 equiv), TBADT (12.5 mg, 0.00375 mmol, 1.5 mol%) and anhydrous MeCN (1.5 mL). The reaction was irradiated for 12 hrs under 100% light intensity, 365 nm plate **[Due to the activated C–H bond, disulfide was not needed]**.

After working up according to **General Procedure C-1**, the basified, dry organic mixture was subjected to further purification by preparative reverse phase HPLC with 30% to 45% MeCN in water (0.1% NH_4OH modifier). Product containing fractions were concentrated to afford desired product as an off-white solid (36.7 mg, 0.11 mmol, 43% yield, d.r = 1.4:1 [determined by NMR]).

^{1}H NMR (500 MHz, $CDCl_3$) δ 7.37 – 7.27 (m, 5H), [4.43 (dd, *J* = 11.6, 1.3 Hz, 0.45H), 4.21 (dd, *J* = 11.1, 3.5 Hz, 0.55H)], [4.02 (d, *J* = 9.5 Hz, 0.4H), 3.96 (d, *J* = 9.2 Hz, 0.6H)], [3.93 (dd, *J* = 11.5, 3.5 Hz, 0.45H), 3.87 – 3.82 (m, 0.55H)], [3.82 (s, 1.3H), 3.75 (s, 1.7H)], 3.61 – 3.51 (m, 4H), [3.38 (d, *J* = 9.4 Hz, 0.45H), 3.08 (d, *J* = 9.2 Hz, 0.55H)], 2.36 (s, 1H), [1.78 (dd, *J* = 9.5, 1.9 Hz, 1.7H), 1.75 (dd, *J* = 9.4, 1.9 Hz, 1.3H)], 1.57 (ddd, *J* = 9.3, 7.3, 1.9 Hz, 3H). **[reported mixtures of diastereomers, pairs of peaks corresponding to two diastereomers have been bracketed]**

^{13}C NMR (126 MHz, $CDCl_3$) δ 173.6, 170.8, 170.3, 170.2, 138.9, 138.8, 129.0, 128.9, 128.7, 128.6, 128.4, 128.3, 83.2, 83.0, 68.8, 67.7, 58.2, 57.4, 55.8, 54.3, 52.4, 52.3, 51.7, 51.7, 40.1, 39.9, 38.3, 38.2. **[reported mixtures of diastereomers]**

HRMS (ESI-TOF) *m*/*z* calcd. for $C_{19}H_{24}NO_5^+$ ($[M+H]^+$) 346.1649, found 346.1649.

Major diastereomer assignment based on ^{1}H NMR, COSY, NOSEY

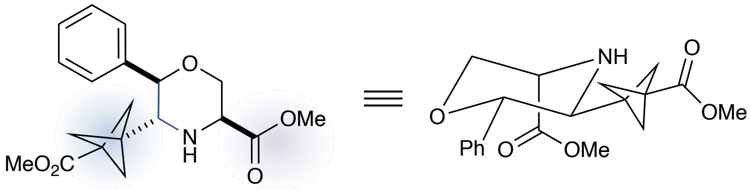

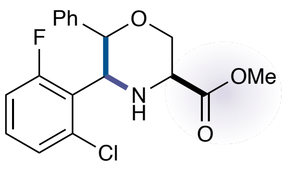

Methyl (3*S*)-5-(2-chloro-6-fluorophenyl)-6-phenylmorpholine-3-carboxylate (42)

Prepared according to **General Procedure C-1** with 2-chloro-6-fluorobenzaldehyde (83.5 mg, 0.5 mmol, 95% purity), methyl *O*-benzyl-*L*-serinate (115.1 mg, 0.55 mmol, 1.1 equiv), benzenesulfonic acid (355.9 mg, 2.25 mmol, 4.5 equiv), TBADT (8.3 mg, 0.0025 mmol, 0.5 mol%), bis(4-methoxyphenyl) disulfide (13.9 mg, 0.05 mmol, 10 mol%), $TBABF_4$ (164.6 mg, 0.5 mmol, 1 equiv) and anhydrous MeCN (3.5 mL). The reaction was irradiated for 8 hrs under 100% light intensity, 365 nm plate.

After working up according to **General Procedure C-1**, the basified, dry organic mixture was subjected to further purification by preparative reverse phase HPLC with 30% to 45% MeCN in water (0.1% NH_4OH modifier). Product containing fractions were concentrated to afford desired product as an off-white solid (83.5 mg, 0.24 mmol, 48% yield). Based on NMR analysis, multiple (>2) diastereomers were generated as cyclized products. The major diastereomer was successfully isolated while other minor diastereomer were isolated as the mixtures. d.r. as determined by uHPLC area ratio analysis: d.r. = 1.68 [combined minor diastereomers] : 1 [major diastereomer]).

Major diastereomer characterization
^{1}H NMR (500 MHz, $CDCl_3$) δ 7.16 (pd, *J* = 4.2, 2.8 Hz, 5H), 7.08 (td, *J* = 8.1, 5.7 Hz, 1H), 6.99 (d, *J* = 8.1 Hz, 1H), 6.96 – 6.88 (m, 1H), 5.09 – 4.90 (m, 2H), 4.61 (dd, *J* = 11.6, 1.2 Hz, 1H), 4.18 (dd, *J* = 11.7, 3.5 Hz, 1H), 3.90 (s, 3H), 3.74 (d, *J* = 3.4 Hz, 1H), 2.48 (s, 1fH).

^{13}C NMR (126 MHz, $CDCl_3$) δ 173.1, 162.3 (d, *J* = 246.8 Hz), 138.3, 129.6 (d, *J* = 10.2 Hz), 128.2, 128.0, 127.8, 127.0, 125.8, 125.0 (d, *J* = 16.0 Hz), 114.9 (d, *J* = 24.2 Hz), 81.0, 68.3, 57.6, 56.1, 52.4.

^{19}F NMR (471 MHz, $CDCl_3$) δ -108.08.

HRMS (ESI-TOF) *m*/*z* calcd. for $C_{18}H_{18}ClFNO_3^+$ ($[M+H]^+$) 350.0954, found 350.0953.

Minor diastereomers characterization [reported as a mixture of minor diastereomers]
^{1}H NMR (500 MHz, $CDCl_3$) [δ 7.25 – 7.21 (m), 7.17 (d, *J* = 4.1 Hz), 7.14 – 6.88 (m), 6.79 – 6.54 (m), 8H], [5.19 – 5.07 (m), 4.80 (dd, *J* = 9.6, 3.2 Hz), 4.64 (d, *J* = 9.4 Hz), 4.57 (d, *J* = 9.6 Hz), 4.37 (td, *J* = 12.2, 3.6 Hz), 4.18 (dd, *J* = 11.9, 5.6 Hz), 3.99 (t, *J* = 5.7 Hz), 3.86 – 3.70 (m), 8H], 3.35 (s, 1H). **[due to generation of the 4 different minor diastereomers, proton counts reported inside the brackets]**

^{19}F NMR (471 MHz, $CDCl_3$) δ -97.48 (d, J = 9.2 Hz), -107.15 (dd, J = 11.6, 5.6 Hz), -109.18 (t, J = 8.2 Hz), -111.01 (dd, J = 9.5, 5.9 Hz).

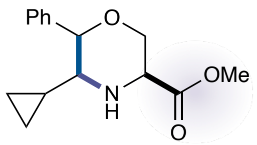

Methyl (3*S*)-5-cyclopropyl-6-phenylmorpholine-3-carboxylate (43)

Prepared according to **General Procedure C-1** with cyclopropanecarbaldehyde (36.1 mg, 38.5 μL, 0.5 mmol, 97% purity), methyl *O*-benzyl-*L*-serinate (115.1 mg, 0.55 mmol, 1.1 equiv), trifluoroacetic acid (85.5 mg, 57.4 μL, 0.75 mmol, 1.5 equiv), TBADT (8.3 mg, 0.0025 mmol, 0.5 mol%), bis(4-methoxyphenyl) disulfide (13.9 mg, 0.05 mmol, 10 mol%) and anhydrous MeCN (3.5 mL). The reaction was irradiated for 24 hrs under 100% light intensity, 365 nm plate.

After working up according to **General Procedure C-1**, the basified, dry organic mixture was subjected to further purification by preparative reverse phase HPLC with 40% to 55% MeCN in water (0.1% NH_4OH modifier). Product containing fractions were concentrated to afford desired product as an off-white solid (58 mg, 0.22 mmol, 44% yield, d.r = 1.5:1 [determined by NMR]).

^{1}H NMR (500 MHz, $CDCl_3$) δ 7.35 – 7.20 (m, 5H), 4.46 (dd, *J* = 11.5, 1.3 Hz, 0.6H), 4.29 – 4.21 (m, 1H), 4.19 (d, *J* = 8.9 Hz, 0.4H), 3.97 (dd, *J* = 11.5, 3.5 Hz, 0.6H), 3.79 (s, 2H), 3.76 (d, *J* = 3.5 Hz, 0.4H), 3.74 (s, 1H), [3.63 (t, *J* = 10.7 Hz, 0.4H), 3.58 (d, *J* = 3.3 Hz, 0.6H)], 2.39 (s, 1H), [2.20 (t, *J* = 9.1 Hz, 0.6H), 1.94 (t, *J* = 9.0 Hz, 0.4H)], [0.69 (ddt, *J* = 13.6, 8.5, 4.2 Hz, 0.4H), 0.59 (tq, *J* = 8.6, 4.3 Hz, 0.6H)], 0.34 (dtd, *J* = 18.3, 8.7, 4.9 Hz, 1H), [0.08 (dq, *J* = 9.9, 5.1 Hz, 0.7H), -0.01 (ddd, *J* = 17.4, 8.8, 4.7 Hz, 0.3H)], -0.57 – -0.67 (m, 1H). **[reported mixtures of diastereomers, pairs of peaks corresponding to two diastereomers have been bracketed]**

^{13}C NMR (126 MHz, $CDCl_3$) δ 173.7, 170.8, 139.7, 139.4, 128.2, 128.1, 128.0, 127.8, 127.7, 85.2, 84.8, 68.6, 67.6, 65.9, 62.0, 57.8, 56.3, 52.31, 52.29, 13.8, 13.5, 4.0, 3.9, 2.0, 1.7. **[reported mixtures of diastereomers]**

HRMS (ESI-TOF) *m*/*z* calcd. for $C_{15}H_{20}NO_3^+$ ($[M+H]^+$) 262.1438, found 262.1444.

13) Mechanistic substrate characterization

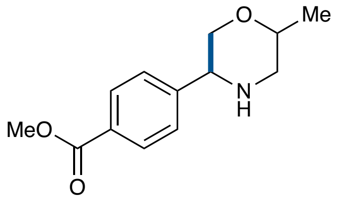

Methyl 4-(-6-methylmorpholin-3-yl)benzoate (S-1)

Prepared according to **General Procedure A-2** with methyl (*E*)-4-(((2-methoxypropyl)imino)methyl)benzoate (72.8 mg, 0.3 mmol), benzenesulfonic acid (213.5 mg, 1.35 mmol, 4.5 equiv), $FeCl_3$ (19.5 mg, 0.12 mmol, 40 mol%), bis(4-methoxyphenyl) disulfide (16.7 mg, 0.06 mmol, 20 mol%), and anhydrous MeCN (2.1 mL). The reaction was irradiated for 20 hrs under 100% light intensity, 420 nm plate.

After working up according to **General Procedure A-2**, the mixture was purified by purification ***method (2) acid/base wash***. With the basified, dry organic mixture, Et_2O (10 mL) was added to partially dissolve the mixture, followed by addition of water (10 mL). Then, concentrated HCl was directly added into the mixture. After mixing, the mixture was transferred to separatory funnel. The layer separated and the aqueous layer was collected. Additional water and concentrated HCl were added into the Et_2O layer to allow the second mixing and layer separation. The process was repeated 3 to 4 times. The collected aqueous layer was basified by saturated $NaHCO_3$ solution and 1M NaOH solution. After the pH of the solution was checked by pH indicators to be greater than 10. The aqueous layer was extracted by EtOAc (4 x 20 mL) to afford light yellow oil (26.6 mg, 0.11 mmol, 38% yield, d.r > 10:1 [determined by NMR]).

^{1}H NMR (500 MHz, $CDCl_3$) δ 8.05 – 7.96 (m, 2H), 7.65 – 7.38 (m, 2H), 3.95 (dd, *J* = 9.7, 3.2 Hz, 1H), 3.91 (s, 3H), 3.86 (dd, *J* = 11.2, 3.2 Hz, 1H), 3.69 (dqd, *J* = 12.4, 6.1, 2.2 Hz, 1H), 3.43 (t, *J* = 10.7 Hz, 1H), 3.06 (dd, *J* = 11.6, 2.4 Hz, 1H), 2.74 (t, *J* = 10.9 Hz, 1H), 1.84 (s, 1H), 1.33 – 1.16 (m, 3H). **[reported mixture of diastereomers]**

^{13}C NMR (126 MHz, $CDCl_3$) δ 167.0, 145.7, 130.0, 129.8, 129.8, 127.9, 127.3, 73.7, 72.5, 71.2, 68.1, 59.9, 53.1, 52.3, 52.2, 19.1, 17.7. **[reported mixture of diastereomers]**

HRMS (ESI-TOF) *m/z* calcd. for $C_{13}H_{18}NO_3^+$ ($[M+H]^+$) 236.1281, found 236.1283.

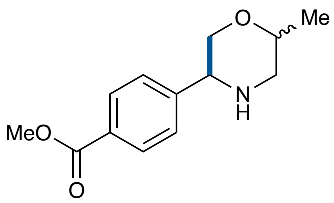

Methyl 4-(6-methylmorpholin-3-yl)benzoate (45)

Prepared according to **General Procedure A-2** with methyl (*R*,*E*)-4-(((2-methoxypropyl)imino)methyl)benzoate (72.8 mg, 0.3 mmol), benzenesulfonic acid (213.5 mg, 1.35 mmol, 4.5 equiv), $FeCl_3$ (19.5 mg, 0.12 mmol, 40 mol%), bis(4-methoxyphenyl) disulfide (16.7 mg, 0.06 mmol, 20 mol%), and anhydrous MeCN (2.1 mL). The reaction was irradiated for 20 hrs under 100% light intensity, 420 nm plate.

After working up according to **General Procedure A-2**, the mixture was purified by purification ***method (2) acid/base wash***. With the basified, dry organic mixture, Et_2O (10 mL) was added to partially dissolve the mixture, followed by addition of water (10 mL). Then, concentrated HCl was directly added into the mixture. After mixing, the mixture was transferred to separatory funnel. The layer separated and the aqueous layer was collected. Additional water and concentrated HCl were added into the Et_2O layer to allow the second mixing and layer separation. The process was repeated 3 to 4 times. The collected aqueous layer was basified by saturated $NaHCO_3$ solution and 1M NaOH solution. After the pH of the solution was checked by pH indicators to be greater than 10. The aqueous layer was extracted by EtOAc (4 x 20 mL) to afford light yellow oil (33 mg, 0.14 mmol, 47% yield, d.r = 8.8:1 [determined by NMR]).

^{1}H NMR (500 MHz, $CDCl_3$) δ 8.09 – 7.97 (m, 2H), 7.64 – 7.46 (m, 2H), 4.15 – 4.07 (m, 0.2H), 3.98 (dd, *J* = 10.1, 3.3 Hz, 1H), 3.94 (d, *J* = 4.8 Hz, 3H), 3.89 (dd, *J* = 11.1, 3.2 Hz, 1H), 3.72 (dqd, *J* = 12.3, 6.1, 2.2 Hz, 0.8H), 3.54 – 3.29 (m, 1H), 3.09 (dd, *J* = 11.7, 2.3 Hz, 1H), 2.77 (dd, *J* = 11.7, 10.1 Hz, 1H), 2.07 – 1.95 (m, 1H), 1.26 (dd, *J* = 41.5, 6.4 Hz, 3H). **[reported mixture of diastereomers]**

^{13}C NMR (126 MHz, $CDCl_3$) δ 167.0, 145.5, 130.0, 129.9, 129.8, 128.0, 127.3, 73.6, 72.4, 71.2, 68.0, 60.0, 53.0, 52.3, 19.0, 17.8. **[reported mixture of diastereomers]**

HRMS (ESI-TOF) *m/z* calcd. for $C_{13}H_{18}NO_3^+$ ($[M+H]^+$) 236.1281, found 236.1282.

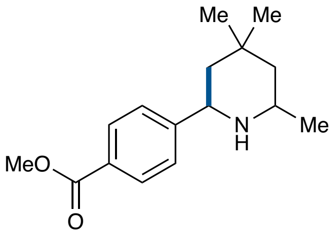

Methyl 4-(4,4,6-trimethylpiperidin-2-yl)benzoate (S-2)

Prepared according to **General Procedure A-1** with methyl 4-formylbenzoate (51.8 mg, 0.3 mmol, 95% purity), 4,4-dimethylpentan-2-amine (40.0 mg, 0.33 mmol, 95% purity), benzenesulfonic acid (189.8 mg, 1.2 mmol, 4 equiv), $FeCl_3$ (9.7 mg, 0.06 mmol, 20 mol%), bis(4-methoxyphenyl) disulfide (25.1 mg, 0.09 mmol, 30 mol%), and anhydrous MeCN (1.5 mL). The reaction was irradiated for 12 hrs under 100% light intensity, 365 nm plate.

After working up according to **General Procedure A-1**, the mixture was purified by purification ***method (3) prepHPLC (basic mode)***. The basified, dry organic mixture was subjected to further purification by preparative reverse phase HPLC. The gradient started with a flash in 32% MeCN in water (0.1% NH_4OH modifier) for 2 min, then increased to 50% followed by slow gradient from 50% to 65 % MeCN in water (0.1% NH_4OH modifier). Product containing fractions were concentrated to afford desired product as an off-white solid (53.7 mg, 0.21 mmol, 68% yield, d.r > 10:1 [determined by NMR]).

^{1}H NMR (500 MHz, $CDCl_3$) δ 7.97 (d, J = 8.4 Hz, 2H), 7.44 (d, J = 8.4 Hz, 2H), 3.95 – 3.90 (m, 1H), 3.90 (s, 3H), 3.01 (dqd, J = 12.3, 6.2, 2.5 Hz, 1H), 1.62 (s, 1H), 1.46 (dt, J = 13.0, 2.5 Hz, 1H), 1.38 (dt, J = 13.0, 2.4 Hz, 1H), 1.29 (t, J = 12.4 Hz, 1H), 1.09 (d, J = 6.2 Hz, 3H), 1.06 (s, 3H), 0.95 (s, 3H).

^{13}C NMR (126 MHz, $CDCl_3$) δ 167.2, 150.9, 129.8, 128.9, 126.8, 57.6, 52.1, 48.3, 47.7, 47.2, 33.3, 31.0, 25.2, 23.1.

HRMS (ESI-TOF) m/z calcd. for $C_{16}H_{24}NO_2^+$ ($[M+H]^+$) 262.1802, found 262.1803.

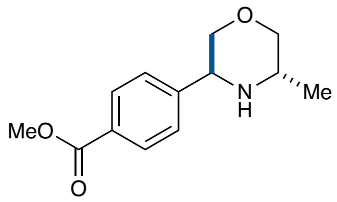

Methyl 4-((5*S*)-5-methylmorpholin-3-yl)benzoate (46)

Prepared according to **General Procedure B-2** with methyl (*R*,*E*)-4-(((1-methoxypropan-2-yl)imino)methyl)benzoate (117.6 mg, 0.5 mmol), sodium persulfate (119.1 mg, 0.5 mmol, 1 equiv), benzenesulfonic acid (355.9 mg, 2.25 mmol, 4.5 equiv), $FeCl_3$ (32.4 mg, 0.2 mmol, 40 mol%), bis(4-methoxyphenyl) disulfide (27.8 mg, 0.1 mmol, 20 mol%), and anhydrous MeCN (3.5 mL). The reaction was irradiated for 20 hrs under 100% light intensity, 420 nm plate.

After working up according to **General Procedure B-2**, the mixture was purified by purification ***method (2) acid/base wash***. With the basified, dry organic mixture, Et_2O (10 mL) was added to partially dissolve the mixture, followed by addition of water (10 mL). Then, concentrated HCl was directly added into the mixture. After mixing, the mixture was transferred to separatory funnel. The layer separated and the aqueous layer was collected. Additional water and concentrated HCl were added into the Et_2O layer to allow the second mixing and layer separation. The process was repeated 3 to 4 times. The collected aqueous layer was basified by saturated $NaHCO_3$ solution and 1M NaOH solution. After the pH of the solution was checked by pH indicators to be greater than 10. The aqueous layer was extracted by EtOAc (4 x 20 mL) to afford light yellow oil (75.8 mg, 0.32 mmol, 64% yield, d.r. not determined).

^{1}H NMR (500 MHz, MeOD) δ 8.03 (d, *J* = 8.4 Hz, 2H), 7.64 (d, *J* = 8.4 Hz, 2H), 4.22 (dd, *J* = 6.5, 3.5 Hz, 1H), 3.93 (s, 3H), 3.91 (d, *J* = 3.4 Hz, 1H), 3.81 (td, *J* = 11.7, 4.1 Hz, 3H), 3.50 (dd, *J* = 11.2, 5.1 Hz, 1H), 3.12 (dtd, *J* = 11.8, 5.8, 3.1 Hz, 1H), 1.24 (d, *J* = 6.7 Hz, 3H).

^{13}C NMR (101 MHz, MeOD) δ 168.4, 147.5, 130.6, 130.4, 130.0, 73.0, 72.1, 54.8, 52.6, 47.3, 16.9.

HRMS (ESI-TOF) *m/z* calcd. for $C_{13}H_{18}NO_3^+$ ($[M+H]^+$) 236.1281, found 236.1285.

14) Diastereoselectivity and major diastereomer discussion

Part I: Overall trends of diastereoselectivity

Based on the assignment of the major diastereomer for multiple substrates across piperidines, morpholines and thiomorpholine (**Figure S13**), *trans* diastereomer is favored across all the substrates. This likely resulted from avoidance of steric clash during the cyclization and is consistent with results from other methodologies which utilize free radical cyclization on to imines to form N-heterocycles.[16-18]

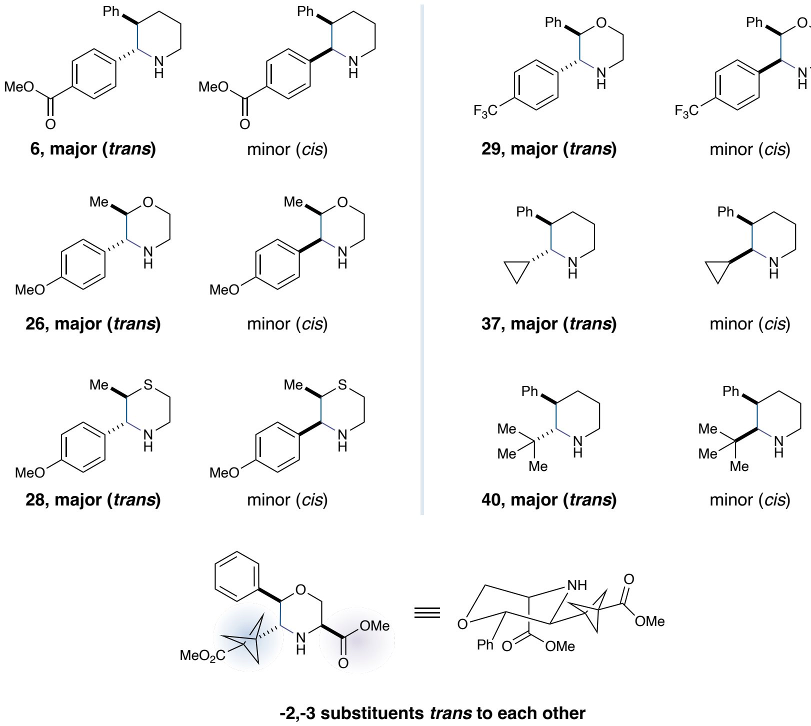

Figure S13. Summary of major diastereomers.

Part II: Diastereoselectivity ratio discussion

Based on **Figure S14**, mediated by different hydrogen atom abstraction (HAA) catalysts, the diastereoselectivity across different substrates varies. For $FeCl_3$ mediated cyclization, the diastereoselectivity ratio is observed to be less than 10:1. For TBADT mediated cyclization, multiple diastereoselectivity ratios are observed to be greater than 10:1. One possible explanation to these observed trends may be the potential HAA events after ring formations. Based on literature surrounding other approaches towards N-heterocycles via radical cyclization onto imine, the initial cyclization is fundamentally a highly diasteroselective process (>10:1 for most examples).[16-18] Thus, irrespective of which catalyst is utilized the initial product is likely >10:1 d.r. However, after cyclization HAA from the product may or may not occur at these chiral sites. With $FeCl_3$ which employs chlorine radical as the HAA species, with less steric bulk, HAA may happen after the cyclization, especially at the generated tertiary (benzylic/α-oxy) C–H bonds. While with TBADT, due to the increased steric bulk, the HAA at the tertiary C–H bonds is relatively hindered. Although

there are literature examples of DT abstracting C-H bonds at tertiary positions,[19] we have seen that it is context and condition dependent. In fact, work from our own laboratory has shown that these positions are not racemized during a late state alpha amino C–H methylation.[20] We should note that the diastereoselectivity will also depends on each substrate to a certain extent. Lastly, when substrates have three different substituents, the diastereoselectivity is lower.

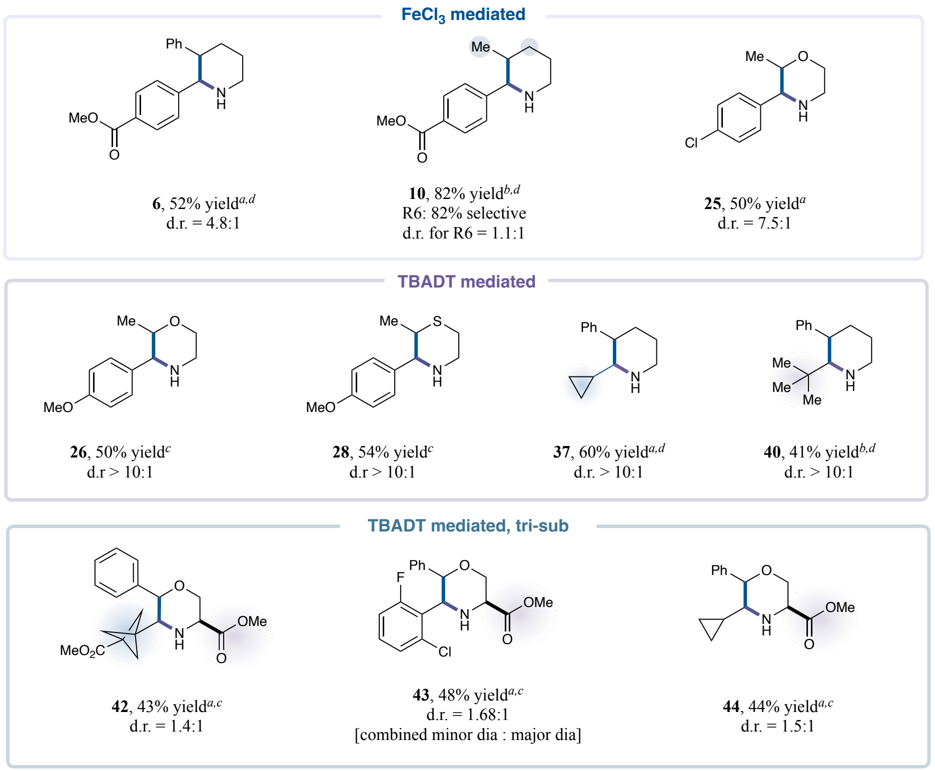

Figure S14. Diastereoselectivity with different hydrogen atom abstraction catalysts.

15) Structure elucidation of the side product from deuterium labeling studies

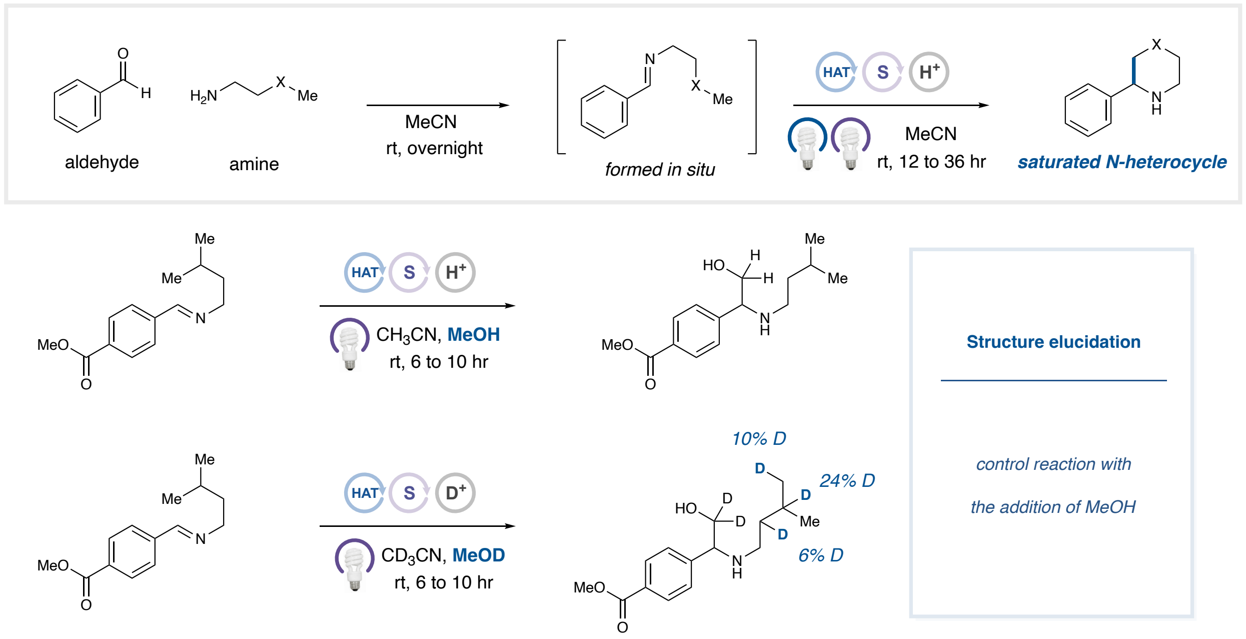

Part I: control reaction without deuteration

To probe the structure of the side product from the deuterium labeling study, a control reaction (no deuterium but with the addition of MeOH) was carried out following the protocol of deuterium labeling.

__Reaction vial__: To an oven-dried 40-mL vial, methyl (*E*)-4-((isopentylimino)methyl)benzoate (116.7 mg, 0.5 mmol), benzenesulfonic acid (316.4 mg, 2.0 mmol, 4 equiv), $FeCl_3$ (16.2 mg, 0.1 mmol, 20 mol%), bis(4-methoxyphenyl) disulfide (41.8 mg, 0.3 mmol, 30 mol%) are dissolved in anhydrous MeCN (2.5 mL). **Then, MeOH (40.5 µL, 1.0 mmo, 2 equiv) was added to the mixture.** The reaction mixture was sparged under nitrogen and sealed with parafilm. The reaction was then irradiated under 365 nm light with 100% light intensity for 12 hours.

After working up according to **General Procedure A-2**, the basified, dry organic mixture was subjected to further purification by preparative reverse phase HPLC with 45% to 55% MeCN in water (0.1% NH_4OH modifier). Product containing fractions were concentrated to afford desired product as a clear oil.

__NMR characterization__: **^{1}H NMR (500 MHz, $CDCl_3$)** δ 8.02 (d, *J* = 8.1 Hz, 2H), 7.37 (d, *J* = 8.0 Hz, 2H), 3.91 (s, 3H), 3.82 (dd, *J* = 8.5, 4.4 Hz, 1H), 3.72 (dd, *J* = 10.7, 4.4 Hz, 1H), 3.52 (dd, *J* = 10.7, 8.4 Hz, 1H), 2.58 – 2.43 (m, 2H), 2.10 – 1.92 (m, 1H), 1.62 (dq, *J* = 13.4, 6.7 Hz, 1H), 1.36 (qd, *J* = 6.9, 2.3 Hz, 2H), 0.86 (d, *J* = 6.7 Hz, 3H), 0.84 (d, *J* = 6.7 Hz, 3H).

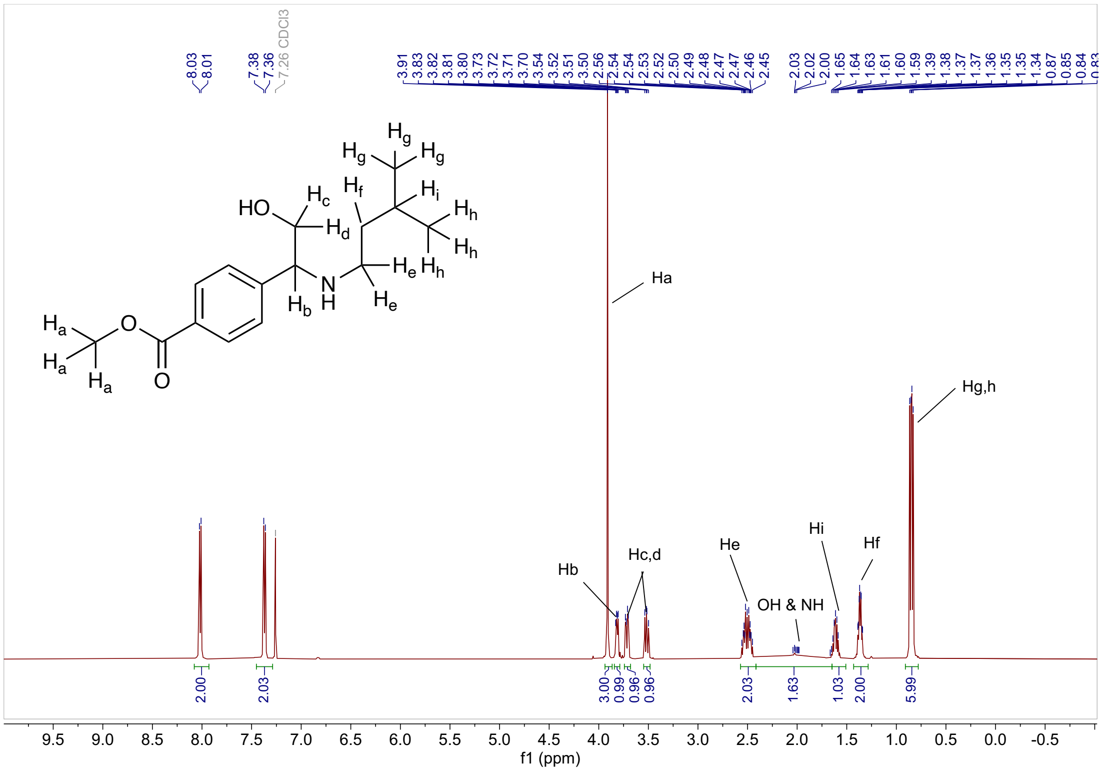

<u>NMR characterization</u>: The structure assignment is further supported by COSY, Attached proton test (APT) and HSQC.

COSY

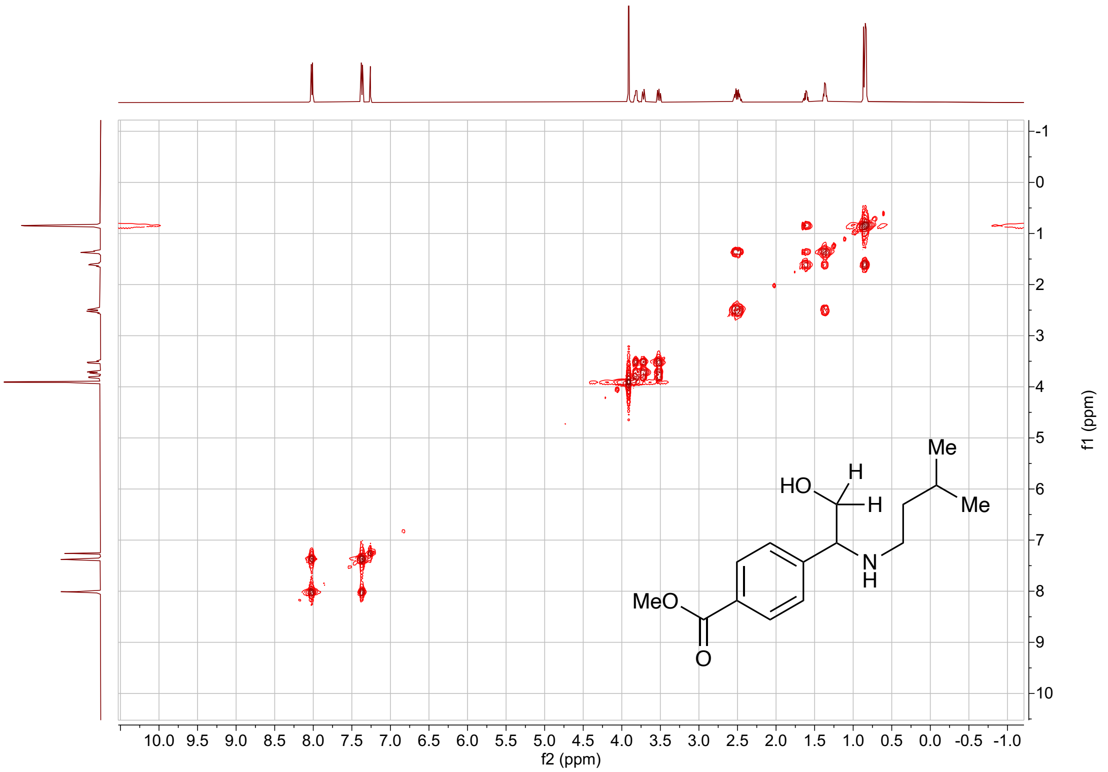

Attached proton test

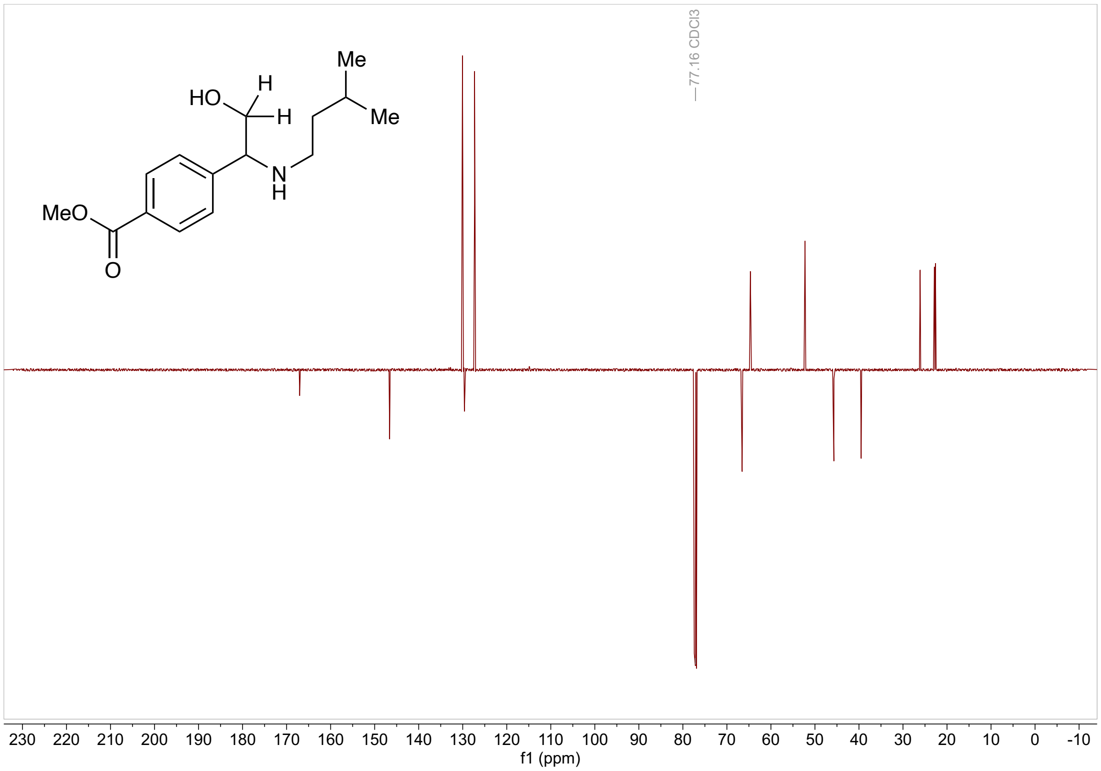

HSQC

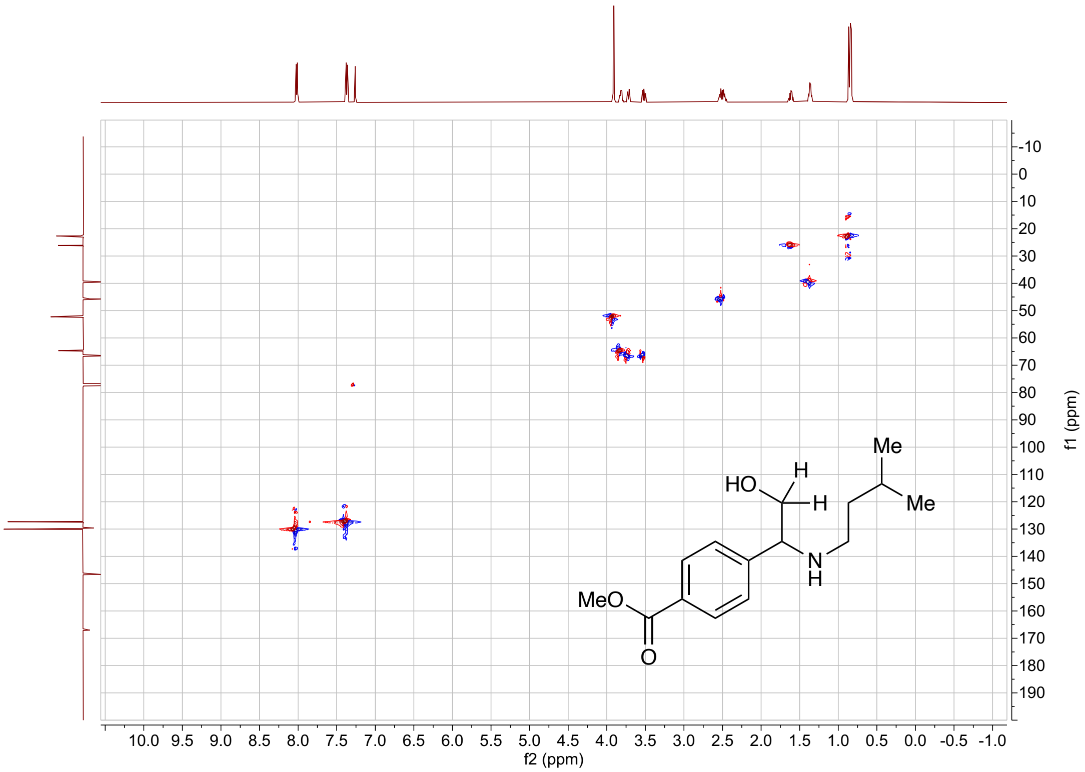

IR characterization: the presence of OH is further supported by IR
IR (neat) ν_{max} 3353 (O–H stretch), 3299, 2953, 2869, 1721, 1611, 1576, 1436, 1367, 1276, 1042, 1018, 966, 858, 771.

HRMS (ESI-TOF) *m/z* calcd. for $C_{15}H_{24}NO_3^+$ ($[M+H]^+$) 266.1751, found 266.1752.

Part II: structure assignment to deuterium incorporated product
Reaction vial: *In the glovebox*, an oven-dried 40-mL vial was charged methyl (*E*)-4-((isopentylimino)methyl)benzoate (58.3 mg, 0.25 mmol), $FeCl_3$ (8.1 mg, 0.05 mmol, 20 mol%), bis(4-methoxyphenyl) disulfide (20.9 mg, 0.075 mmol, 30 mol%) and *d*-benzenesulfonic acid (159.2 mg, 1.0 mmol, 4 equiv, 62% deuteration). Then, 1.25 mL of anhydrous CD_3CN was added to dissolve the mixture. The reaction mixture was brought out from the glovebox and sealed with parafilm. The reaction was then irradiated under 365 nm light with 100% light intensity for 12 hours.

After working up according to **General Procedure A-2**, the basified, dry organic mixture was subjected to further purification by preparative reverse phase HPLC with 45% to 55% MeCN in water (0.1% NH_4OH modifier). Product containing fractions were concentrated to afford desired product as a clear oil.

Percentage of deuterium incorporation: Based on the NMR analysis, various C–H bonds obtained deuterium incorporation. In particular, tertiary C–H bond has highest deuterium incarnation of 24%. C–H bonds of *gem*-Dimethyl group also have 10% deuterium incorporation. Additionally, non-α-amino secondary C–H bonds were also detected with 6% deuterium incorporation. Lastly, due to the protonation, α-amino C–H bonds have no deuterium incorporation, which is consistent with our mechanistic design and proposal.

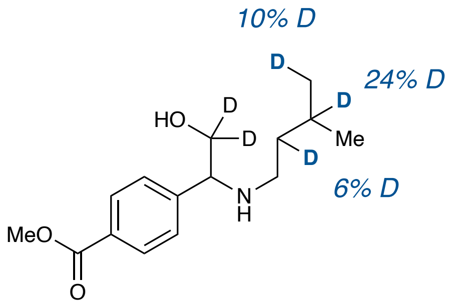

NMR characterization: **^{1}H NMR (500 MHz, $CDCl_3$)** δ 8.02 (d, J = 7.9 Hz, 2H), 7.37 (d, J = 7.9 Hz, 2H), 3.91 (s, 3H), 3.81 (s, 1H), 2.67 – 2.38 (m, 2H), 2.08 (s, 1H), 1.67 – 1.55 (m, J = 6.8 Hz, 0.76H), 1.36 (hept, J = 7.0 Hz, 1.89H), [0.86 (d, J = 6.7 Hz), 0.83 (d, J = 6.7 Hz), 5.43H].

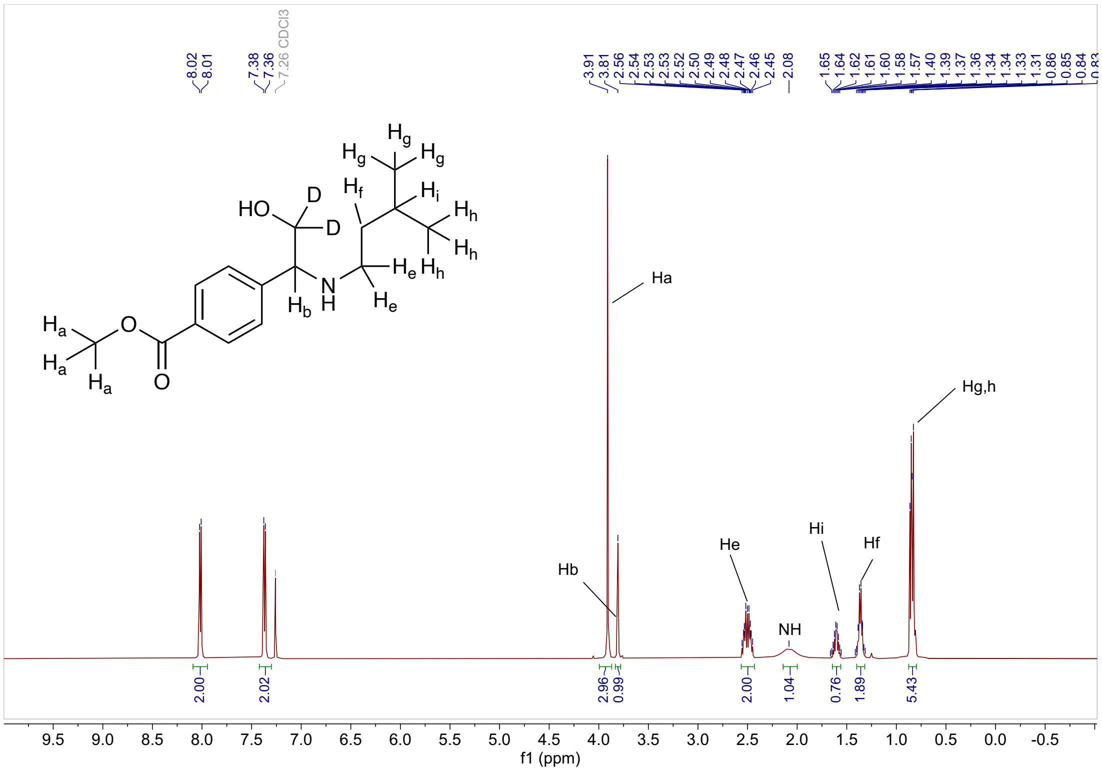

NMR characterization: The structure assignment is further supported by COSY, Attached proton test (APT) and HSQC.

COSY

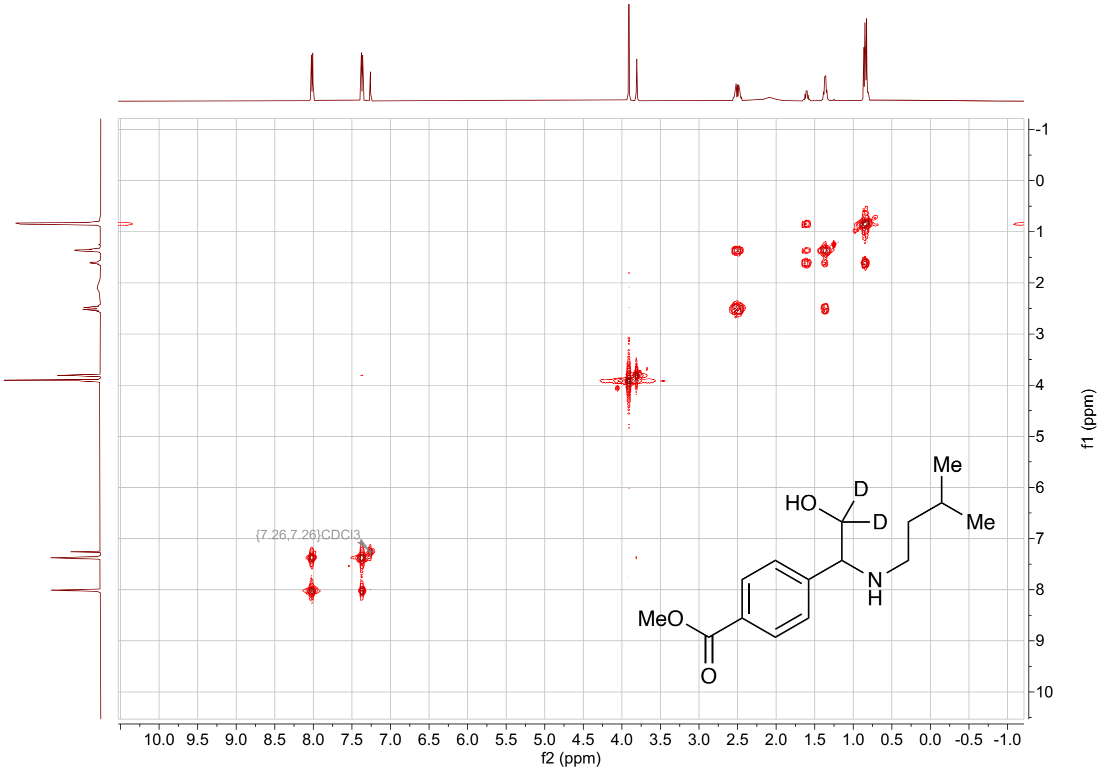

Attached proton test

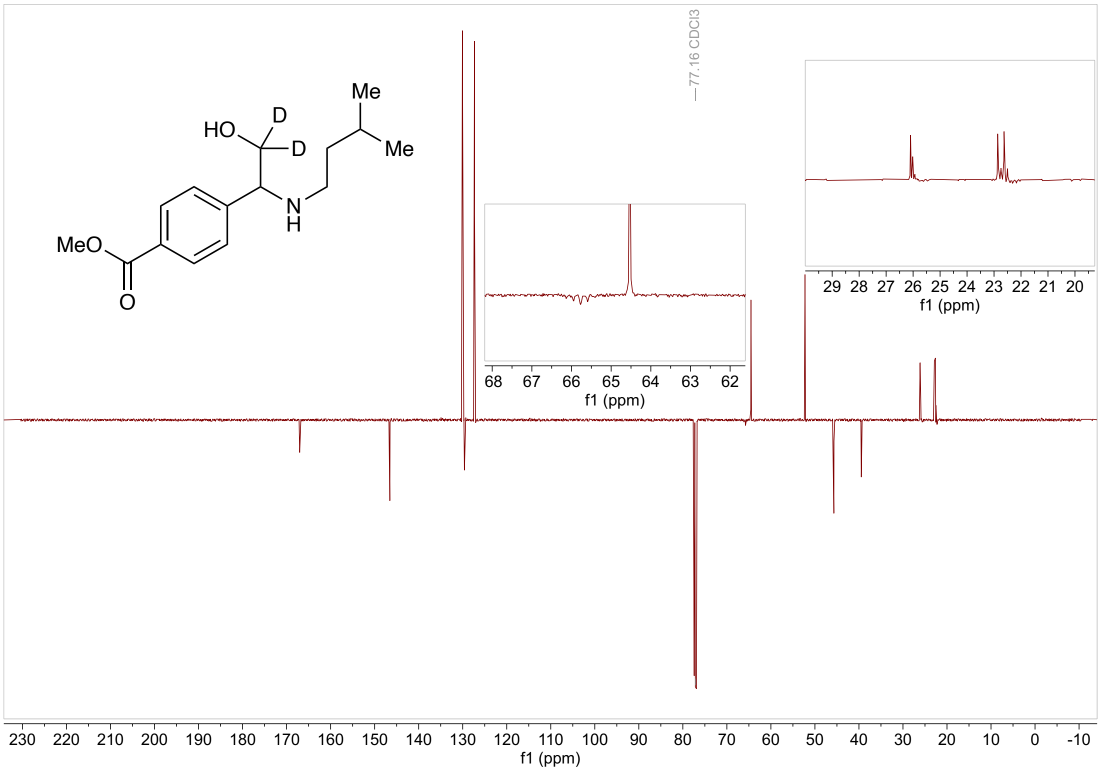

HSQC

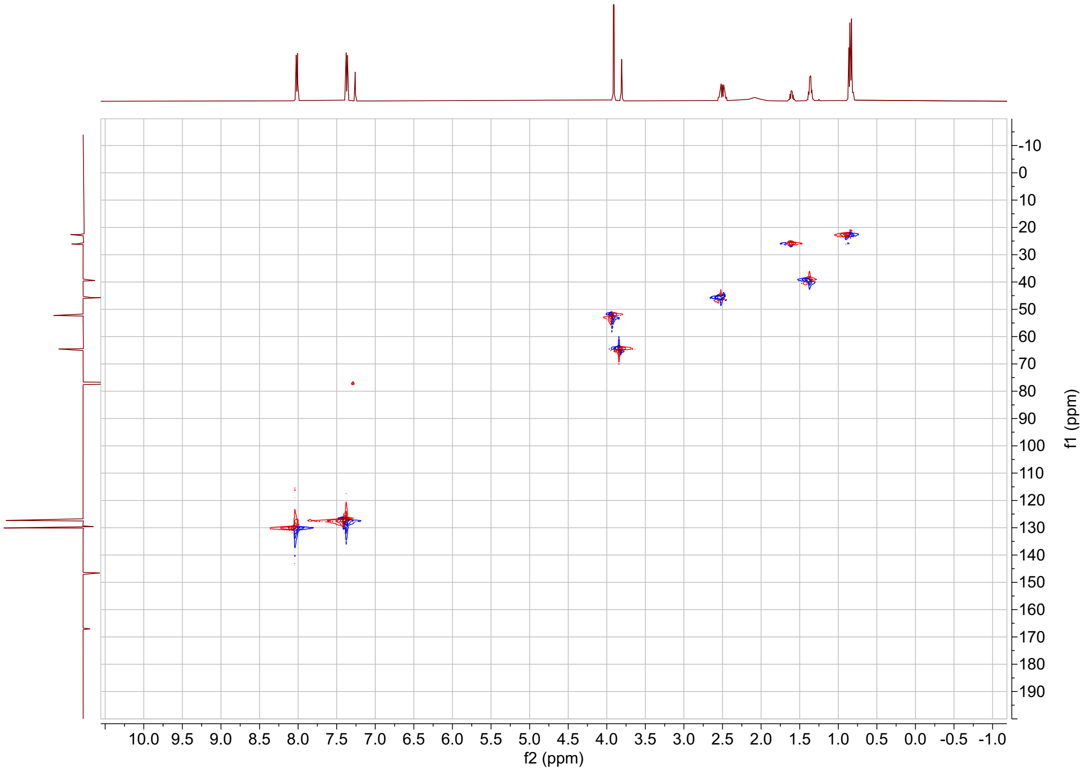

<u>IR characterization:</u> the presence of OH is further supported by IR

IR (neat) ν_{max} 3317 (O–H stretch), 3281, 2952, 2868, 1721, 1610, 1576, 1435, 1416, 1275, 1191, 1109, 1019, 967, 856, 826, 707.

HRMS (ESI-TOF)

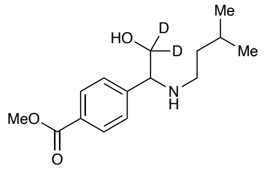

m/z calcd for $C_{15}H_{22}D_2NO_3^+$ [M+H]$^+$ 268.1876, found 268.1874

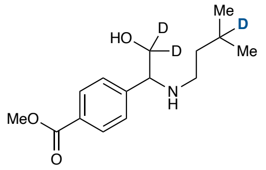

m/z calcd for $C_{15}H_{21}D_3NO_3^+$ [M+H]$^+$ 269.1939, found 269.1933

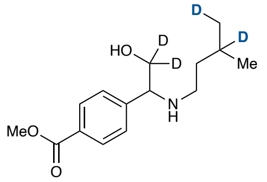

m/z calcd for $C_{15}H_{20}D_4NO_3^+$ [M+H]$^+$ 270.2002, found 270.1998

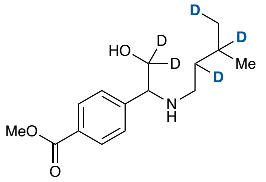

m/z calcd for $C_{15}H_{19}D_5NO_3^+$ [M+H]$^+$ 271.2065, found 271.2059

Part III: Before and after deuterium incorporation

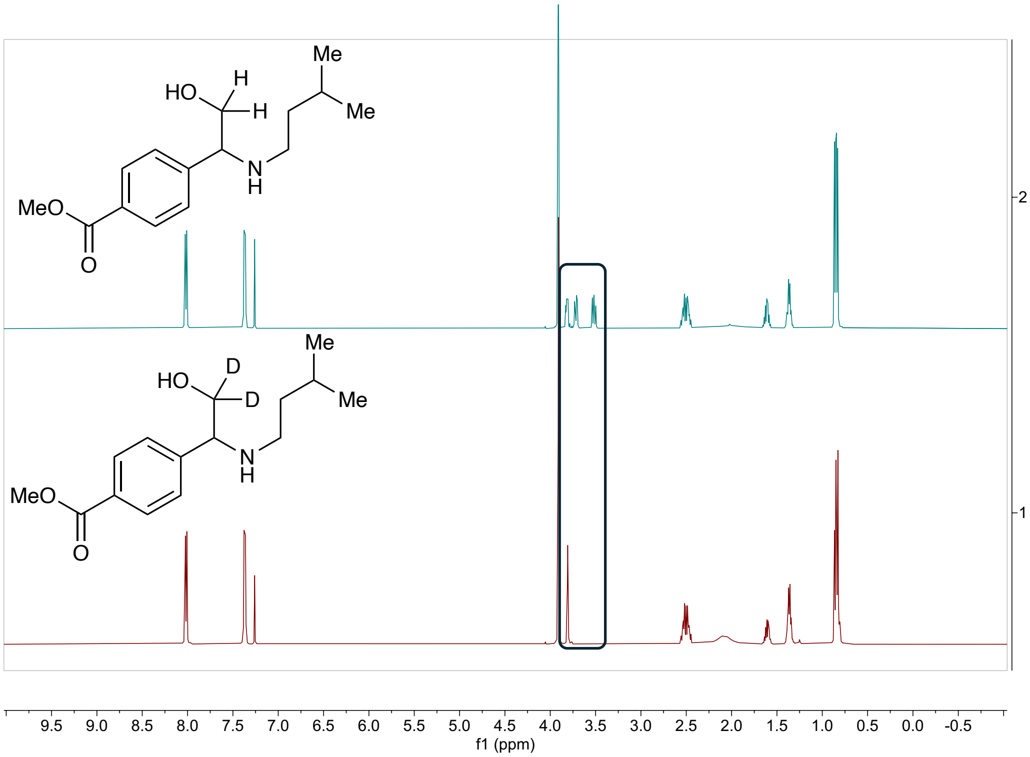

16) Supplemental substrate scopes

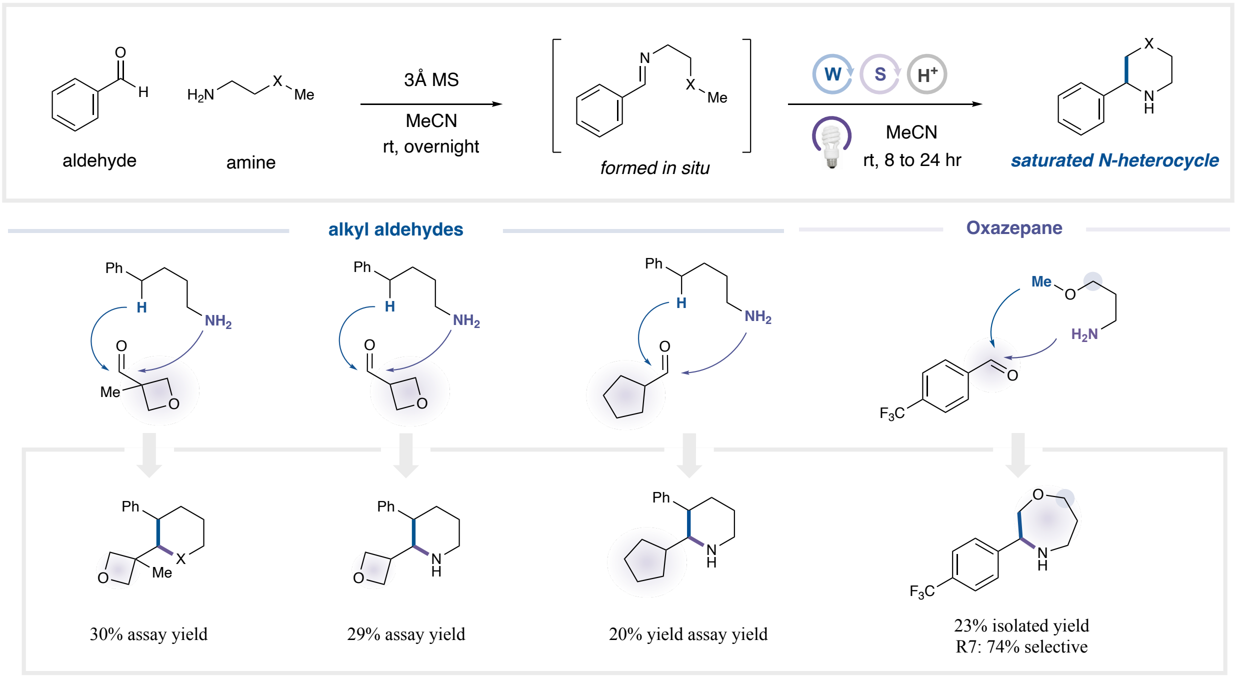

Figure S15. Supplemental substrate scopes.

16) References

1. Perrin, D. D.; Armarego, W. L. F. *Purification of Laboratory Chemicals*, 3rd ed.; Pergamon Press, 1988.
2. Pangborn, A. B.; Giardello, M. A.; Grubbs, R. H.; Rosen, R. K.; Timmers, F. J. Safe and Convenient Procedure for Solvent Purification. *Organometallics* **1996**, *15*, 1518–1520.
3. Le, C. C.; Wismer, M. K.; Shi, Z.-C.; Zhang, R.; Conway, D. V.; Li, G.; Vachal, P.; Davies, I. W.; MacMillan, D. W. C. A General Small-Scale Reactor To Enable Standardization and Acceleration of Photocatalytic Reactions. *ACS Cent. Sci.* **2017**, *3*, 647–653. DOI: 10.1021/acscentsci.7b00159
4. Still, W. C.; Kahn, M.; Mitra, A. Rapid Chromatographic Technique for Preparative Separations with Moderate Resolution. *J. Org. Chem.* **1978**, *43*, 2923–2925.
5. Kelly, C. B.; Patel, N. R.; Primer, D. N.; Jouffroy, M.; Tellis, J. C.; Molander, G. A. Preparation of Visible-Light Activated Metal Complexes and Their Use in Photoredox/Nickel Dual Catalysis. *Nat. Protoc.* **2017**, *12*, 472–492. DOI: 10.1038/nprot.2016.176
6. Frisch, M. J.; Trucks, G. W.; Schlegel, H. B.; Scuseria, G. E.; Robb, M. A.; Cheeseman, J. R.; Scalmani, G.; Barone, V.; Mennucci, B.; Petersson, G. A.; Nakatsuji, H.; Caricato, M.; Li, X.; Hratchian, H. P.; Izmaylov, A. F.; Bloino, J.; Zheng, G.; Sonnenberg, J. L.; Hada, M.; Ehara, M.; Toyota, K.; Fukuda, R.; Hasegawa, J.; Ishida, M.; Nakajima, T.; Honda, Y.; Kitao, O.; Nakai, H.; Vreven, T.; Montgomery, J. A.; Peralta, J. E.; Ogliaro, F.; Bearpark, M.; Heyd, J. J.; Brothers, E.; Kudin, K. N.; Staroverov, V. N.; Kobayashi, R.; Normand, J.; Raghavachari, K.; Rendell, A.; Burant, J. C.; Iyengar, S. S.; Tomasi, J.; Cossi, M.; Rega, N.; Millam, J. M.; Klene, M.; Knox, J. E.; Cross, J. B.; Bakken, V.; Adamo, C.; Jaramillo, J.; Gomperts, R.; Stratmann, R. E.; Yazyev, O.; Austin, A. J.; Cammi, R.; Pomelli, C.; Ochterski, J. W.; Martin, R. L.; Morokuma, K.; Zakrzewski, V. G.; Voth, G. A.; Salvador, P.; Dannenberg, J. J.; Dapprich, S.; Daniels, A. D.; Farkas; Foresman, J. B.; Ortiz, J. V.; Cioslowski, J.; Fox, D. J. Gaussian 09; Gaussian Inc.: Wallingford, CT, **2009**.
7. Zhao, Y.; Truhlar, D. G. The M06 suite of density functionals for main group thermochemistry, thermochemical kinetics, noncovalent interactions, excited states, and transition elements: two new functionals and systematic testing of four M06-class functionals and 12 other functionals. *Theor. Chem. Acc.* **2008**, *120* (1), 215-241. DOI: 10.1007/s00214-007-0310-x.
8. Weigend, F. Accurate Coulomb-fitting basis sets for H to Rn. *Physical Chemistry Chemical Physics* **2006**, *8* (9), 1057-1065, 10.1039/B515623H. DOI: 10.1039/B515623H.
9. Marenich, A. V.; Cramer, C. J.; Truhlar, D. G. Universal Solvation Model Based on Solute Electron Density and on a Continuum Model of the Solvent Defined by the Bulk Dielectric Constant and Atomic Surface Tensions. *J. Chem. Phys. B.* **2009**, *113* (18), 6378-6396. DOI: 10.1021/jp810292n.
10. Luo, Y.-R. *Comprehensive Handbook of Chemical Bond Energies*, 1st ed. CRC Press, **2007.**

11. Martin Newcomb, S.-Y. C., and John H. Horner. Adjusting the Top End of the Alkyl Radical Kinetic Scale. Laser Flash Photolysis Calibrations of Fast Radical Clocks and Rate Constants for Reactions of Benzeneselenol. *J. Org. Chem.* **1999**, *64*, 1225-1231.
12. Ravelli, D.; Fagnoni, M.; Fukuyama, T.; Nishikawa, T.; Ryu, I. Site-Selective C–H Functionalization by Decatungstate Anion Photocatalysis: Synergistic Control by Polar and Steric Effects Expands the Reaction Scope. *ACS Catal.* **2017**, *8* (1), 701-713. DOI: 10.1021/acscatal.7b03354.
13. Renneke, R. F.; Pasquali, M.; Hill, C. L. Polyoxometalate systems for the catalytic selective production of nonthermodynamic alkenes from alkanes. Nature of excited-state deactivation processes and control of subsequent thermal processes in polyoxometalate photoredox chemistry. *J. Am. Chem. Soc.* **1990**, *112* (18), 6585-6594.
14. Waele, V. D.; Poizat, O.; Fagnoni, M.; Bagno, A.; Ravelli, D. Unraveling the Key Features of the Reactive State of Decatungstate Anion in Hydrogen Atom Transfer (HAT) Photocatalysis. *ACS Catal.* **2016**, *6* (10), 7174-7182. DOI: 10.1021/acscatal.6b01984.
15. Ni, H.; Li, C.; Shi, X.; Hu, X.; Mao, H. Visible-Light-Promoted Fe(III)-Catalyzed N-H Alkylation of Amides and N-Heterocycles. *J Org Chem* **2022**, *87* (15), 9797-9805. DOI: 10.1021/acs.joc.2c00854.
16. Luescher, M. U.; Vo, C. V. T.; Bode, J. W. *Org. Lett.* **2014**, *16* (4), 1236-1239.
17. Hsieh, S. Y.; Bode, J. W. *ACS Cent. Sci.* **2017**, *3* (1), 66-72.
18. Bissonnette, N. B.; Ellis, J. M.; Hamann, L. G.; Romanov-Michailidis, F. *Chem. Sci.* **2019**, *10*, 9591-9596.
19. Zhang, Y. A.; Palani, V.; Seim, A. E.; Wang, K. J.; Wendlandt, A. E. *Science* **2022**, *378* (6618), 383-390.
20. Mao, E.; MacMillan, D. W. C. *J. Am. Chem. Soc.* **2023**, *145* (5), 2787-2793.

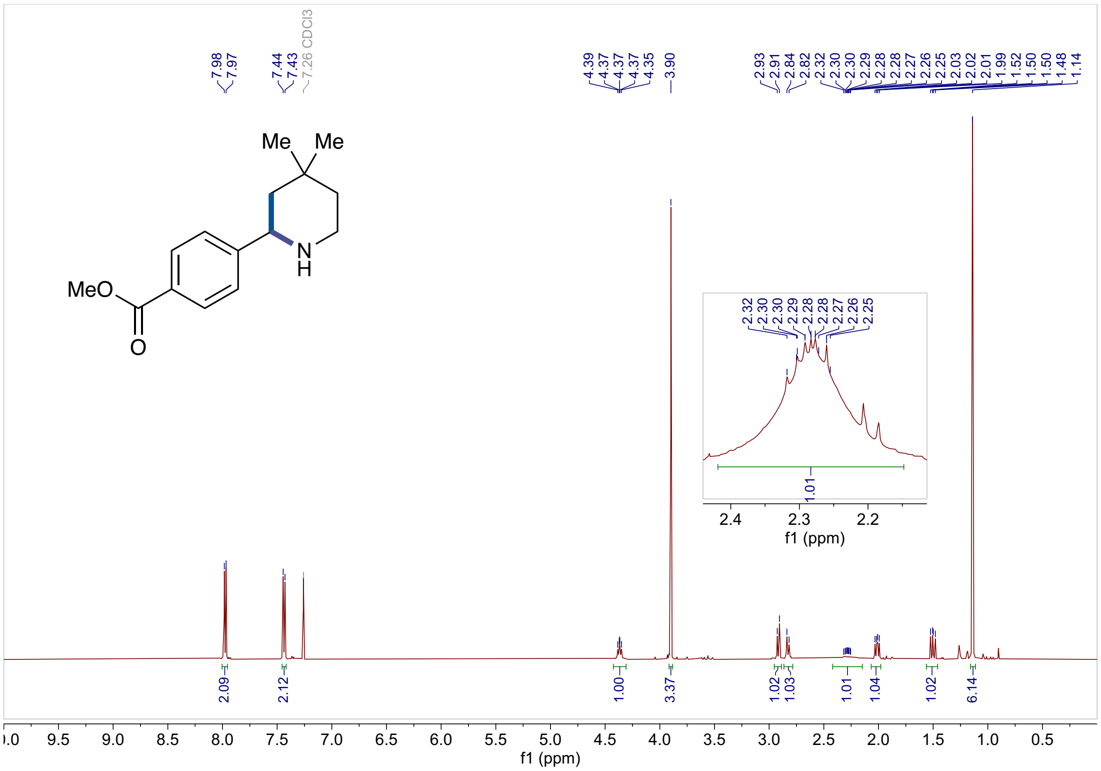

3 – ^{1}H NMR (500 MHz, $CDCl_3$)

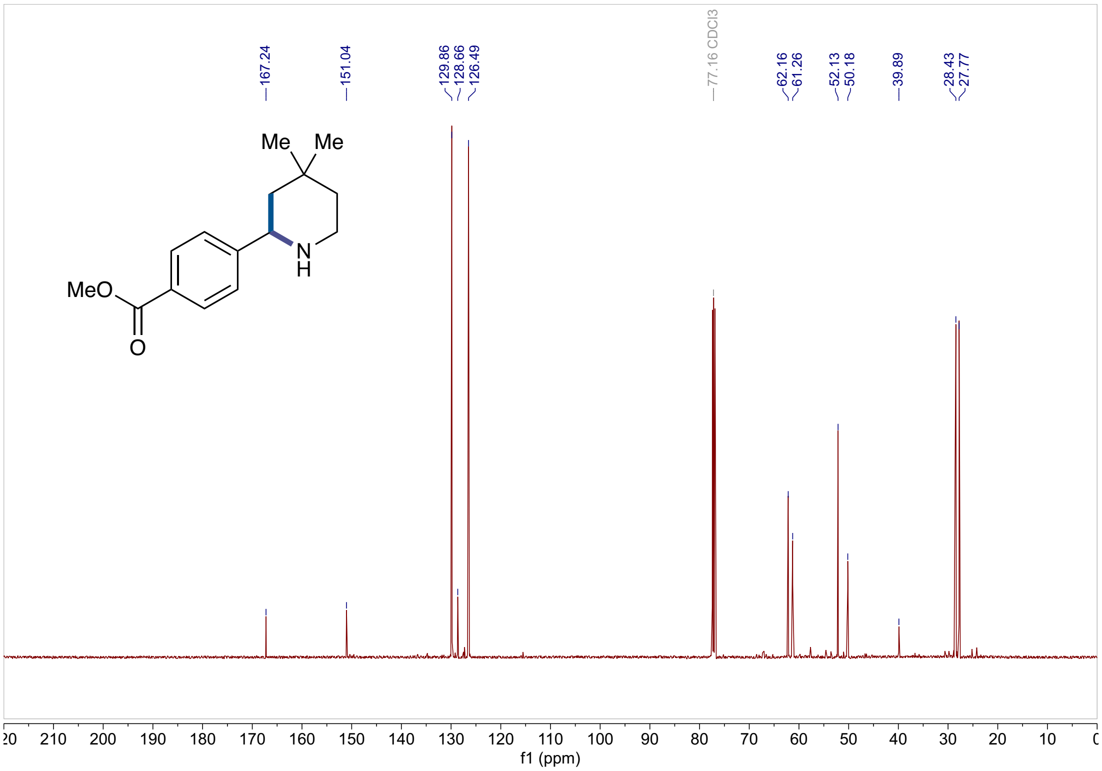

3 – ^{13}C NMR (126 MHz, $CDCl_3$)

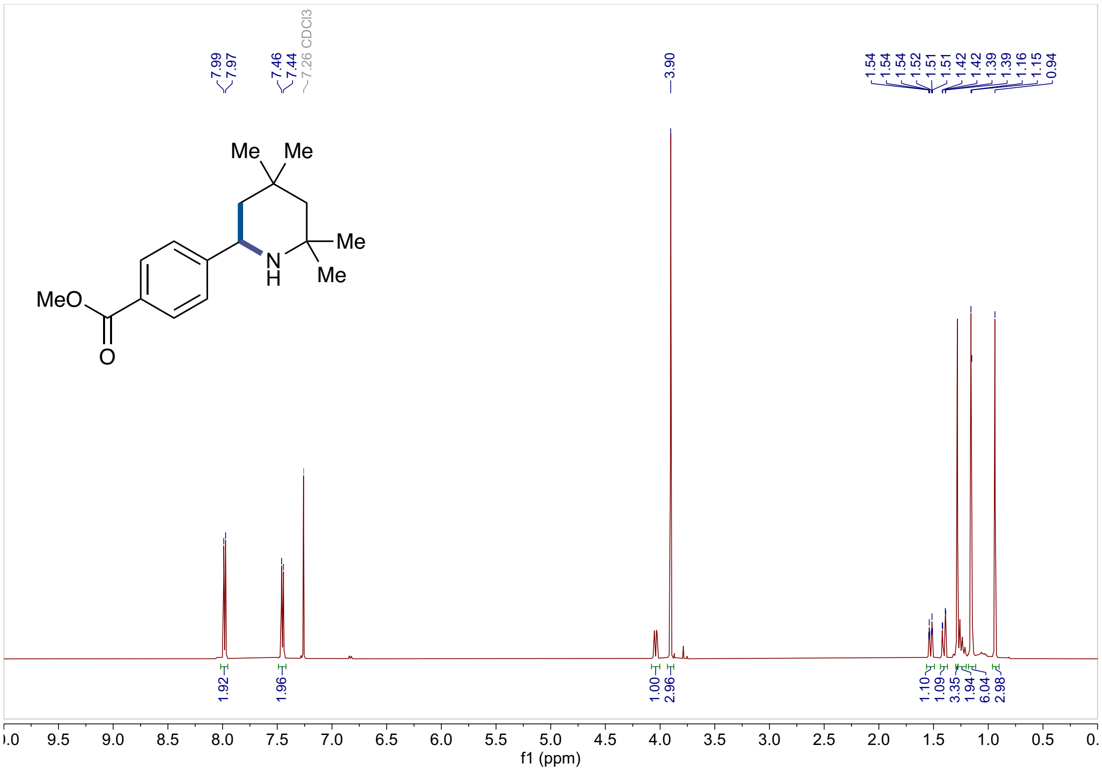

4 – ^{1}H NMR (500 MHz, $CDCl_3$)

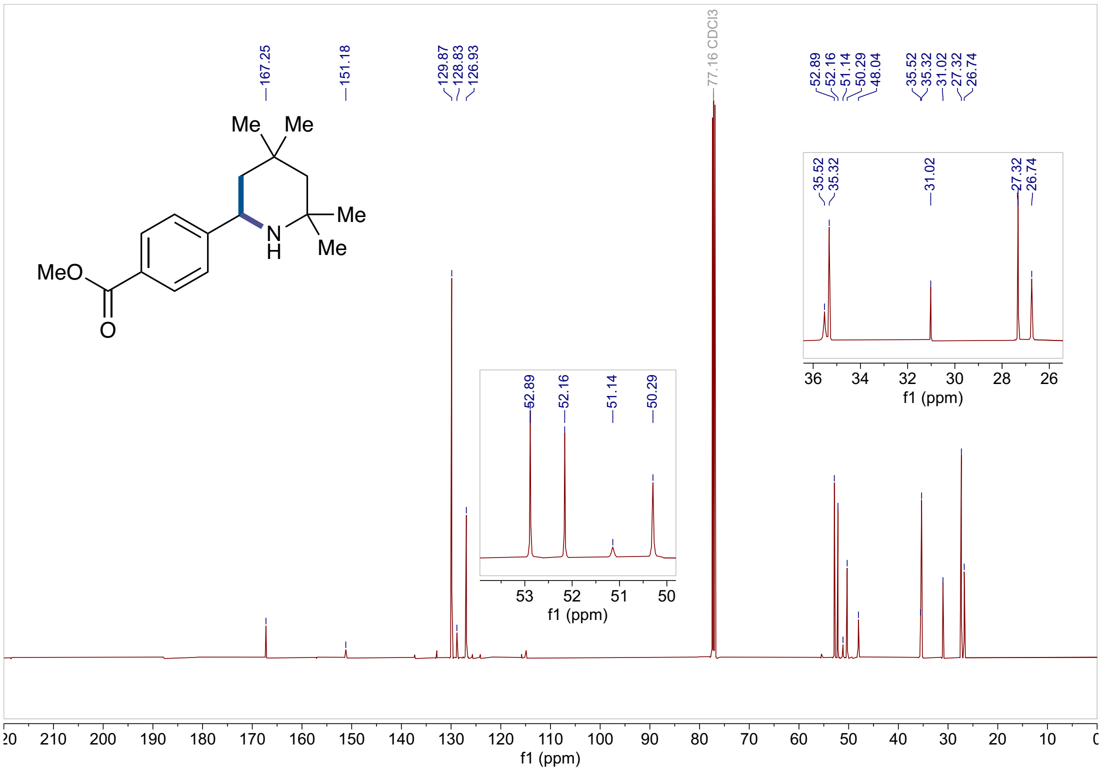

4 – ^{13}C NMR (126 MHz, $CDCl_3$)

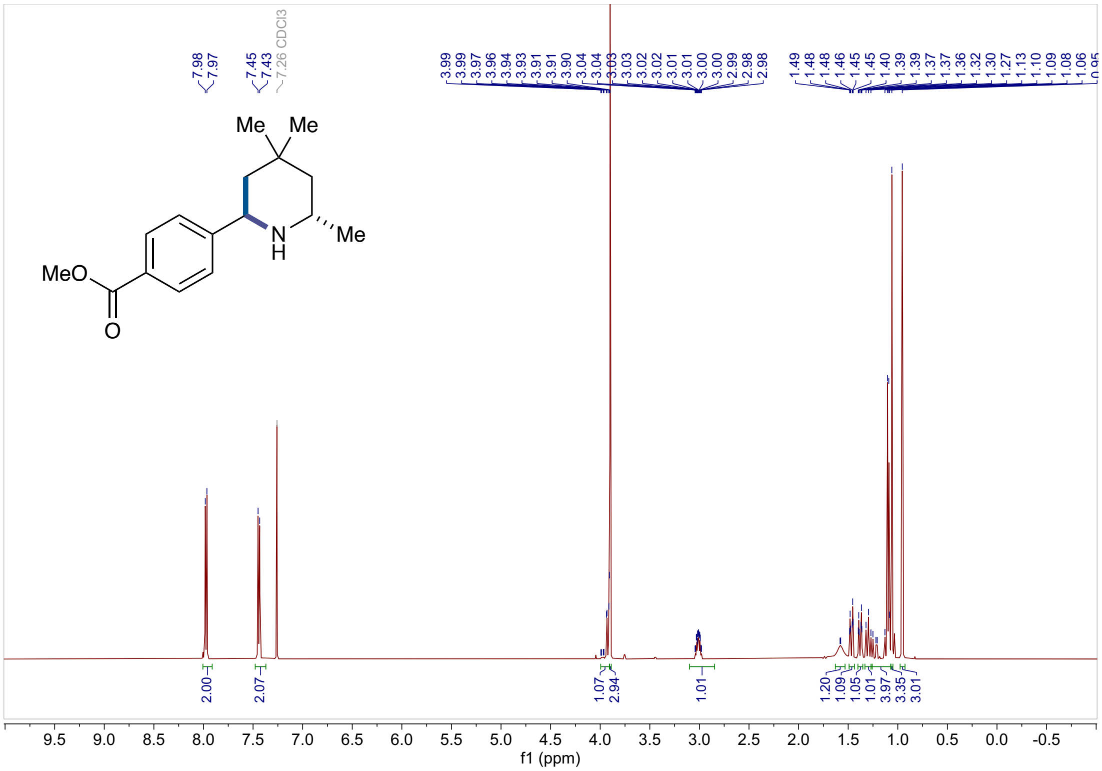

5 – ^{1}H NMR (500 MHz, CDCl$_3$)

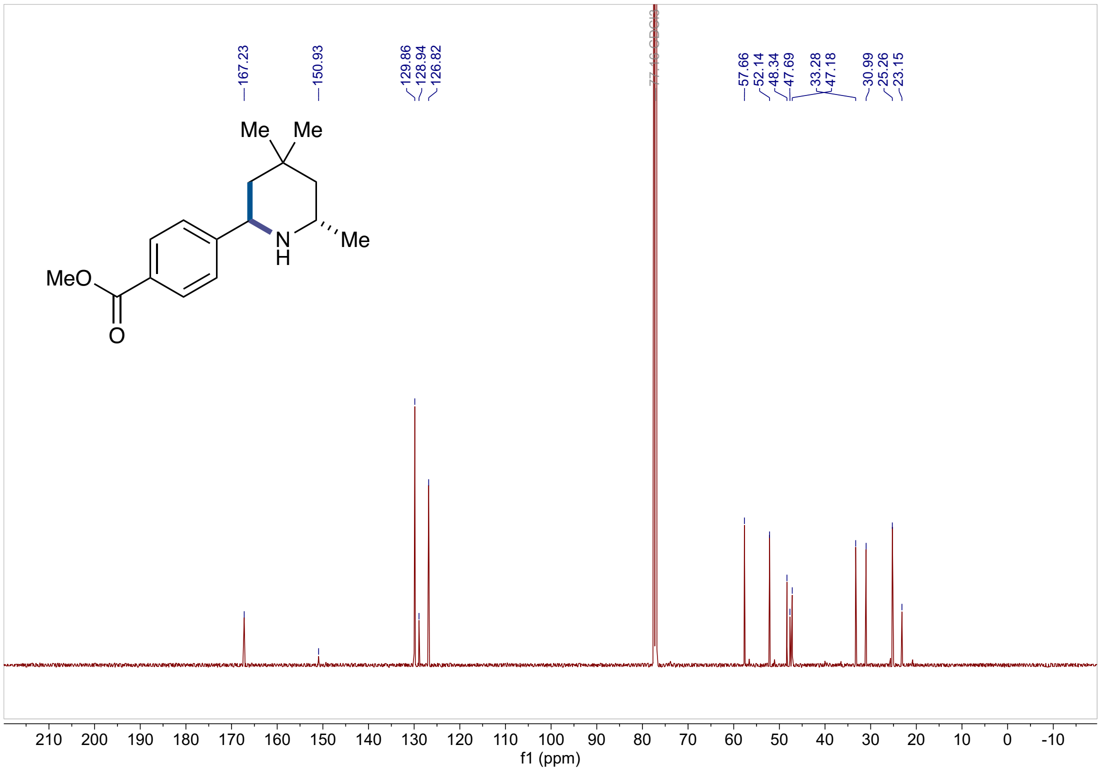

5 – ^{13}C NMR (126 MHz, CDCl$_3$)

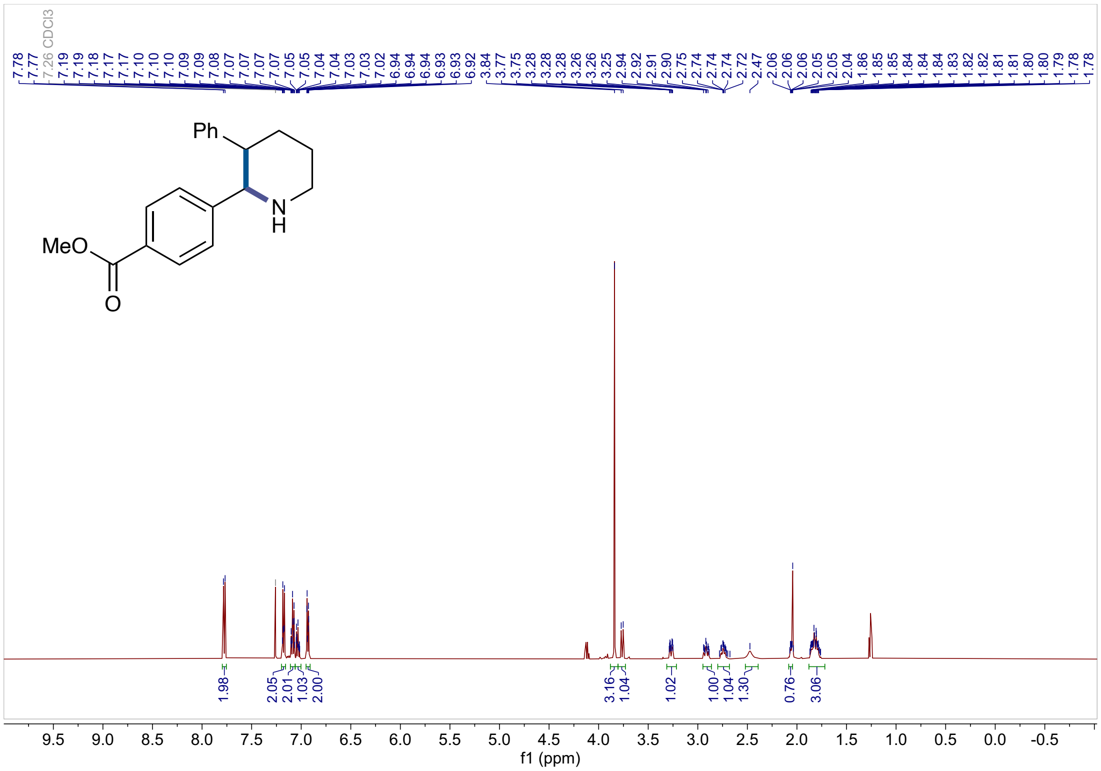

6 – ^{1}H NMR (500 MHz, $CDCl_3$)

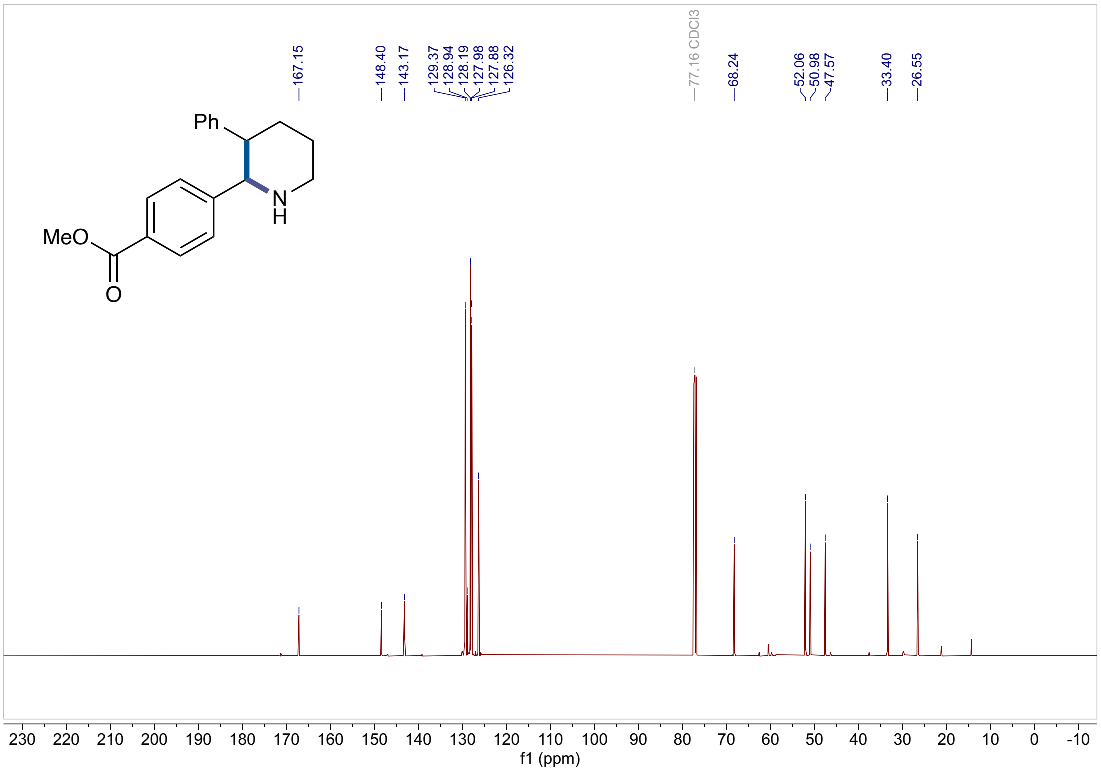

6 – ^{13}C NMR (126 MHz, $CDCl_3$)

Compound **6** – uHPLC analysis for d.r. determination

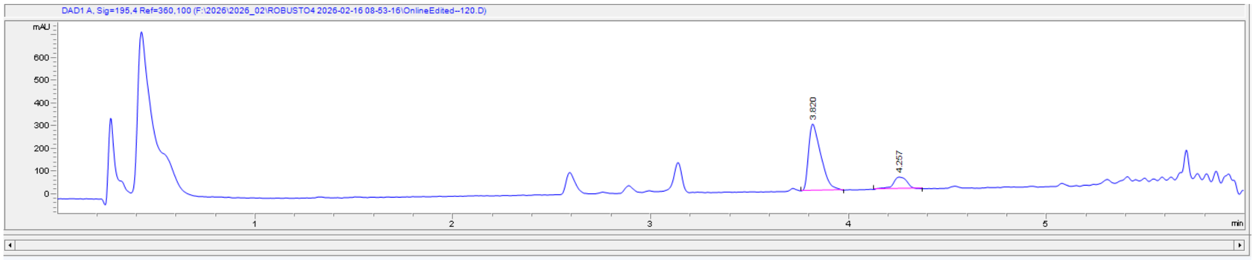

#	Time	Type	Area	Height	Width	Area%	Symmetry
1	3.82	BB	1281.6	294.6	0.0628	82.665	0.481
2	4.257	BB	268.7	52.9	0.0813	17.335	0.876

Compound **6** – COSY analysis for major diastereomer determination

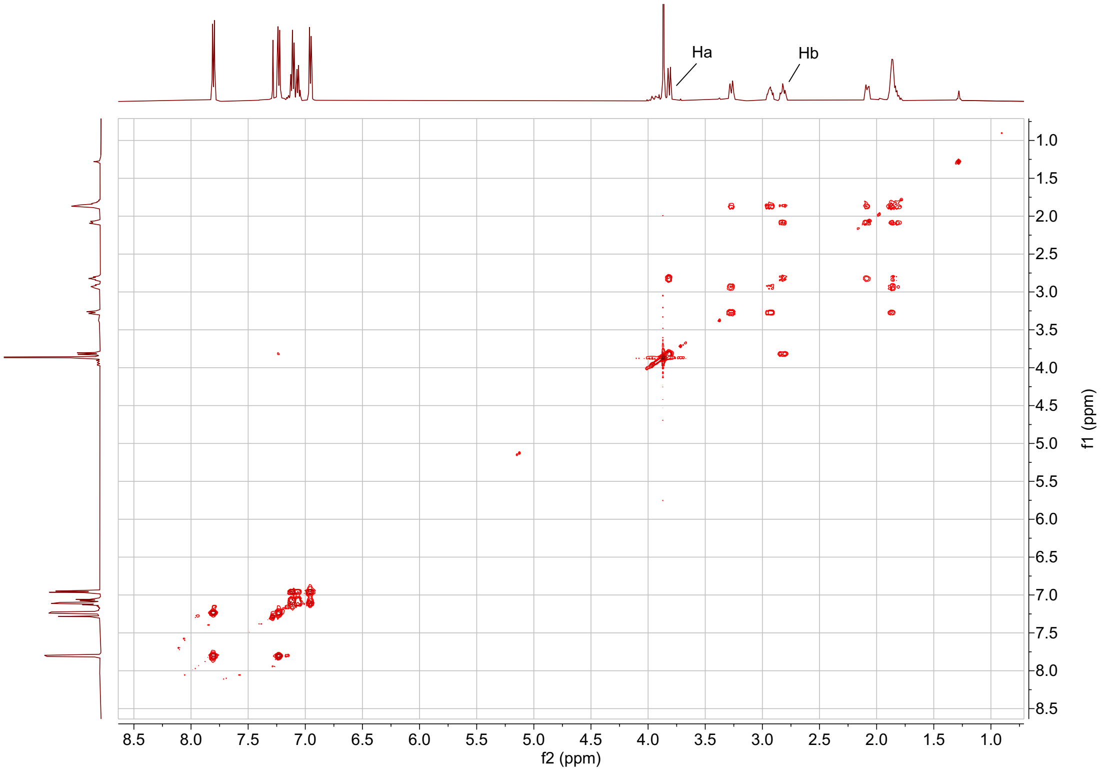

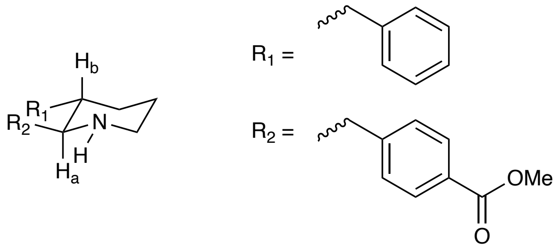

^{1}H NMR (500 MHz, CDCl3) and COSY

δ 3.81 H_a (d, 10.2 Hz)

δ 2.82 H_b (td, 10.6, 3.8 Hz)

based on *J*-coupling of H_a and H_b, major diastereomer is *trans*

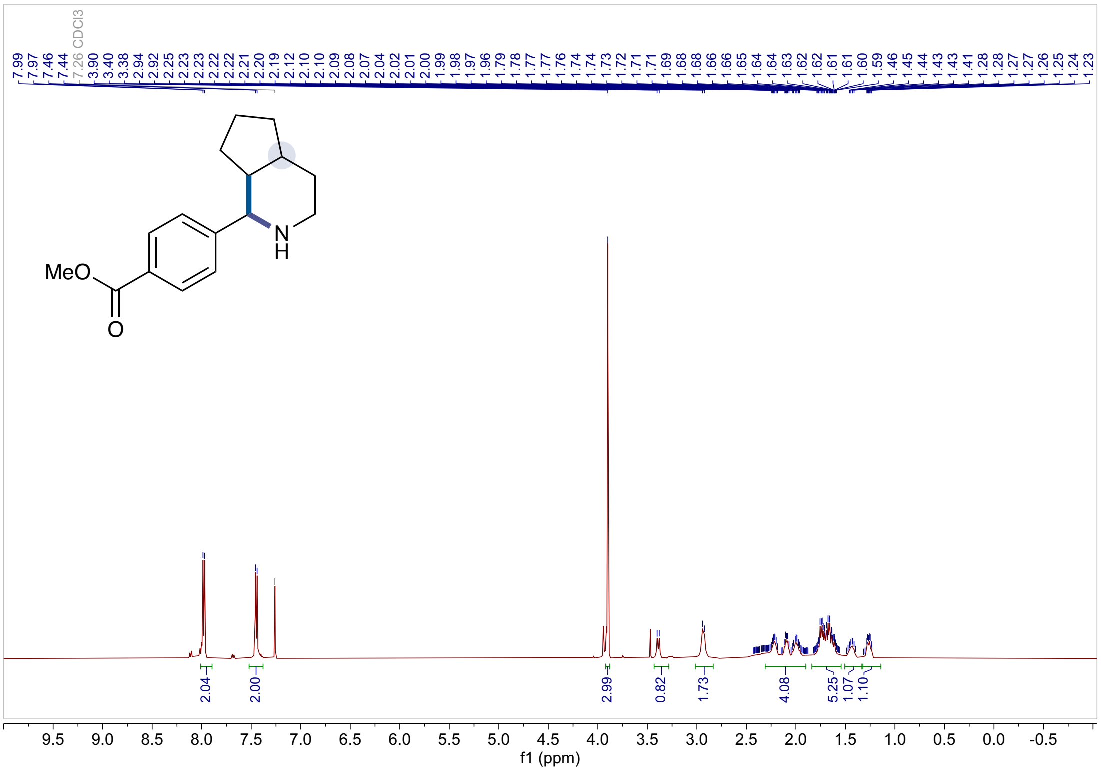

7 – ^{1}H NMR (500 MHz, $CDCl_3$)

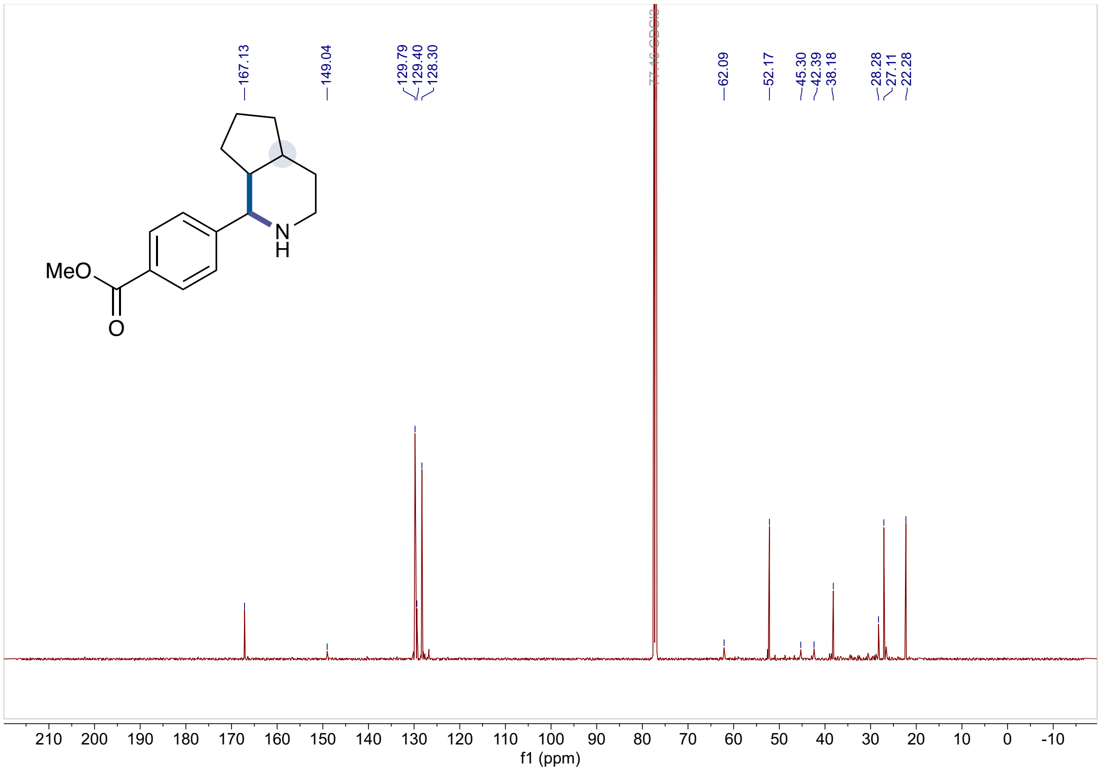

7 – ^{13}C NMR (126 MHz, $CDCl_3$)

Substrate #	Point #	Wavelength	STD retention time	STD absorption	Pdt retention time	Product absorption	pdt/std absorption ration	NMR Pdt int	NMR STD int (4H)	molar ratio (STD/pdt)
	1			311.2		97.477	0.313229434	2.41	4	1.205
7	2	250 nm	3.476 min	171.7		87.7	0.510774607	4	4	2
	3			197		76.9	0.39035533	3.11	4	1.555

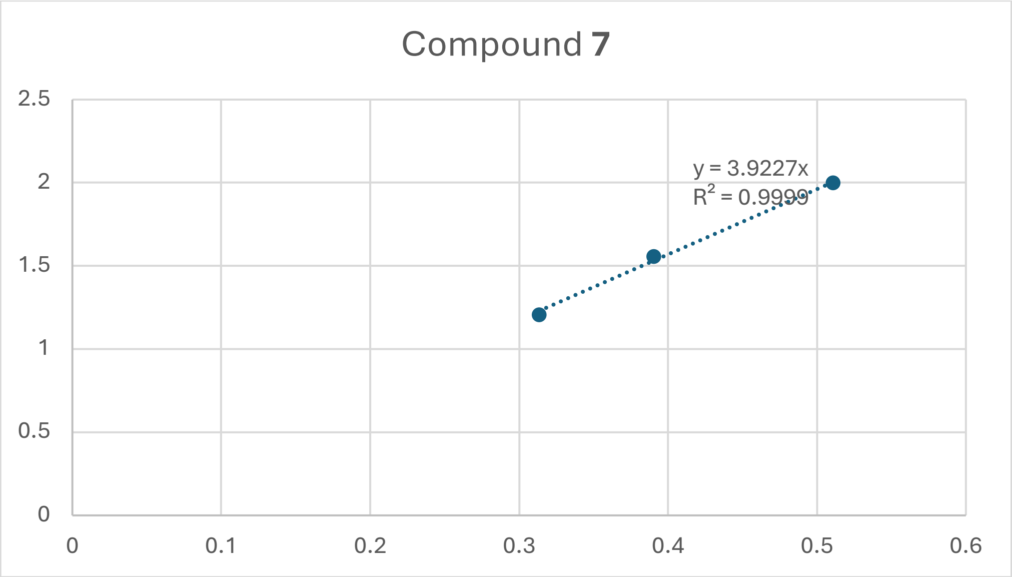

Compound **7** – calibration curve traces (made with isolated product [NMR shown on **page 132**])

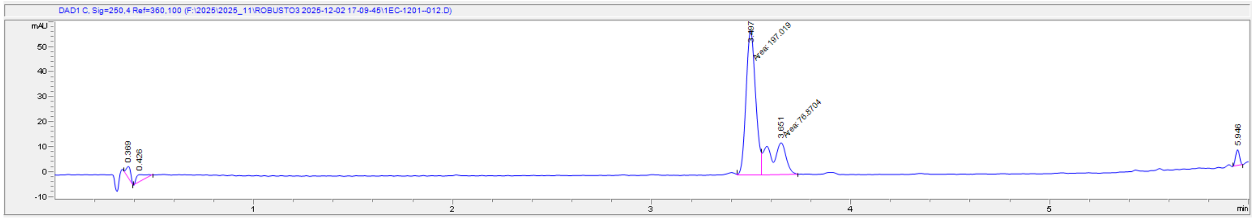

Based on uHPLC calibration curve traces, the isolatable and identifiable pure product [demonstrated by NMRs on page 132] of compound **7** contains a mixture of two diastereomers of six-membered cyclized products. Therefore, to be consistent and avoid overestimation of the yields due to the coelution of the products on the uHPLC, the assay yields only accounted for the isolatable and identifiable six-membered cyclized products.

Compound **7** - uHPLC crude reaction traces

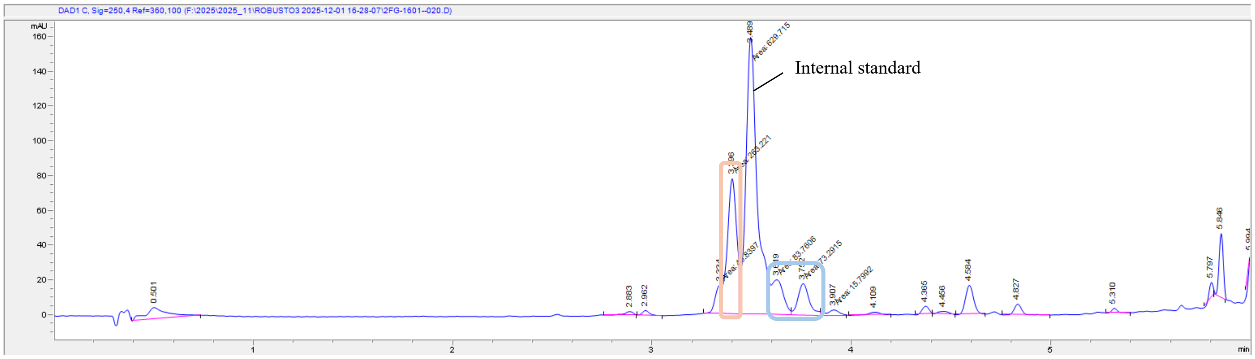

The two peaks highlighted in blue correspond to isolable and identifiable six-membered cyclized products. For rigorous peak assignment, only these blue-highlighted peaks were used to calculate the assay yield. However, the peak highlighted in orange contains a substantial amount of cyclized products. Because of coelution, the area underneath was overlapped, and it could not be definitively assigned as either a six- or five-membered cyclized product. As a result, by only accounting for the peaks highlighted in blue, the reported overall assay yields might be underestimated.

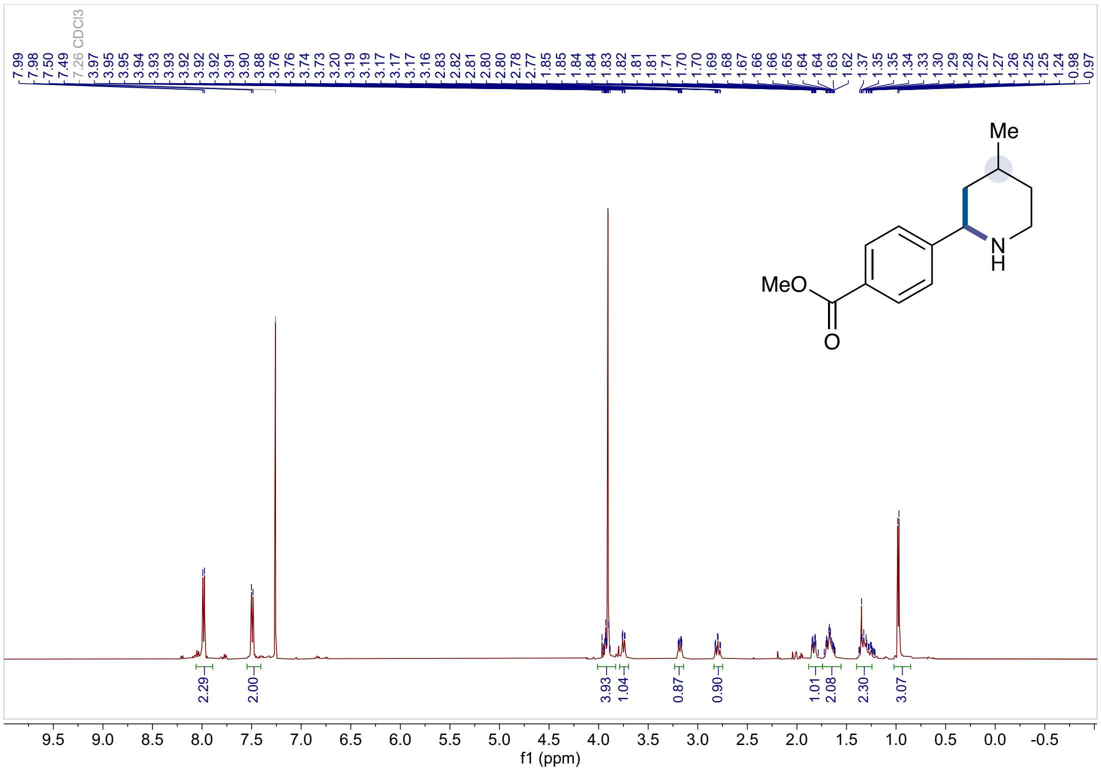

8 – ^{1}H NMR (500 MHz, $CDCl_3$)

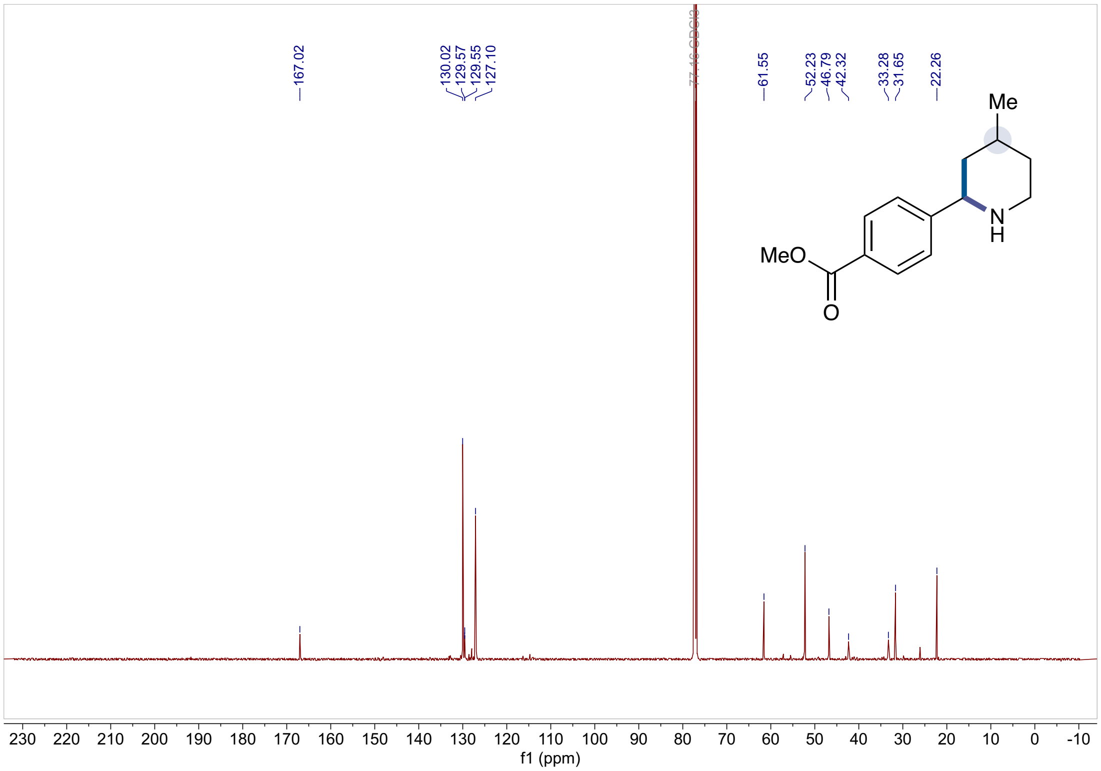

8 – ^{13}C NMR (126 MHz, $CDCl_3$)

Calibration curve – **8**

Substrate #	Point #	Wavelength	STD retention t	STD absorption	Pdt retention time	Product absorption	pdt/std absorption ration	NMR Pdt int	NMR STD int (4	molar ratio (STD/pdt)
	1			995.1		351.8	0.353532308	0.77	4	0.385
8	2	195 nm	3.480 min	961	3.017 min	589.1	0.613007284	1.28	4	0.64
	3			829.3		400.9	0.483419752	0.97	4	0.485

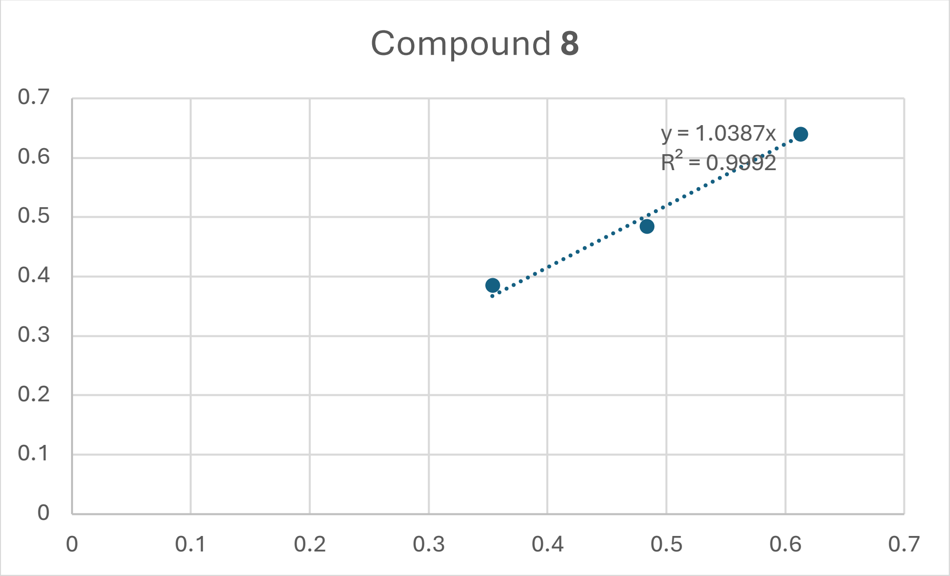

Compound **8** - uHPLC crude reaction traces

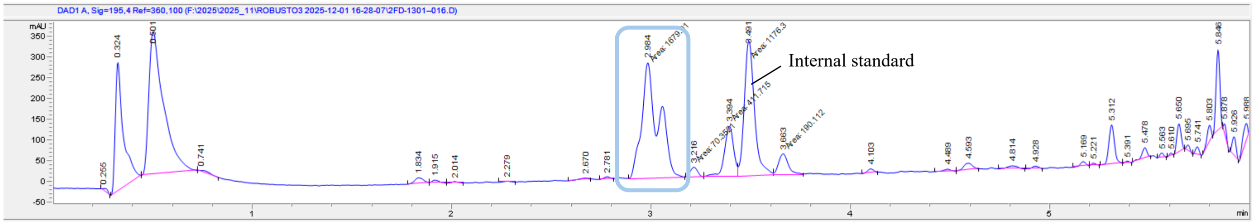

The highlighted two peaks correspond to the two diastereomers of the six-membered cyclized products. The minor regioisomer, five-membered cyclized product is barely observed. The uHPLC crude traces are consistent with the isolation results that the isolatable products are two diastereomers of the six-membered cyclized products.

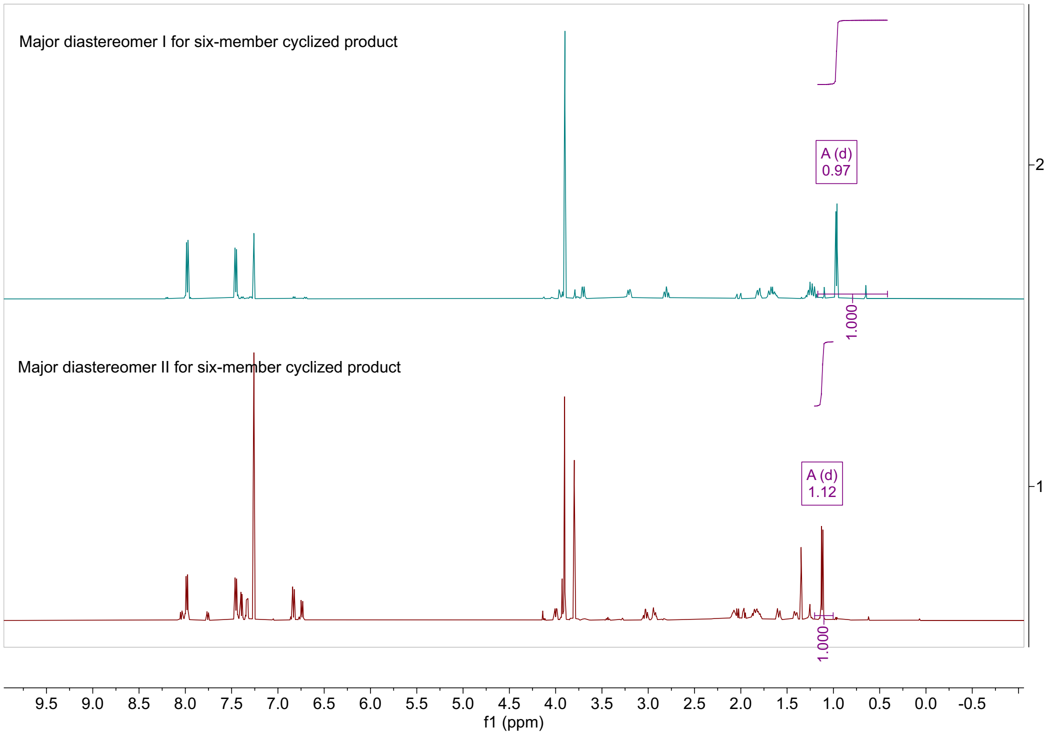

Isolable two major products: two diastereomers of six-member cyclized products
(yields reported as uHPLC assay yields)

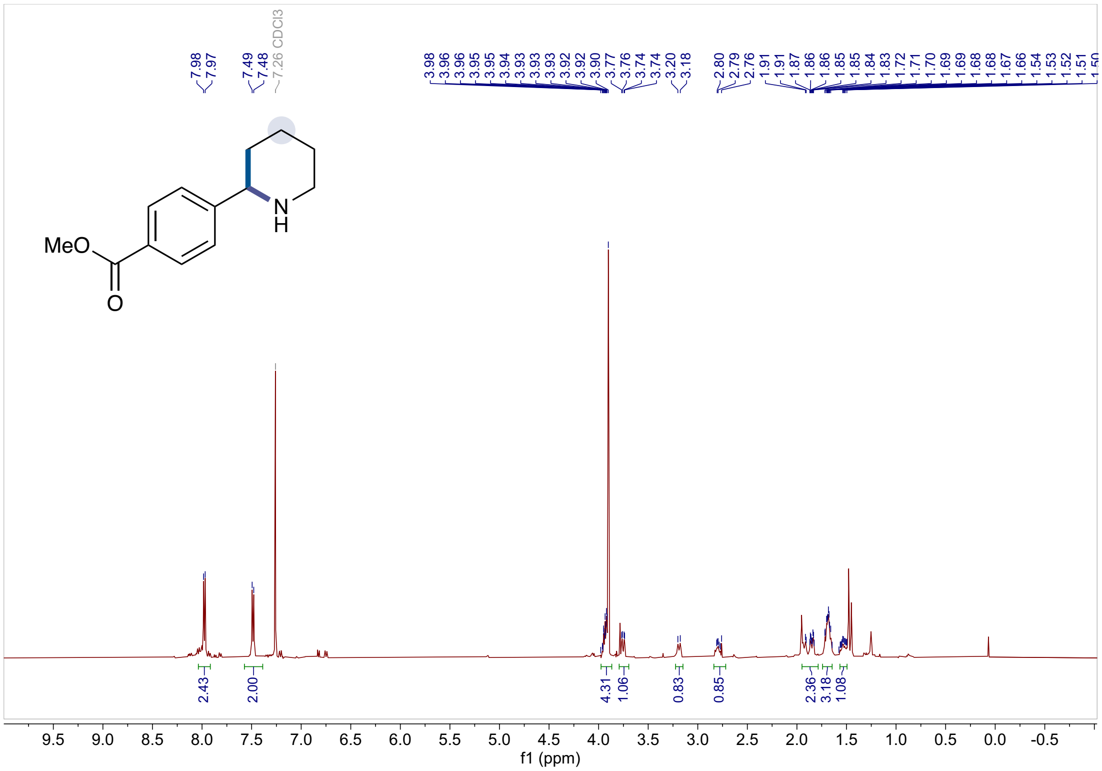

9 – ^{1}H NMR (500 MHz, $CDCl_3$)

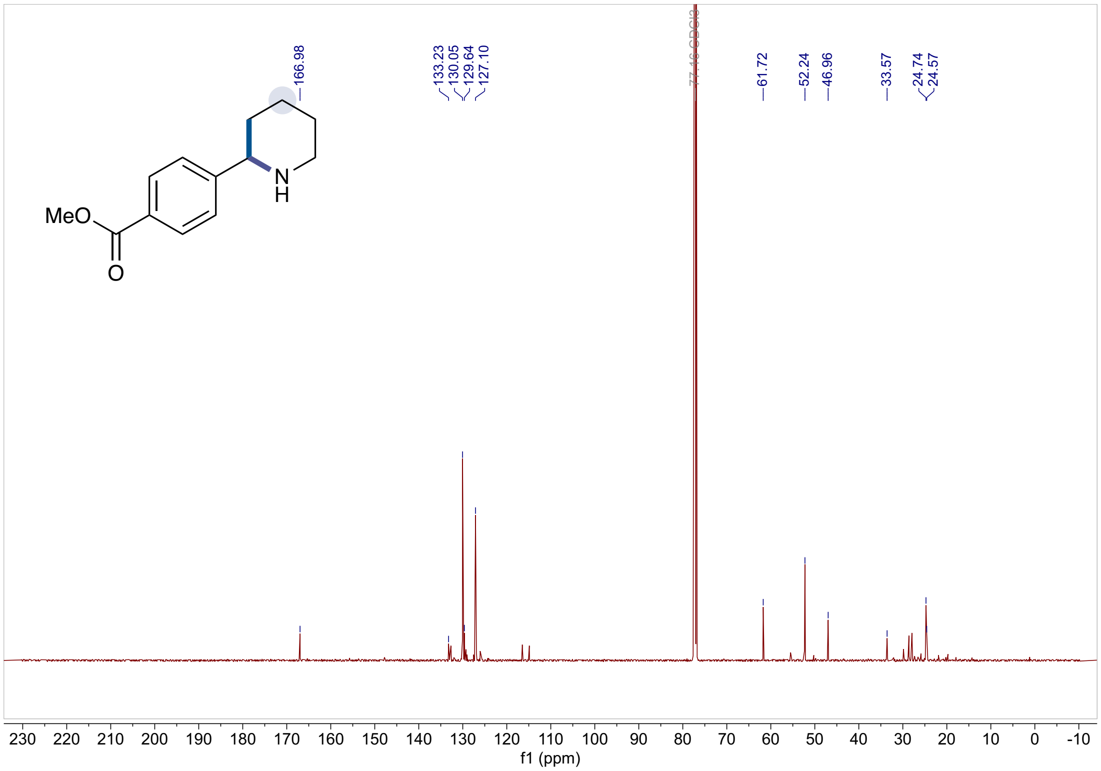

9 – ^{13}C NMR (126 MHz, $CDCl_3$)

Substrate #	Point #	Wavelength	STD retention time	STD absorption	Pdt retention time	Product absorption	pdt/std absorption ration	NMR Pdt int	NMR STD int (4H)	molar ratio (STD/pdt)
9	1	195 nm	3.569 min	305.9	2.337 min	56.6	0.185027787	0.27	4	0.27
	2			294.5		87.3	0.296434635	0.42	4	0.42

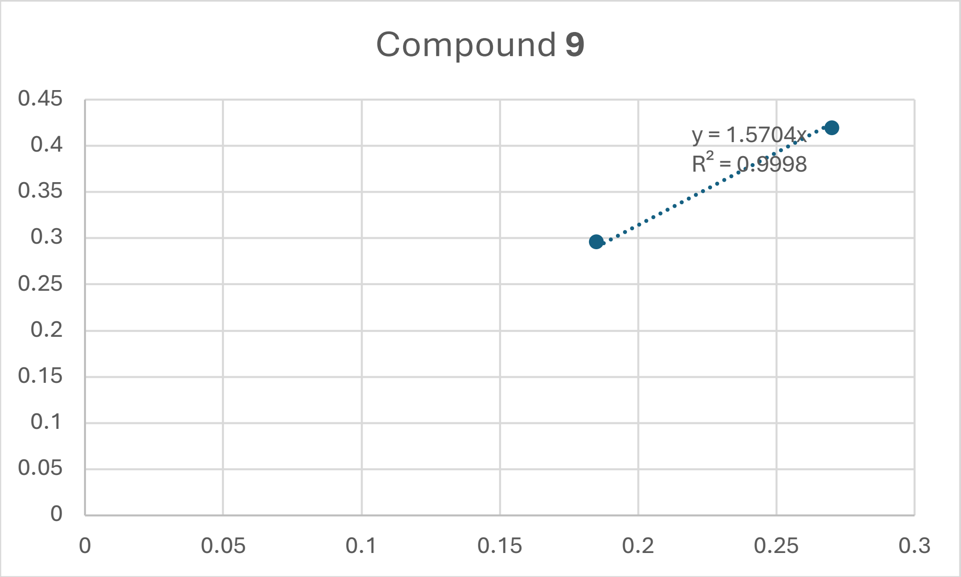

Compound **9** - uHPLC crude reaction traces

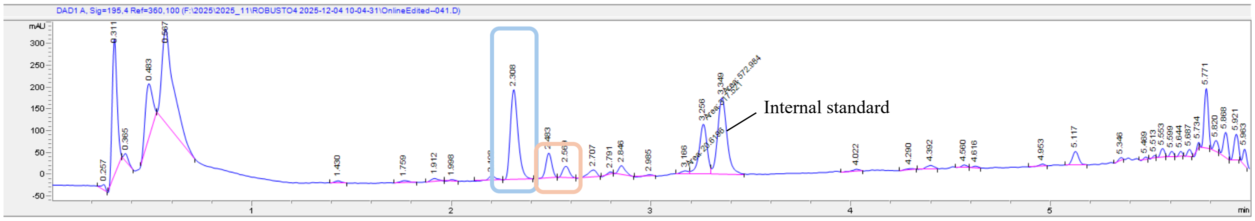

The highlighted peak (blue) corresponds to the six-membered cyclized product, which does not have any other diastereomer. The minor regioisomer, five-membered cyclized products, highlighted in orange, contain two diastereomers. The uHPLC crude traces are consistent with the isolation results that the isolatable product is six-membered cyclized product. The five-membered, minor regioisomer can be observed in trace yield with the diagonostic double from the methyl group (J = 7.2 Hz).

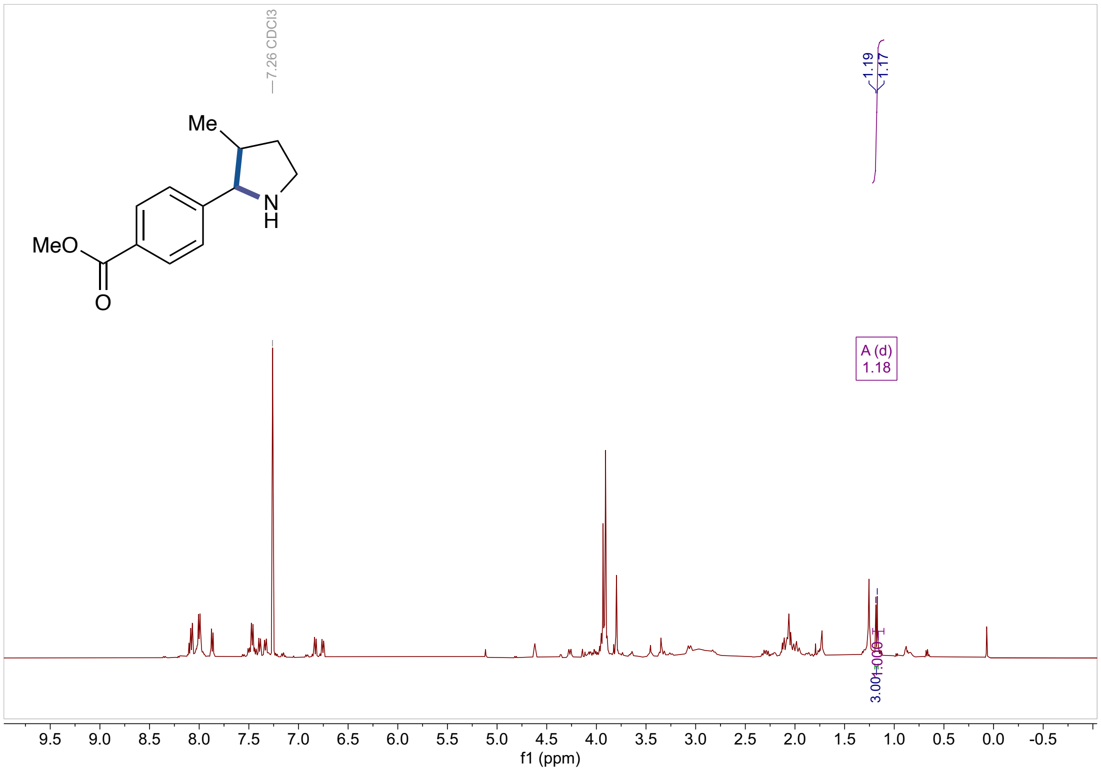

Five-membered, minor regioisomer observed in fraction mixtures

(total yields reported as uHPLC assay yields)

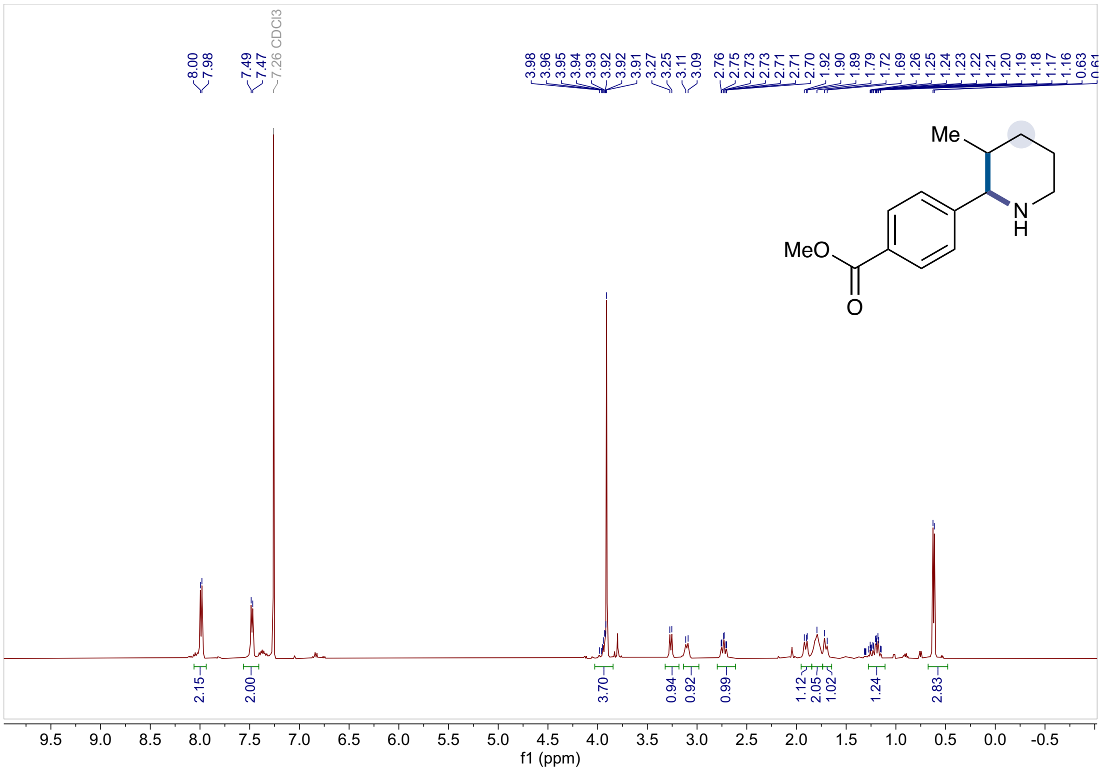

10 – ^{1}H NMR (500 MHz, $CDCl_3$)

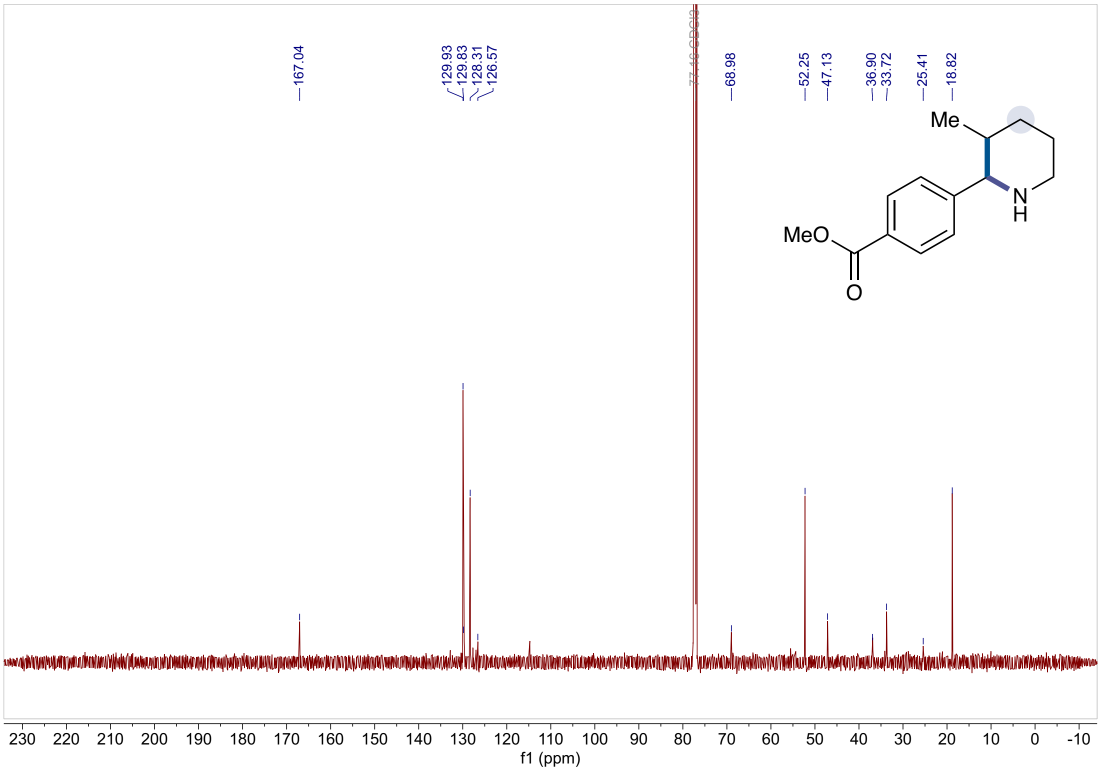

10 – ^{13}C NMR (126 MHz, $CDCl_3$)

Substrate #	Point #	Wavelength	STD retention time	STD absorpti	Pdt retention	Product absorption	pdt/std absorption ration	NMR Pdt int	NMR STD int (4H)	molar ratio (STD/pdt)
	1			862.1		179	0.207632525	0.42	4	0.21
10	2	195 nm	3.476 min	943	2.988 min	203.1	0.215376458	0.43	4	0.215
	3			1236.1		303.5	0.245530297	0.47	4	0.235

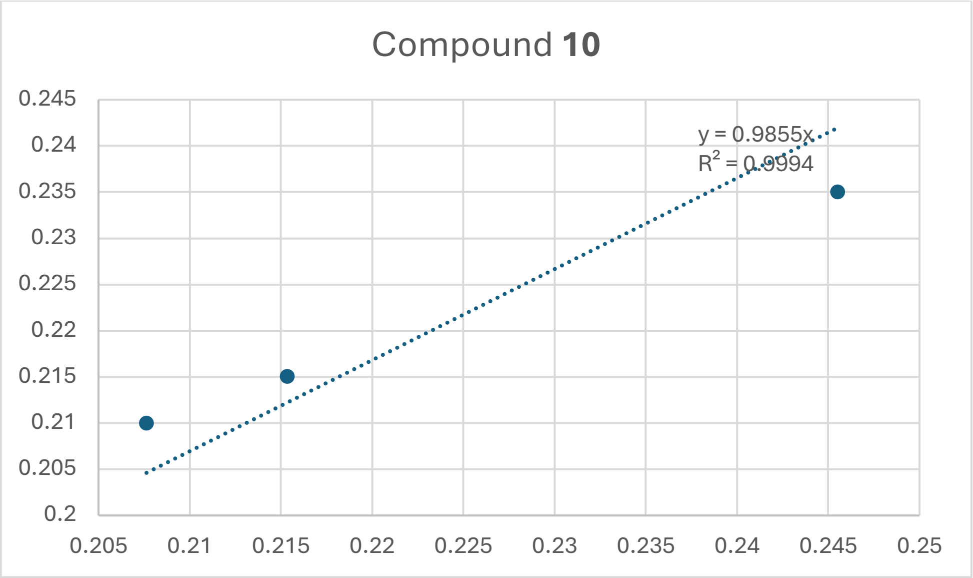

Compound **10** - uHPLC crude reaction traces

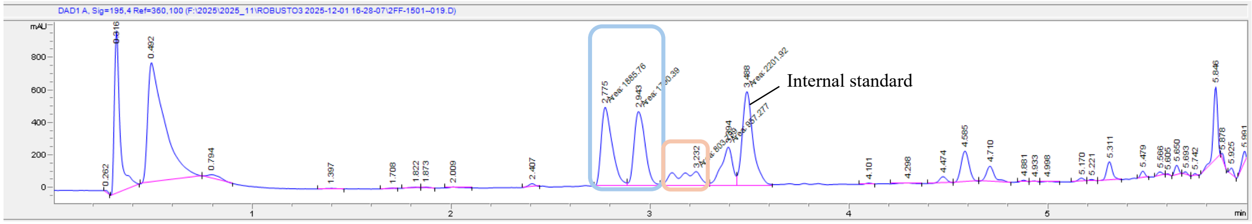

The highlighted two peaks correspond to the two diastereomers of the six-membered cyclized products. The minor regioisomer, five- and seven- membered cyclized product are observed in significantly lower yields (orange). The uHPLC crude traces are consistent with the isolation results that the isolatable products are two diastereomers of the six-membered cyclized products.

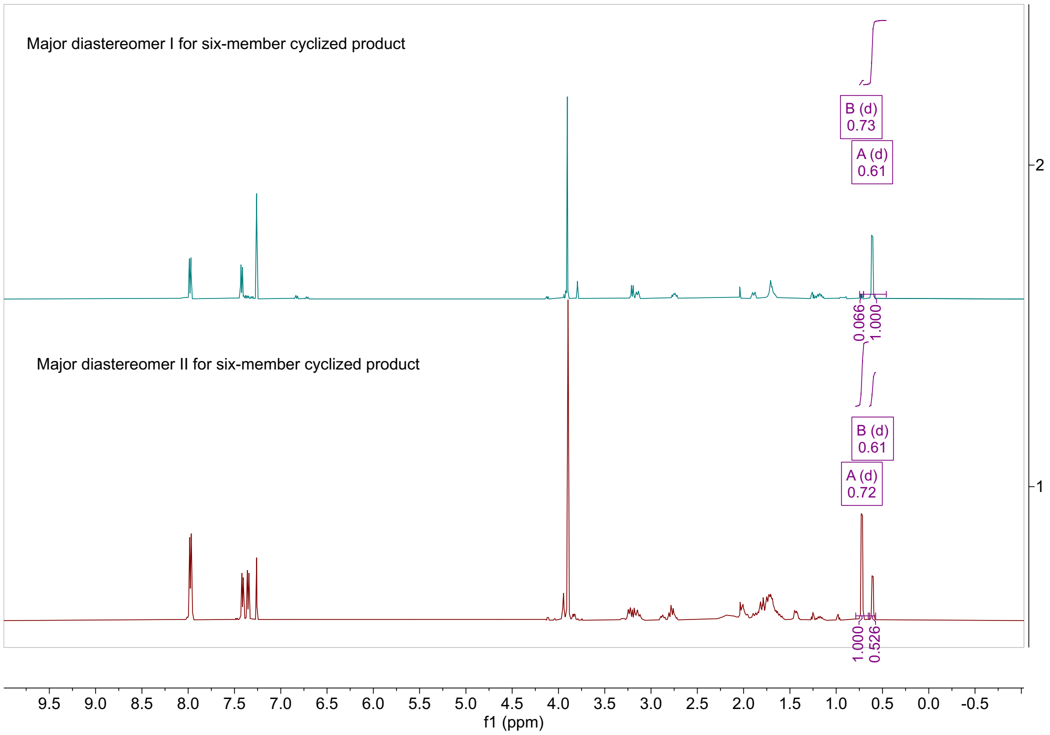

Isolable two major products: two diastereomers of six-member cyclized products
(yields reported as uHPLC assay yields)

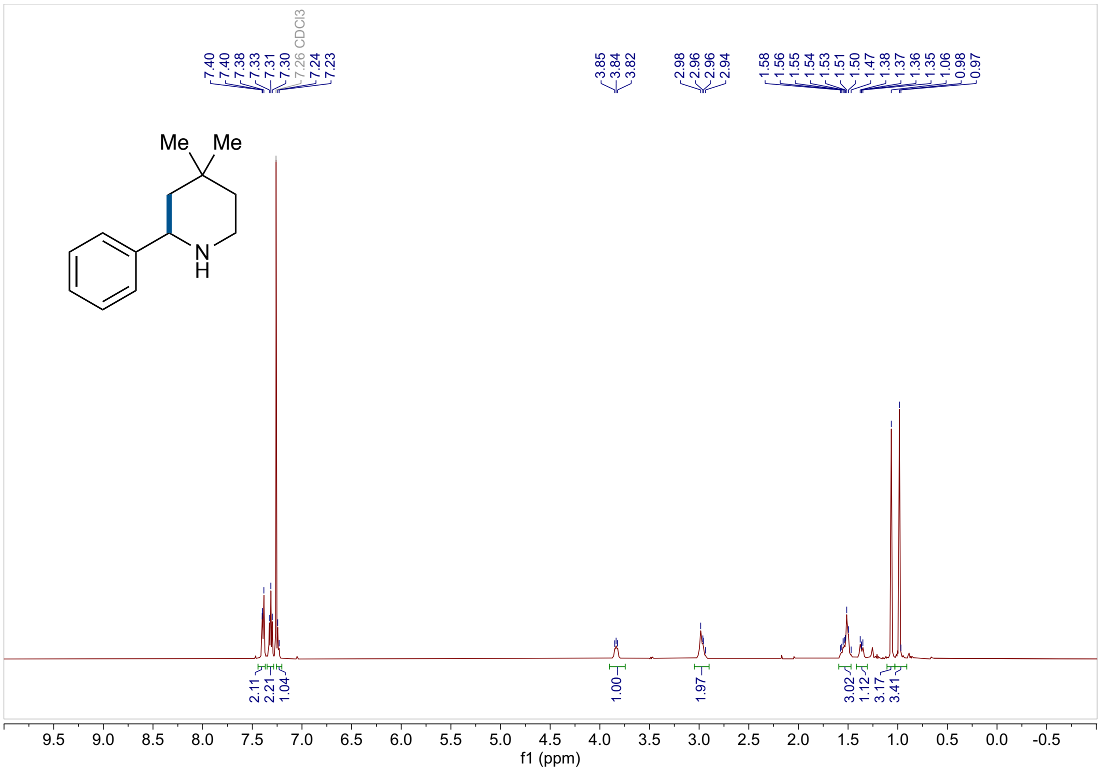

11 – ^{1}H NMR (500 MHz, $CDCl_3$)

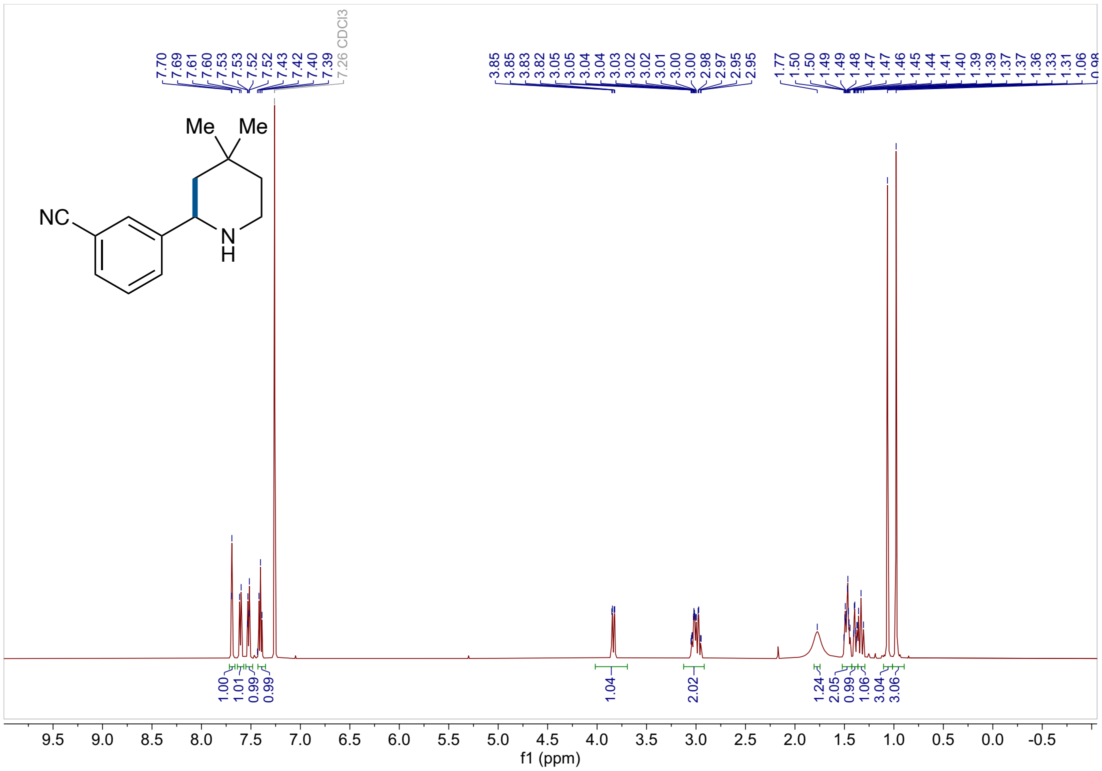

12 – ^{1}H NMR (500 MHz, $CDCl_3$)

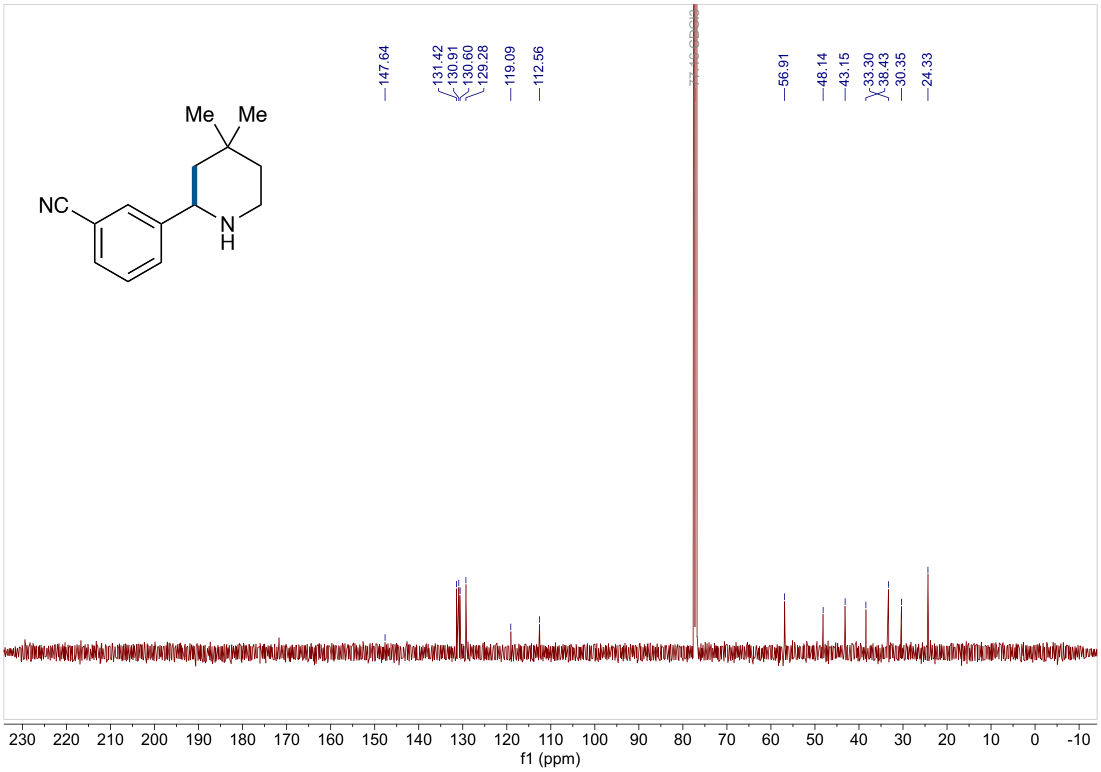

12 – ^{13}C NMR (126 MHz, $CDCl_3$)

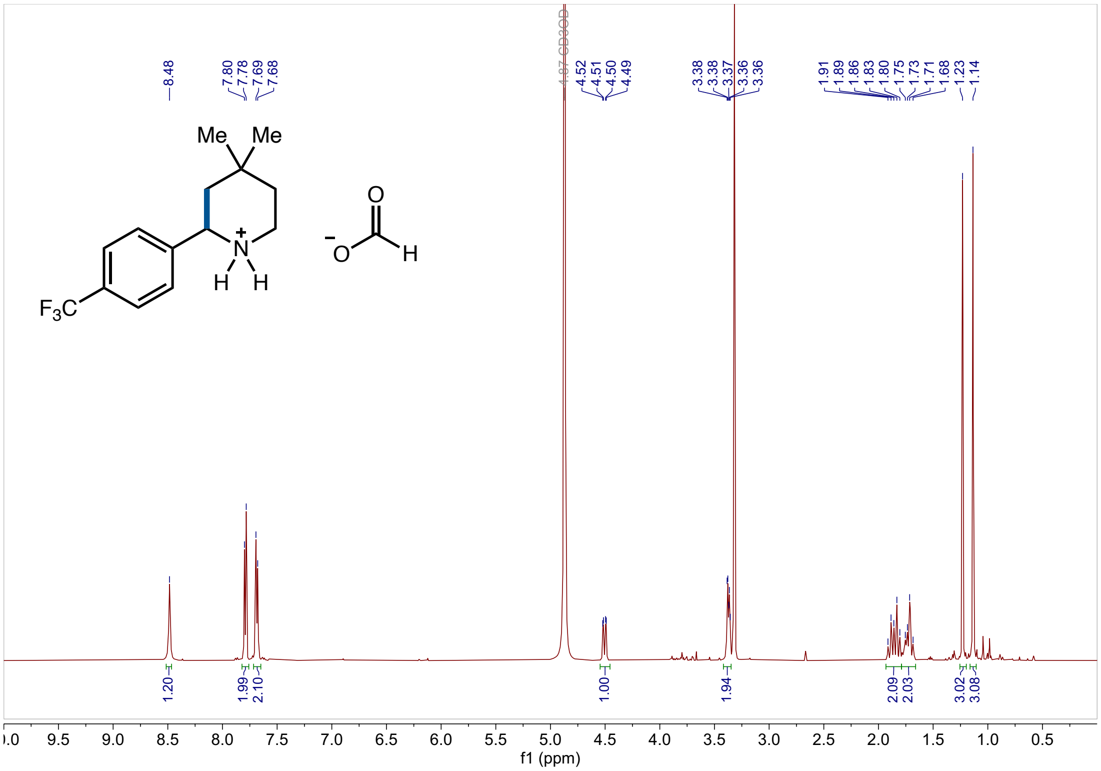

13 – ^{1}H NMR (500 MHz, MeOD)

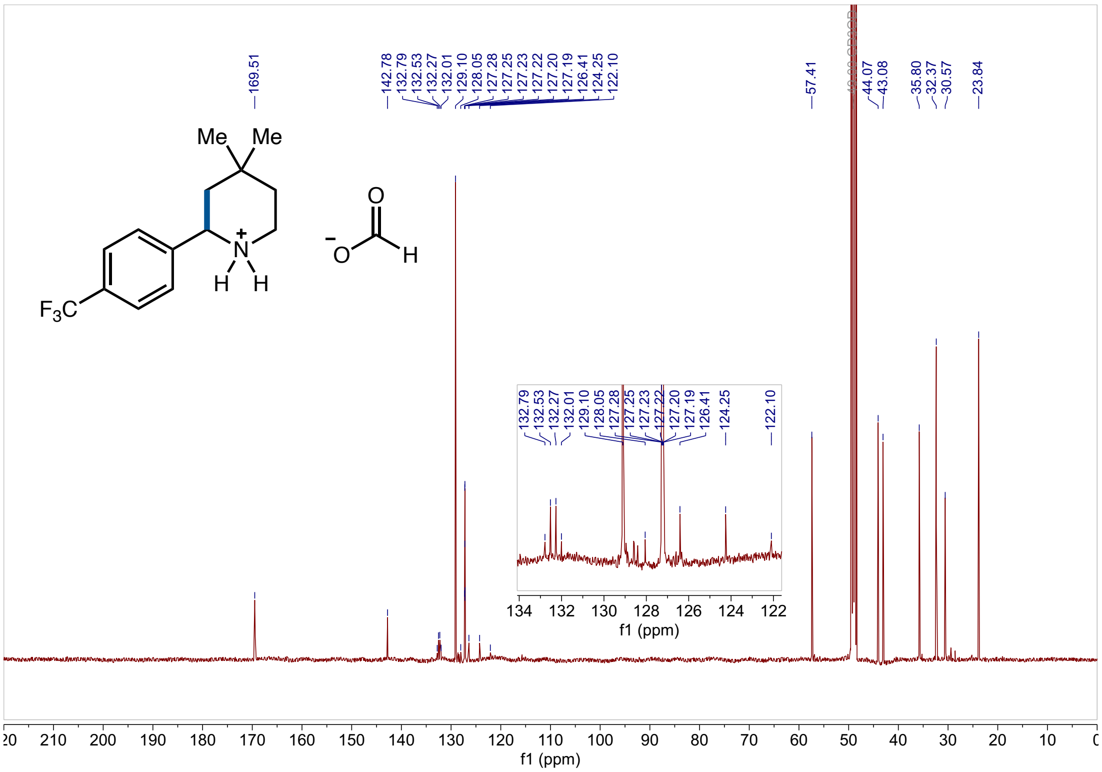

13 – ^{13}C NMR (126 MHz, MeOD)

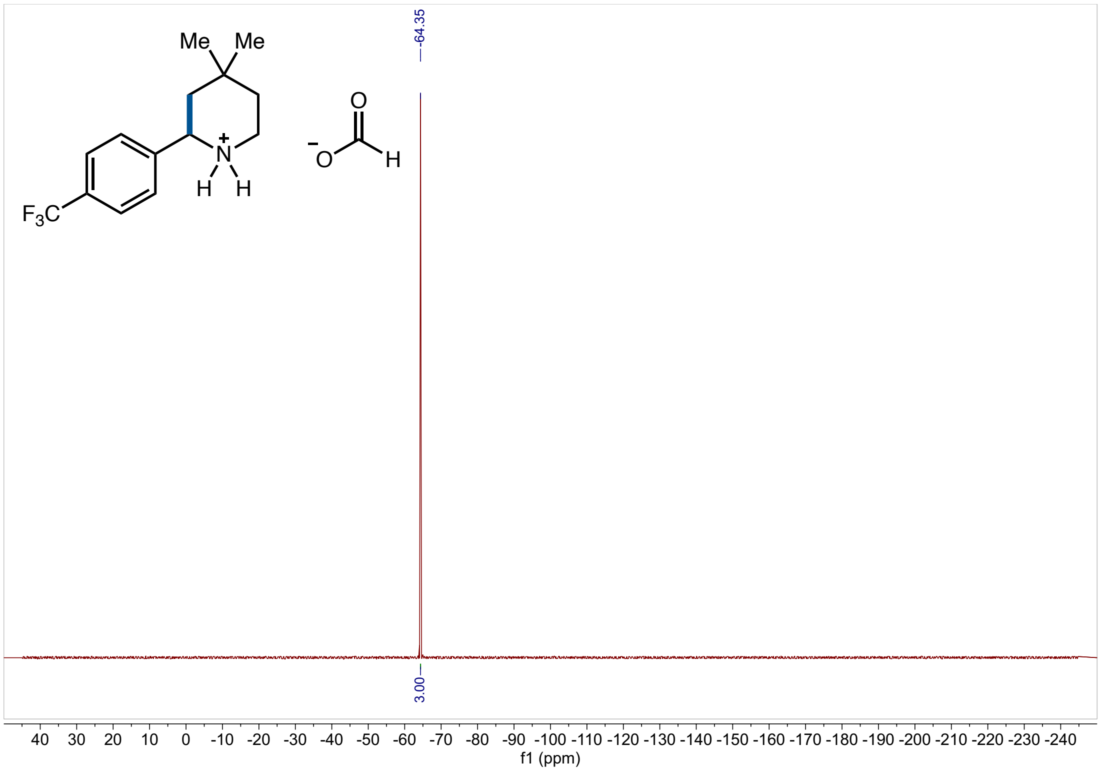

13 – ^{19}F NMR (282 MHz, MeOD)

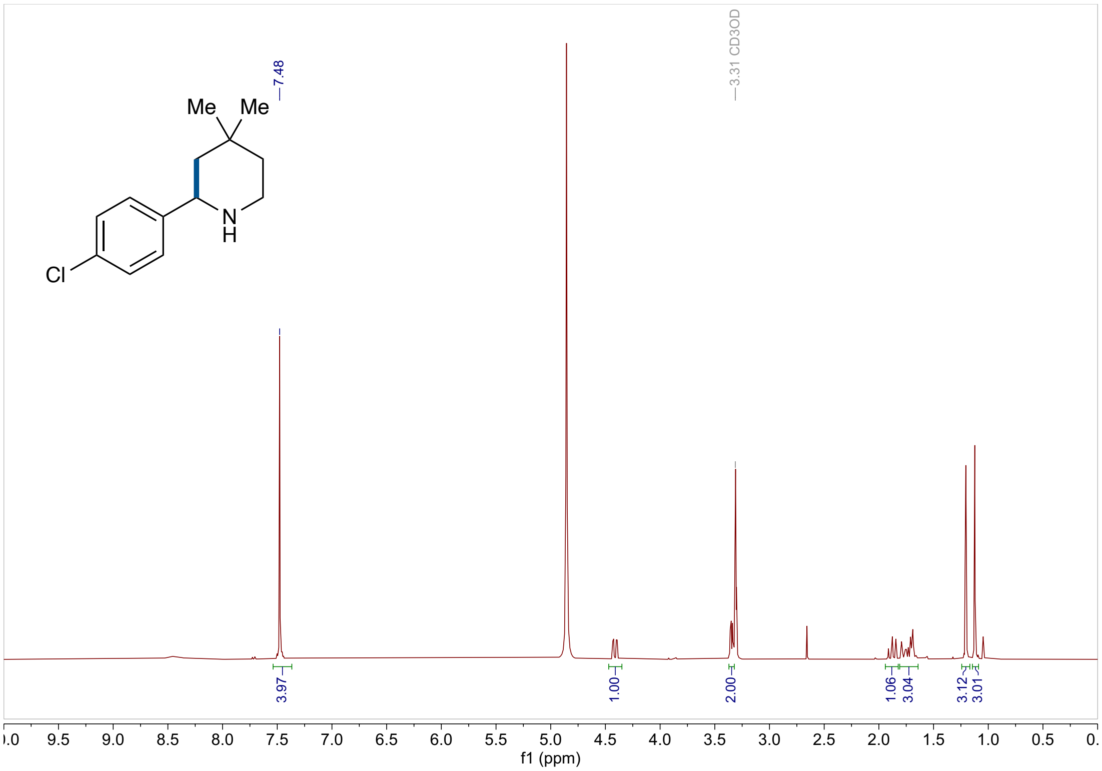

14 – ^{1}H NMR (400 MHz, MeOD)

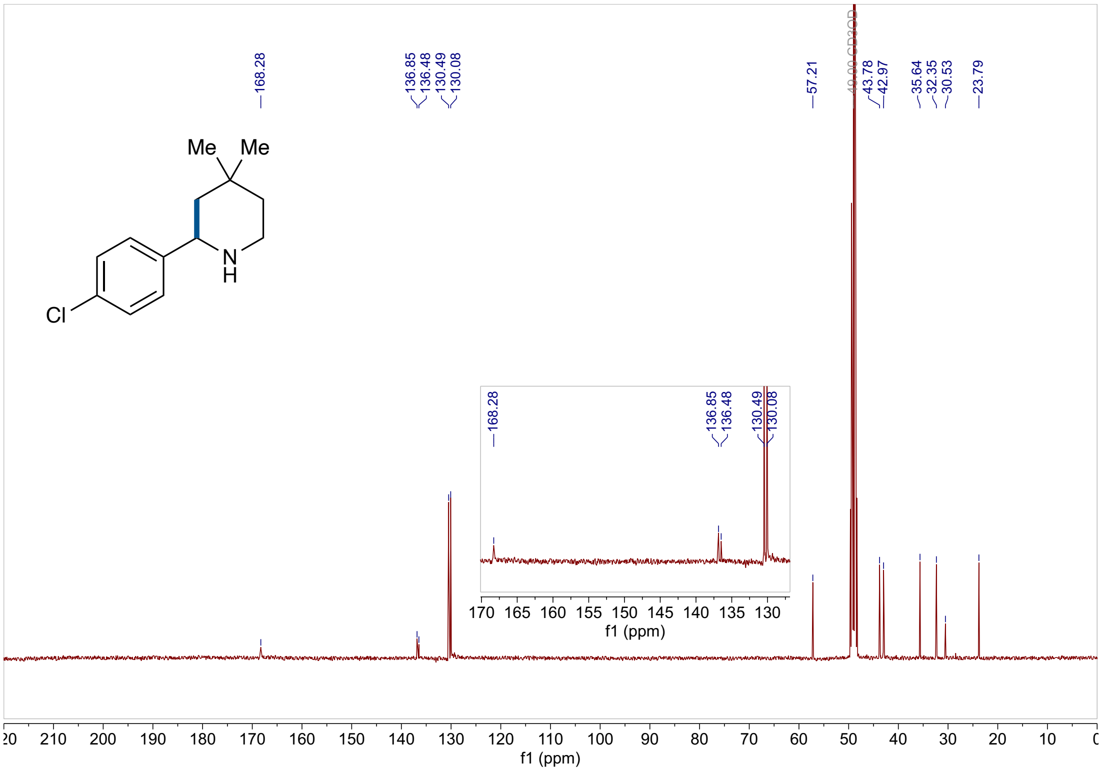

14 – ^{13}C NMR (101 MHz, MeOD)

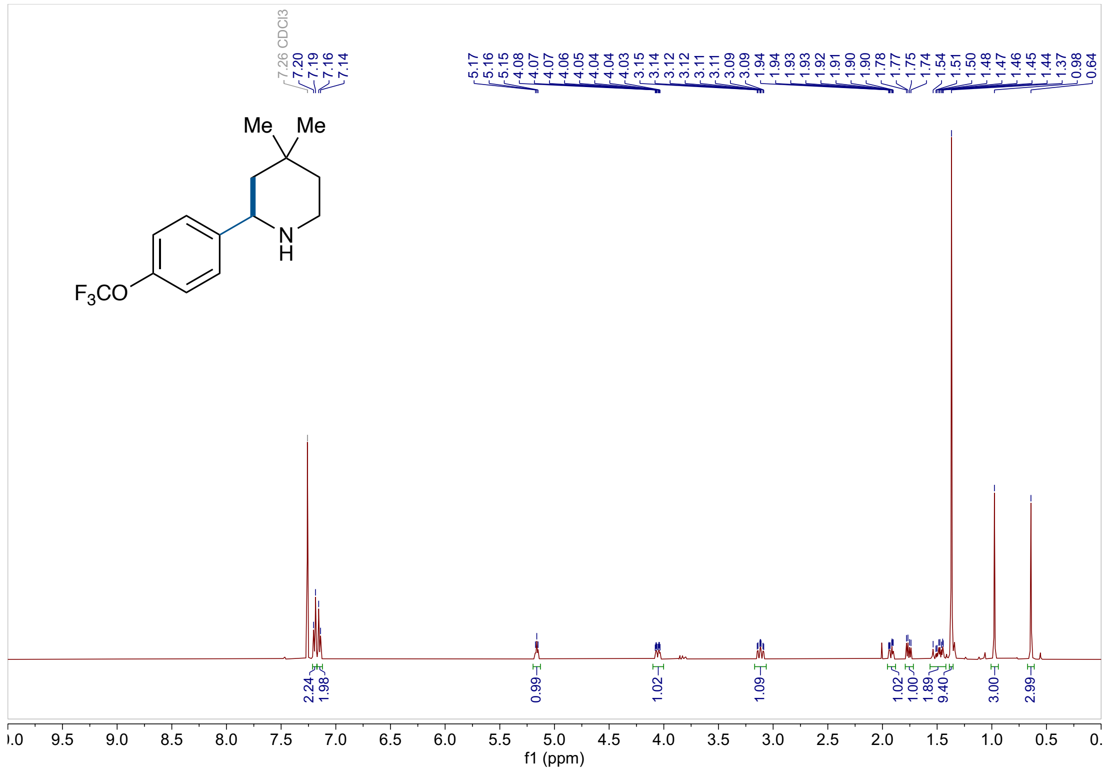

15 – ^{1}H NMR (500 MHz, $CDCl_3$)

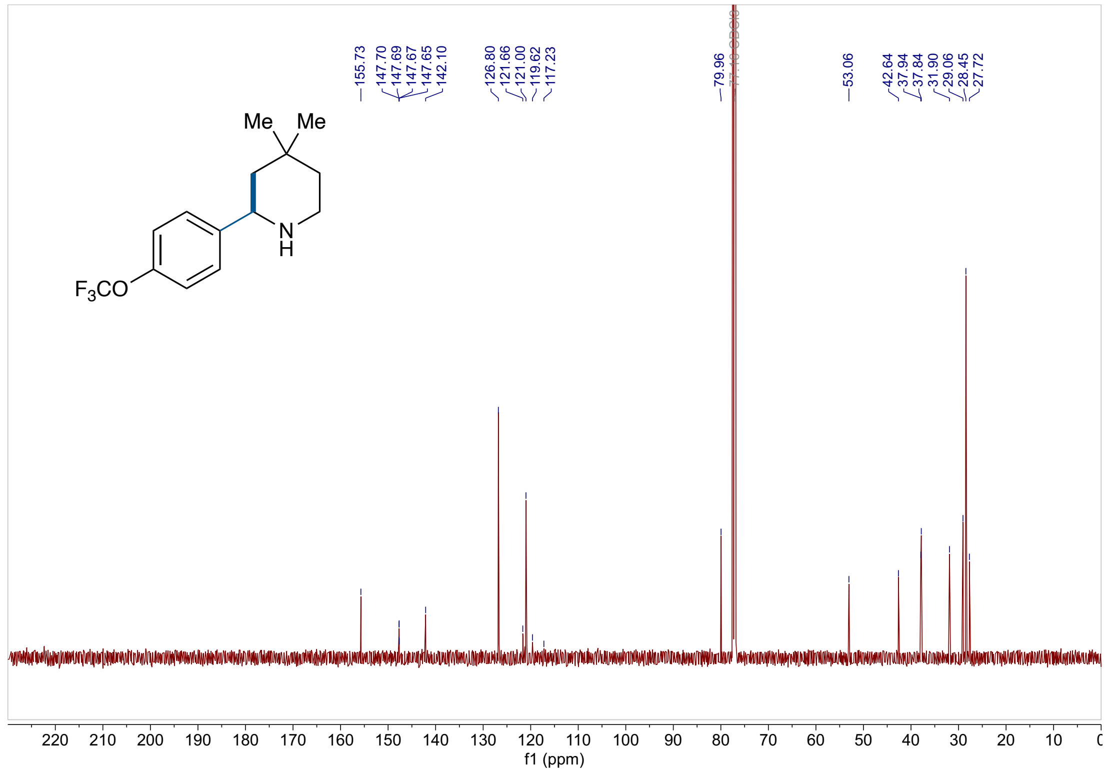

15 – ^{13}C NMR (126 MHz, $CDCl_3$)

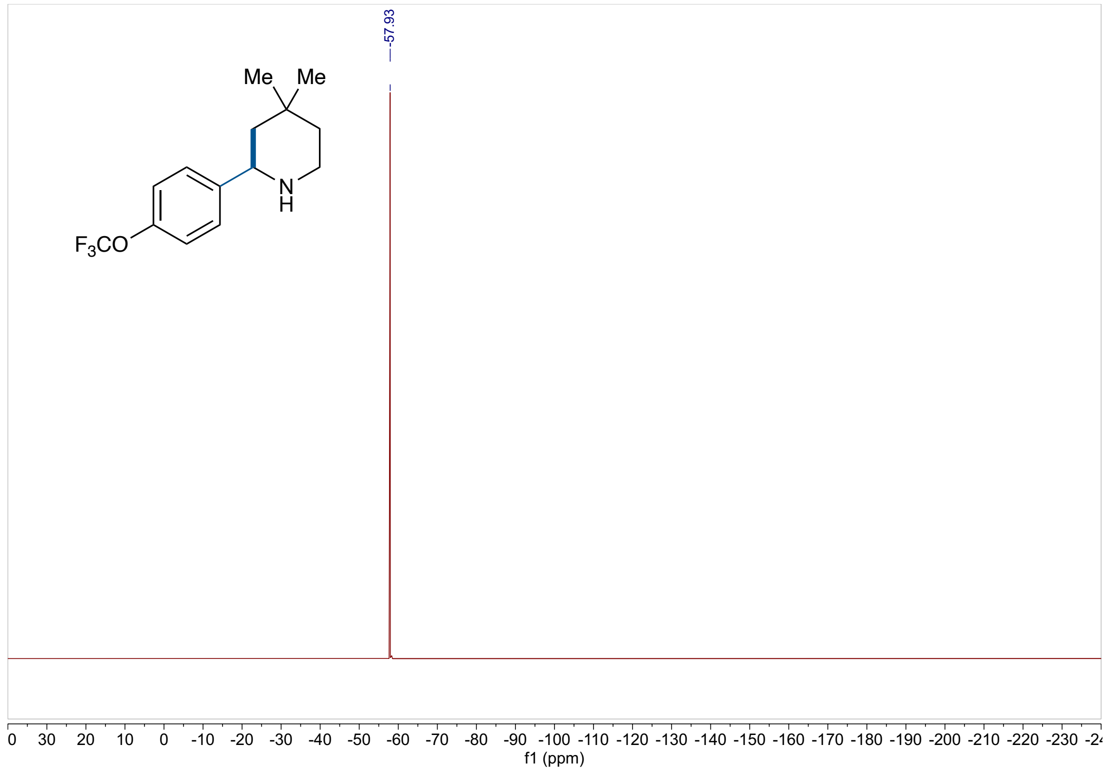

15 – ^{19}F NMR (471 MHz, $CDCl_3$)

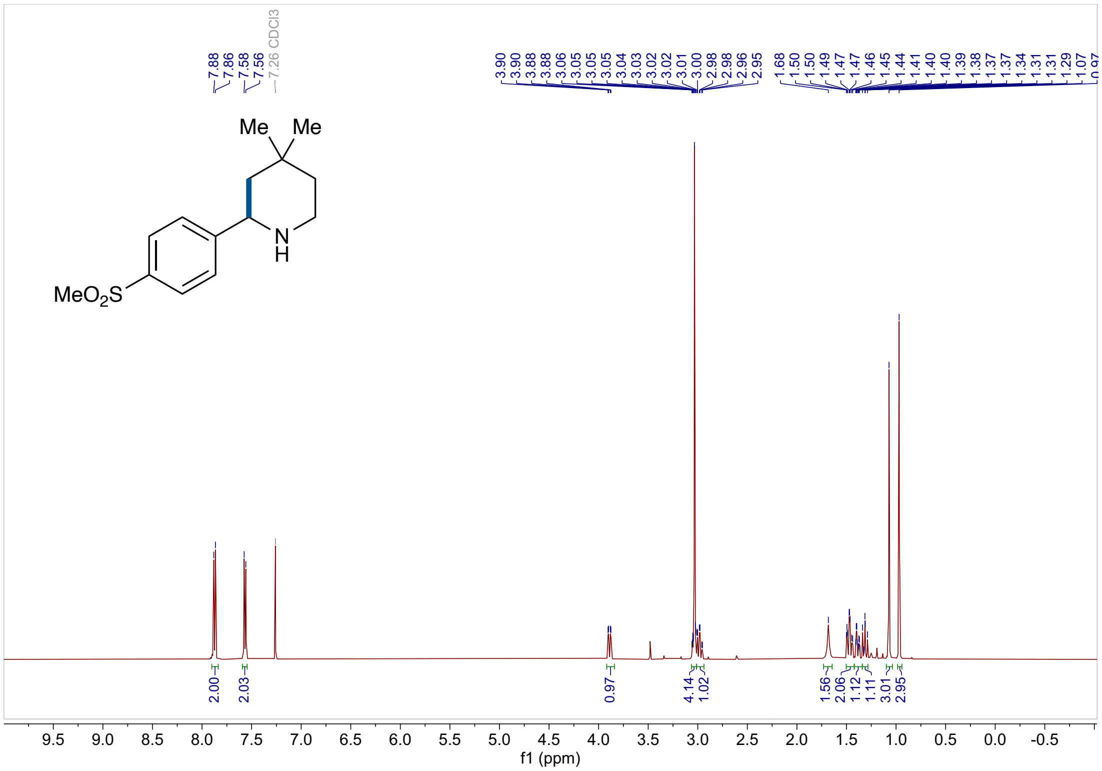

16 – ^{1}H NMR (500 MHz, $CDCl_3$)

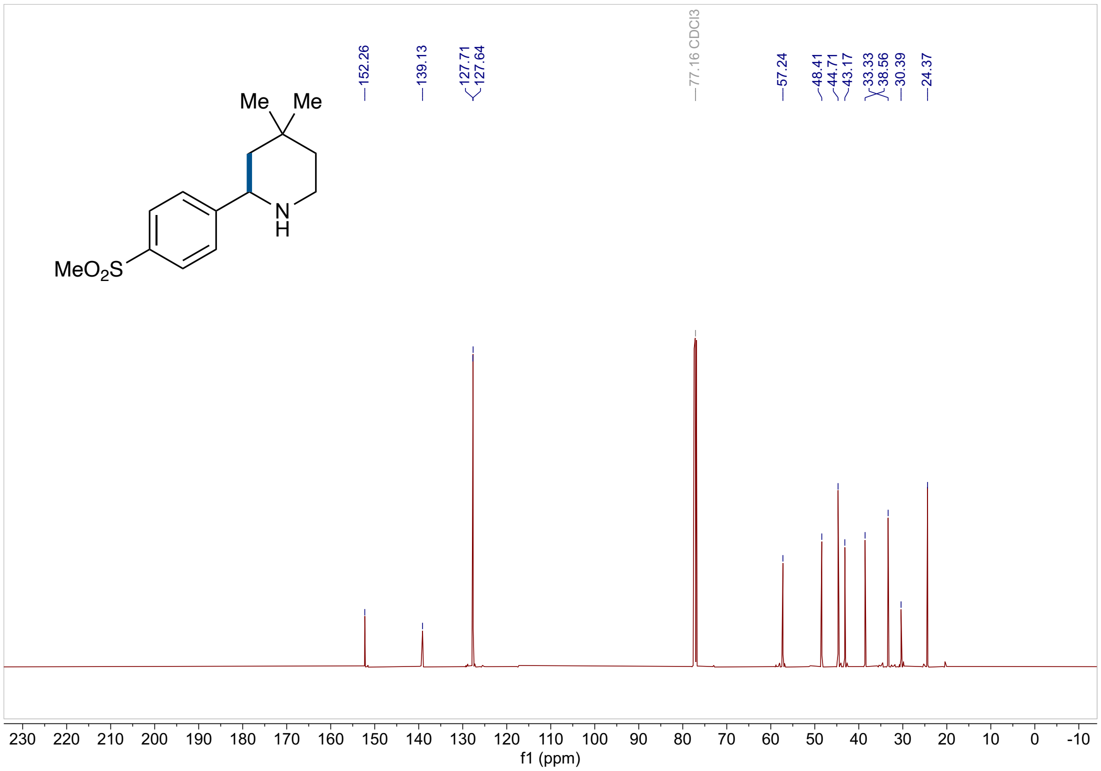

16 – ^{13}C NMR (126 MHz, $CDCl_3$)

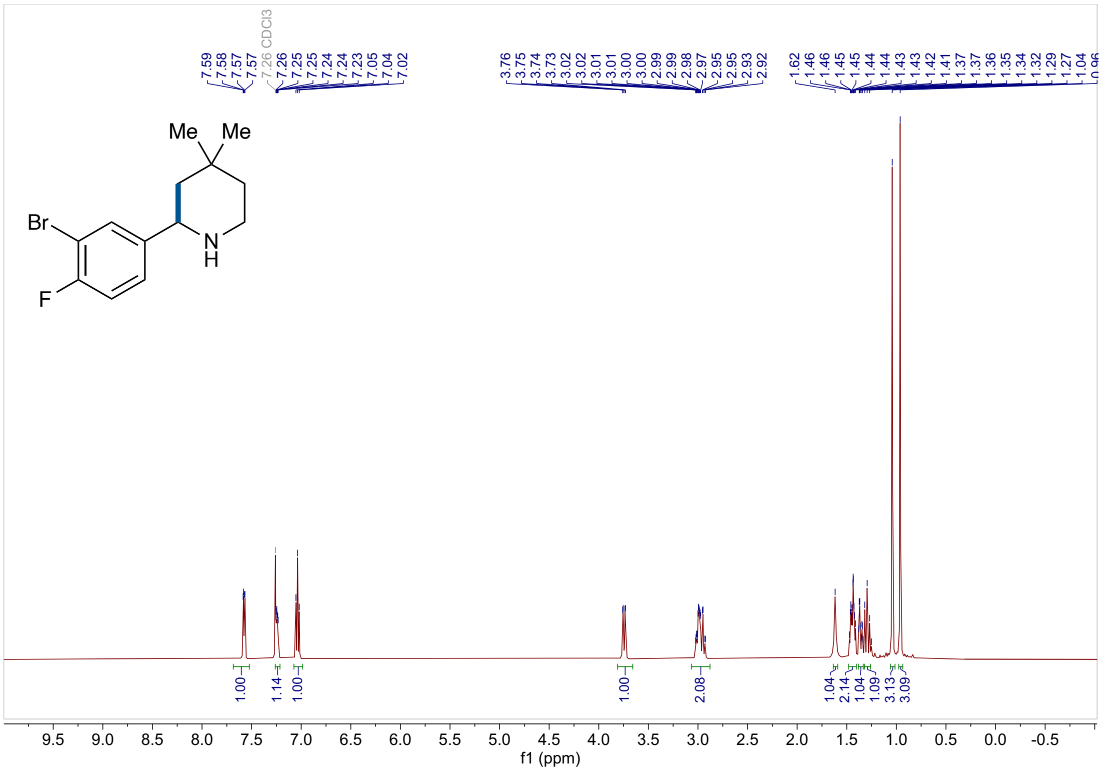

17 – ^{1}H NMR (500 MHz, $CDCl_3$)

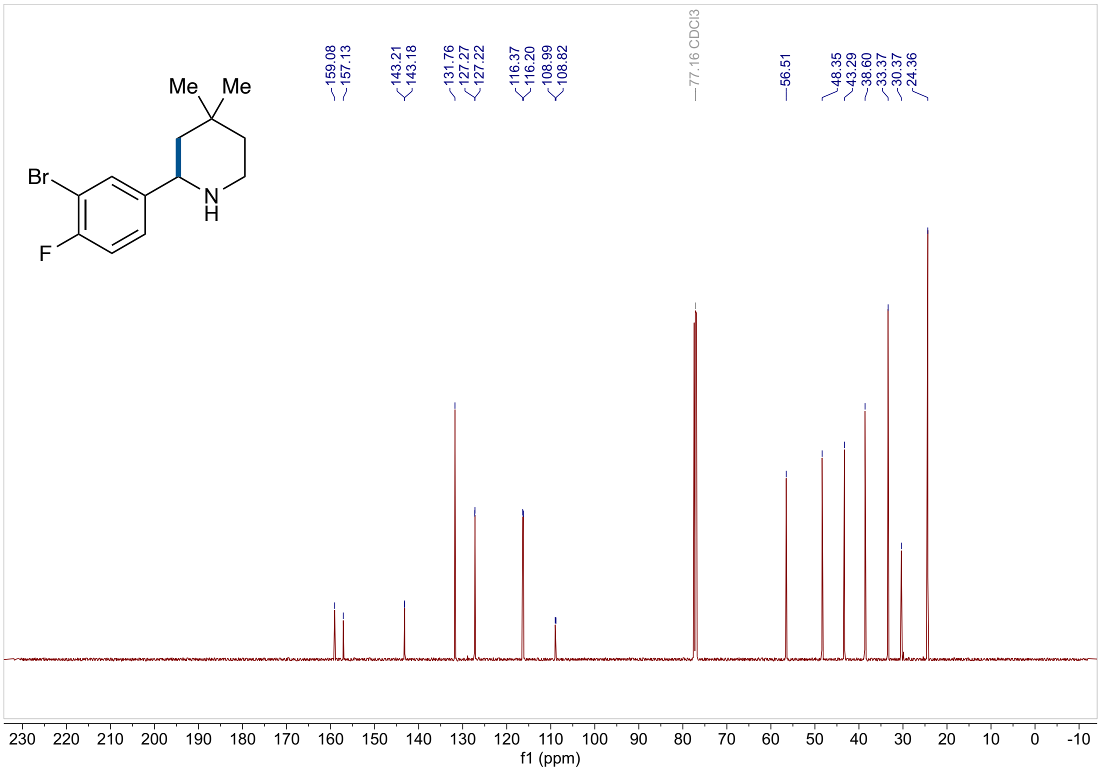

17 – ^{13}C NMR (126 MHz, $CDCl_3$)

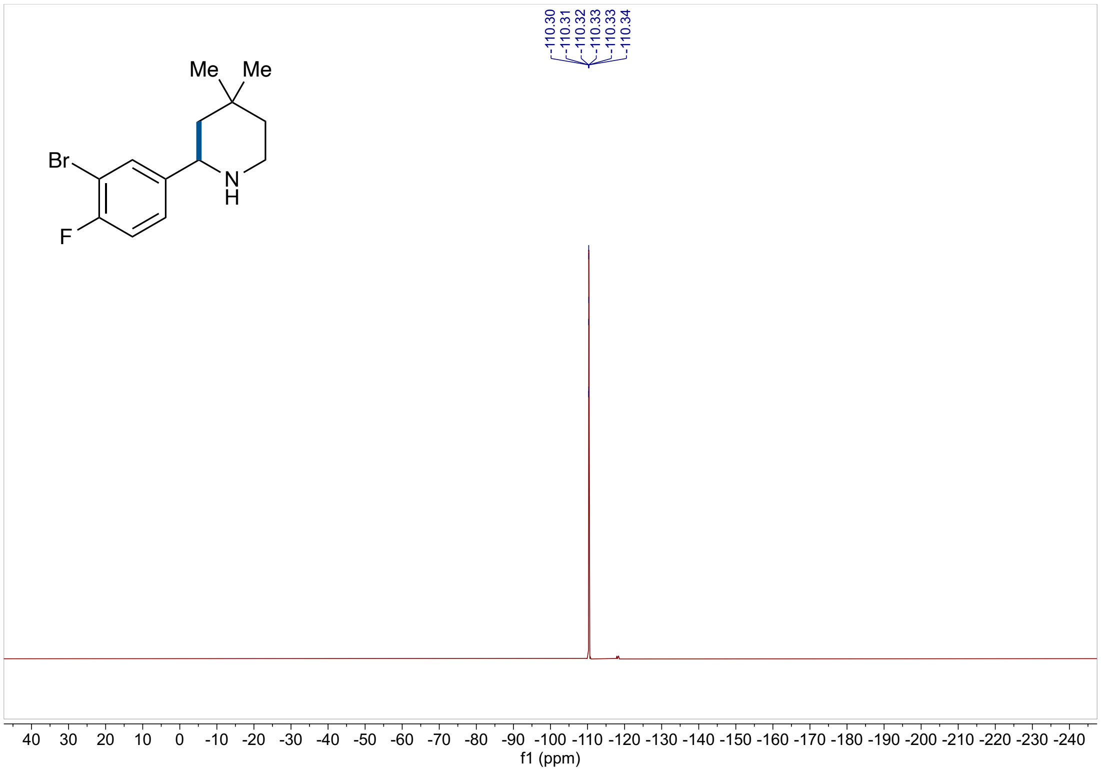

17 – ^{19}F NMR (471 MHz, $CDCl_3$)

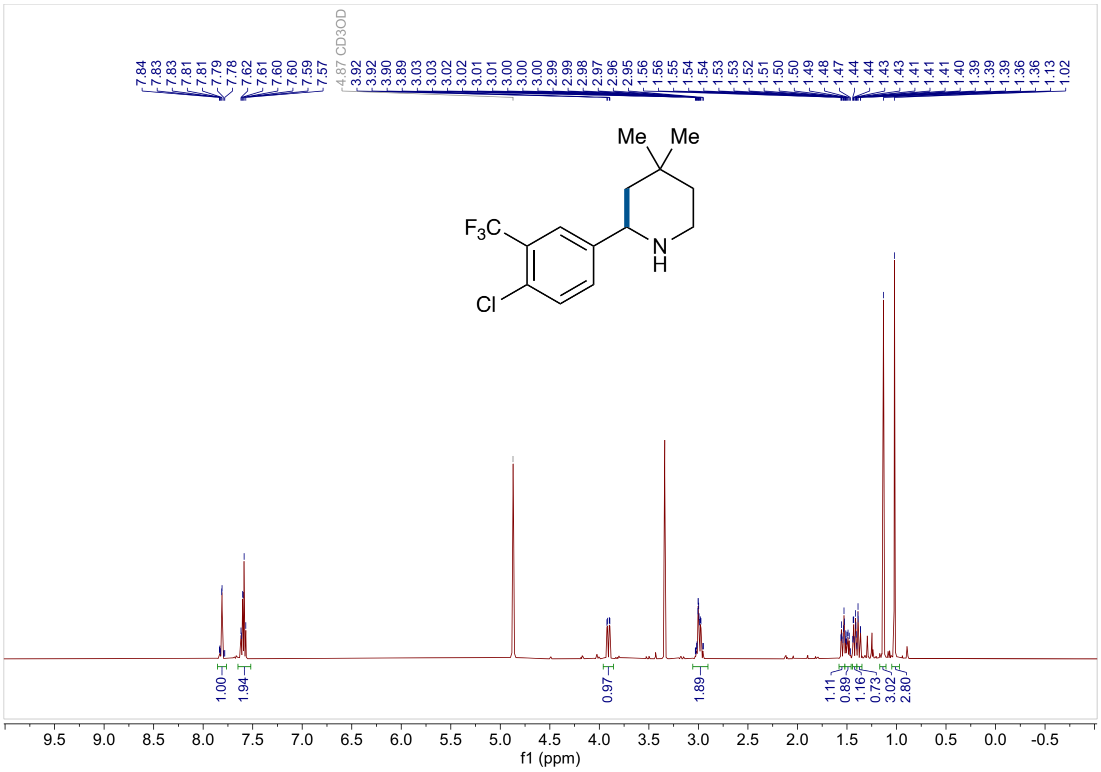

18 – ^{1}H NMR (500 MHz, MeOD)

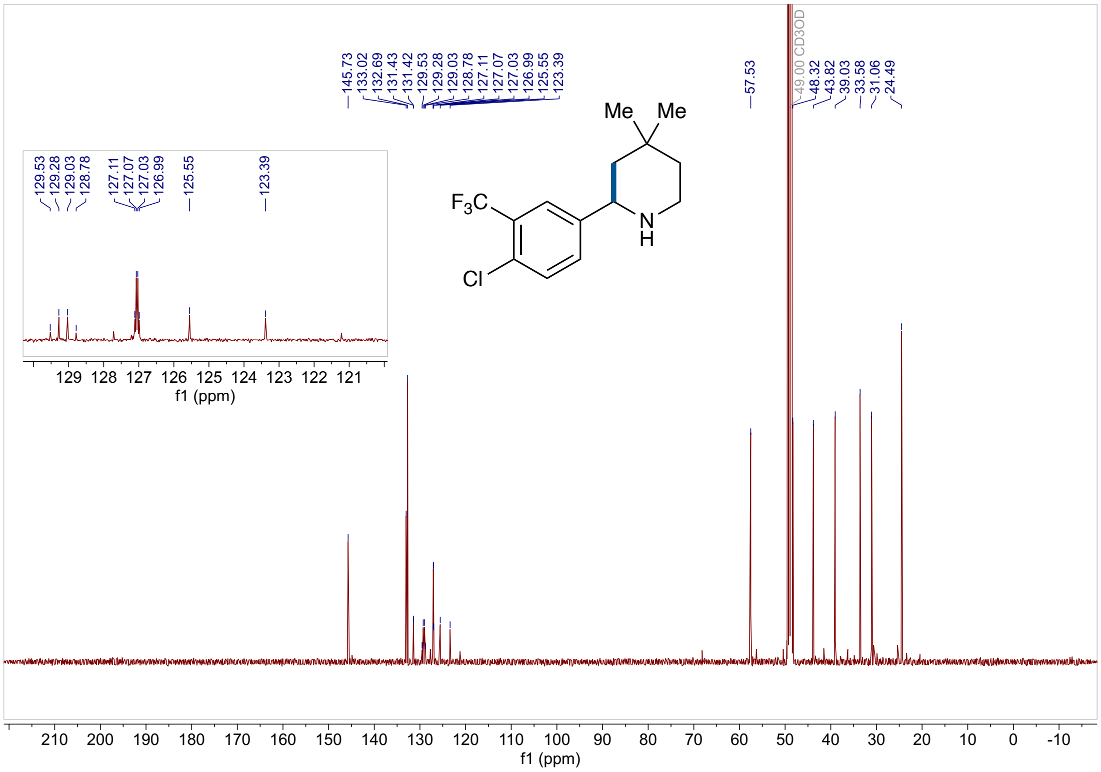

18 – ^{13}C NMR (126 MHz, MeOD)

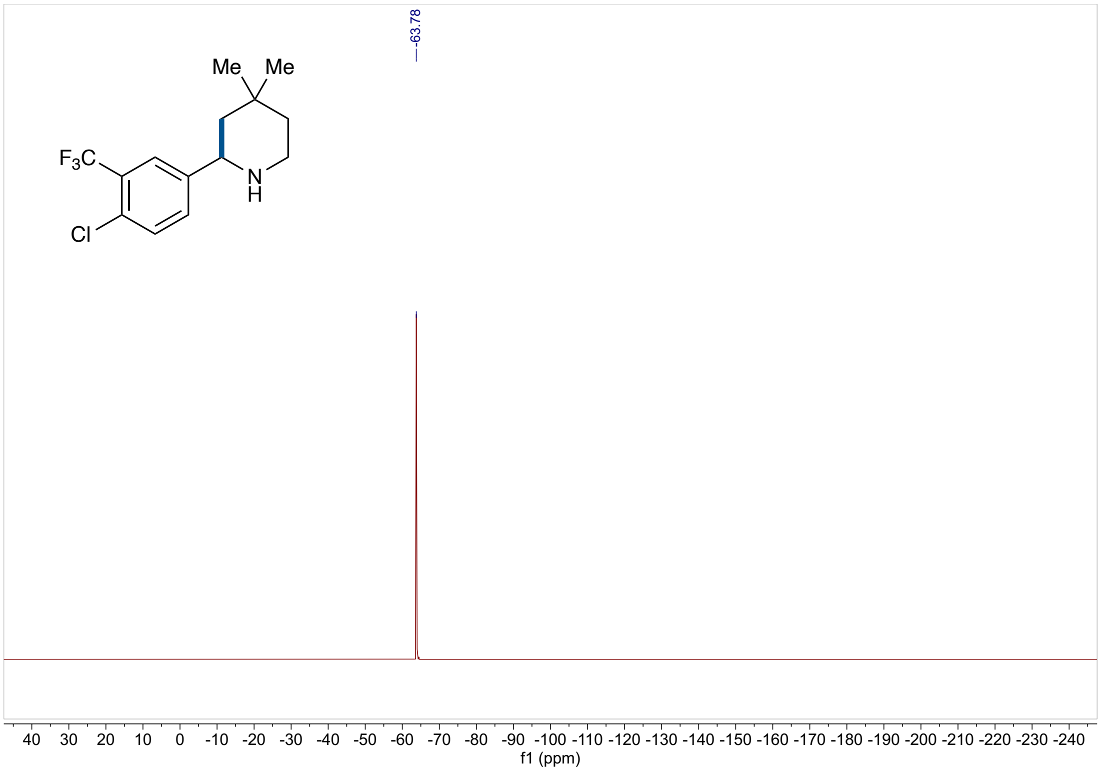

18 – ^{19}F NMR (471 MHz, MeOD)

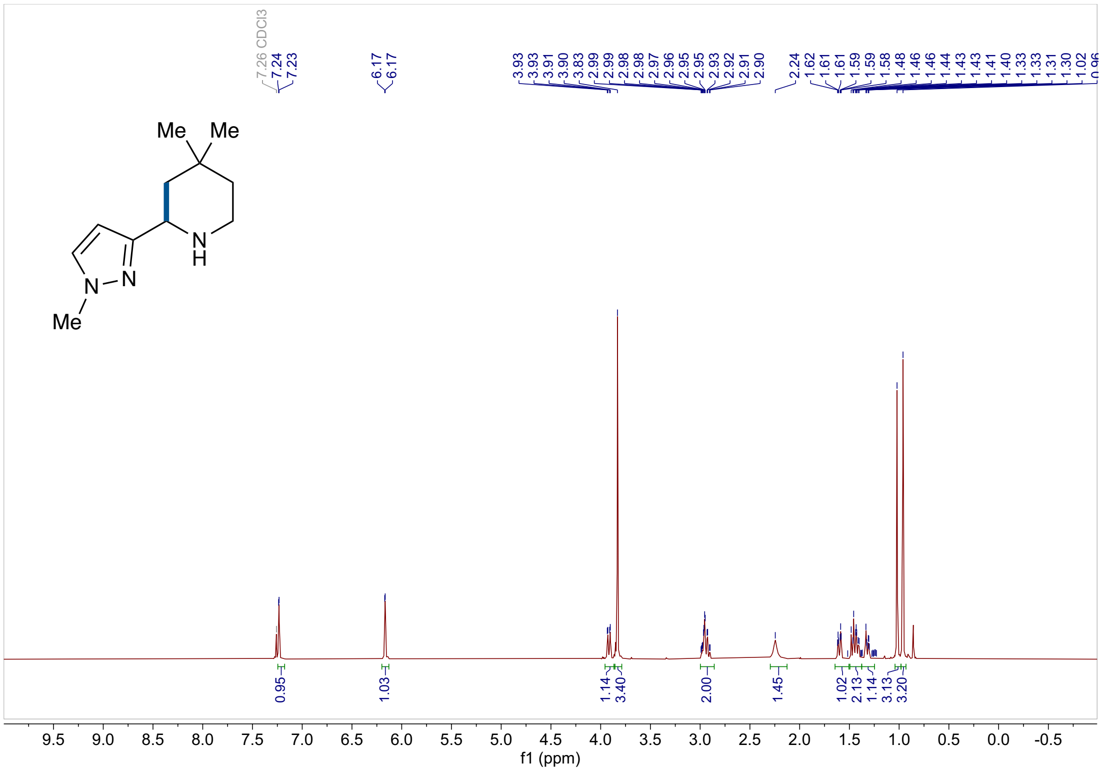

19 – ^{1}H NMR (500 MHz, $CDCl_3$)

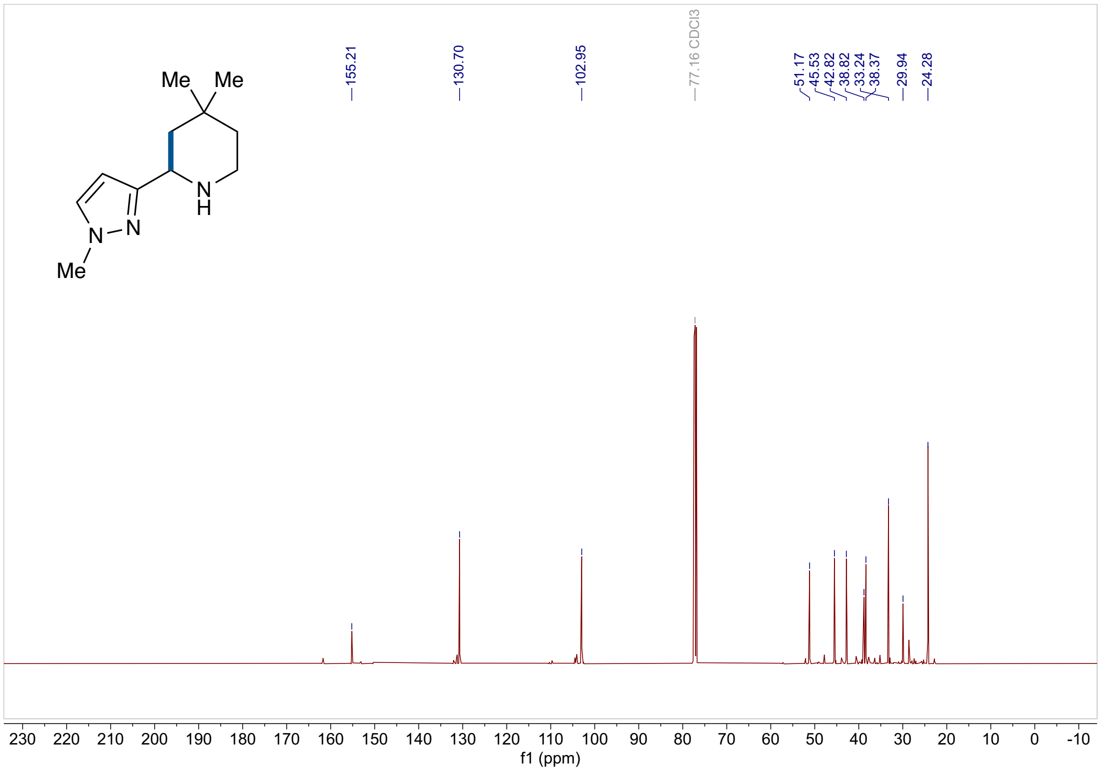

19 – ^{13}C NMR (126 MHz, $CDCl_3$)

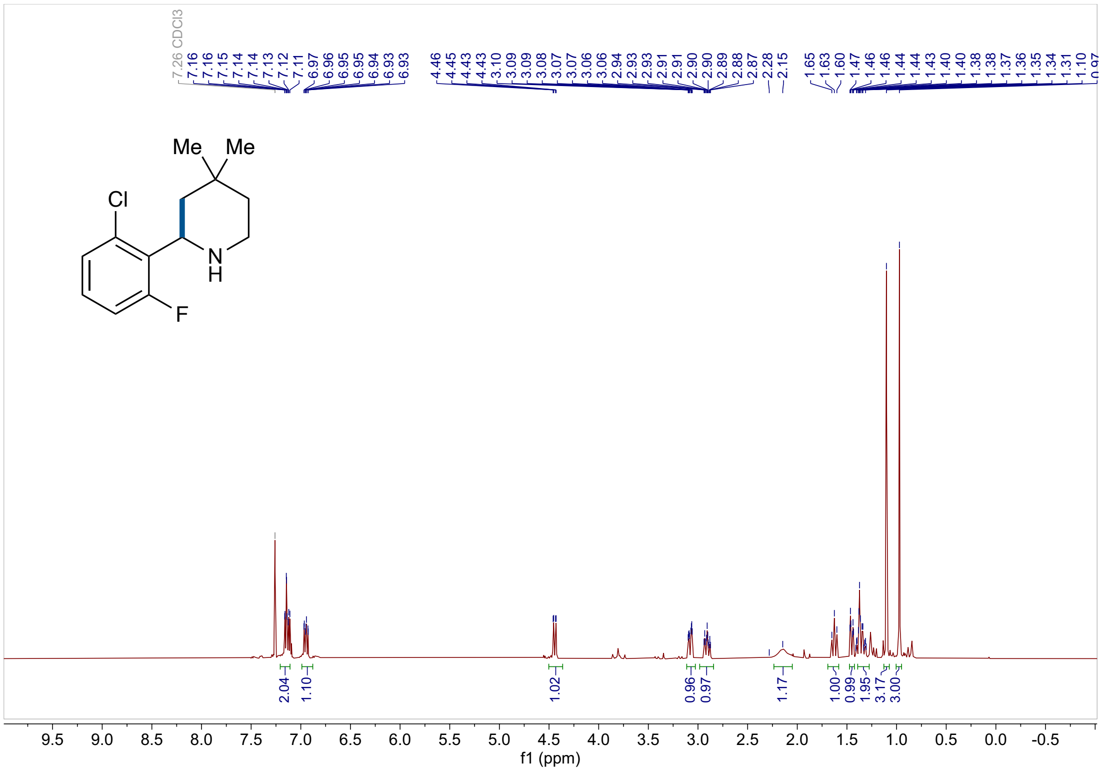

20 – ^{1}H NMR (500 MHz, CDCl$_3$)

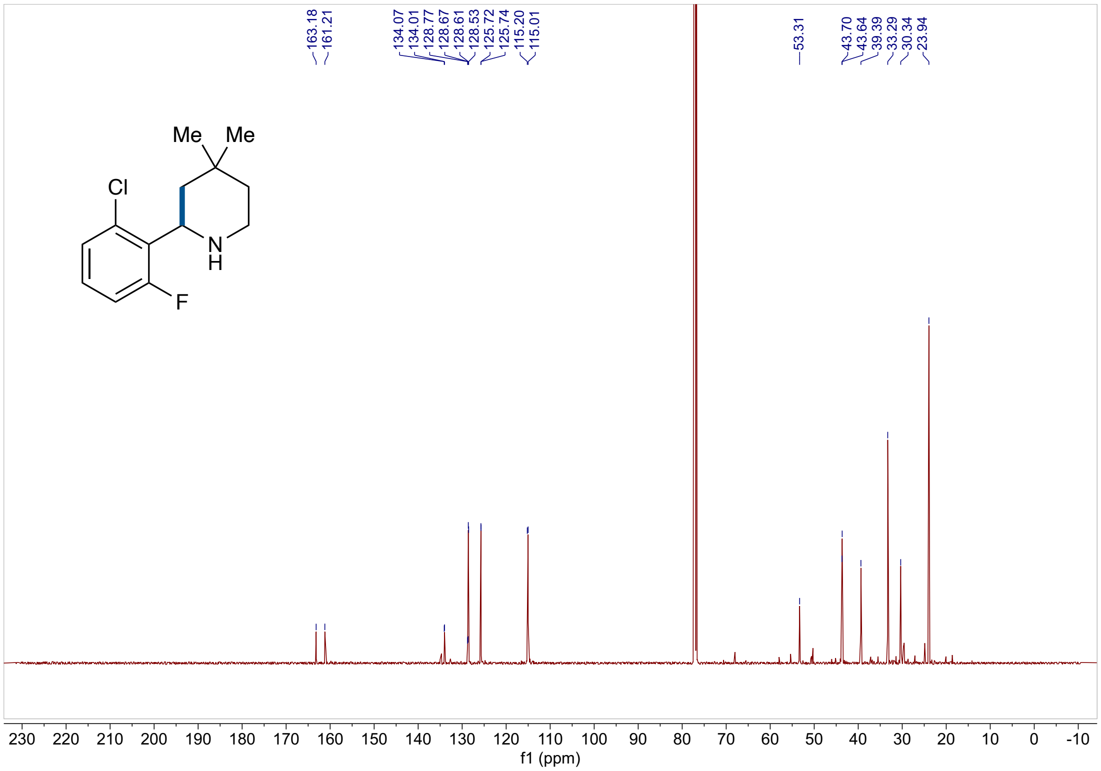

20 – ^{13}C NMR (126 MHz, CDCl$_3$)

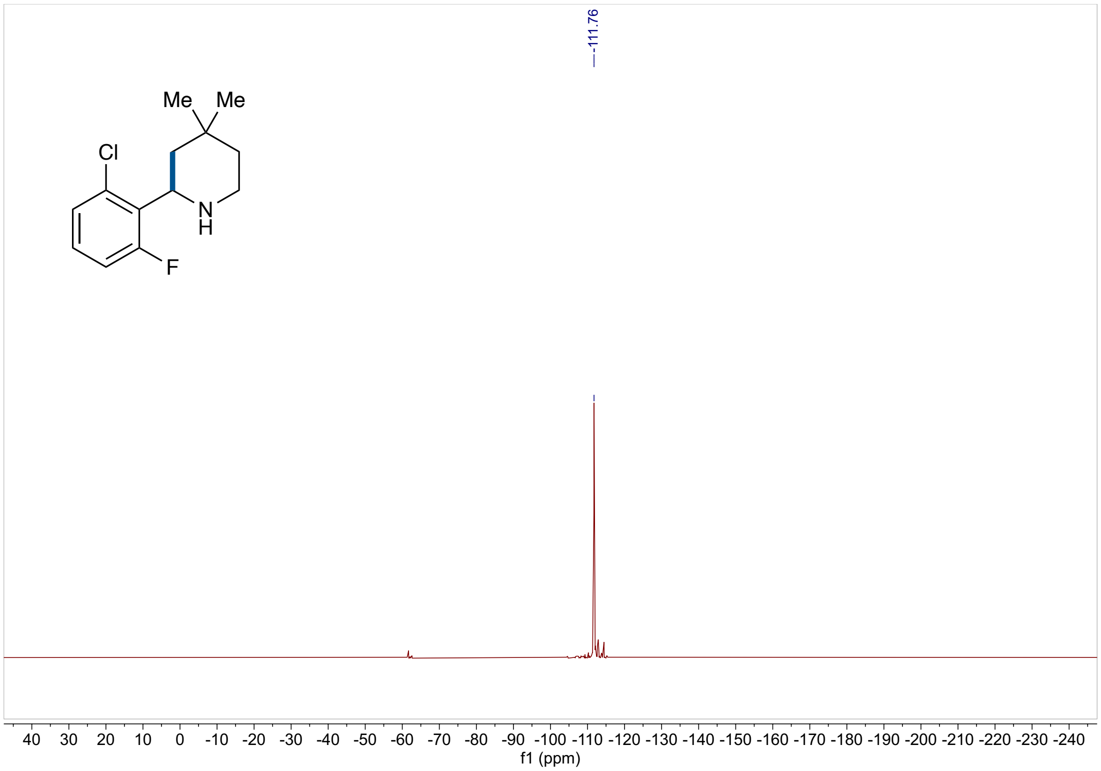

20 – ^{19}F NMR (471 MHz, $CDCl_3$)

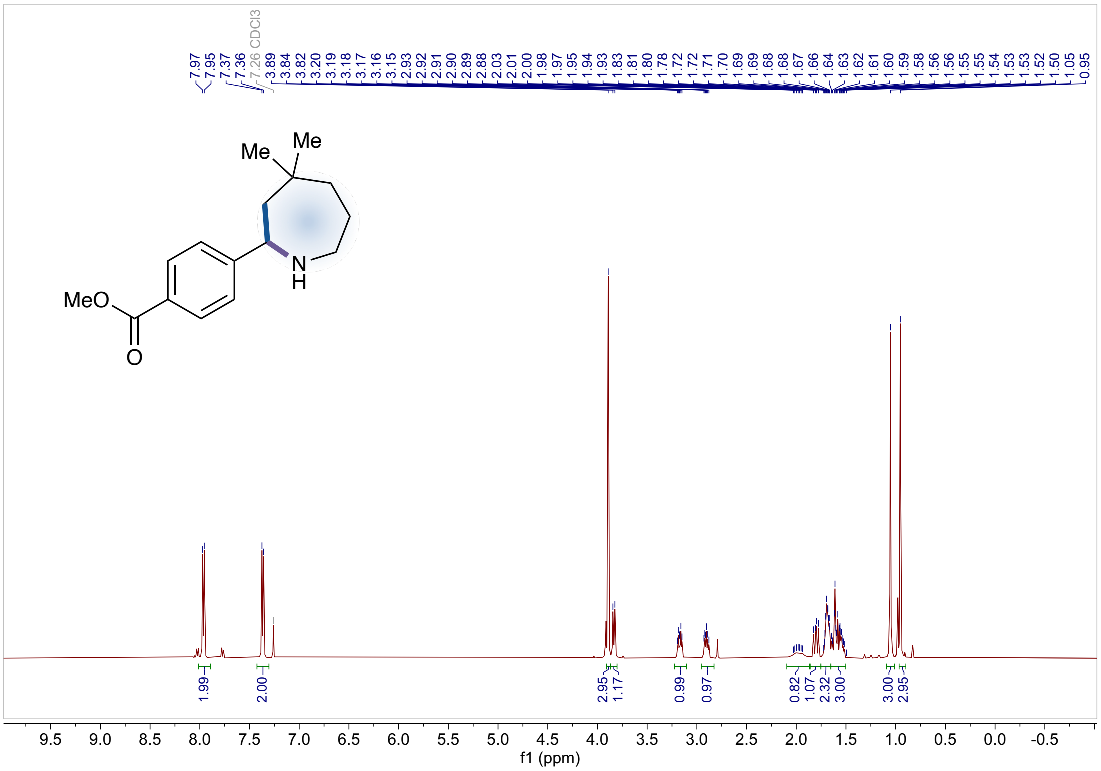

21 – ^{1}H NMR (500 MHz, CDCl$_3$)

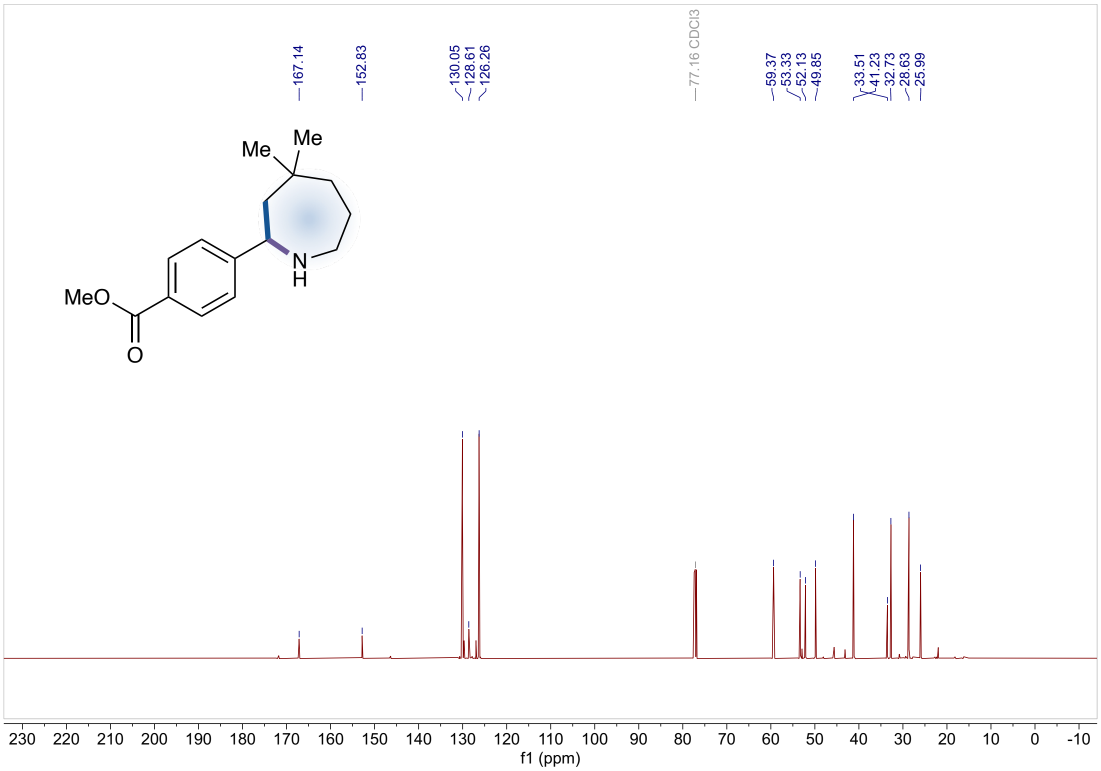

21 – ^{13}C NMR (126 MHz, CDCl$_3$

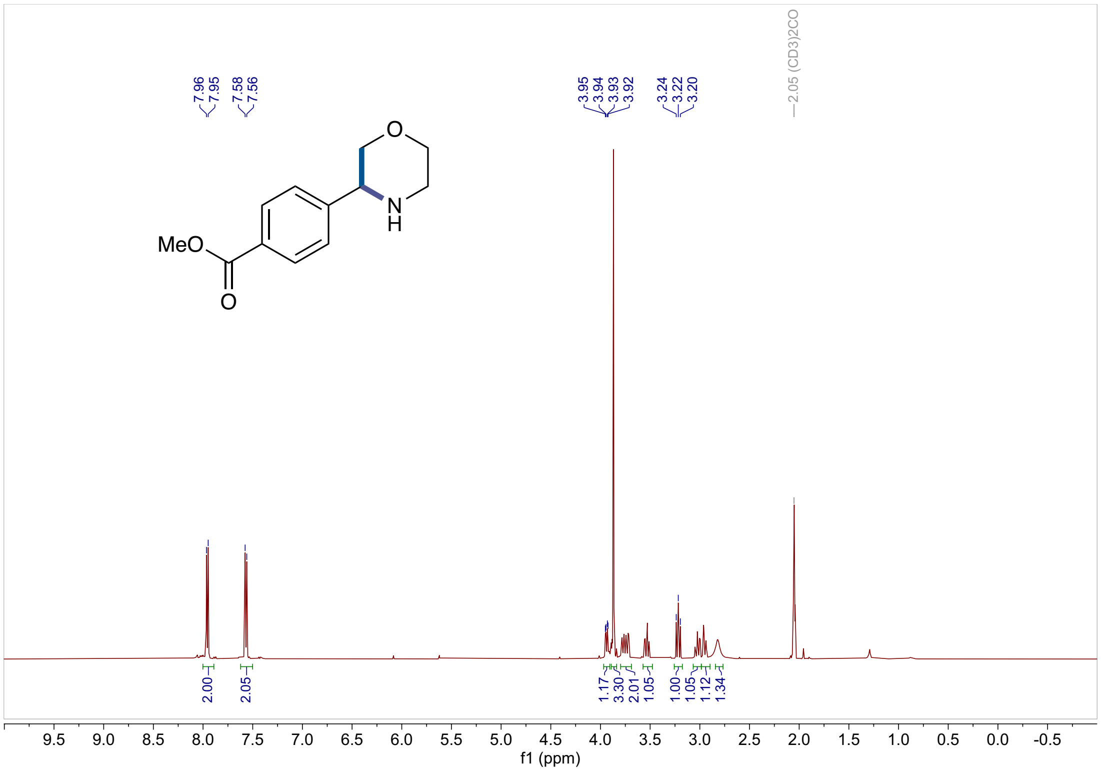

22 – ^{1}H NMR (500 MHz, Acetone)

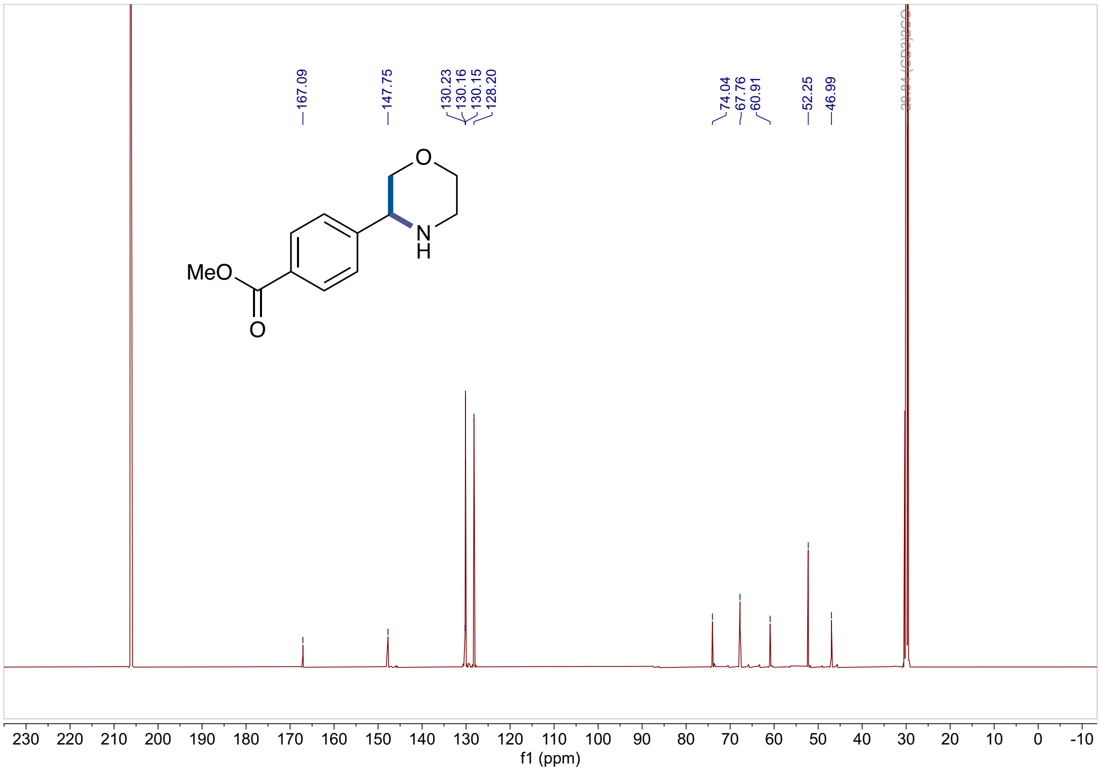

22 – ^{13}C NMR (126 MHz, Acetone)

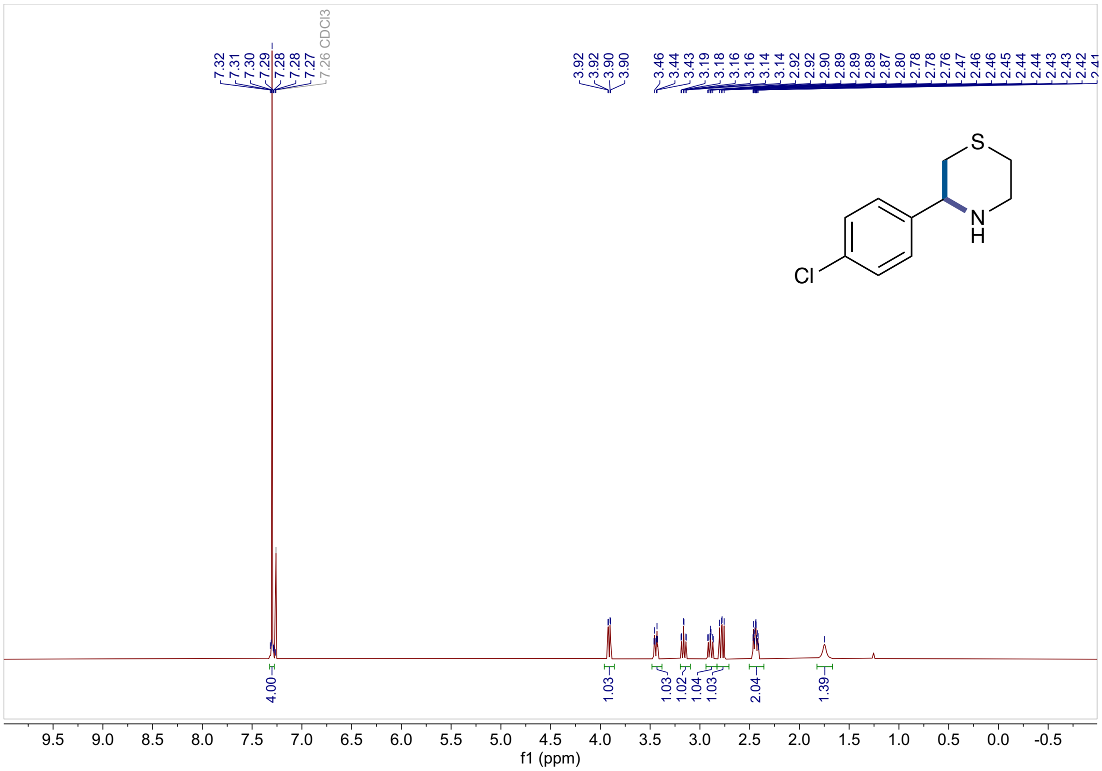

23 – ^{1}H NMR (500 MHz, CDCl$_3$)

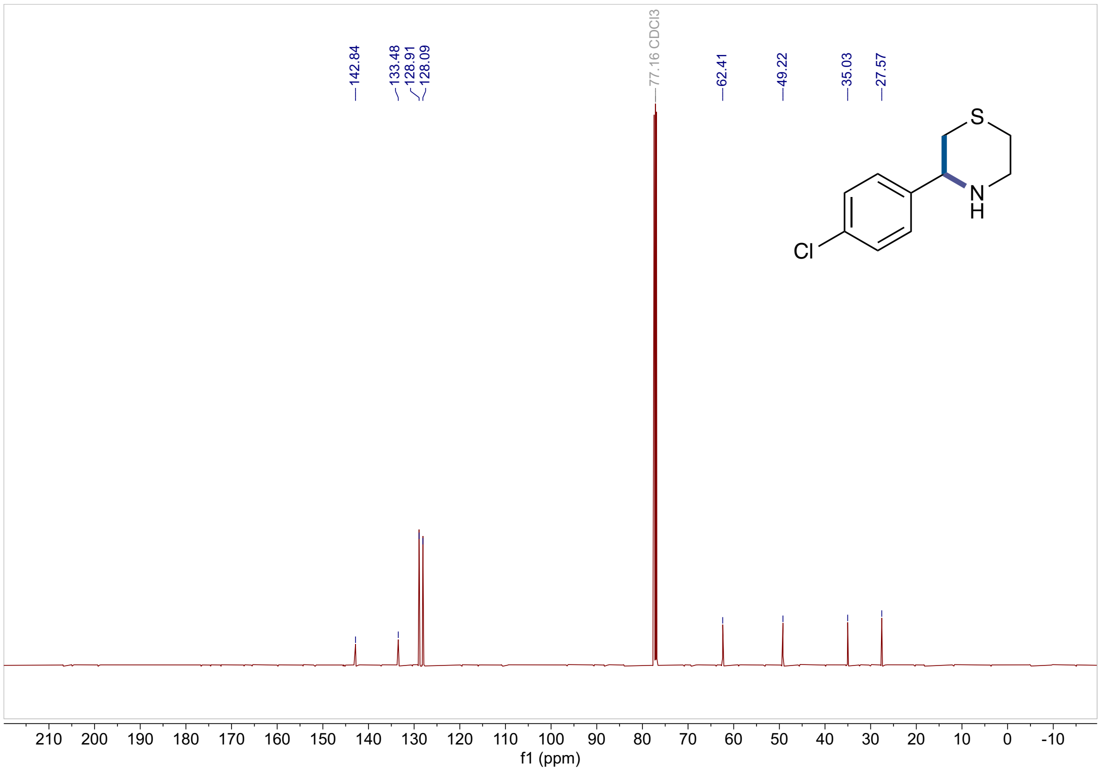

23 – ^{13}C NMR (126 MHz, CDCl$_3$)

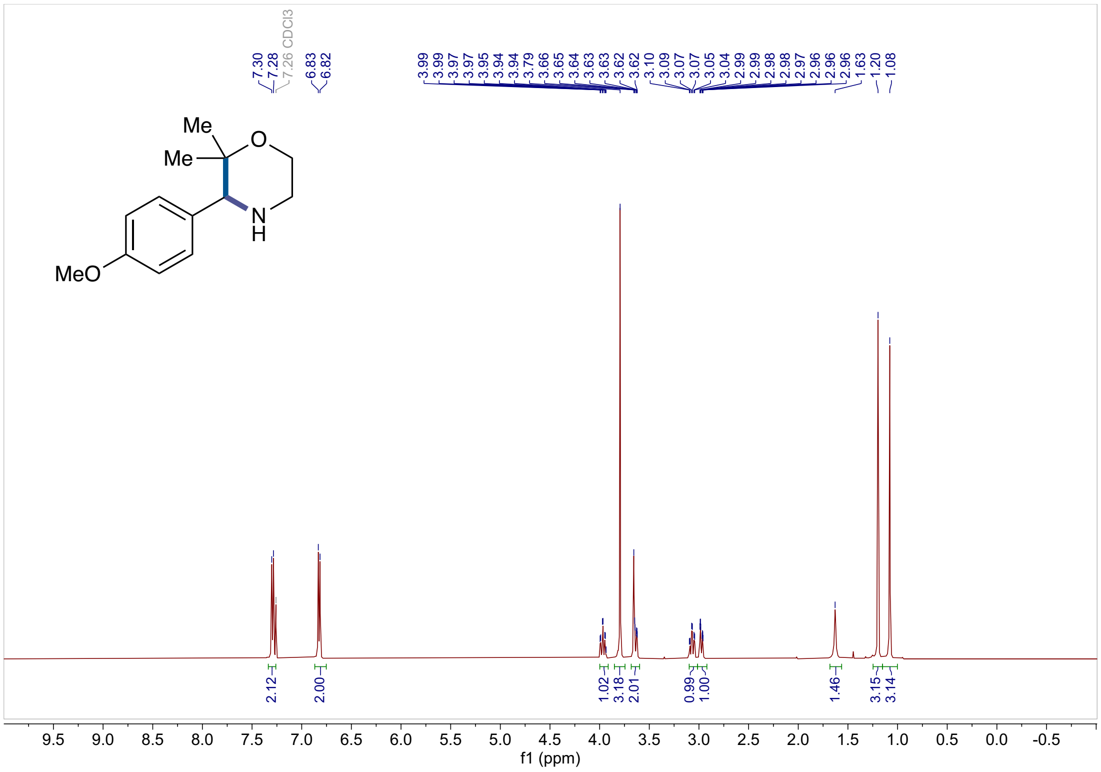

24 – ^{1}H NMR (500 MHz, $CDCl_3$)

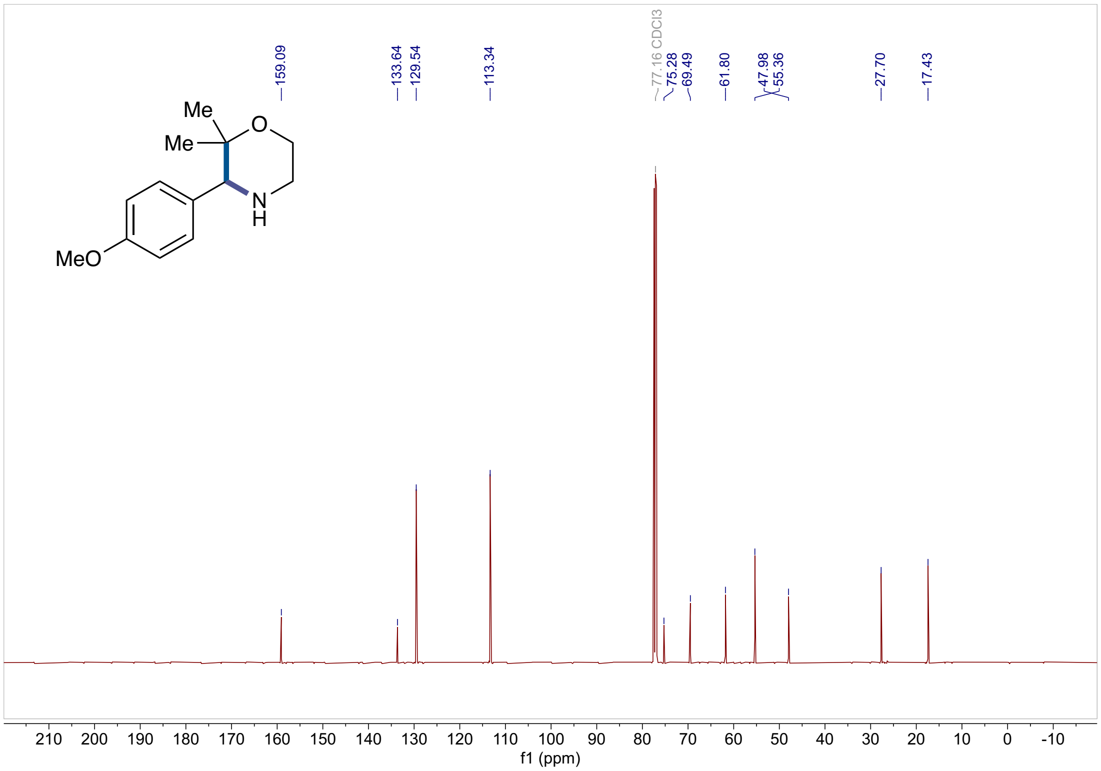

24 – ^{13}C NMR (126 MHz, $CDCl_3$)

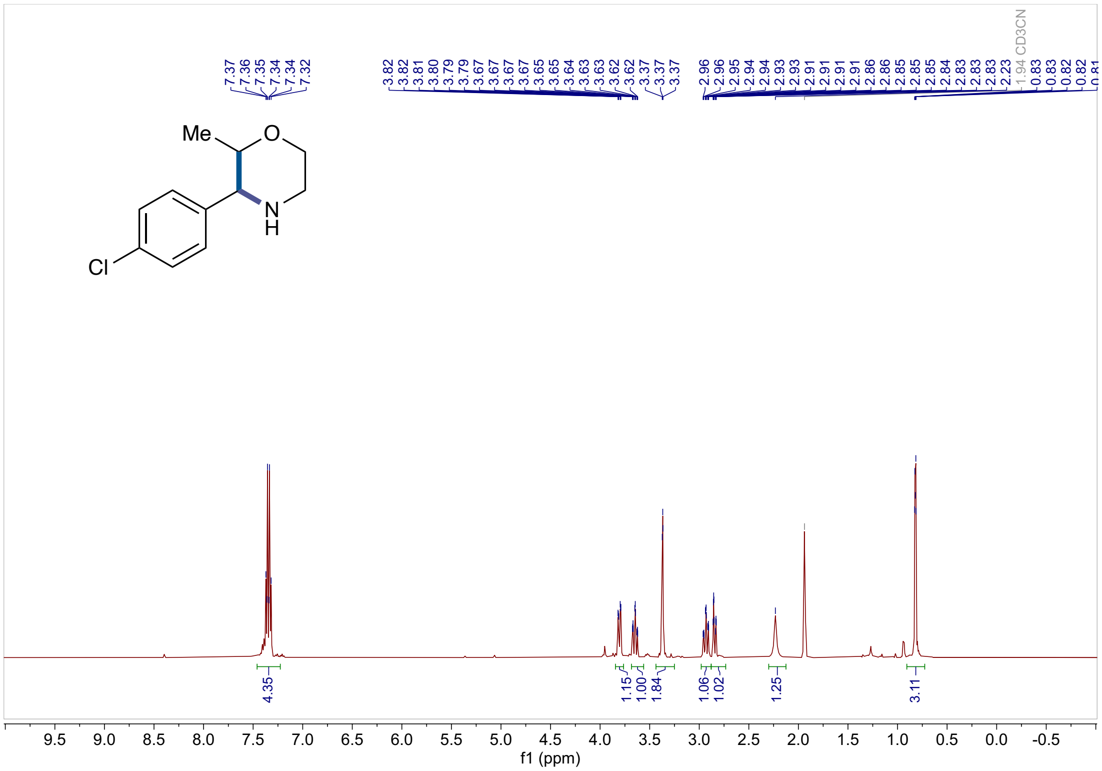

25 – ^{1}H NMR (500 MHz, CD_3CN)

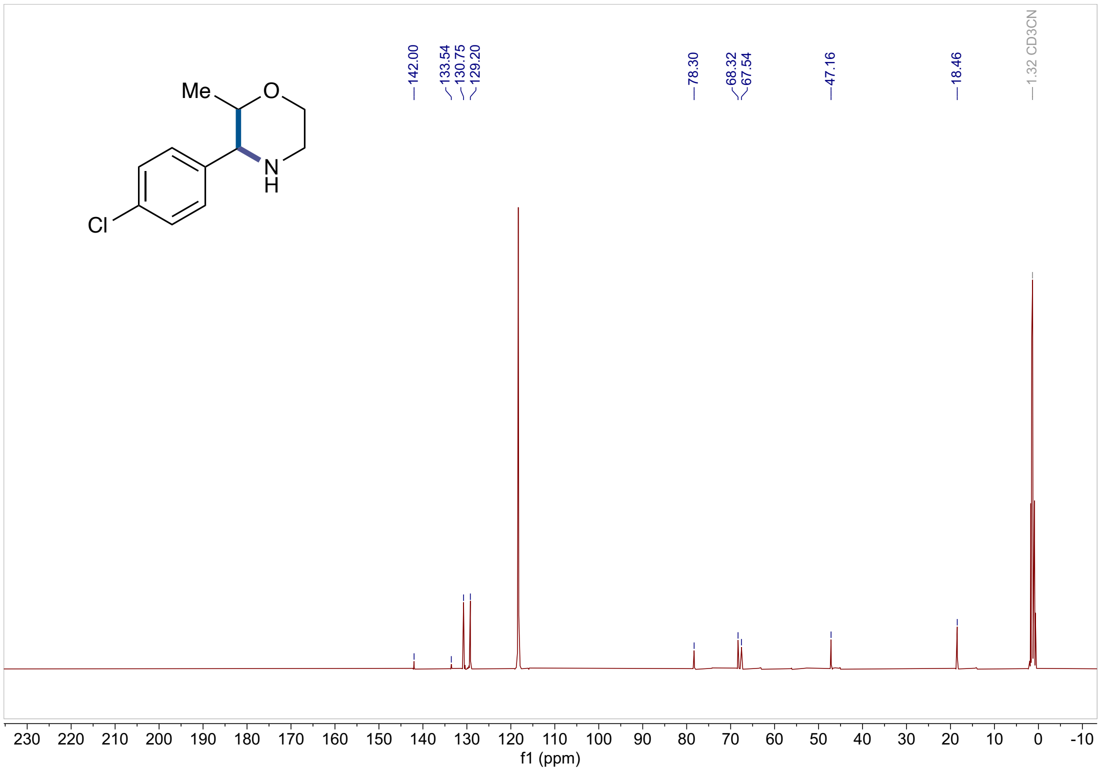

25 – ^{13}C NMR (101 MHz, CD_3CN)

Compound **25** – uHPLC analysis for d.r. determination

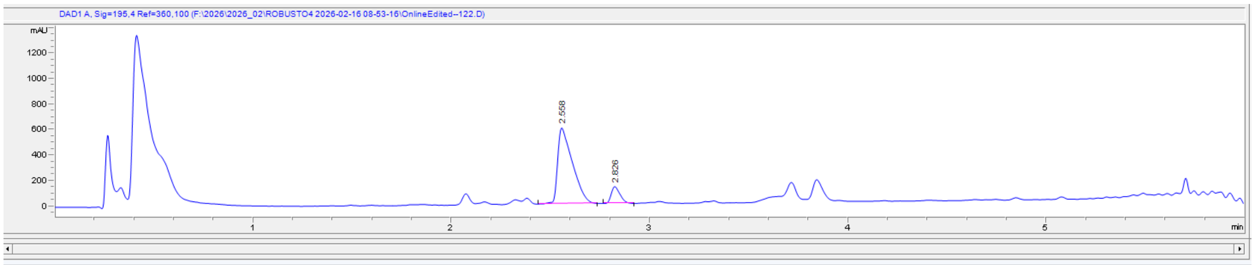

#	Time	Type	Area	Height	Width	Area%	Symmetry
1	2.558	BB	3104.3	598.3	0.0715	88.265	0.365
2	2.826	BB	412.7	133.1	0.0462	11.735	0.639

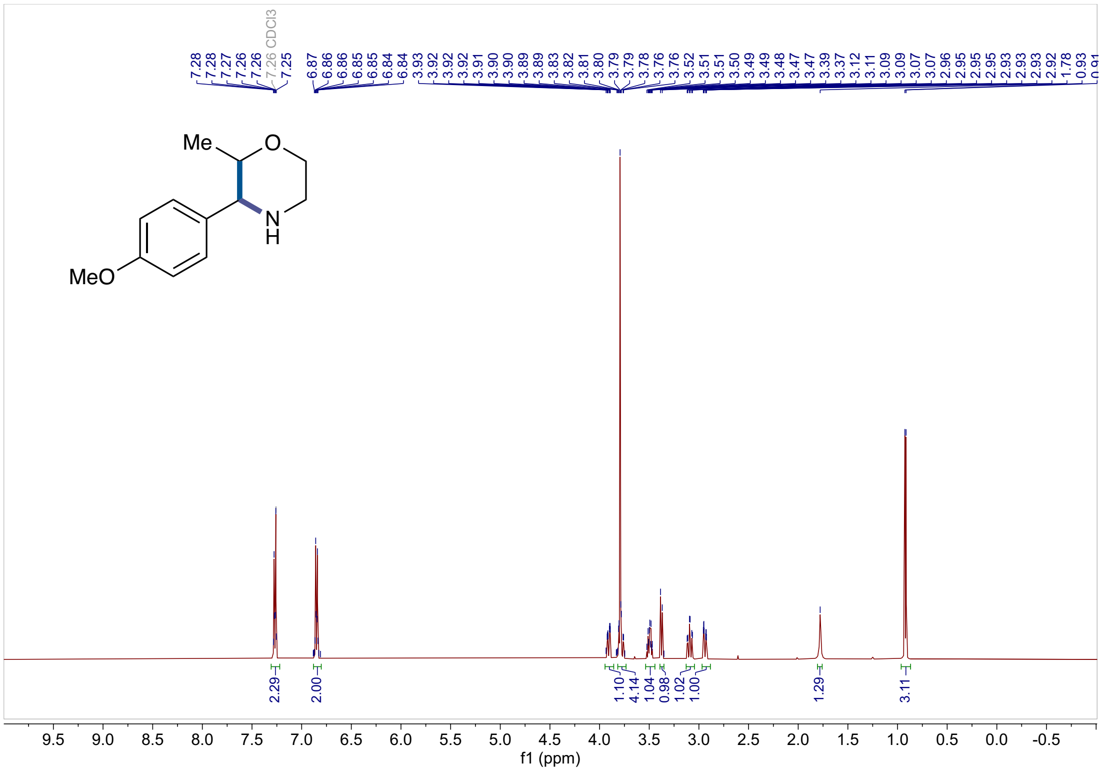

26 – ^{1}H NMR (500 MHz, CDCl$_3$)

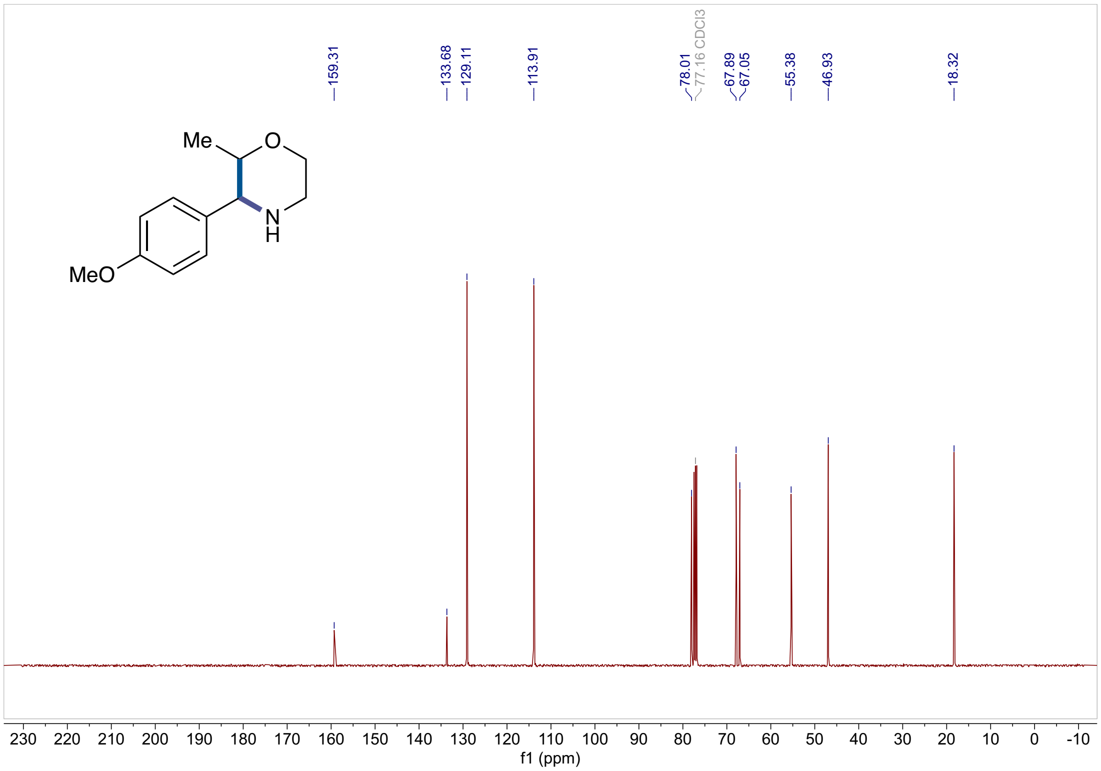

26 – ^{13}C NMR (101 MHz, CDCl$_3$)

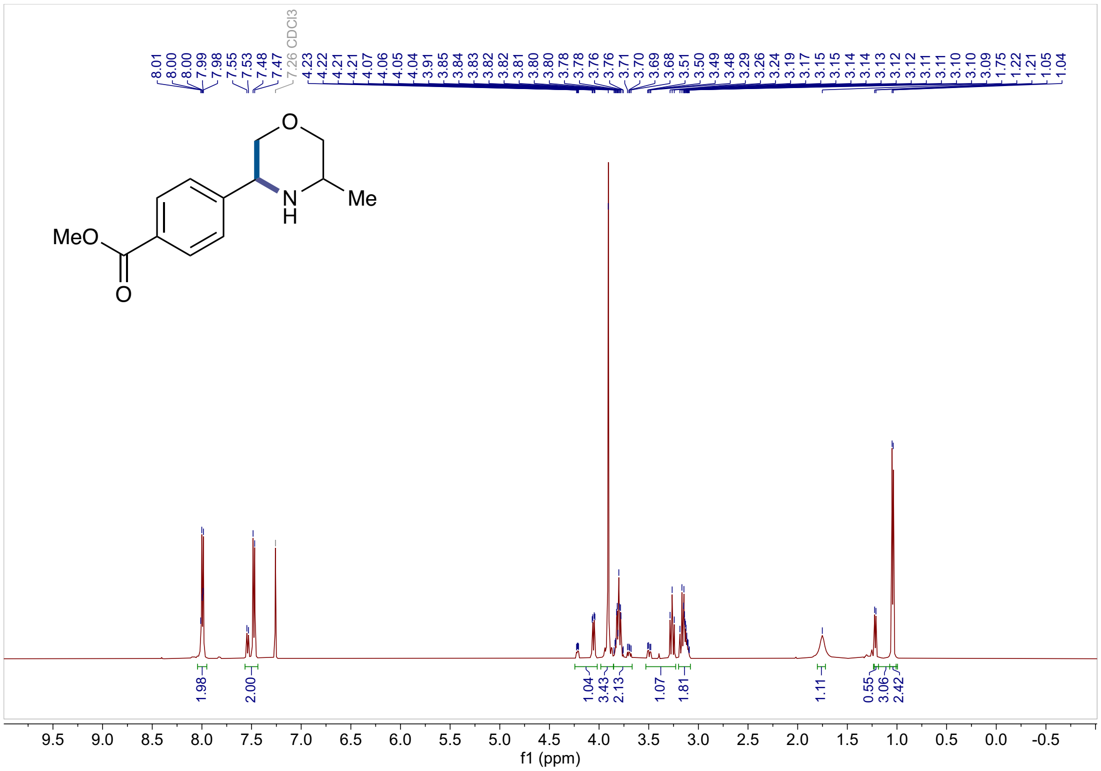

27 – ^{1}H NMR (500 MHz, $CDCl_3$)

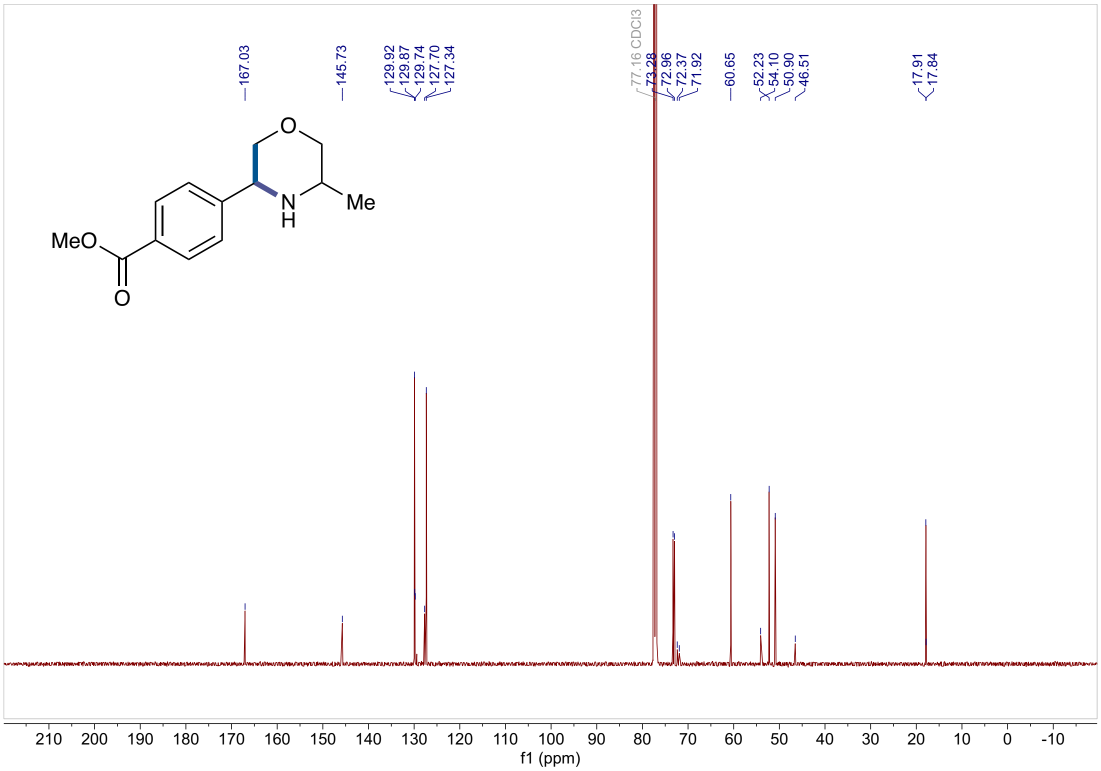

27 – ^{13}C NMR (126 MHz, $CDCl_3$)

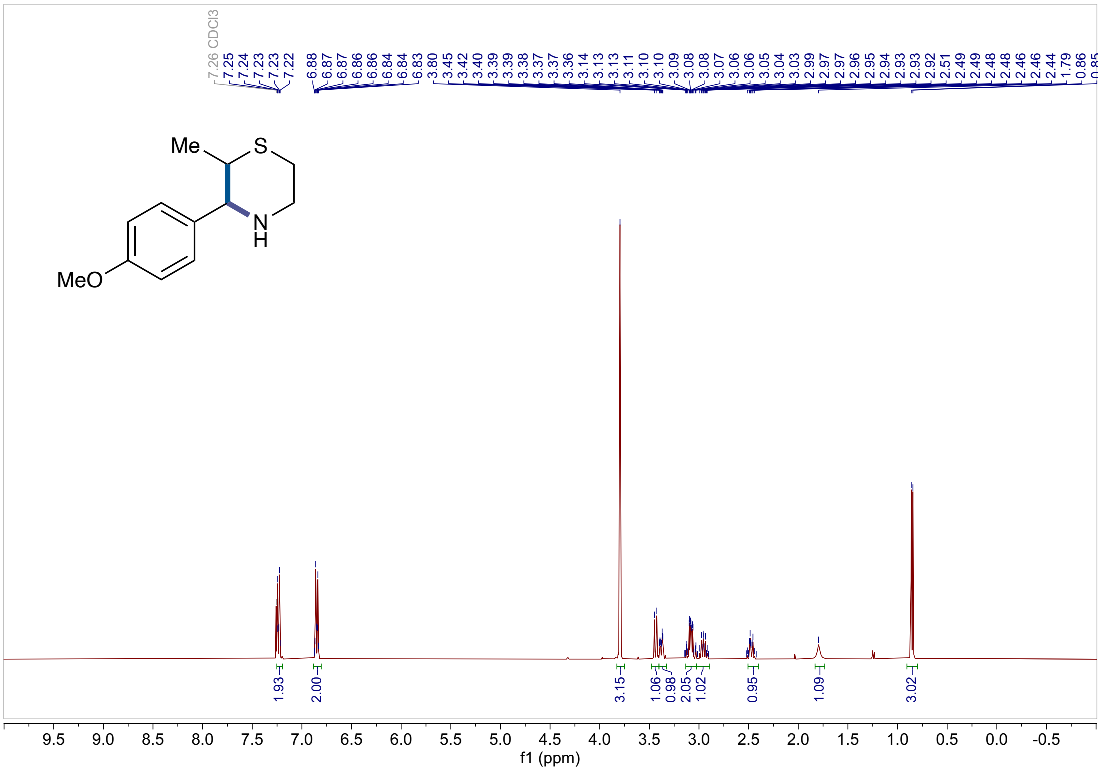

28 – ^{1}H NMR (400 MHz, CDCl$_3$)

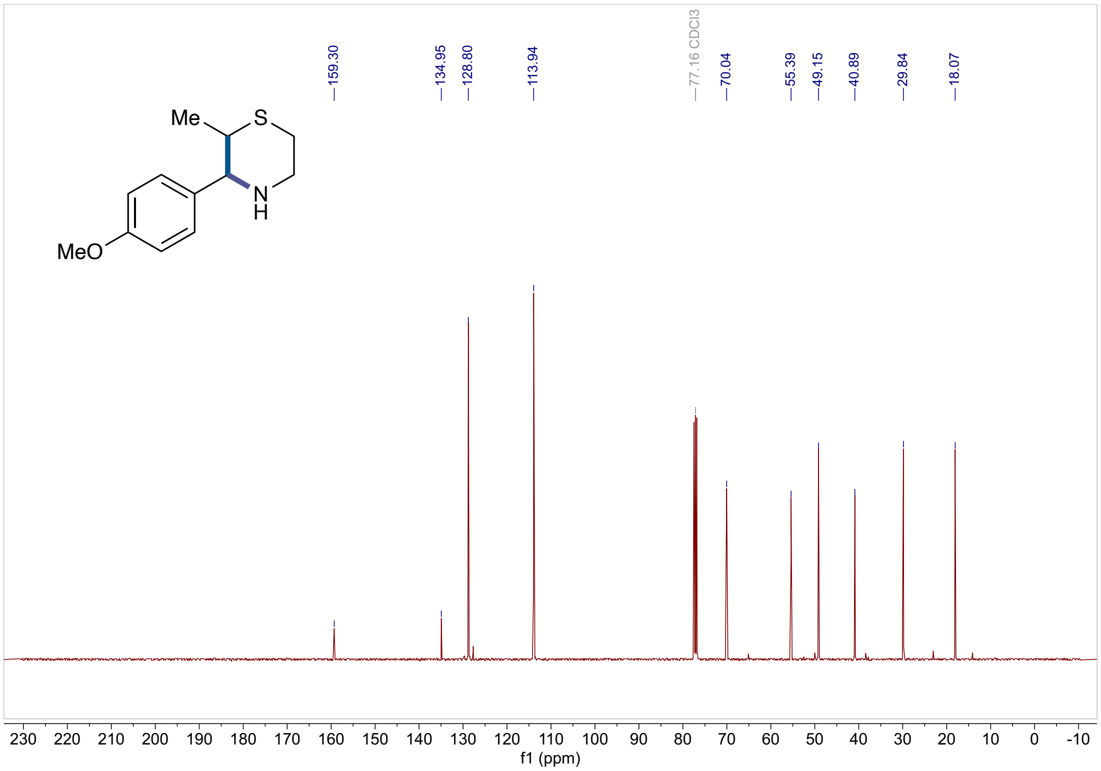

28 – ^{13}C NMR (101 MHz, CDCl$_3$)

Compound **28** – COSY analysis for major diastereomer determination

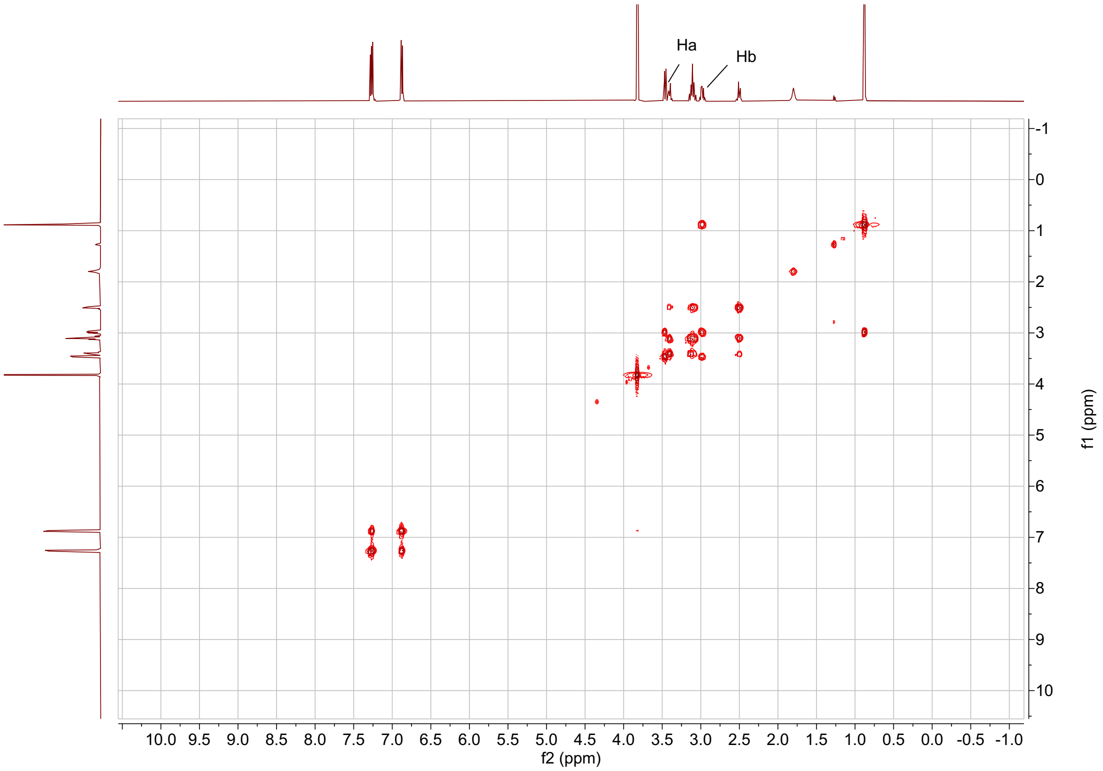

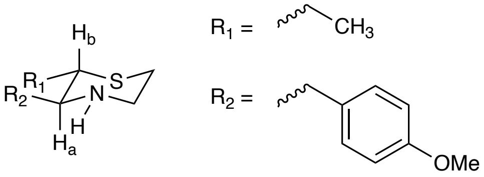

^{1}H NMR (400 MHz, CDCl3) and COSY

δ 3.46 H_a (d, 9.3 Hz)

δ 2.98 H_b (td, 9.3, 6.8 Hz)

based on *J*-coupling of H_a and H_b, major diastereomer is *trans*

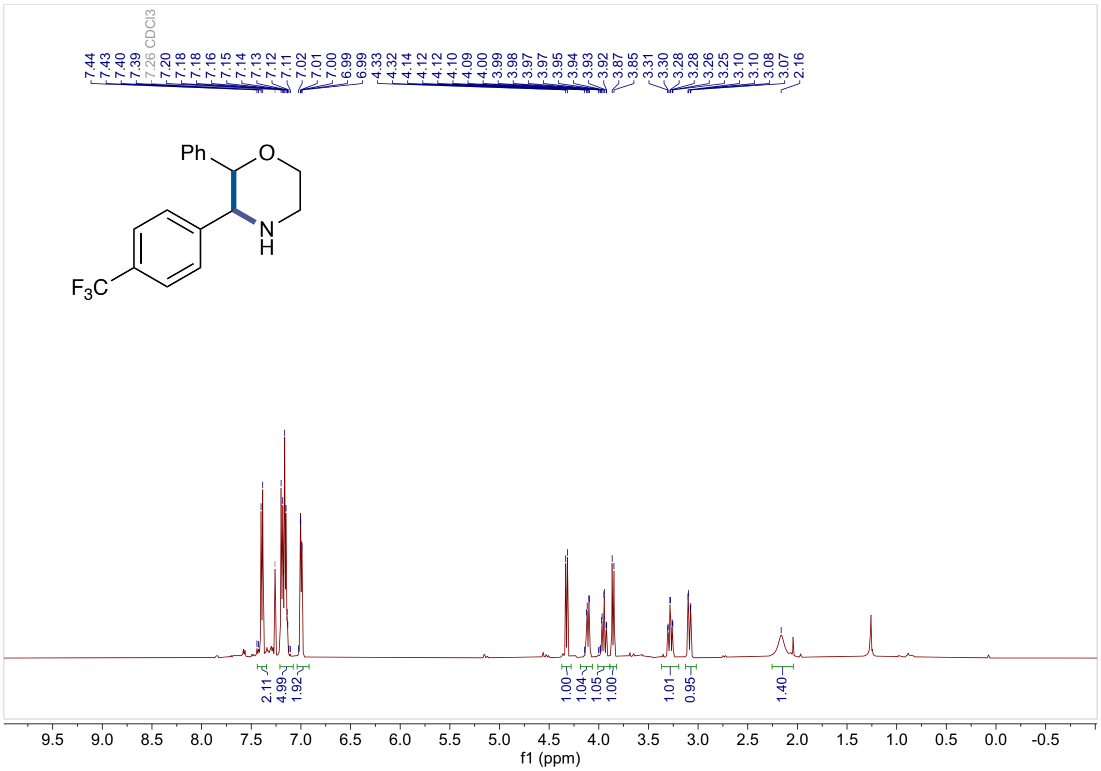

29 – ^{1}H NMR (500 MHz, $CDCl_3$)

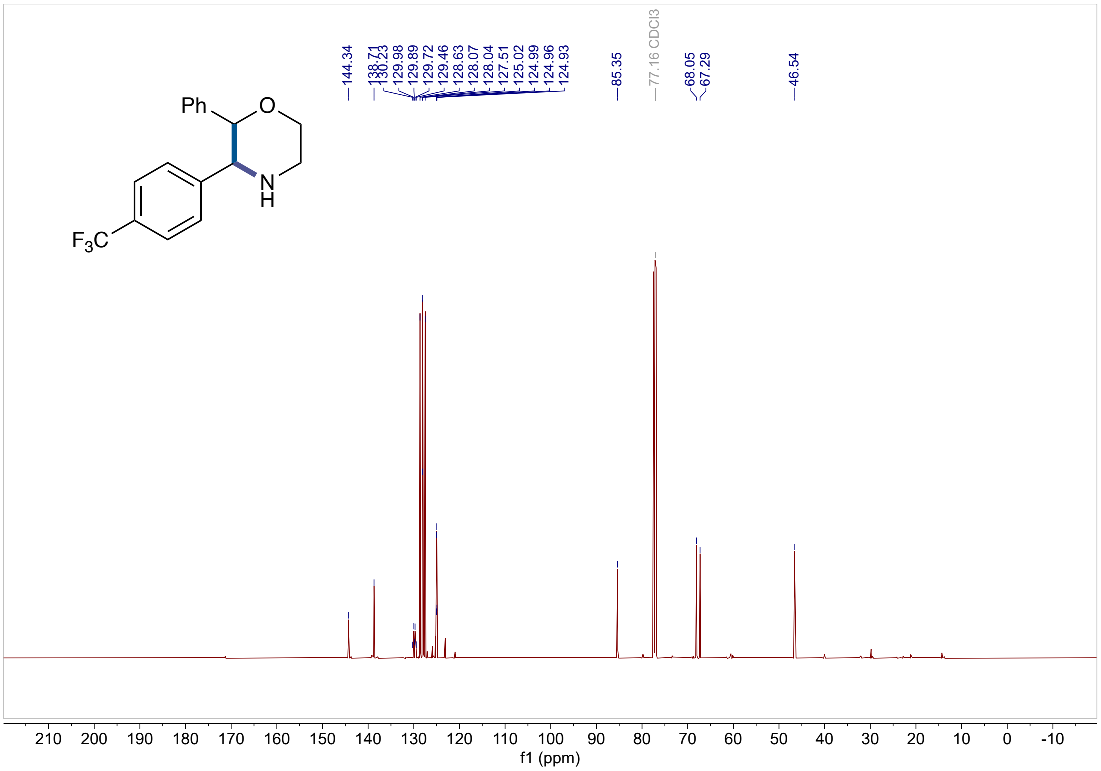

29 – ^{13}C NMR (126 MHz, $CDCl_3$)

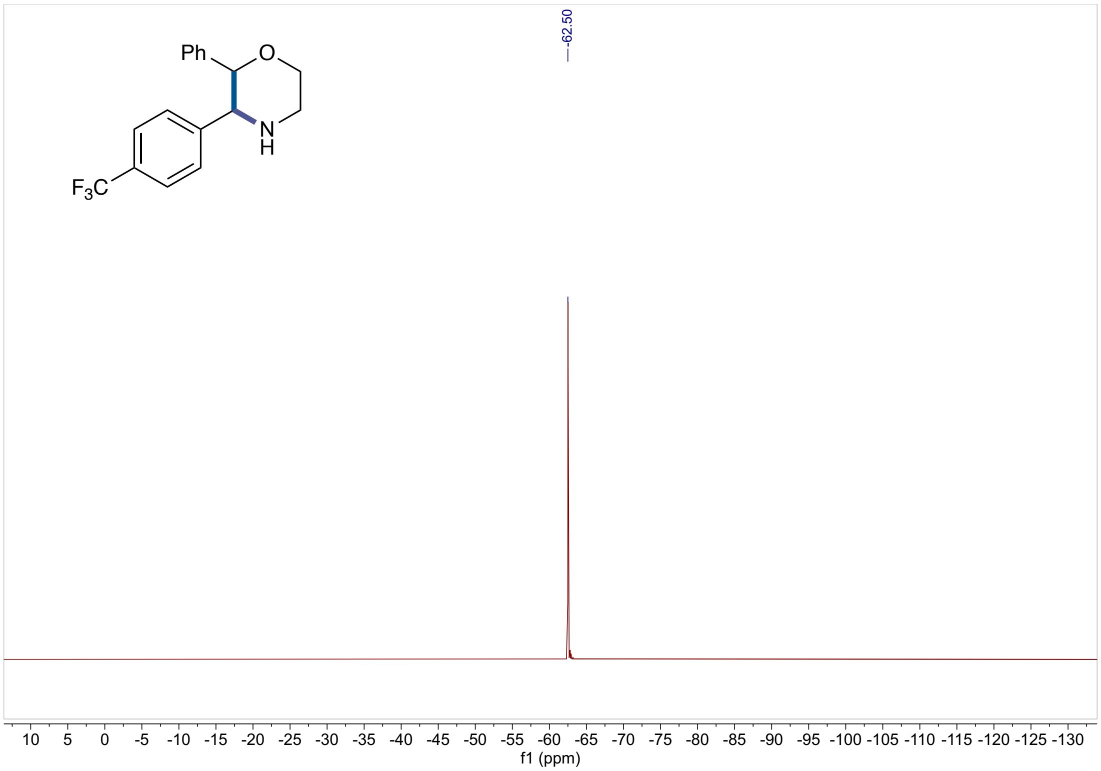

29 – ^{19}F NMR (471 MHz, $CDCl_3$)

Compound **29** – uHPLC analysis for d.r. determination

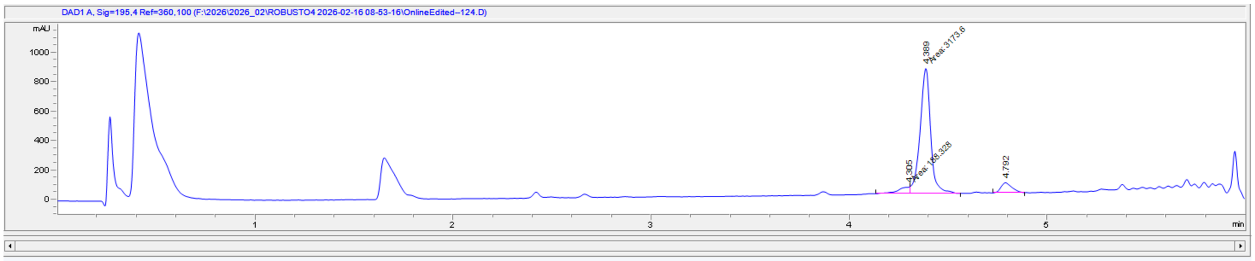

#	Time	Type	Area	Height	Width	Area%	Symmetry
1	4.305	MF	188.3	45.3	0.0692	5.191	1726.67
2	4.389	FM	3173.6	852.6	0.062	87.483	1.135
3	4.792	BB	265.8	68.4	0.0573	7.326	0.68

Compound **29** – COSY analysis for major diastereomer determination

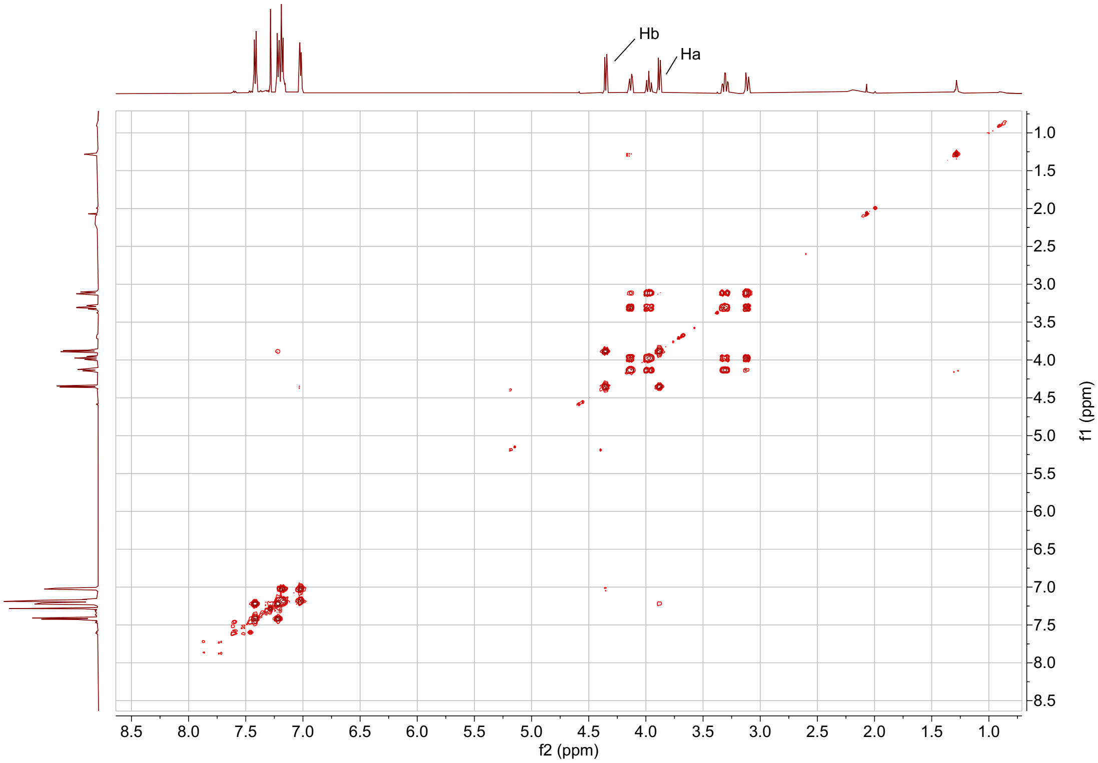

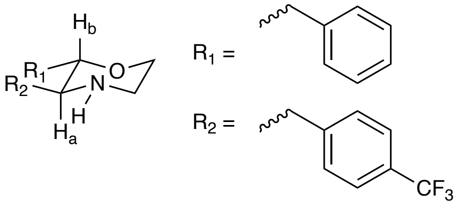

^{1}H NMR (500 MHz, CDCl3) and COSY

δ 3.88 H_a (d, 8.8 Hz)

δ 4.35 H_b (d, 8.7 Hz)

based on *J*-coupling of H_a and H_b, major diastereomer is *trans*

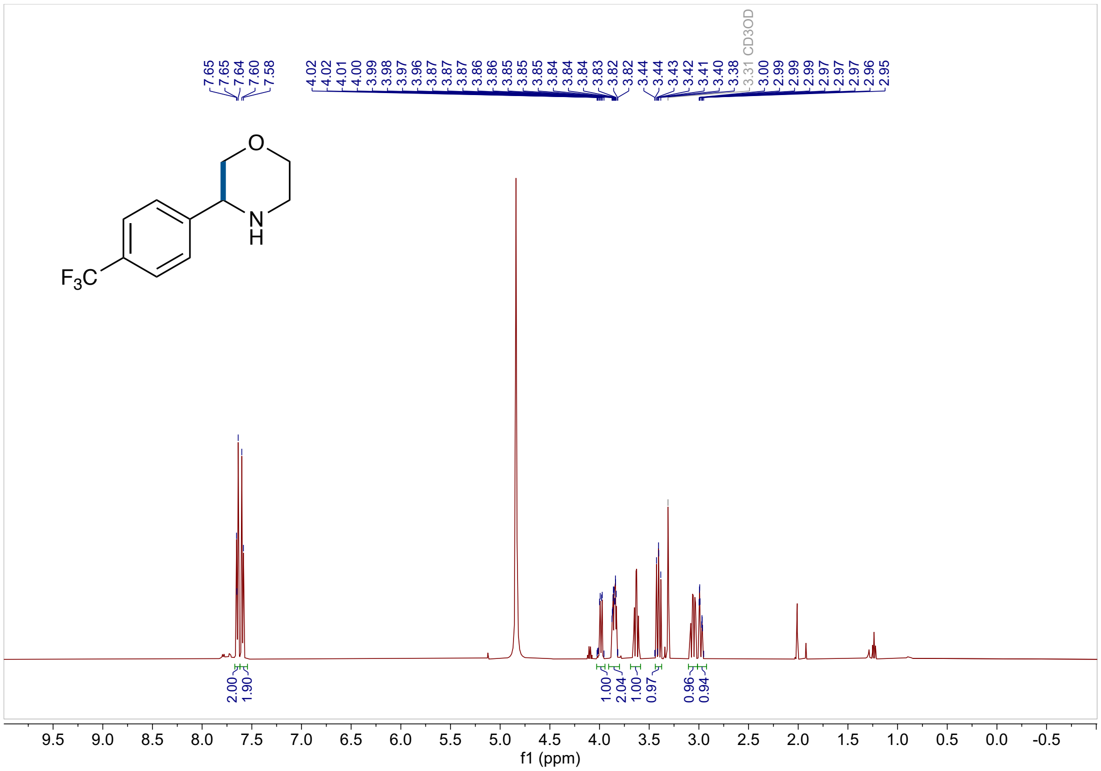

30 – ^{1}H NMR (500 MHz, MeOD)

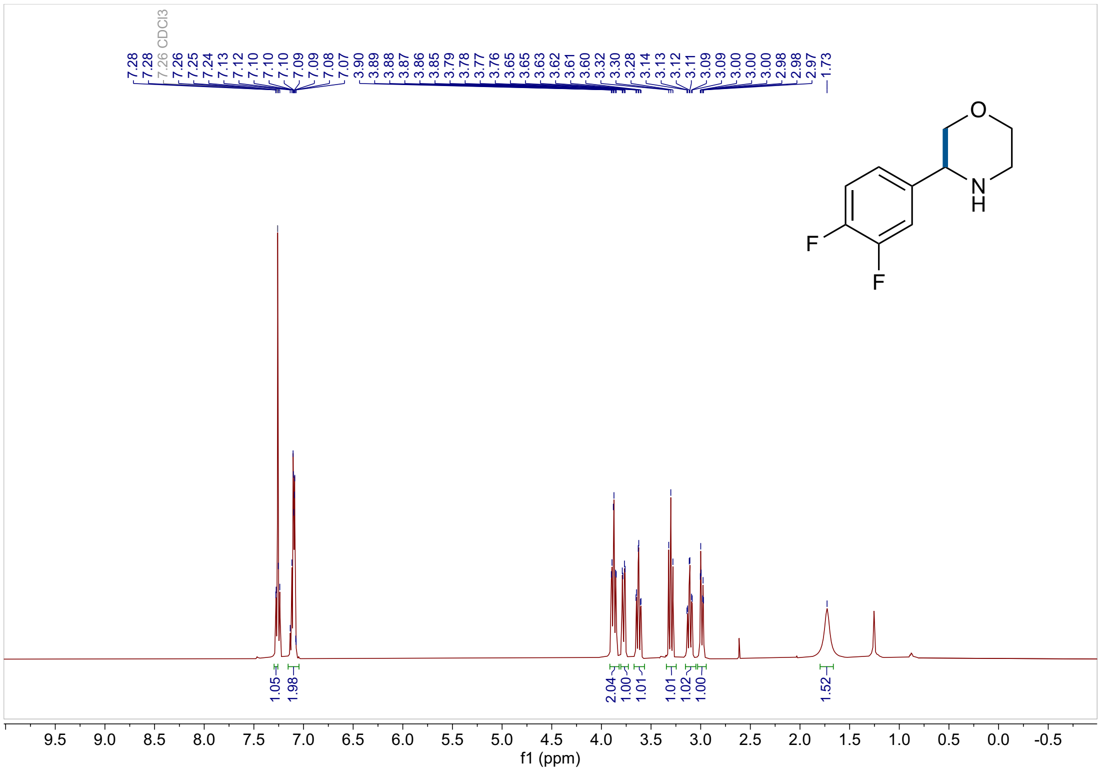

31 – ^{1}H NMR (500 MHz, $CDCl_3$)

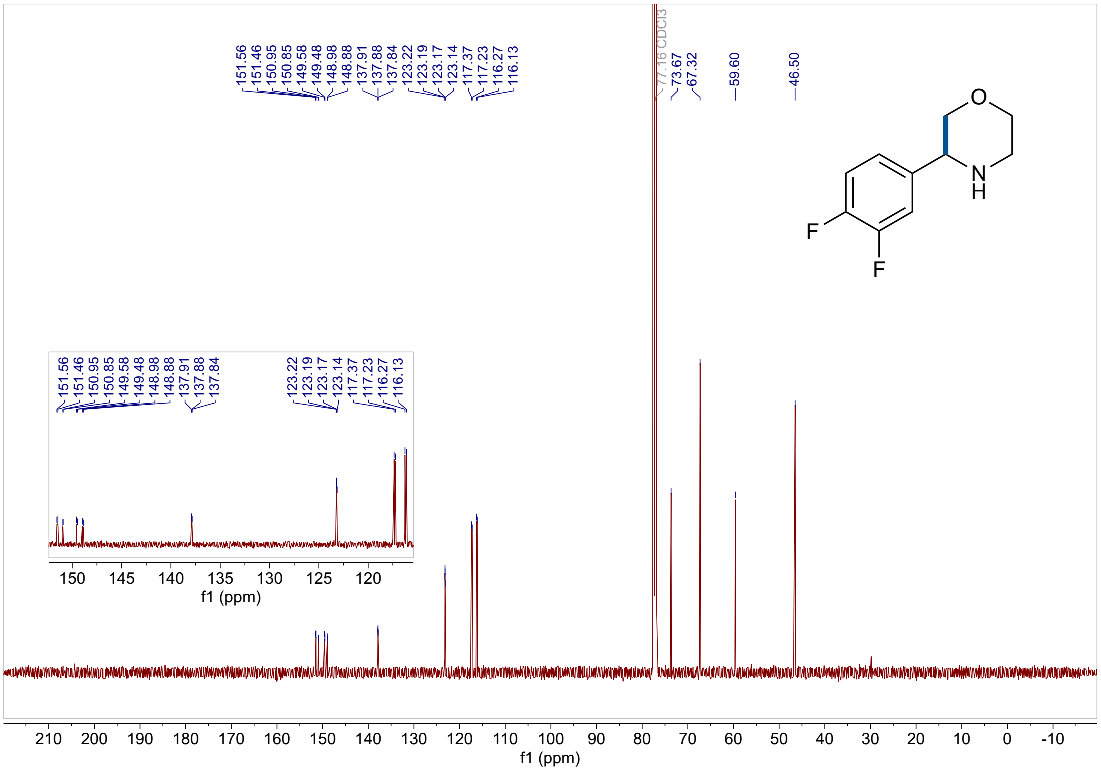

31 – ^{13}C NMR (126 MHz, $CDCl_3$)

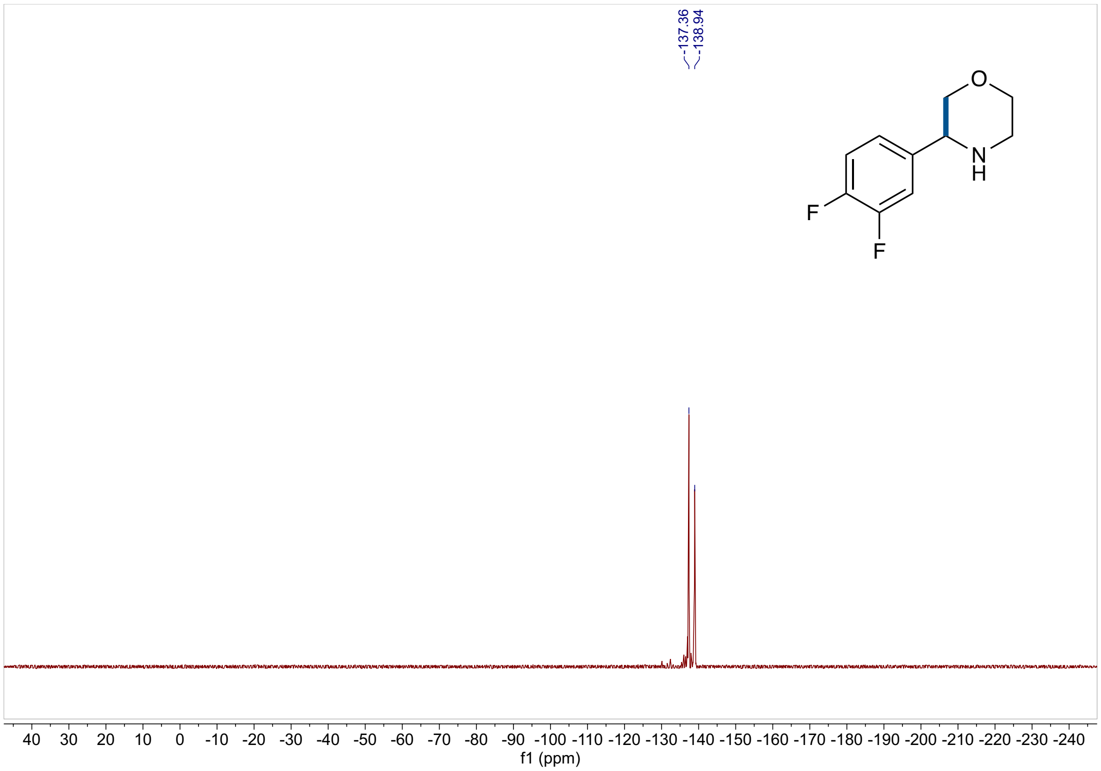

31 – ^{19}F NMR (471 MHz, $CDCl_3$)

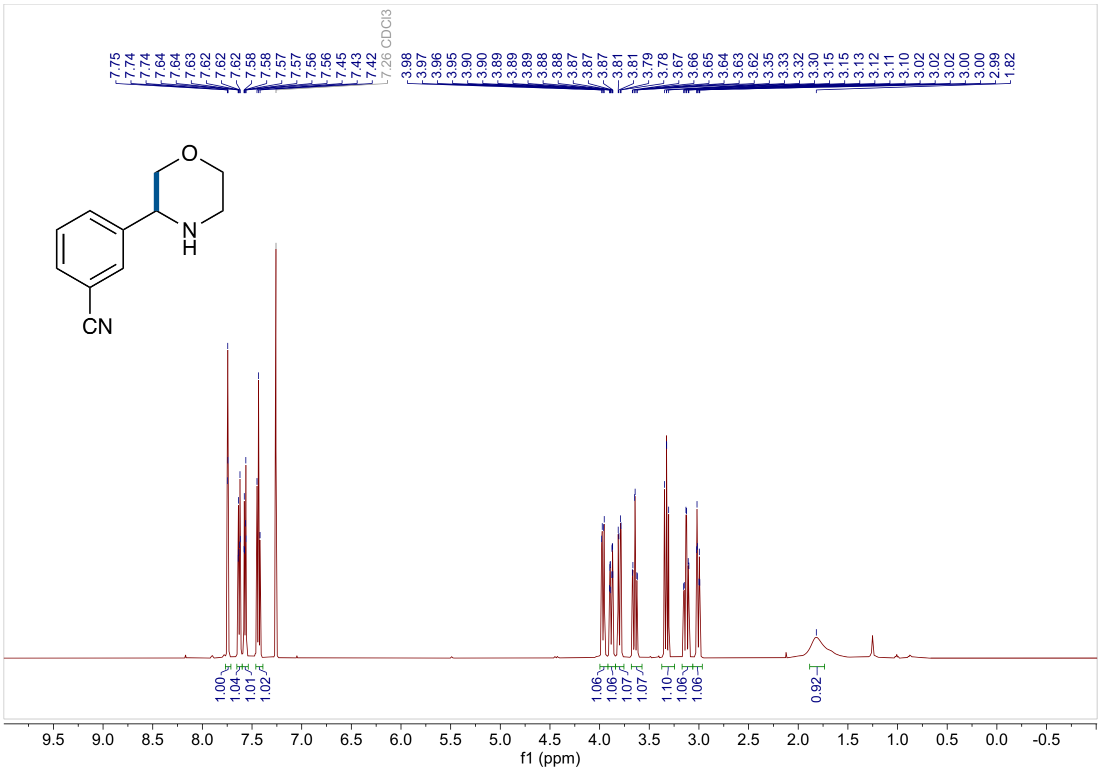

32 – ^{1}H NMR (500 MHz, $CDCl_3$)

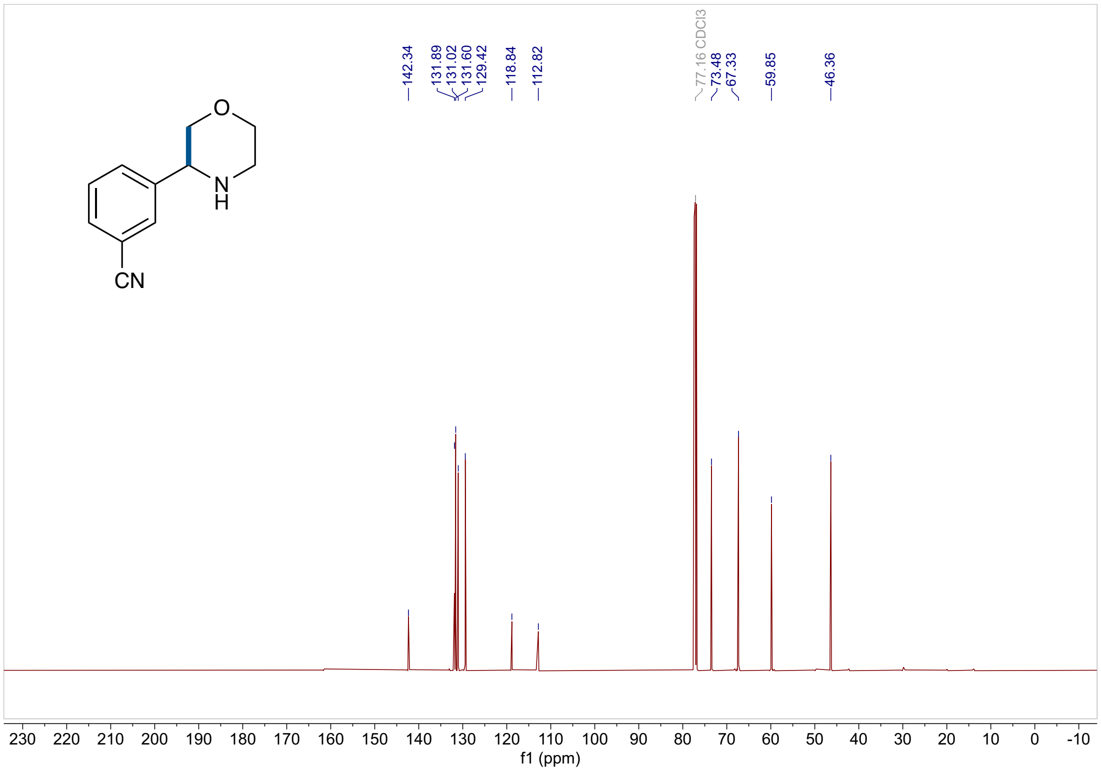

32 – ^{13}C NMR (126 MHz, $CDCl_3$)

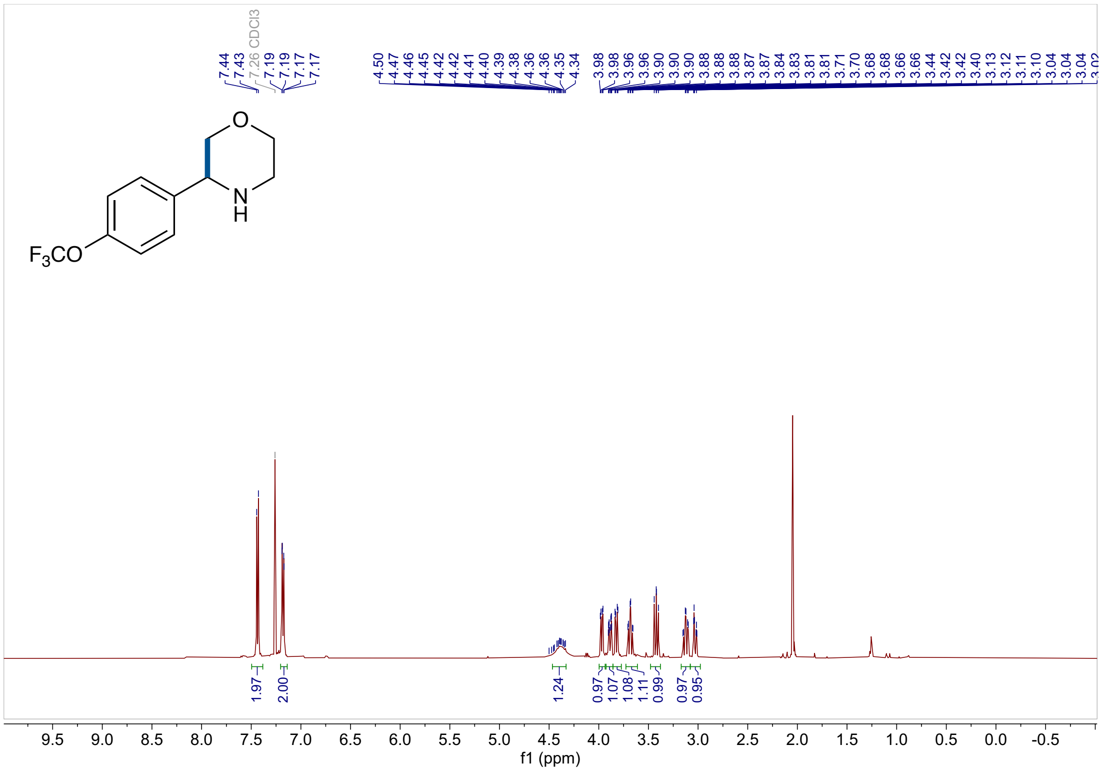

33 – ^{1}H NMR (500 MHz, CDCl$_3$)

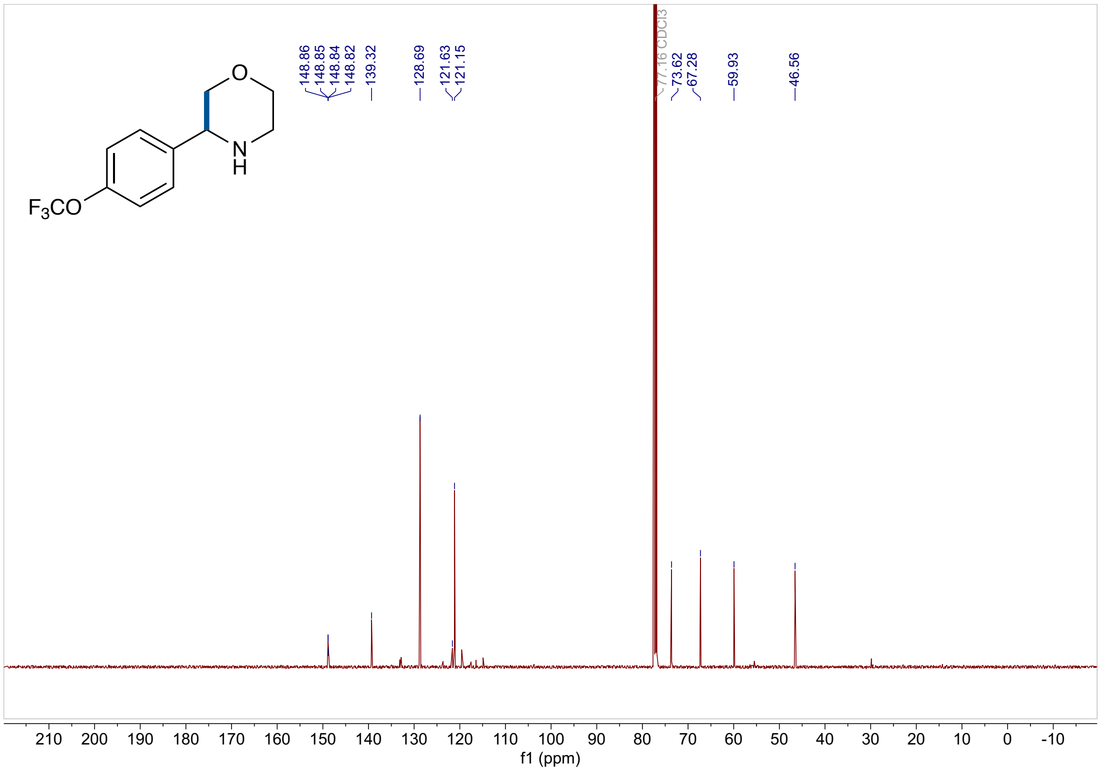

33 – ^{13}C NMR (126 MHz, CDCl$_3$)

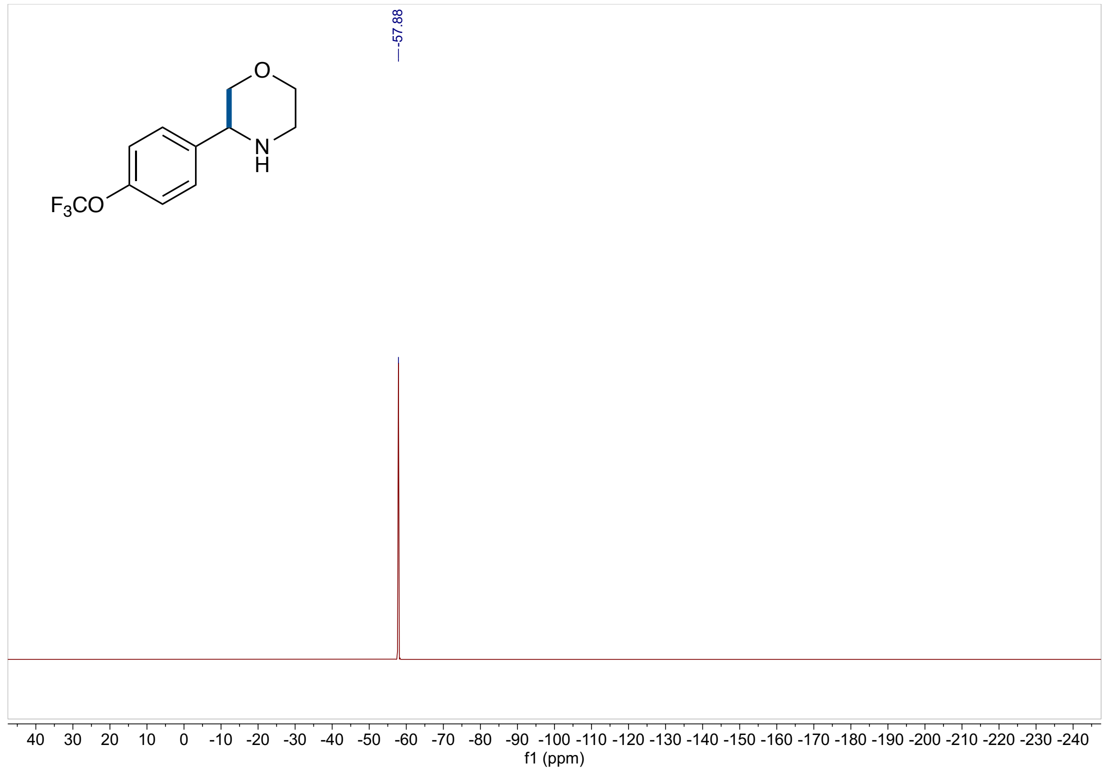

33 – ^{19}F NMR (471 MHz, $CDCl_3$)

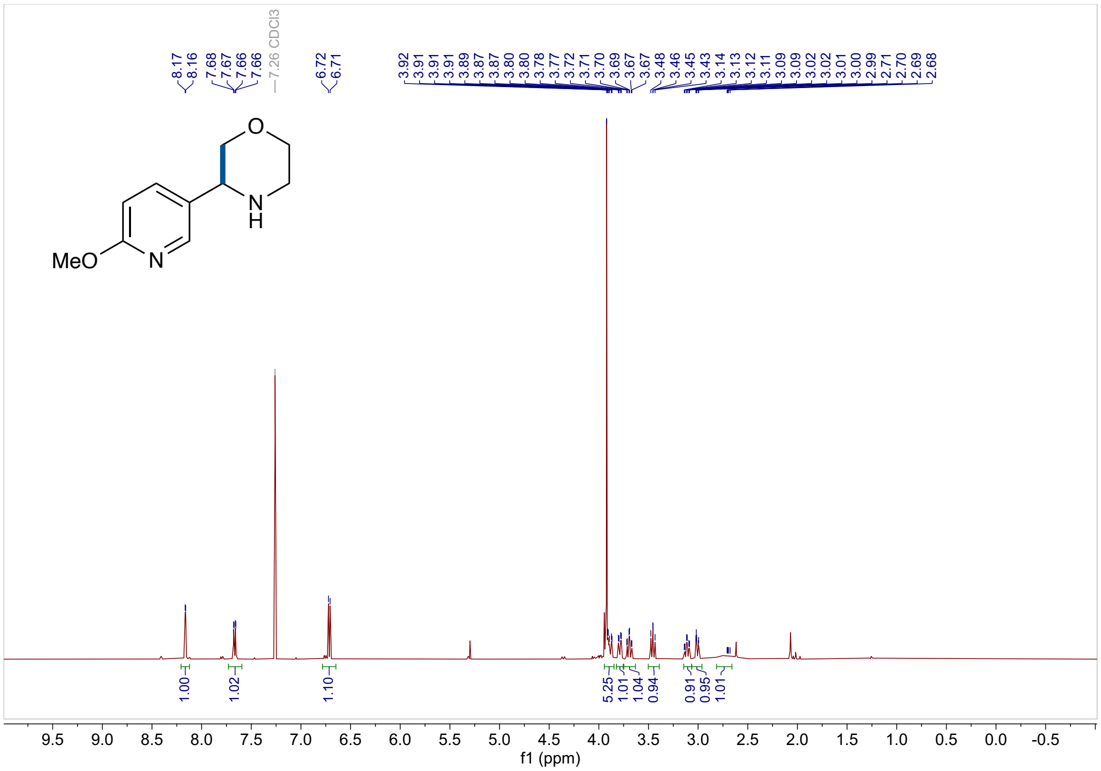

34 – ^{1}H NMR (500 MHz, CDCl$_3$)

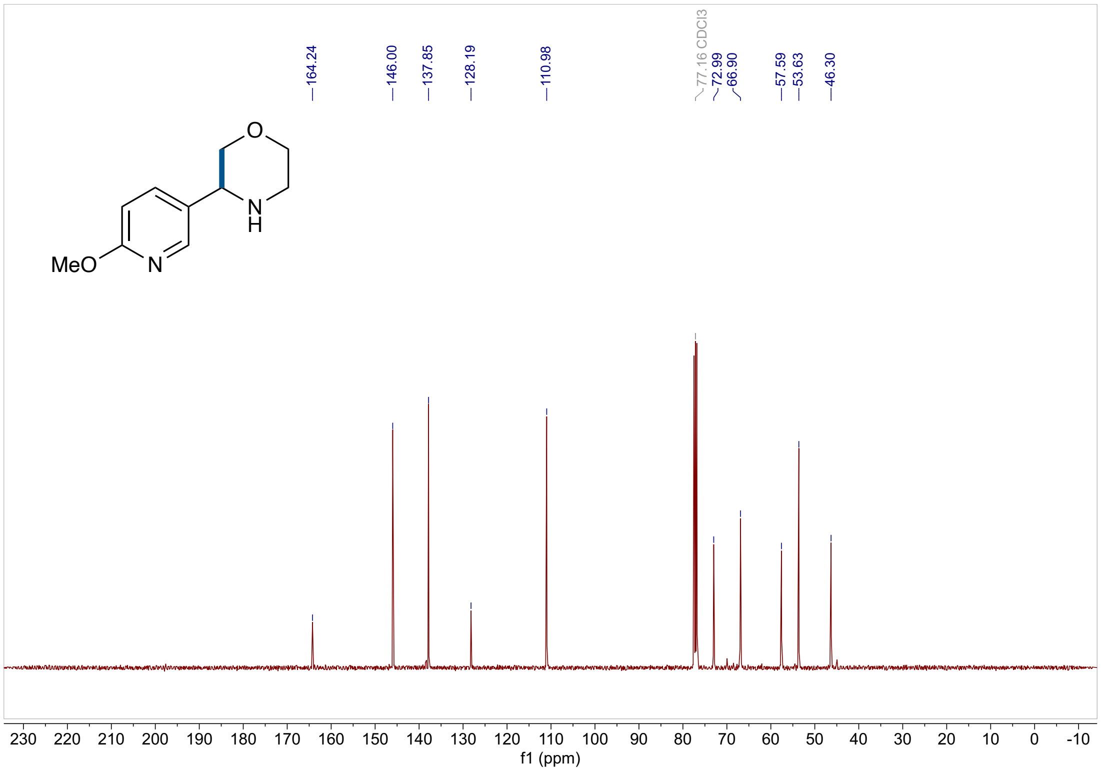

34 – ^{13}C NMR (101 MHz, CDCl$_3$)

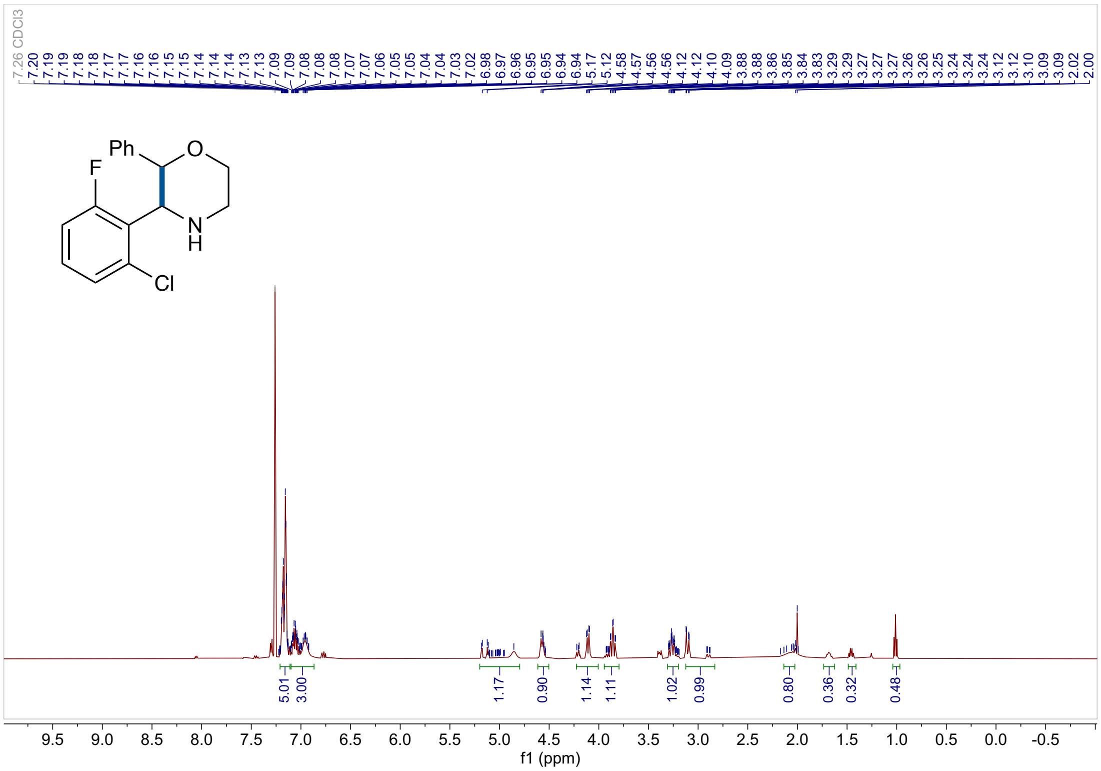

35 – ^{1}H NMR (500 MHz, $CDCl_3$)

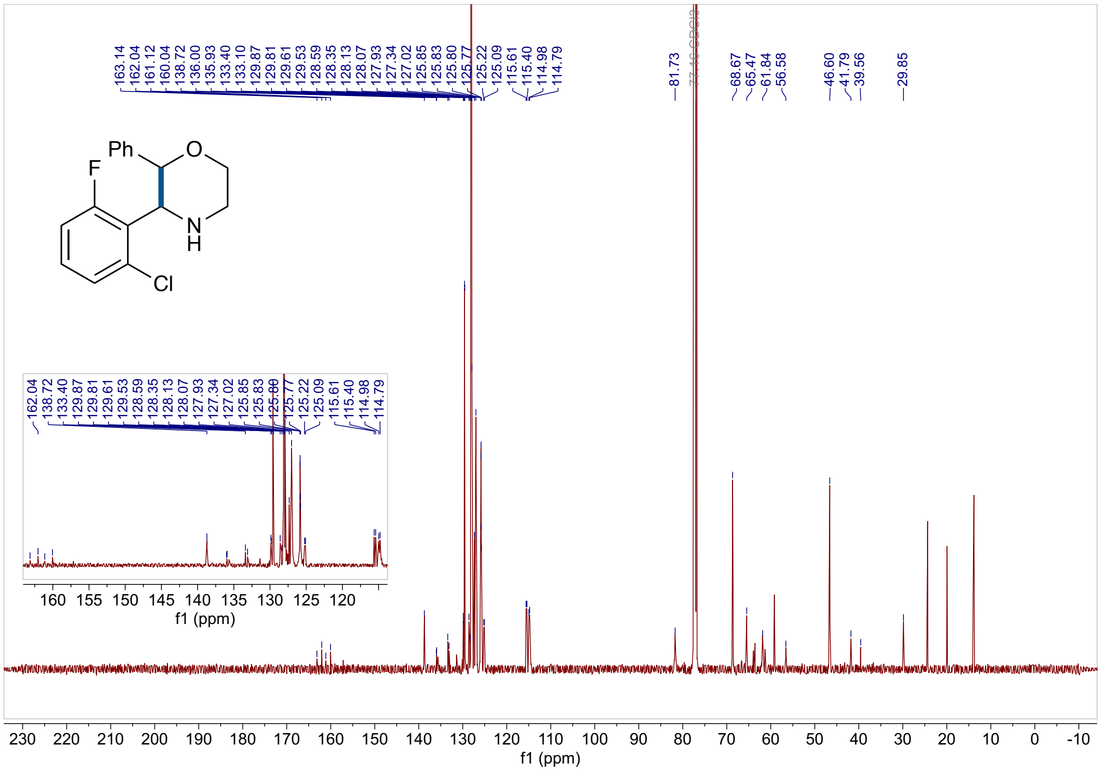

35 – ^{13}C NMR (126 MHz, $CDCl_3$)

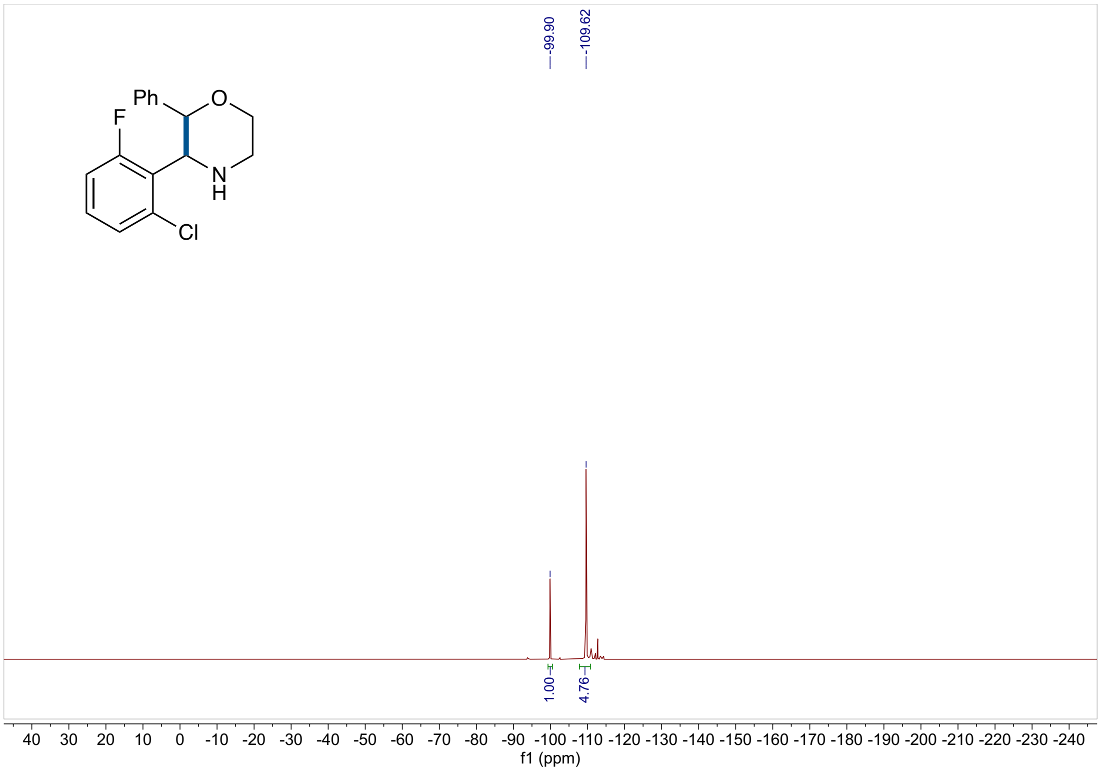

35 – ^{19}F NMR (471 MHz, $CDCl_3$)

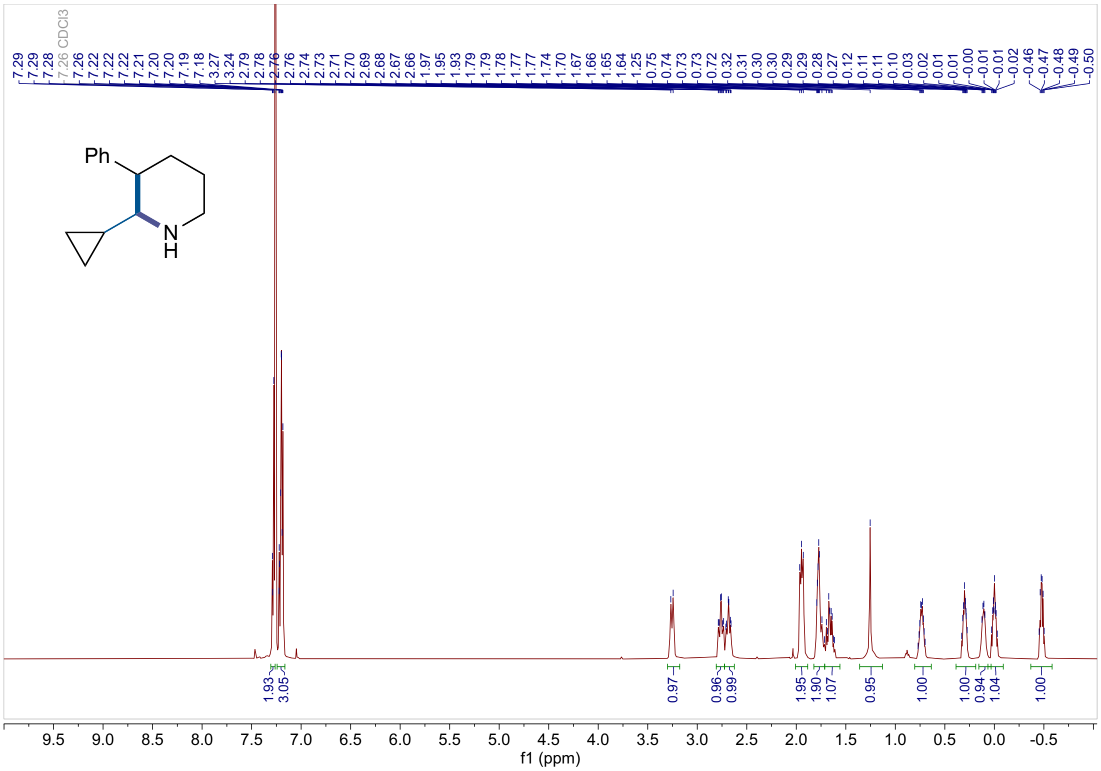

36 – ^{1}H NMR (500 MHz, $CDCl_3$)

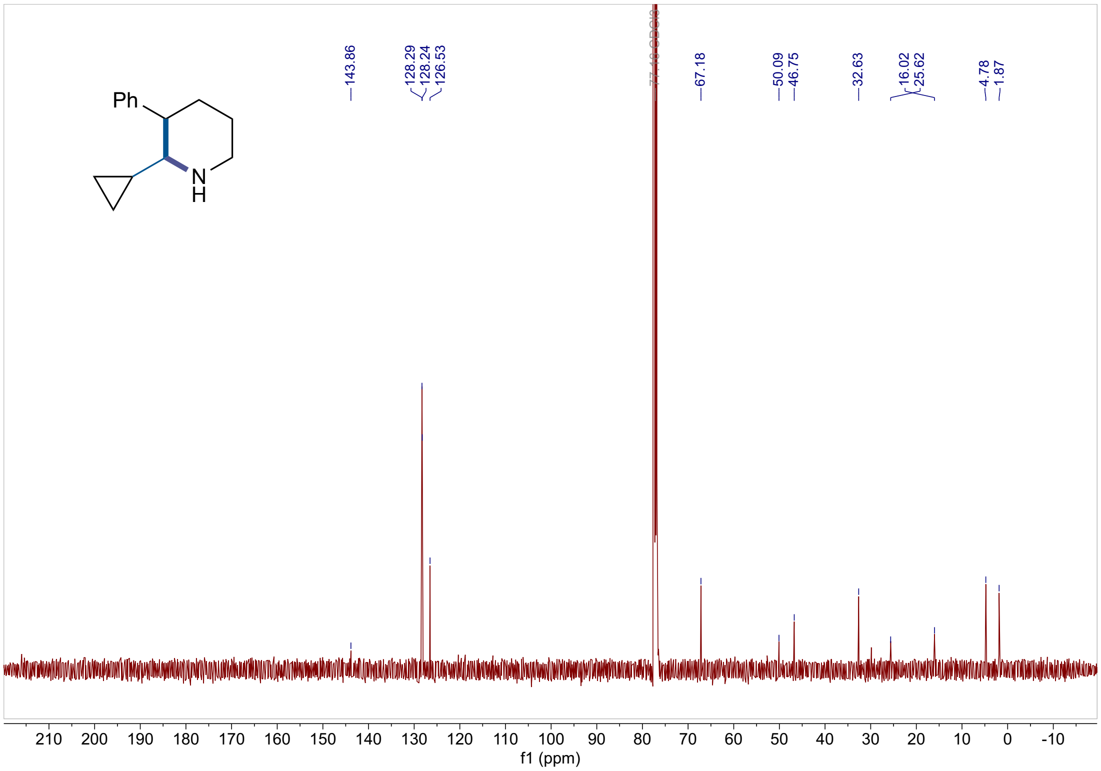

36 – ^{13}C NMR (126 MHz, $CDCl_3$)

Compound **36** – COSY analysis for major diastereomer determination

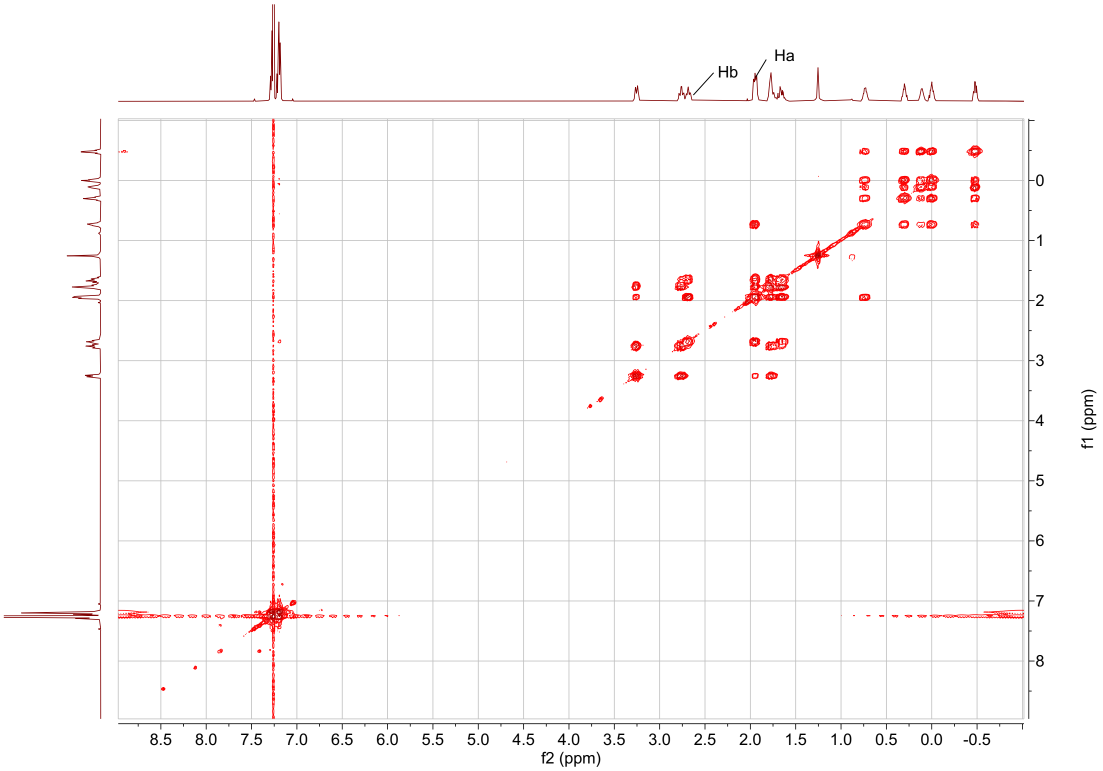

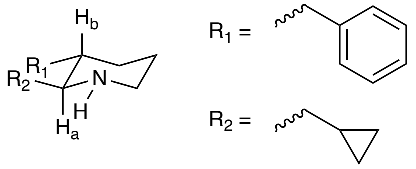

¹H NMR (500 MHz, CDCl3) and COSY

δ 1.95 H_a (m, overlapped with the other H)

δ 2.68 H_b (td, 11.5, 3.3 Hz)

based on *J*-coupling of H_a and H_b, major diastereomer is *trans*

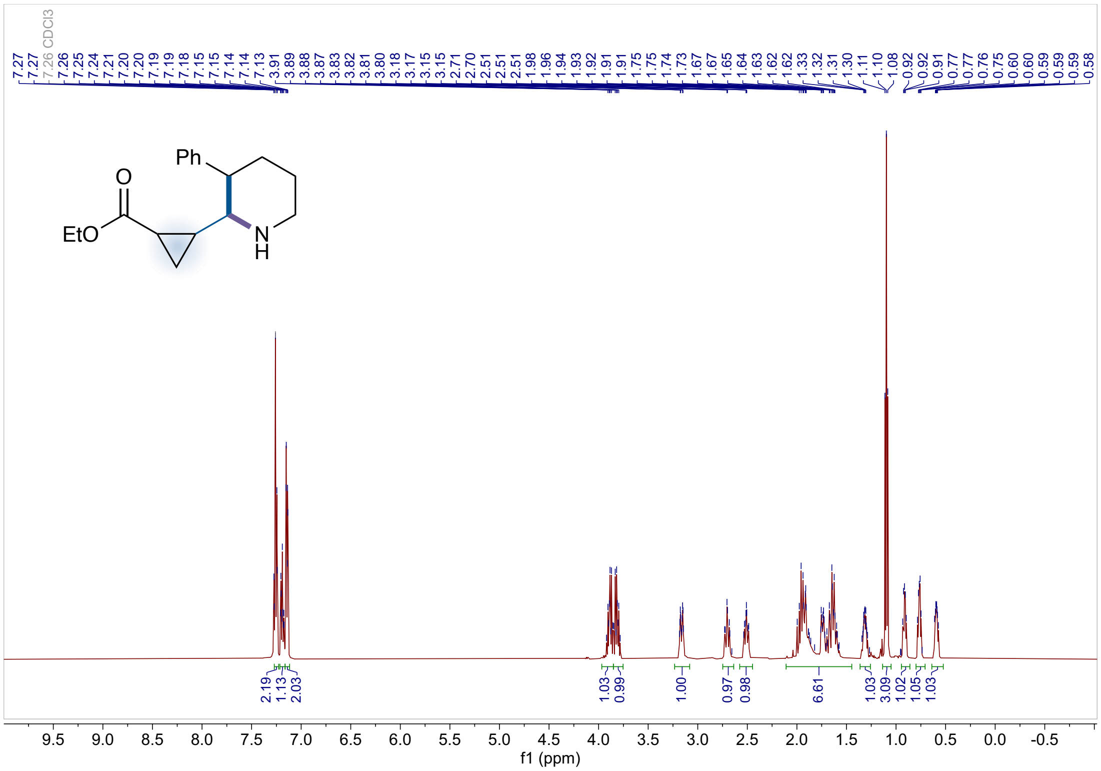

37 – ^{1}H NMR (500 MHz, $CDCl_3$) [major diastereomer]

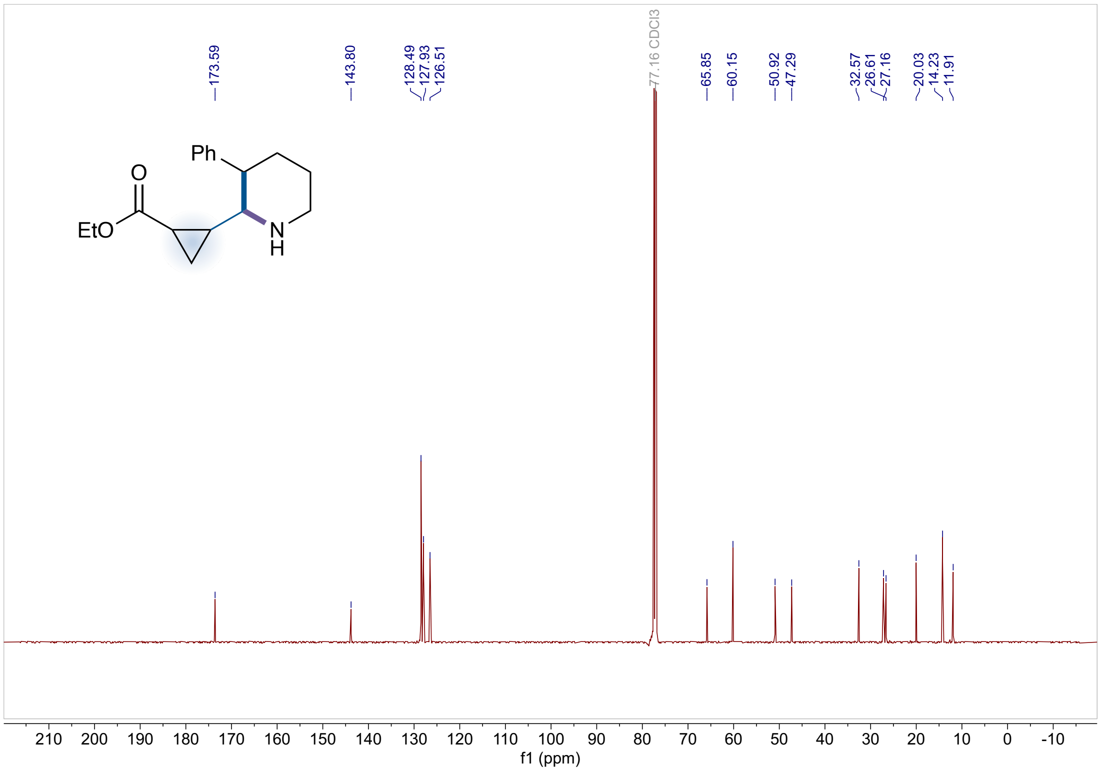

37 – ^{13}C NMR (126 MHz, $CDCl_3$) [major diastereomer]

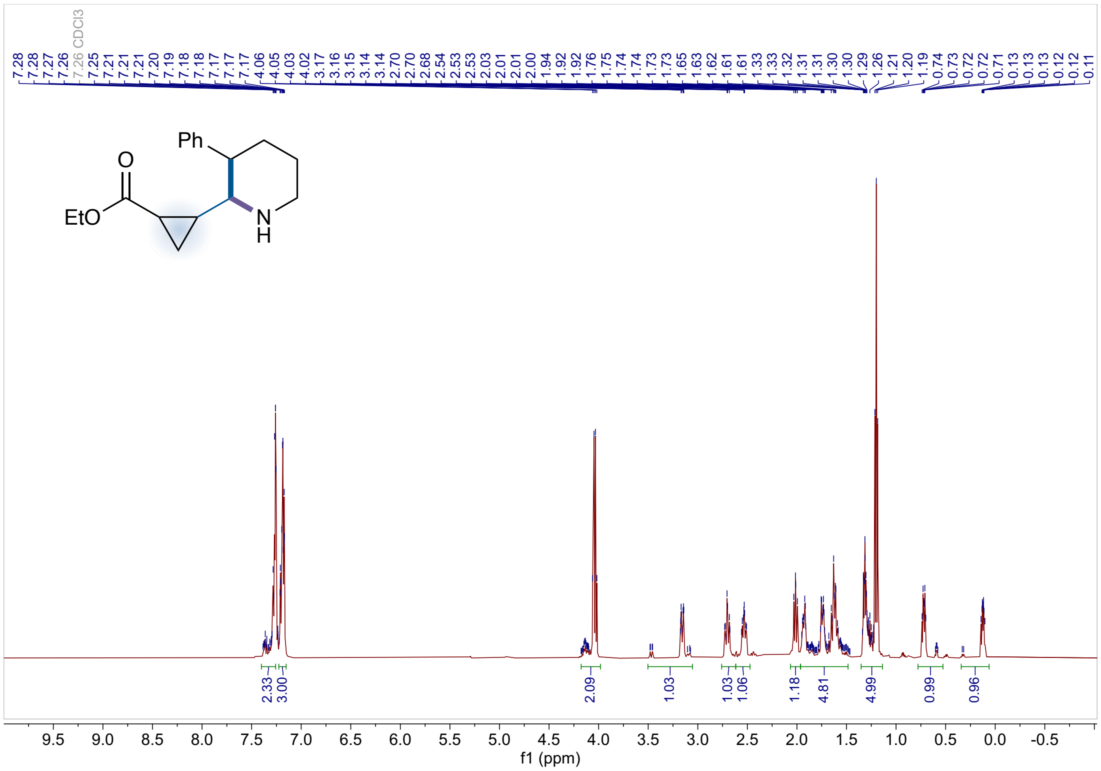

37 – ^{1}H NMR (500 MHz, $CDCl_3$) [mixture of minor diastereomers]

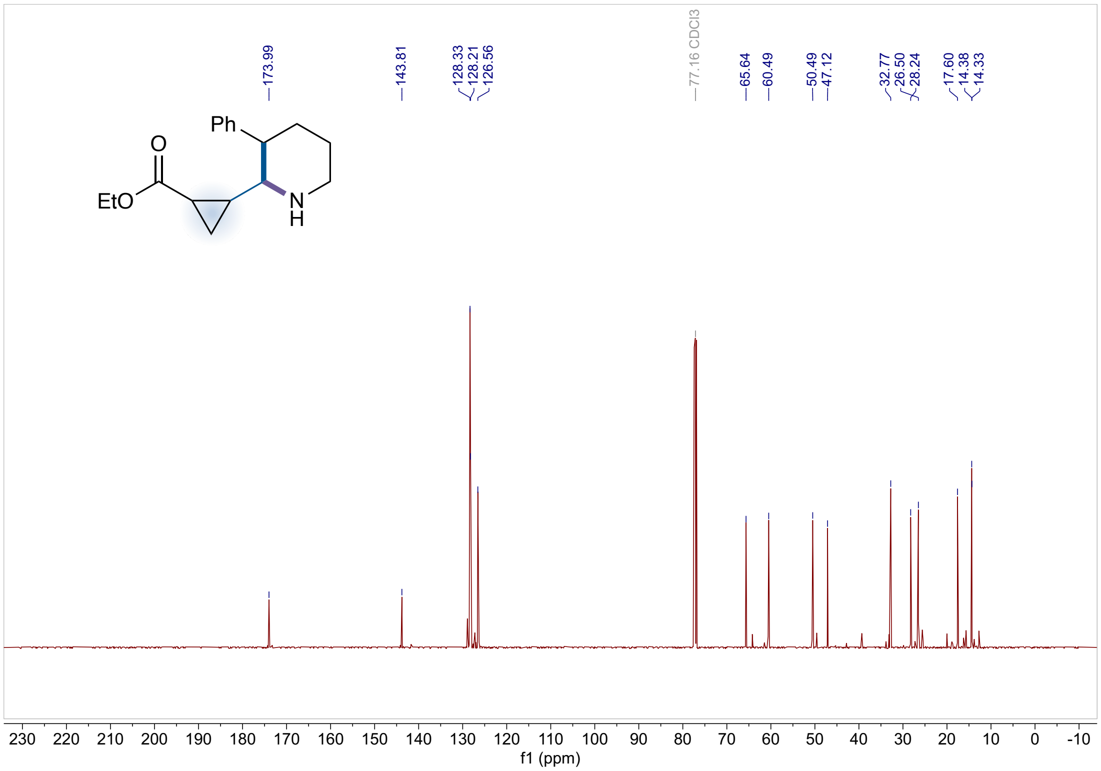

37 – ^{13}C NMR (126 MHz, $CDCl_3$) [mixture of minor diastereomers]

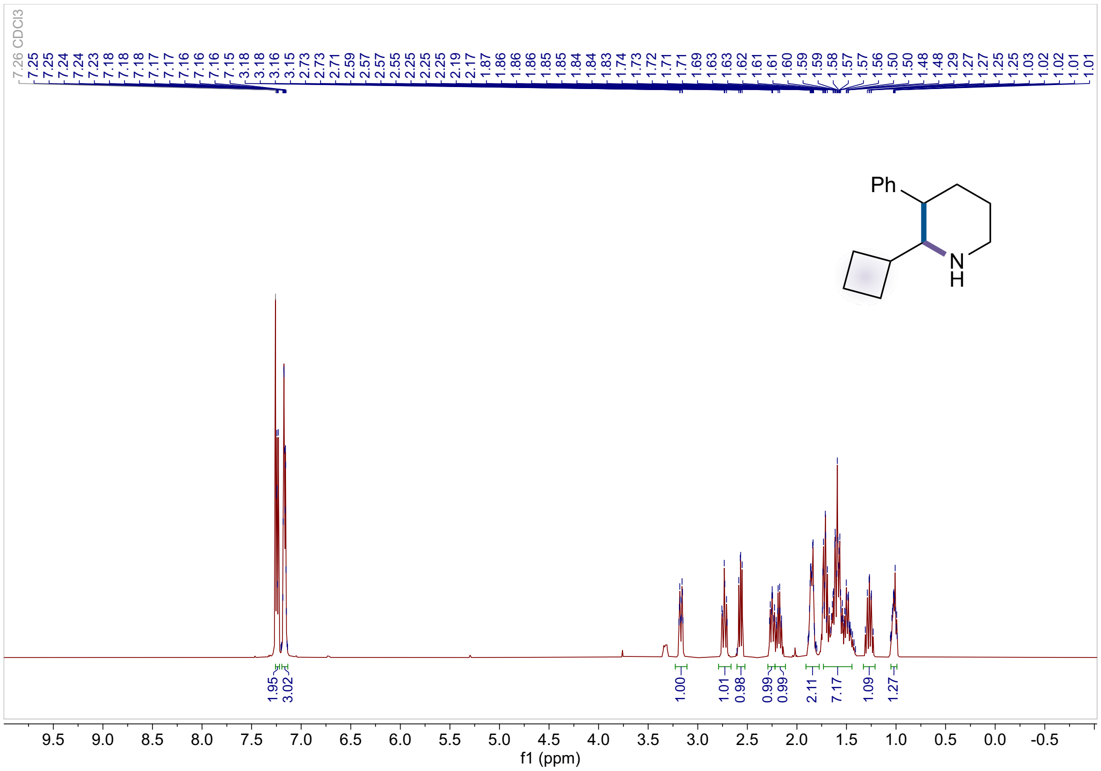

38 – ^{1}H NMR (500 MHz, $CDCl_3$)

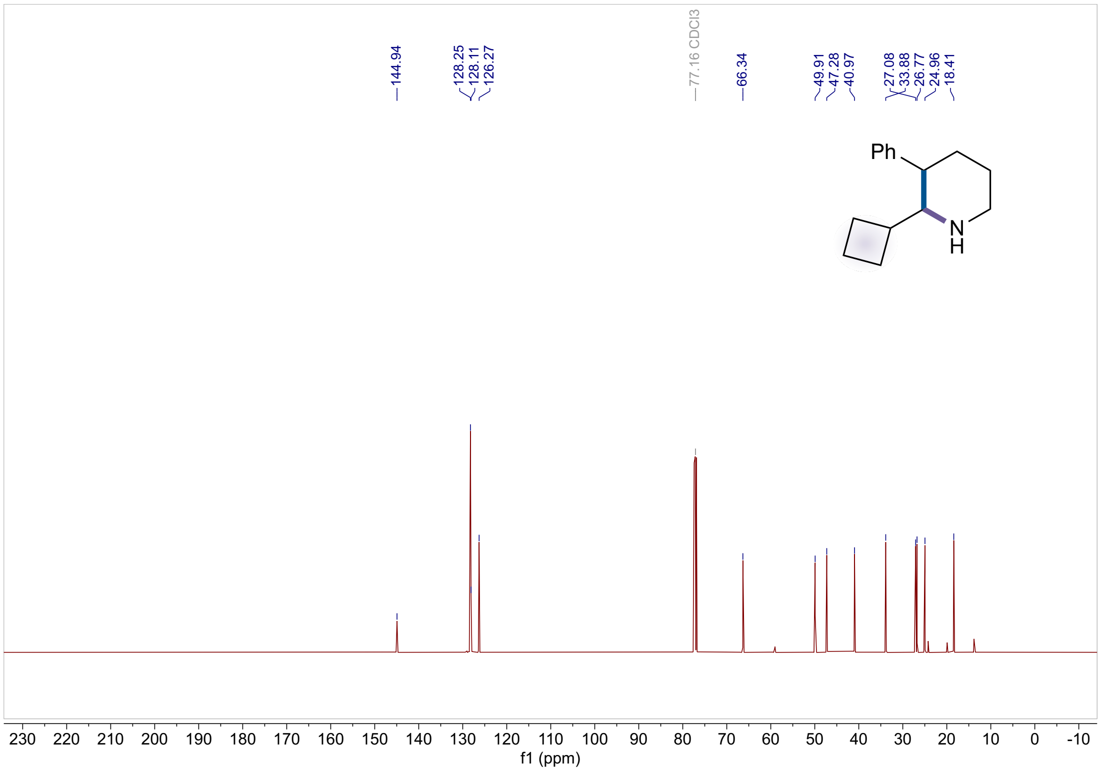

38 – ^{13}C NMR (126 MHz, $CDCl_3$) [trace minor diastereomer was observed]

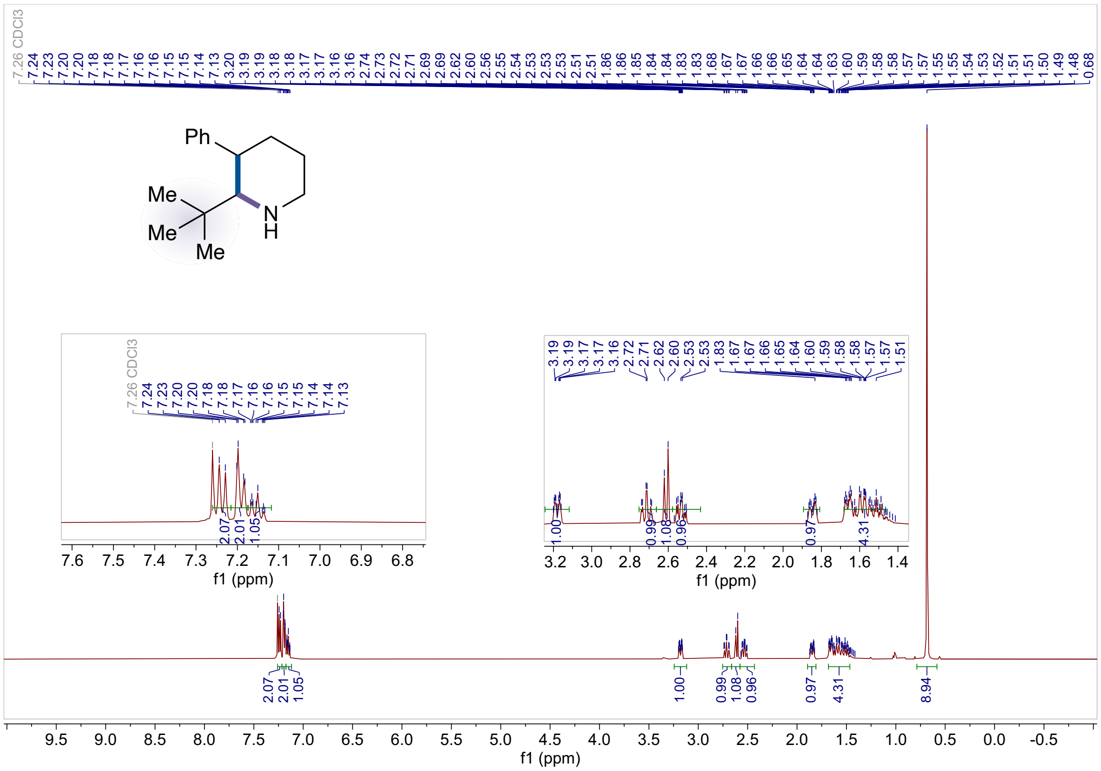

39 – ^{1}H NMR (500 MHz, $CDCl_3$)

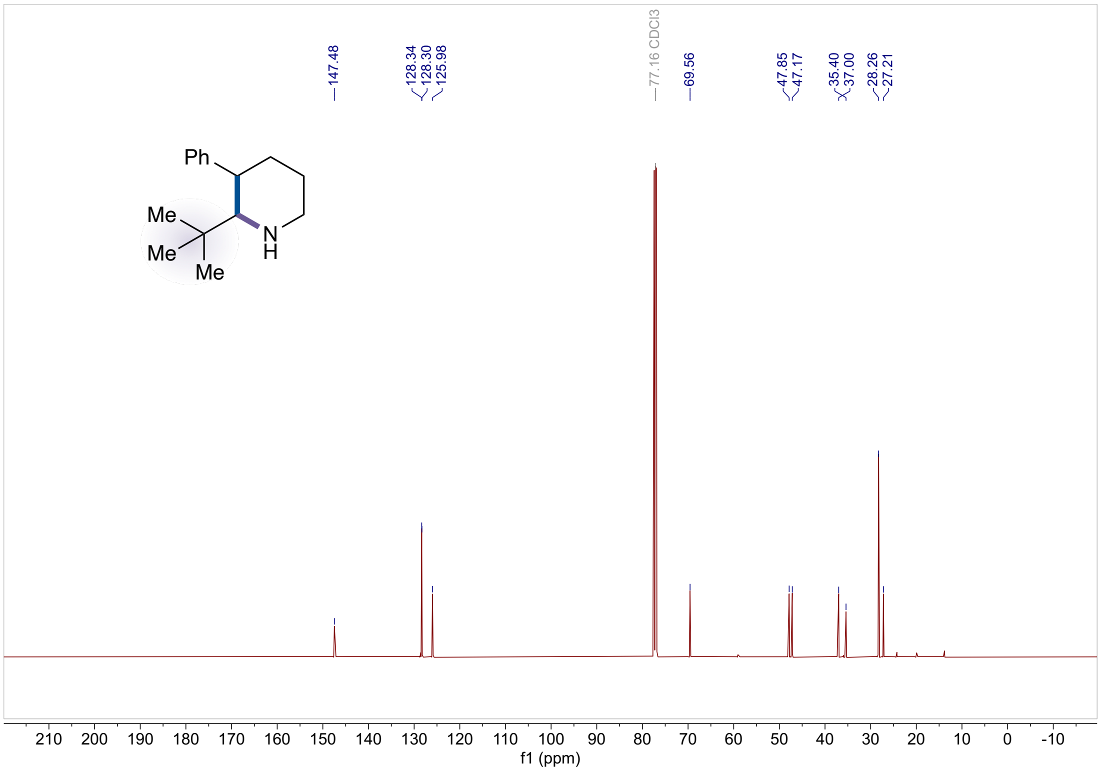

39 – ^{13}C NMR (126 MHz, $CDCl_3$)

Compound **39** – COSY analysis for major diastereomer determination

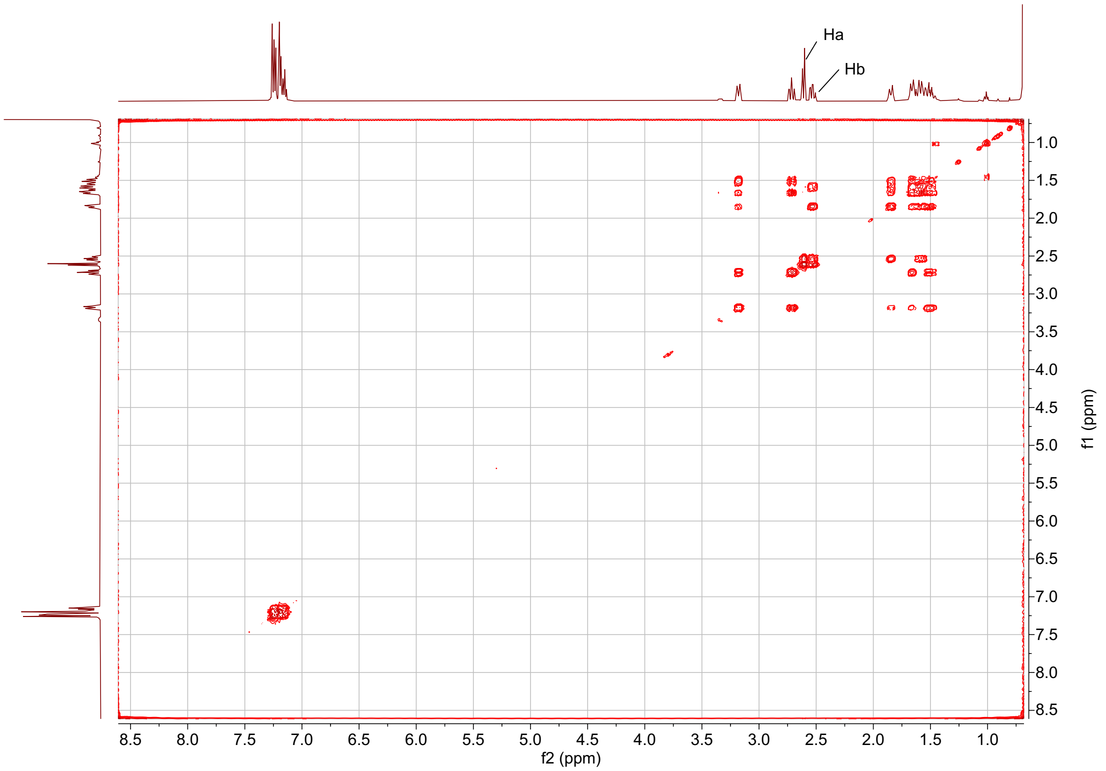

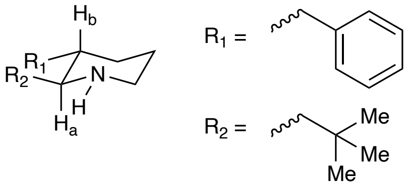

^{1}H NMR (500 MHz, CDCl3) and COSY

δ 2.61 H_a (d, 9.9 Hz)

δ 2.53 H_b (ddd, 11.5, 9.8, 3.7 Hz)

based on *J*-coupling of H_a and H_b, major diastereomer is *trans*

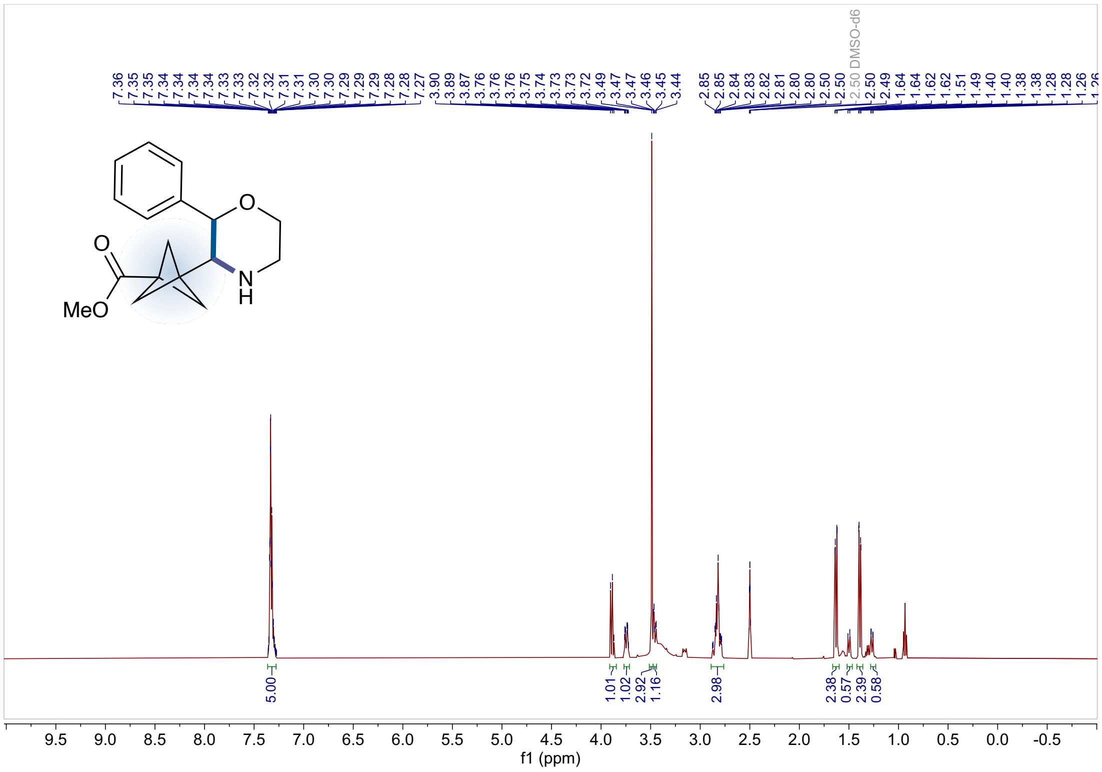

40 – ^{1}H NMR (500 MHz, DMSO)

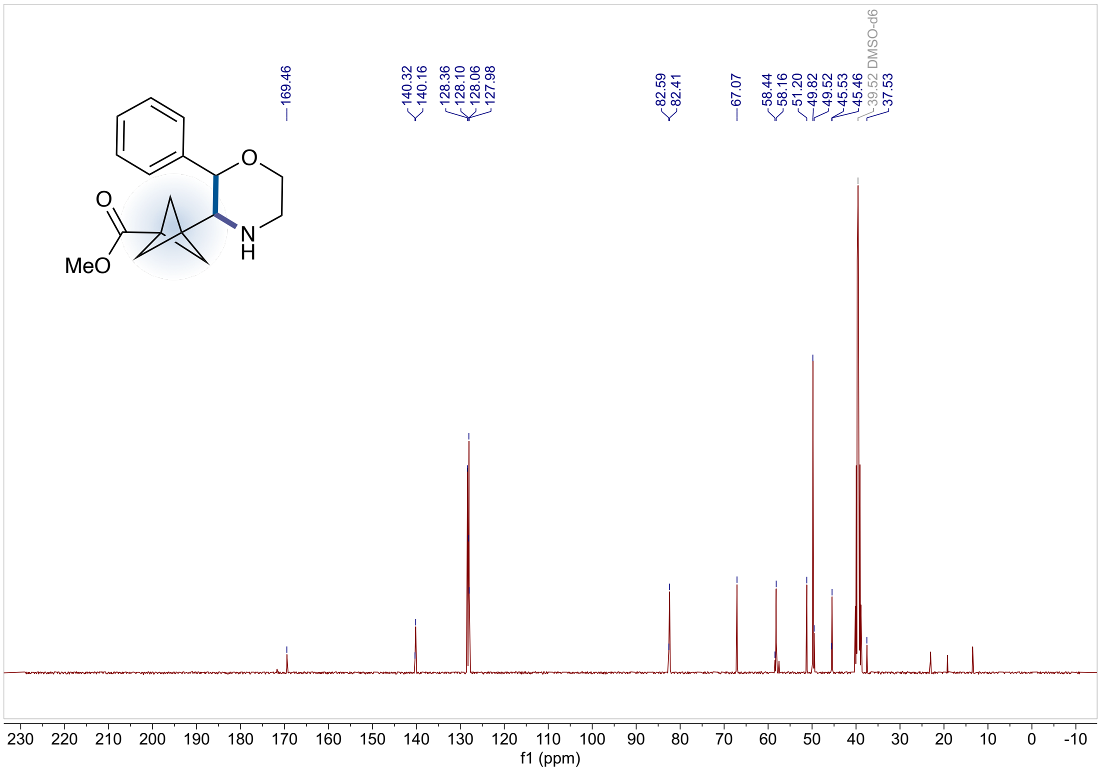

40 – ^{13}C NMR (101 MHz, DMSO)

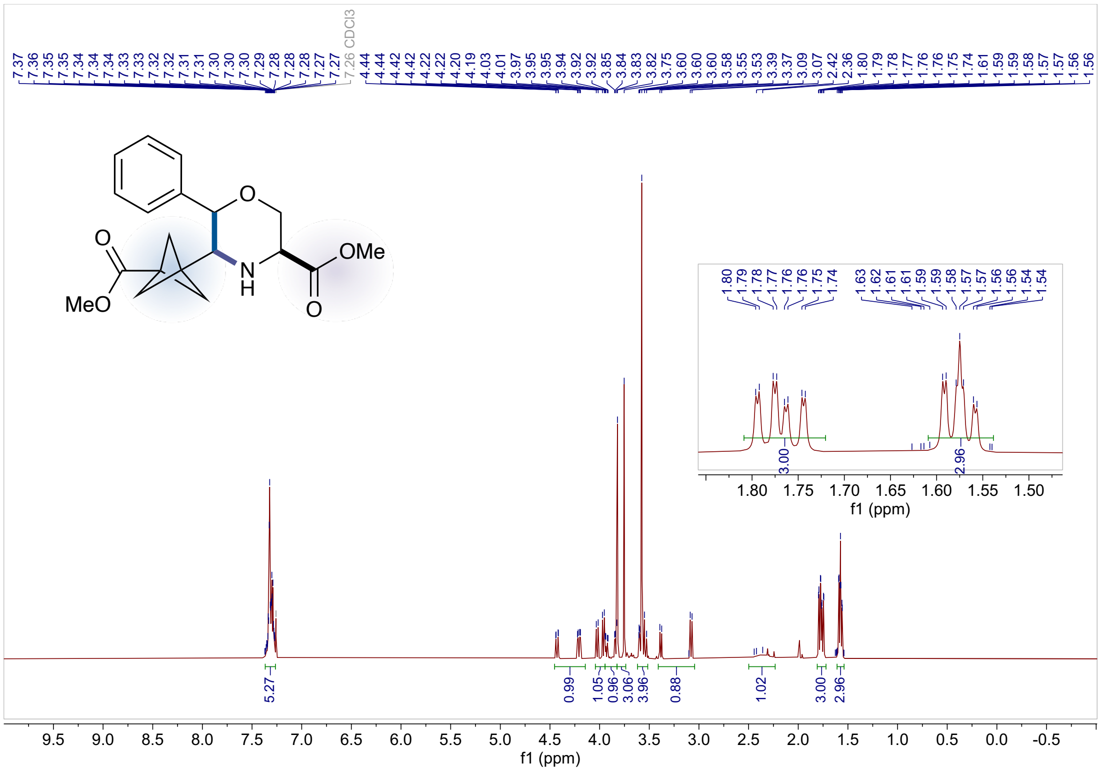

41 – ^{1}H NMR (500 MHz, CDCl$_3$)

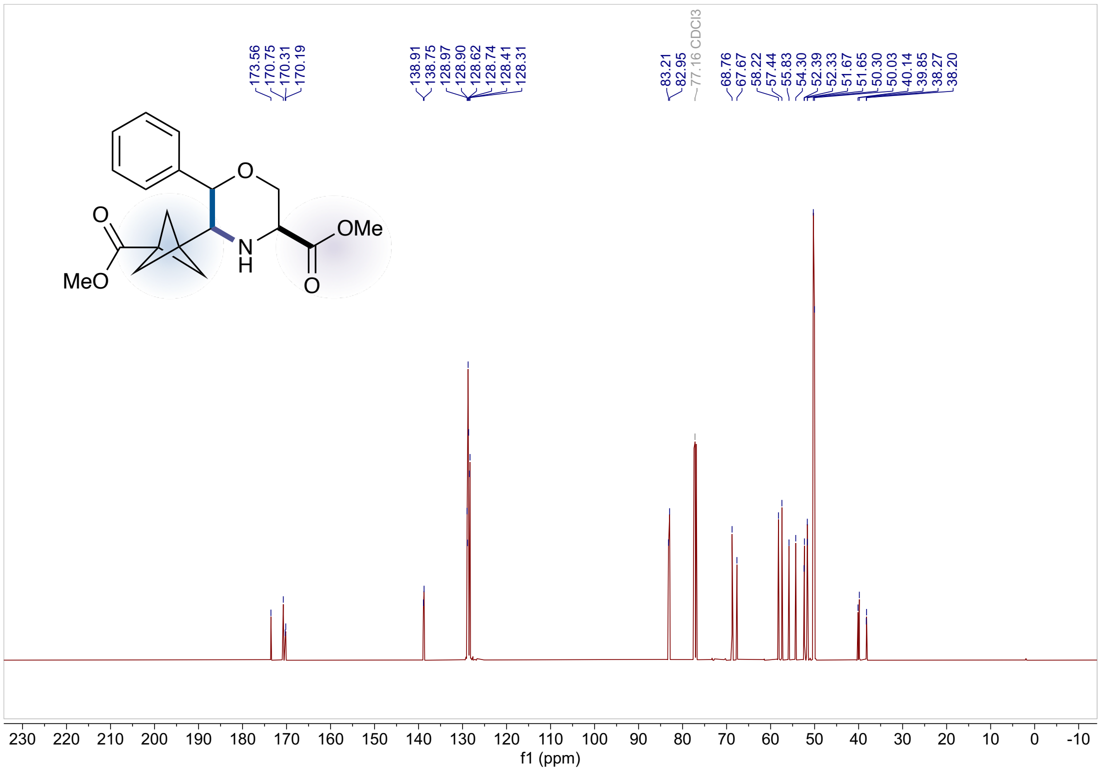

41 – ^{13}C NMR (126 MHz, CDCl$_3$)

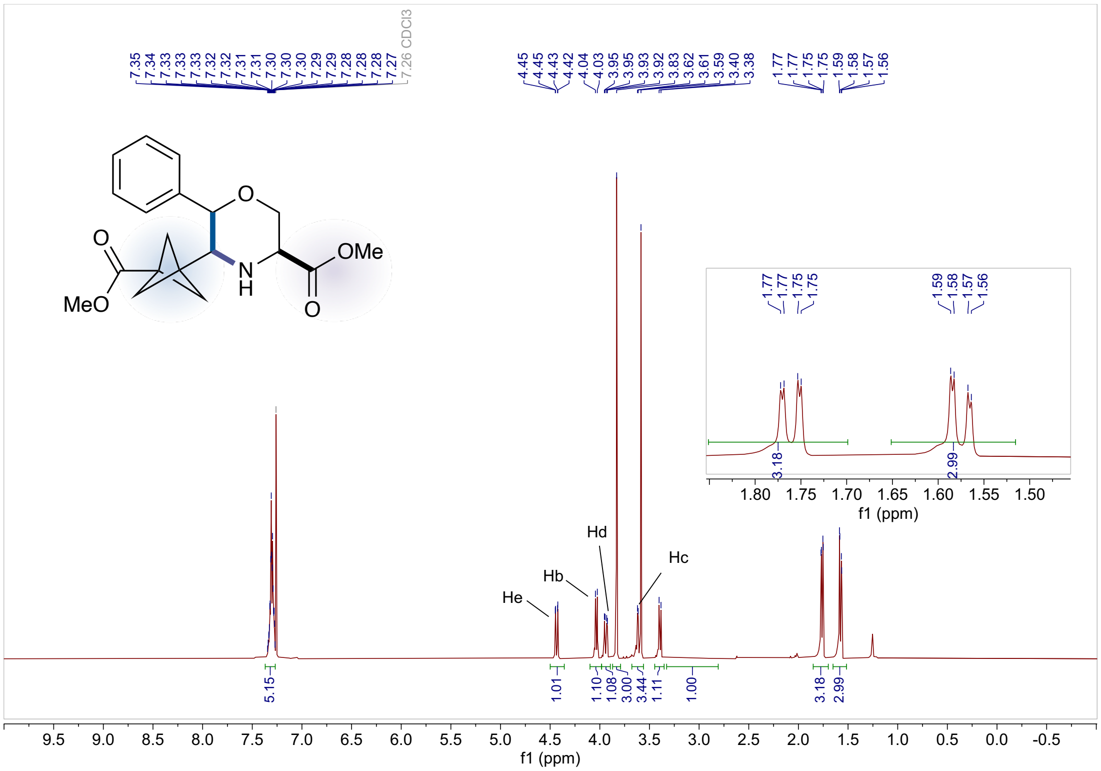

41 – ^{1}H NMR (500 MHz, $CDCl_3$) [major diastereomer]

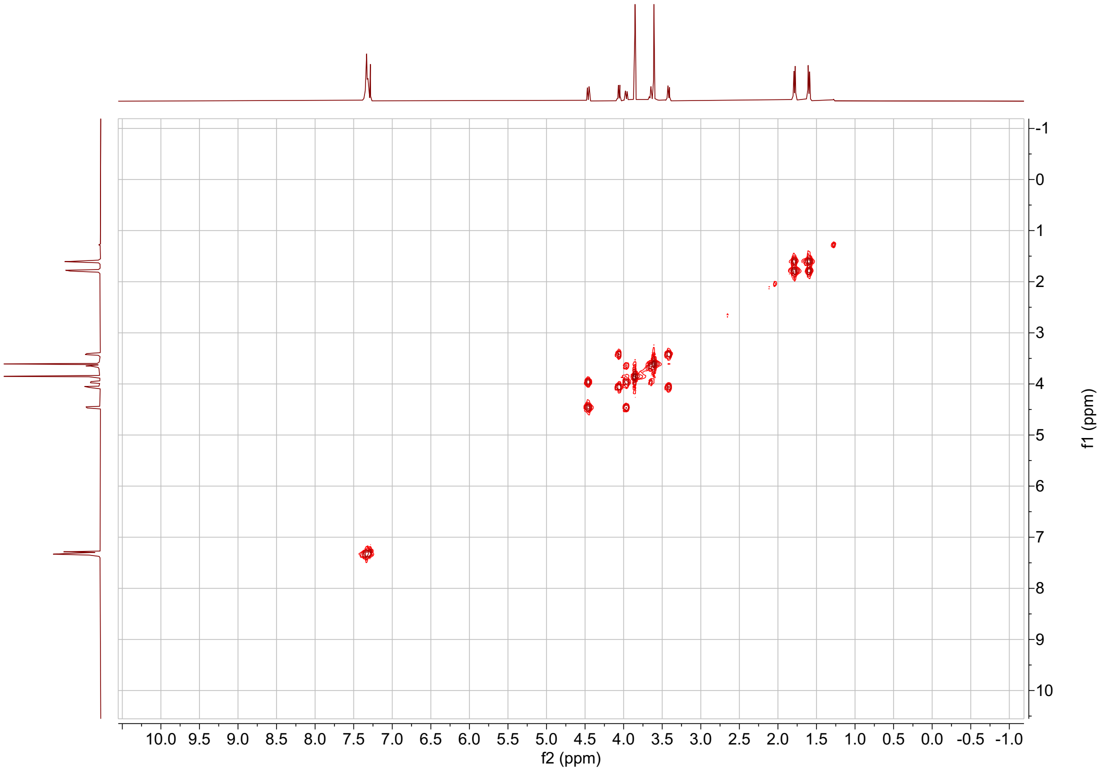

41 – COSY [major diastereomer]

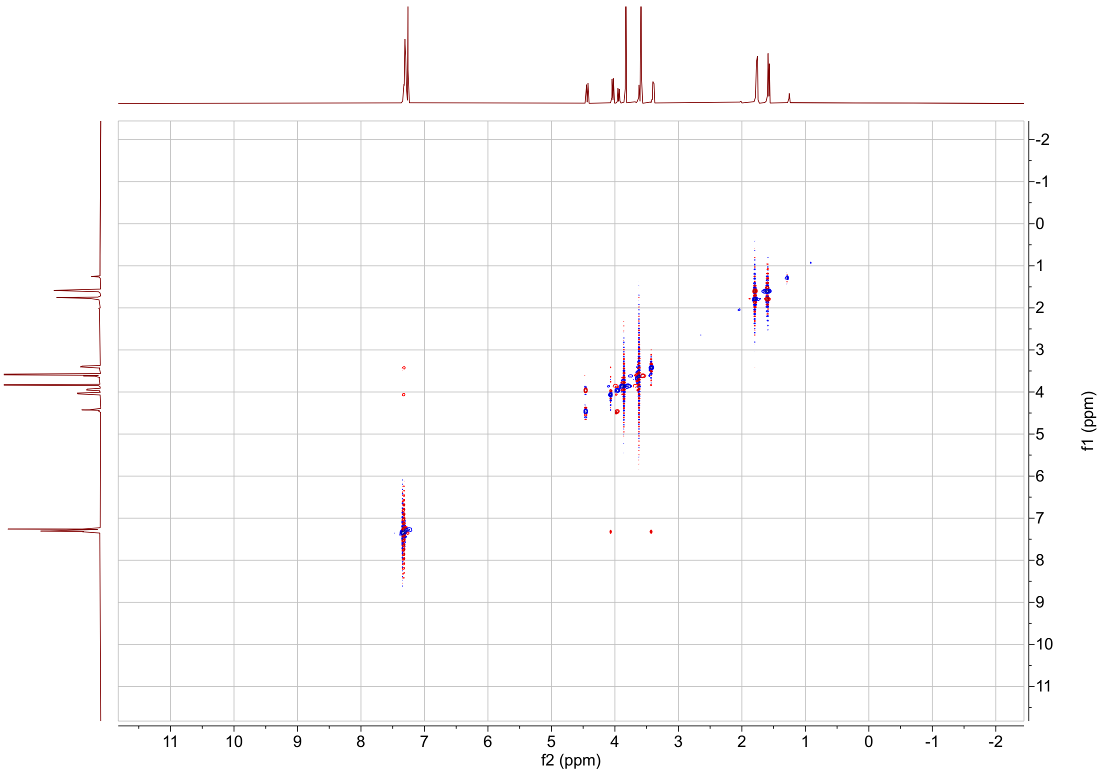

41 – NOSEY [major diastereomer]

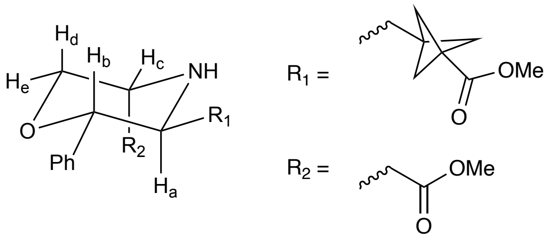

^{1}**H NMR** (500 MHz, CDCl3), COSY and NOSEY

δ 4.44 H_e (dd, 11.4, 1.3 Hz)

δ 4.03 H_b (d, 9.5 Hz)

δ 3.94 H_d (dd, 11.5, 3.5 Hz)

δ 3.63 H_c (d, 3.5 Hz, 1.3 Hz not observed)

δ 3.39 H_a (d, 9.5 Hz)

based on *J*-coupling of H_a and H_b, the relation between Ph and BCP is *trans*

based on *J*-coupling of H_d and H_e, 11.4 Hz corresponds to geminal coupling

based on *J*-coupling of H_d and H_c, 3.5 Hz corresponds to equatorial-axial coupling

based on *J*-coupling of H_e and H_c, 1.3 Hz corresponds to equatorial-equatorial coupling

NOSEY didn't demonstrate strong spatial between H_a and H_c, implying greater distance between the two protons.

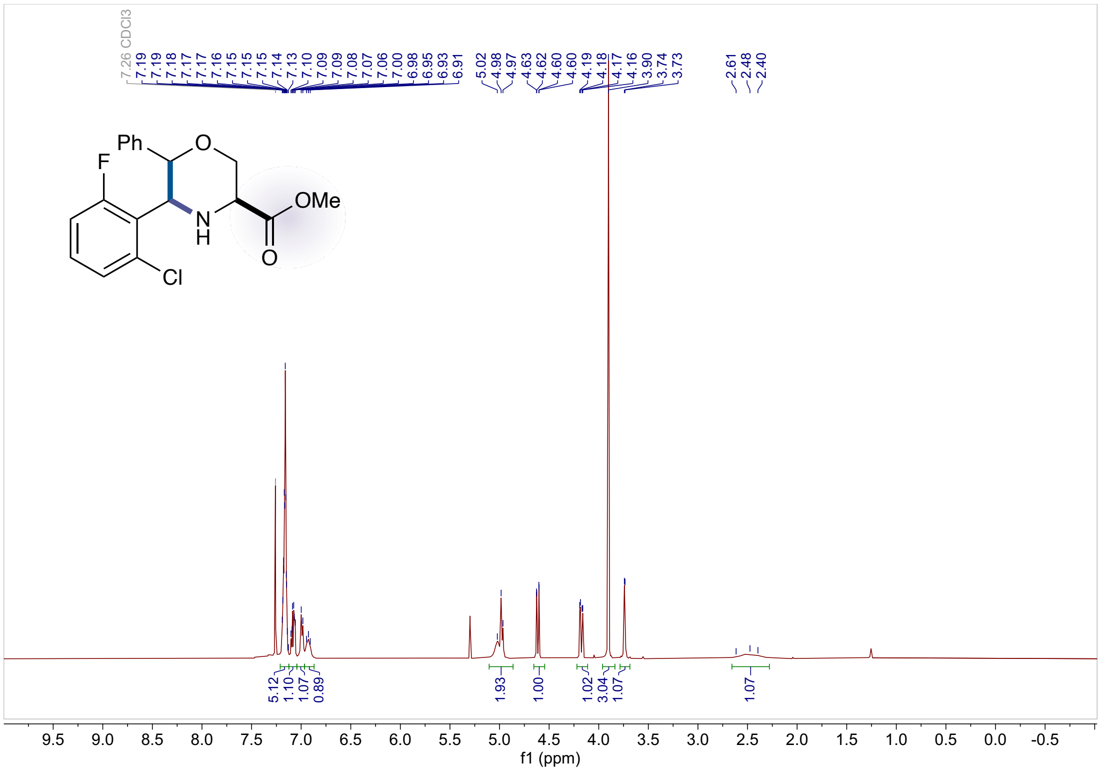

42 – ^{1}H NMR (500 MHz, $CDCl_3$) [major diastereomer]

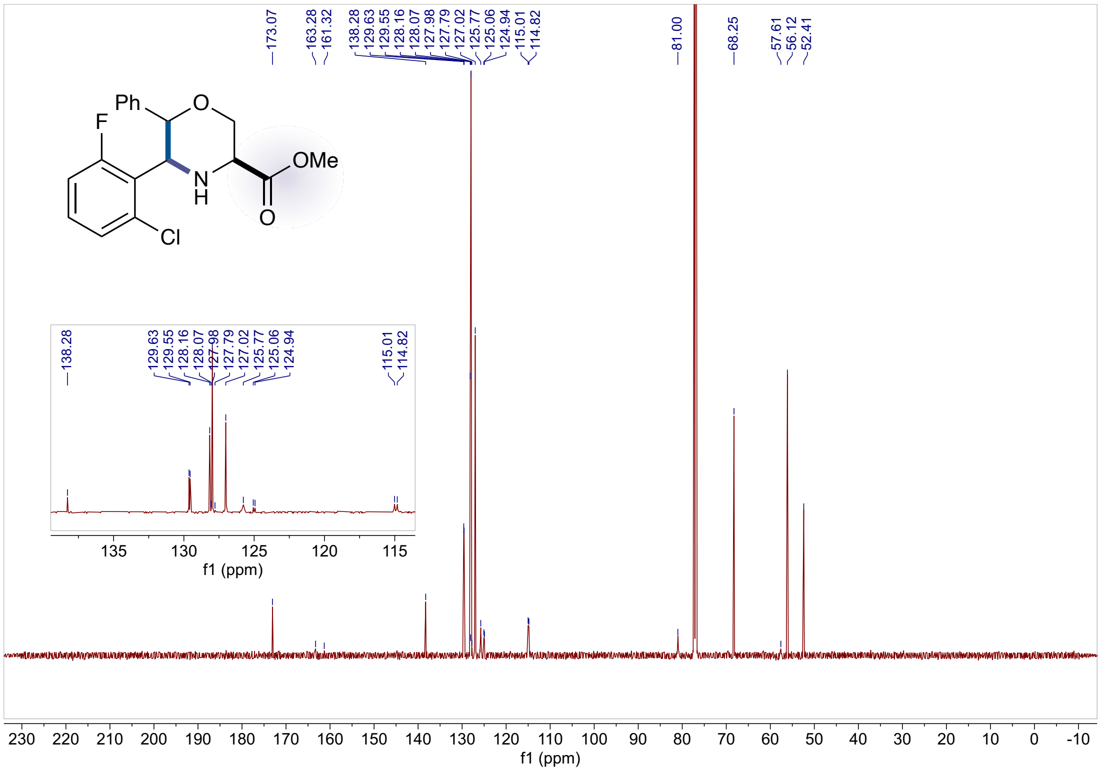

42 – ^{13}C NMR (101 MHz, $CDCl_3$) [major diastereomer]

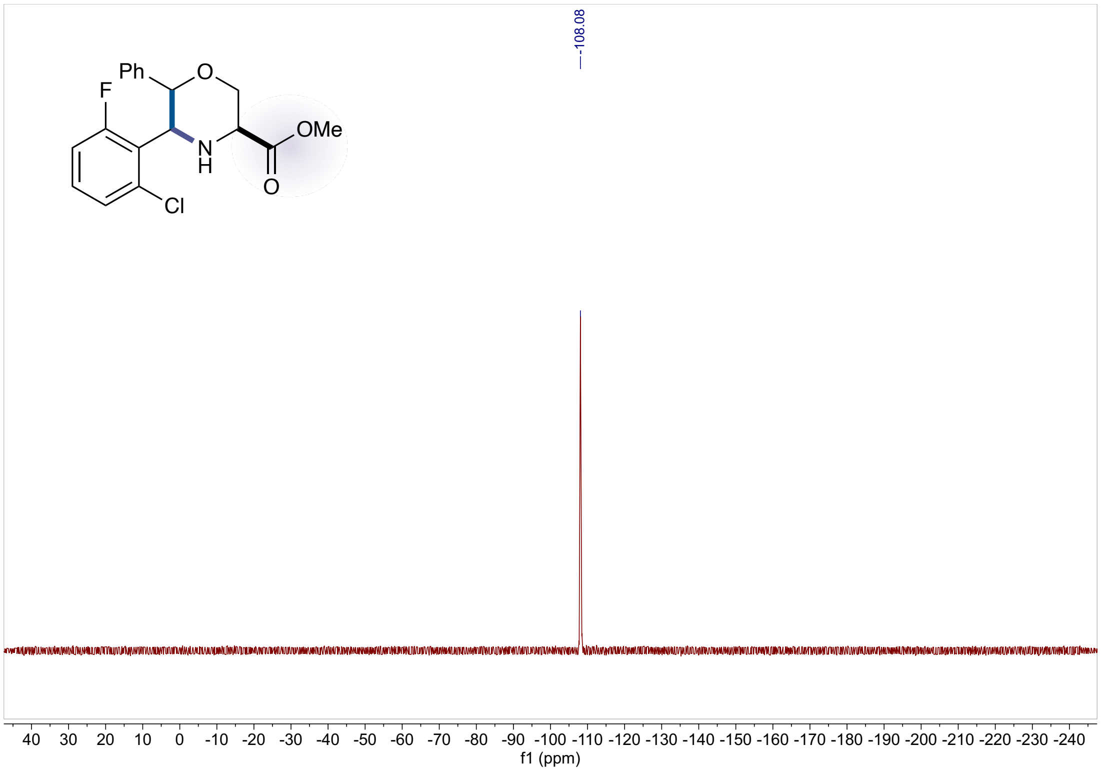

42 – ^{19}F NMR (471 MHz, $CDCl_3$) [major diastereomer]

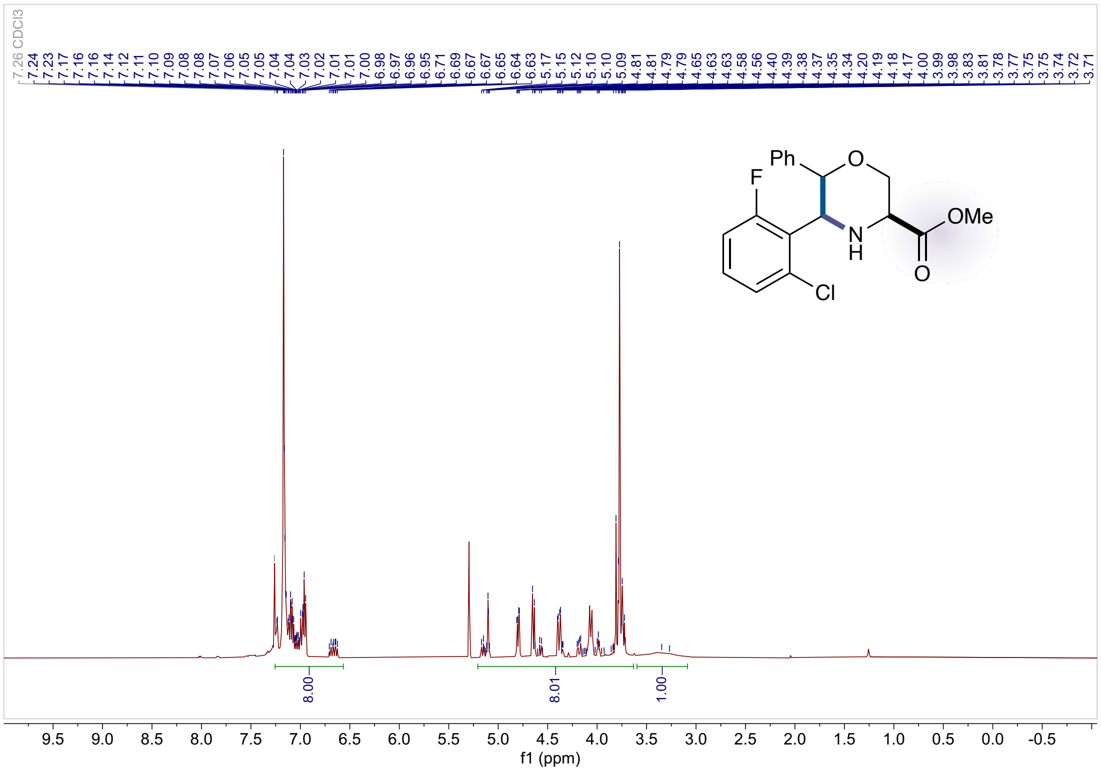

42 – ^{1}H NMR (500 MHz, $CDCl_3$) [mixture of minor diastereomers]

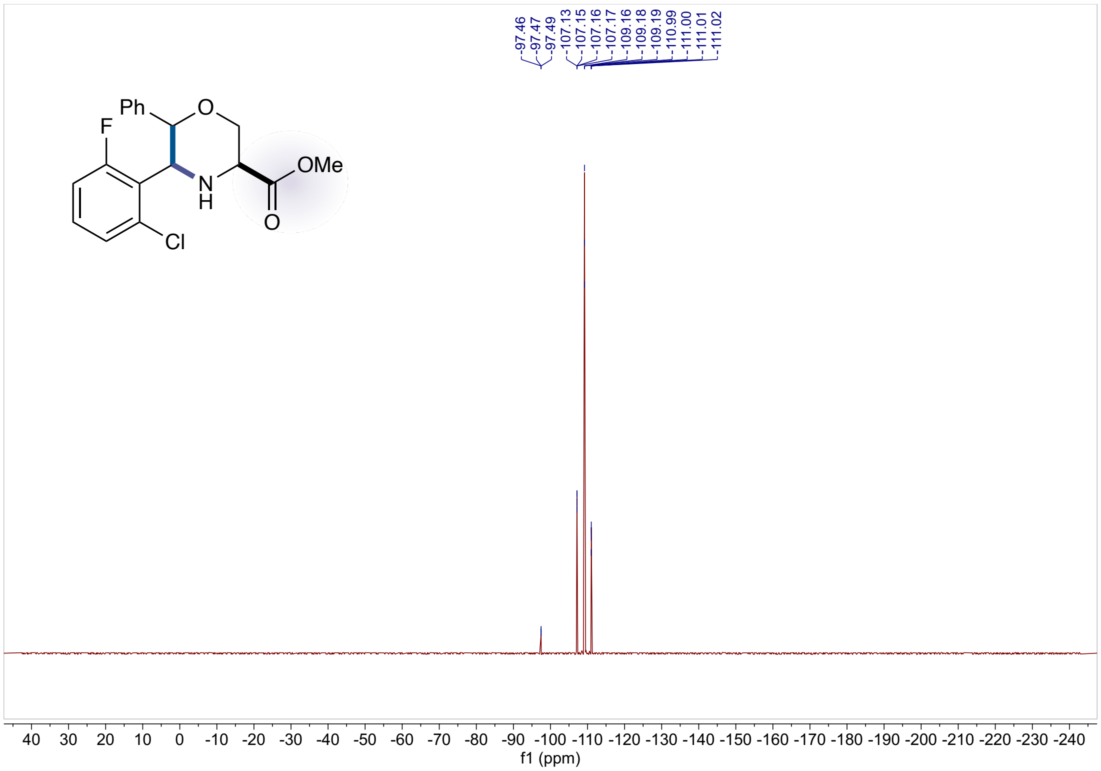

42 – ^{19}F NMR (471 MHz, $CDCl_3$) [mixture of minor diastereomers]

Compound **42** – uHPLC analysis for d.r. determination

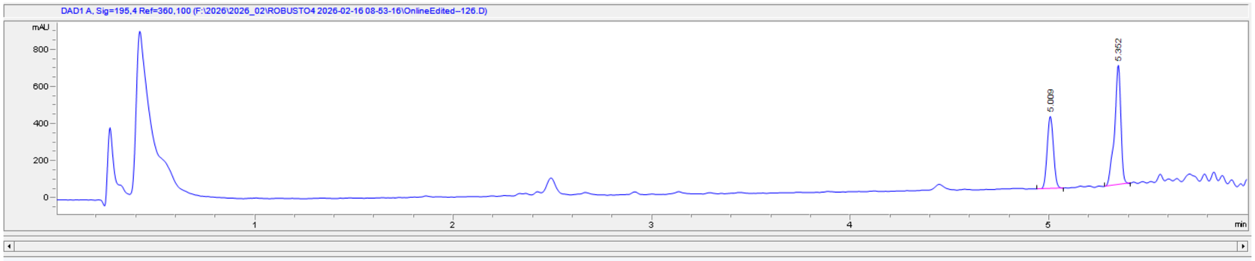

#	Time	Type	Area	Height	Width	Area%	Symmetry
1	5.009	BB	850.8	388.1	0.0346	37.344	0.935
2	5.352	BB	1427.5	645.8	0.0328	62.656	1.416

d.r. = 1.68 [combined minor dia]:1 [major dia] (based on correspondence between NMR data and uHPLC retention time, peak at 5.009 min is major diastereomer; peak at 5.352 min contains multiple minor diastereomers)

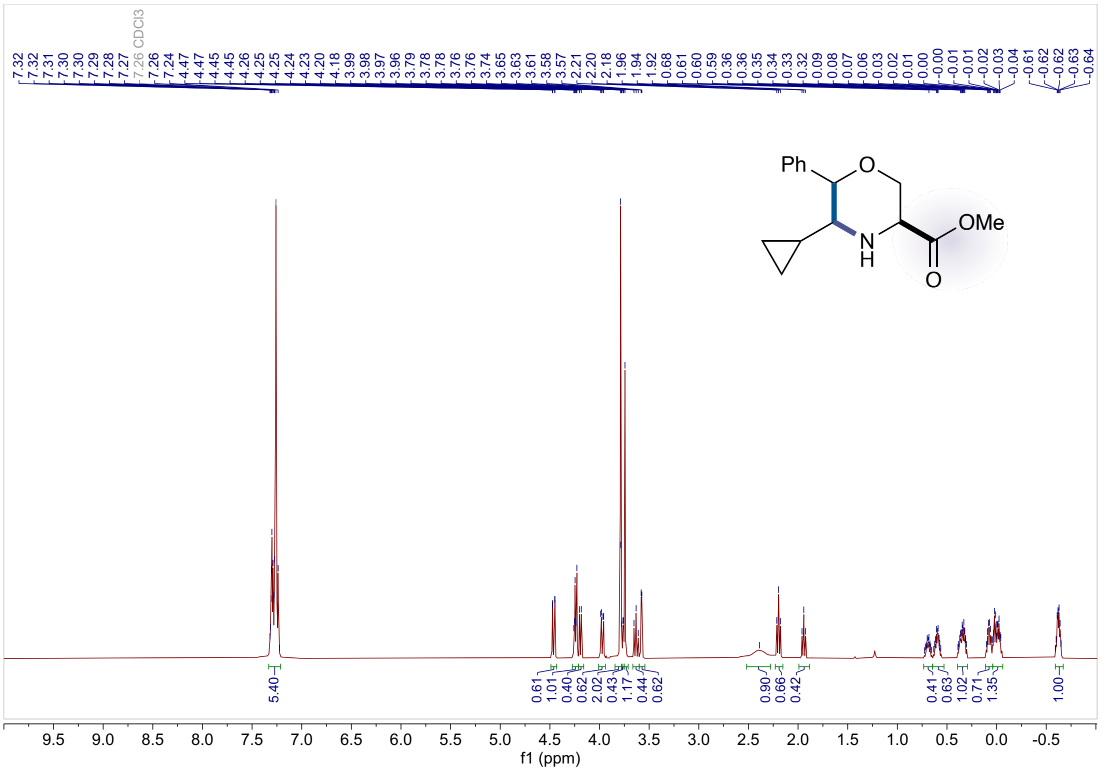

43 – ^{1}H NMR (500 MHz, $CDCl_3$)

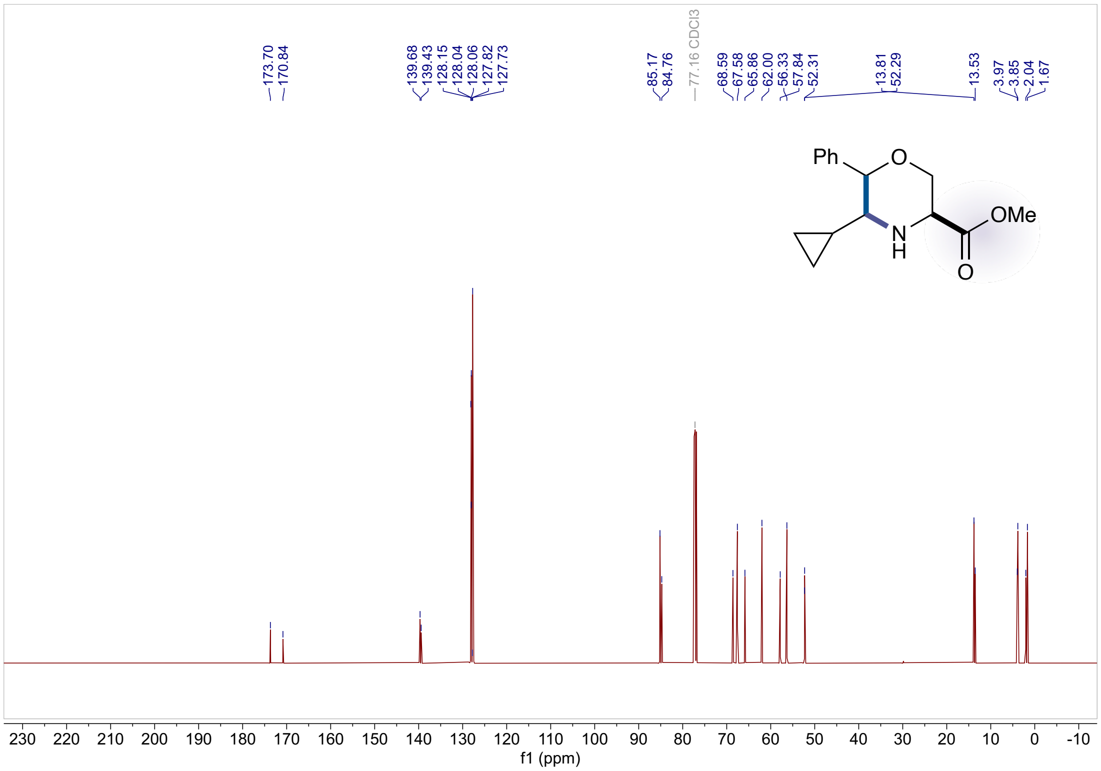

43 – ^{13}C NMR (126 MHz, $CDCl_3$)

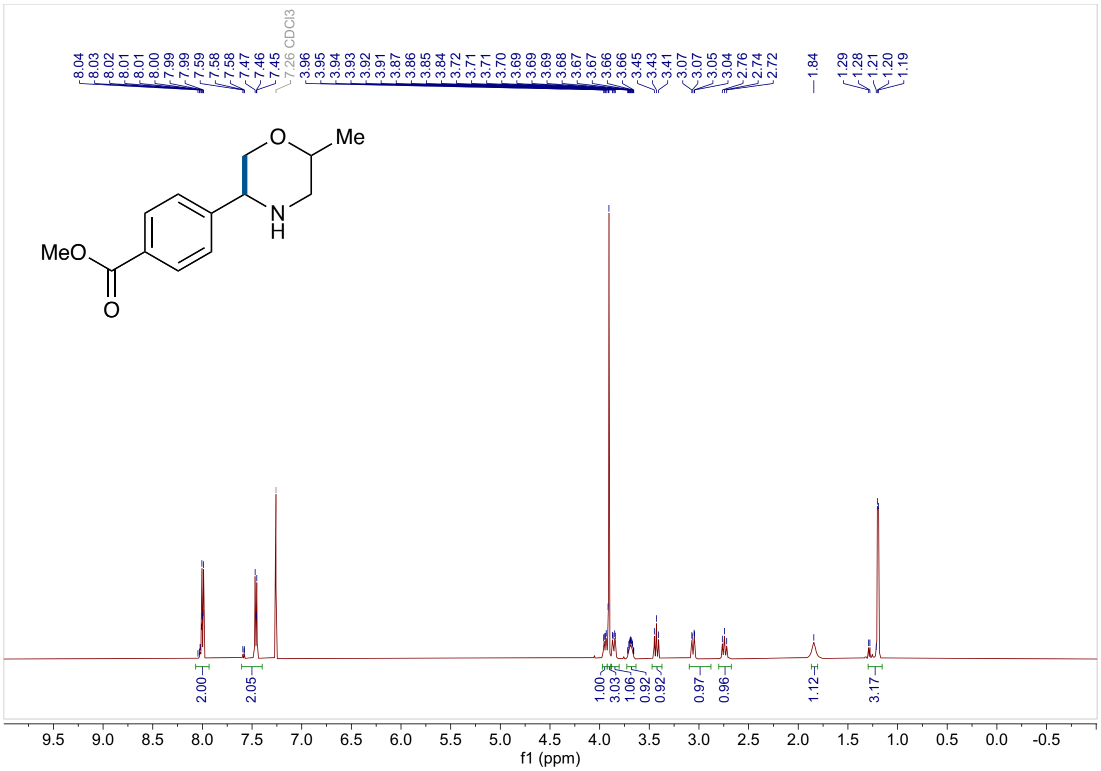

S-1 – ^{1}H NMR (500 MHz, $CDCl_3$)

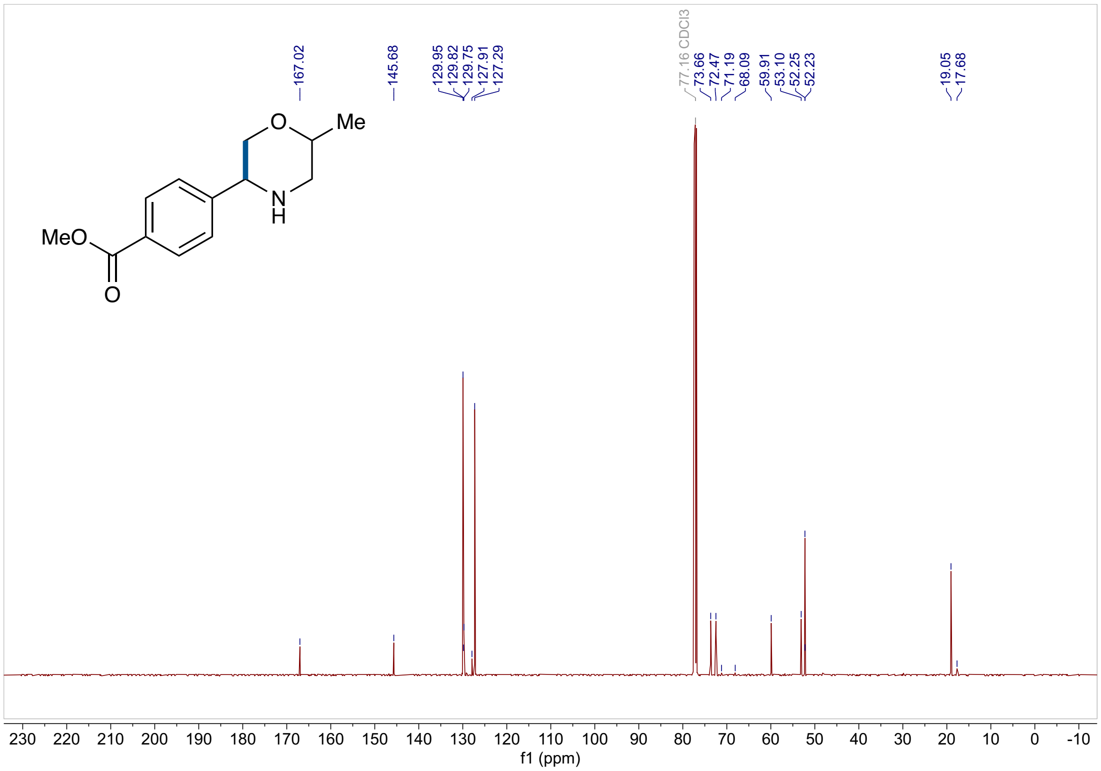

S-1 – ^{13}C NMR (126 MHz, $CDCl_3$)

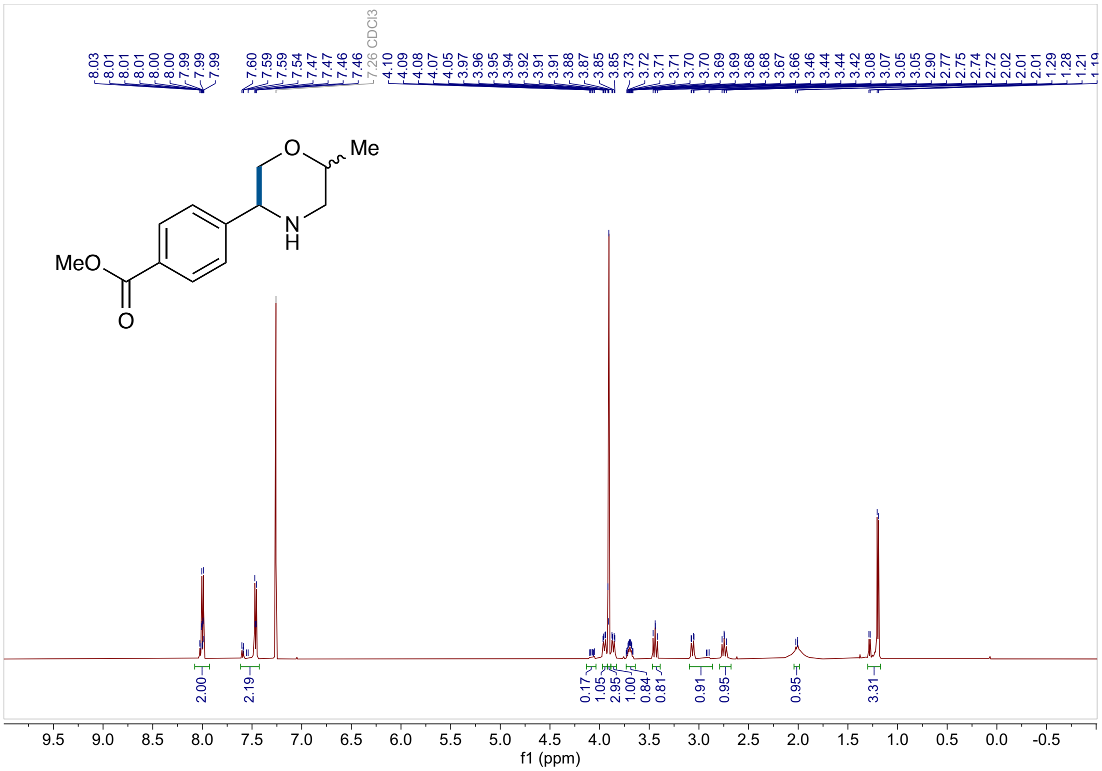

45 – ^{1}H NMR (500 MHz, CDCl$_3$)

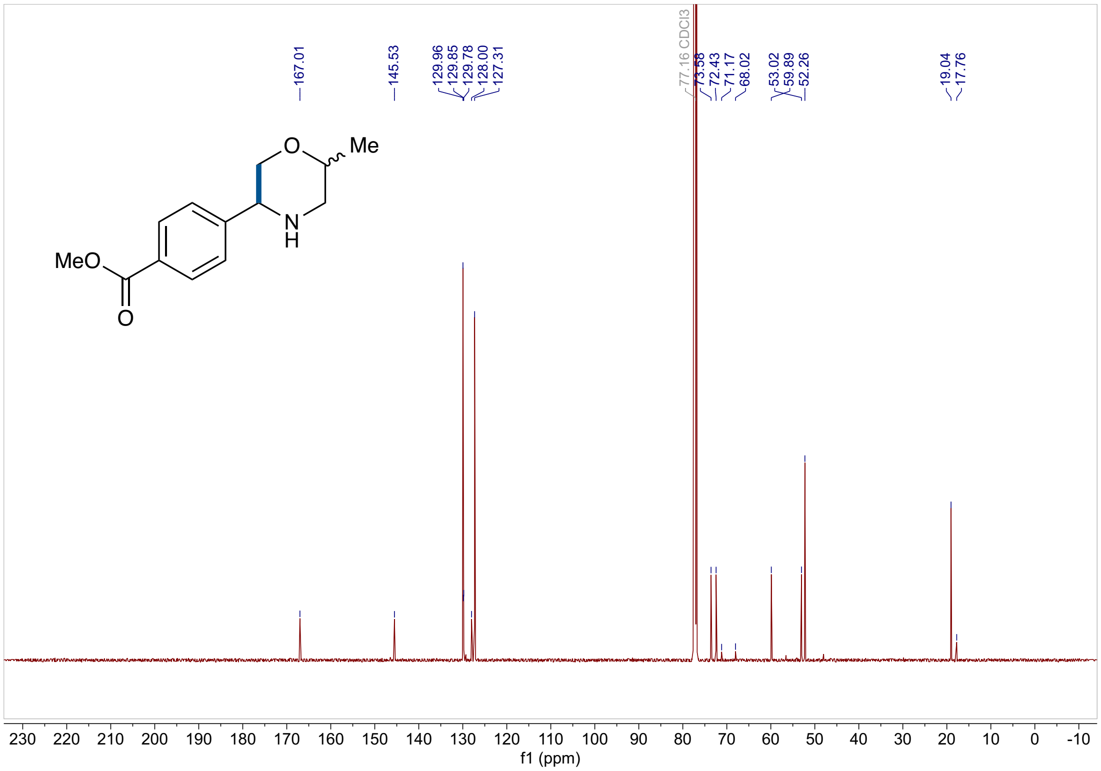

45 – ^{13}C NMR (126 MHz, CDCl$_3$)

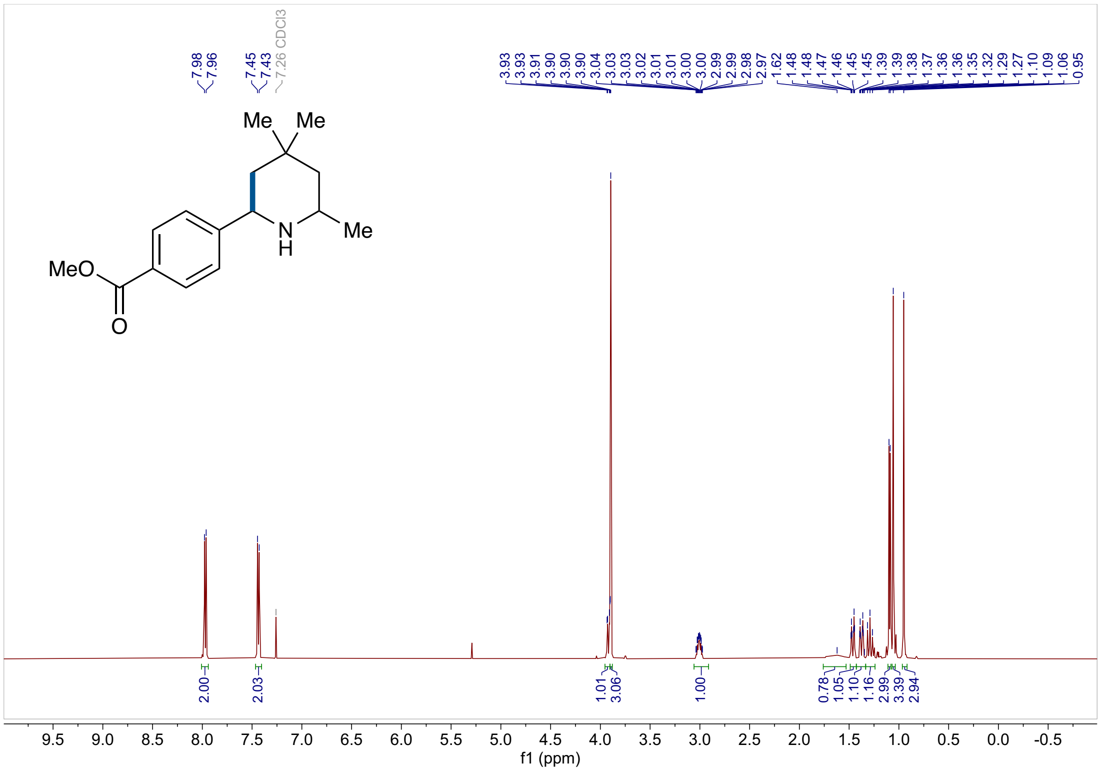

S-2 – ^{1}H NMR (500 MHz, $CDCl_3$)

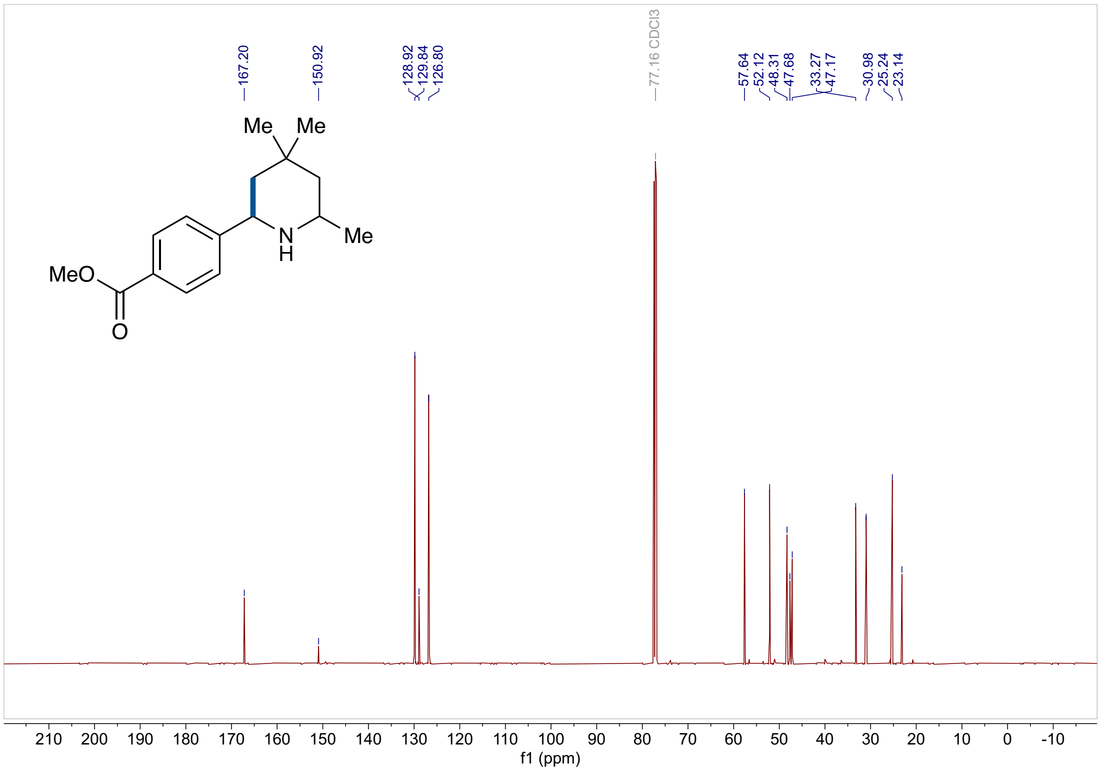

S-2 – ^{13}C NMR (126 MHz, $CDCl_3$)

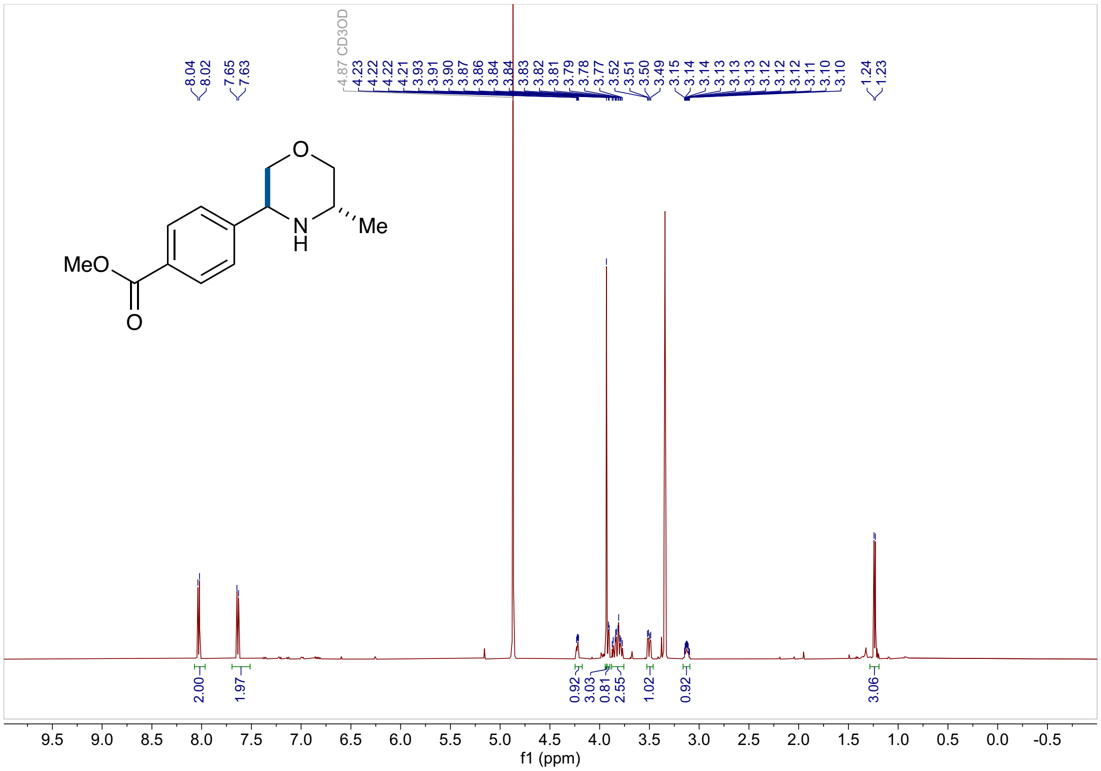

46 – ^{1}H NMR (500 MHz, MeOD)

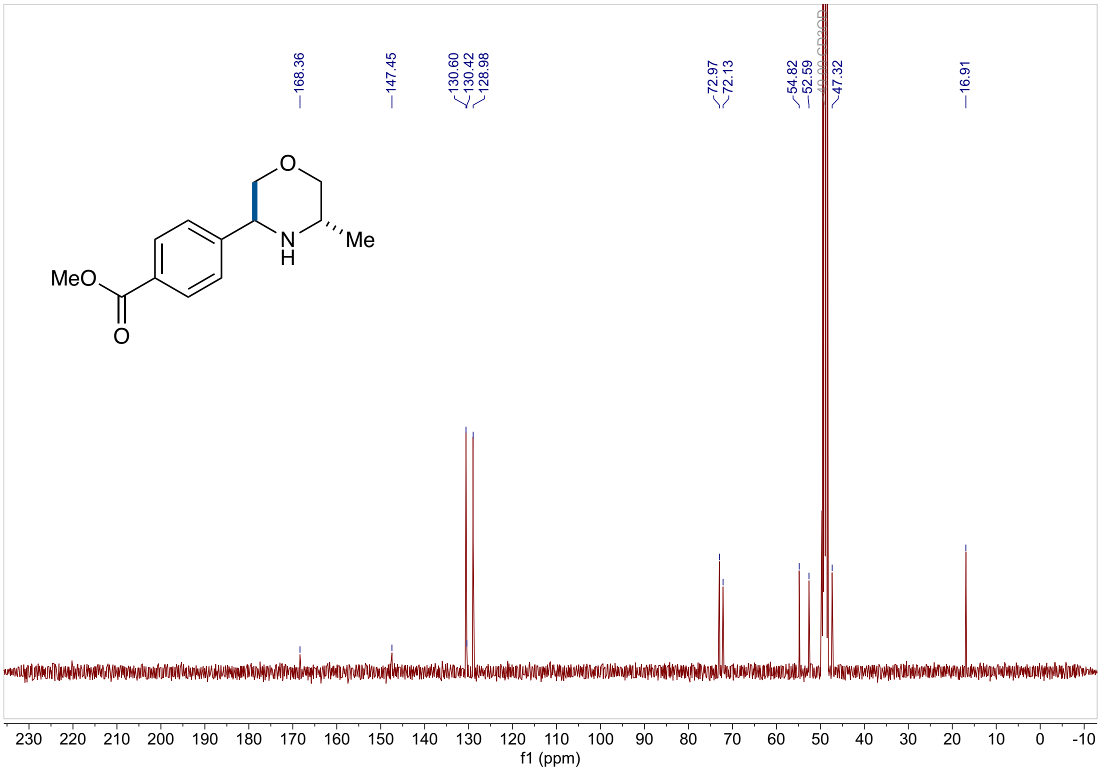

46 – ^{13}C NMR (101 MHz, MeOD)

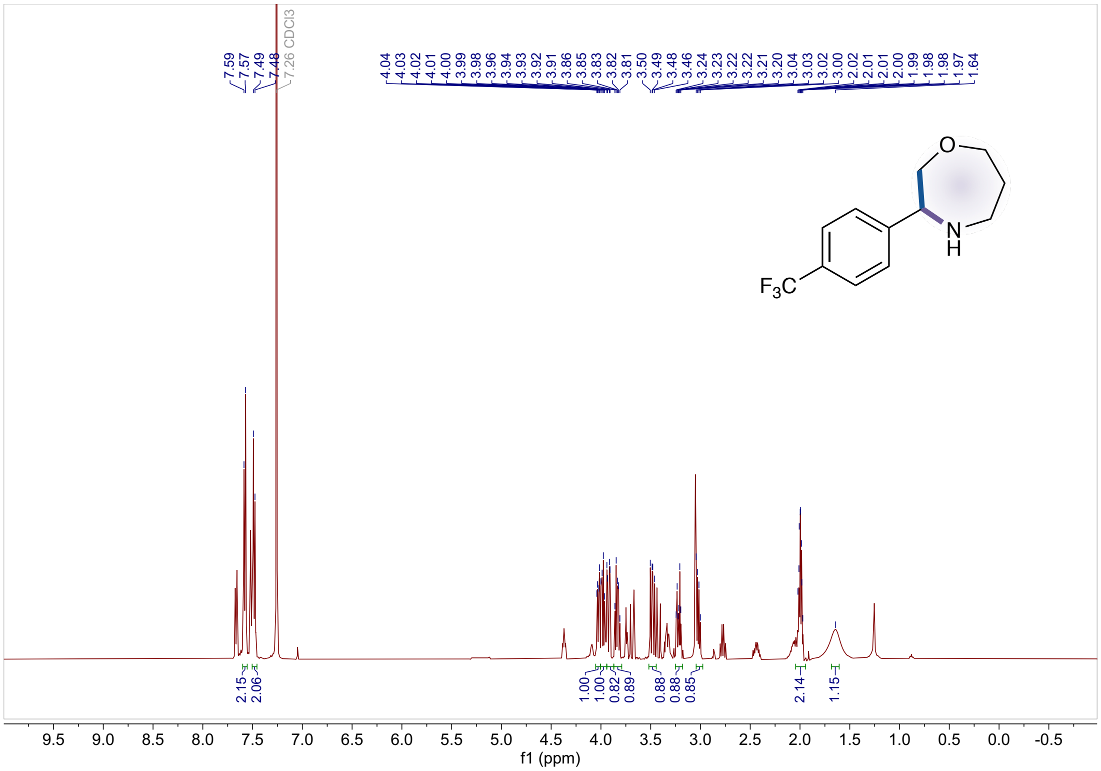

SI-sub – ^{1}H NMR (500 MHz, $CDCl_3$) [integration for major product – oxazepane]

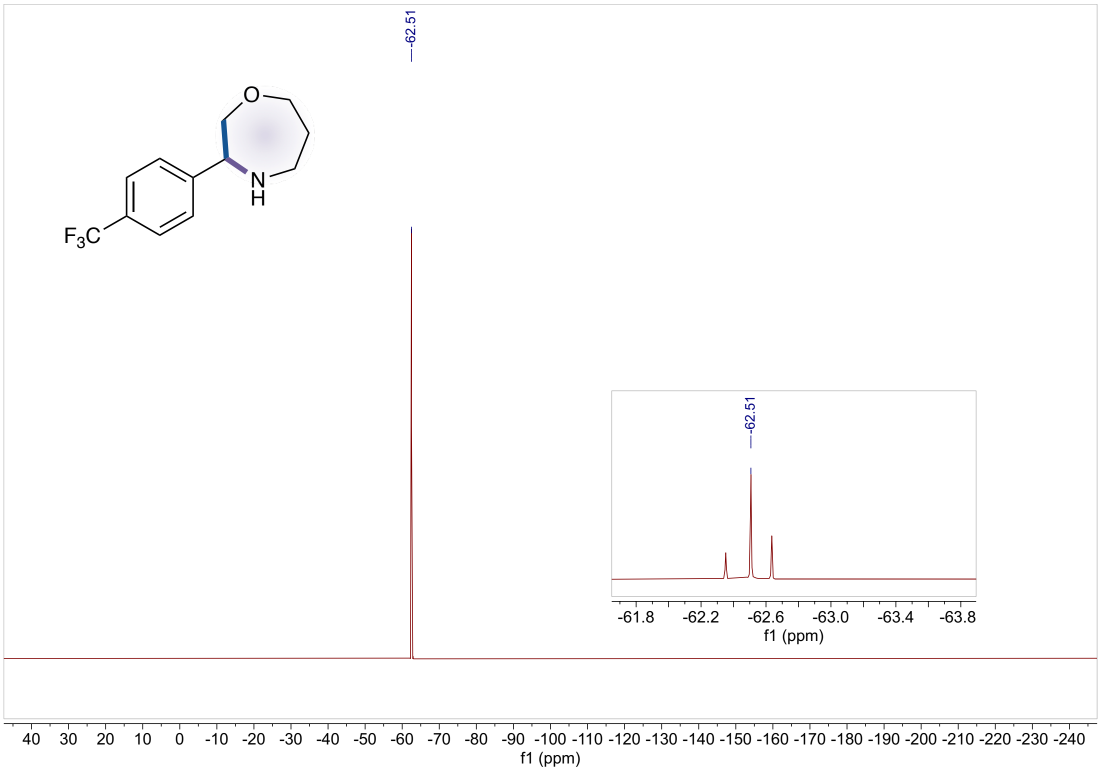

SI-sub – ^{19}F NMR (471 MHz, $CDCl_3$) [assignment for major product – oxazepane]

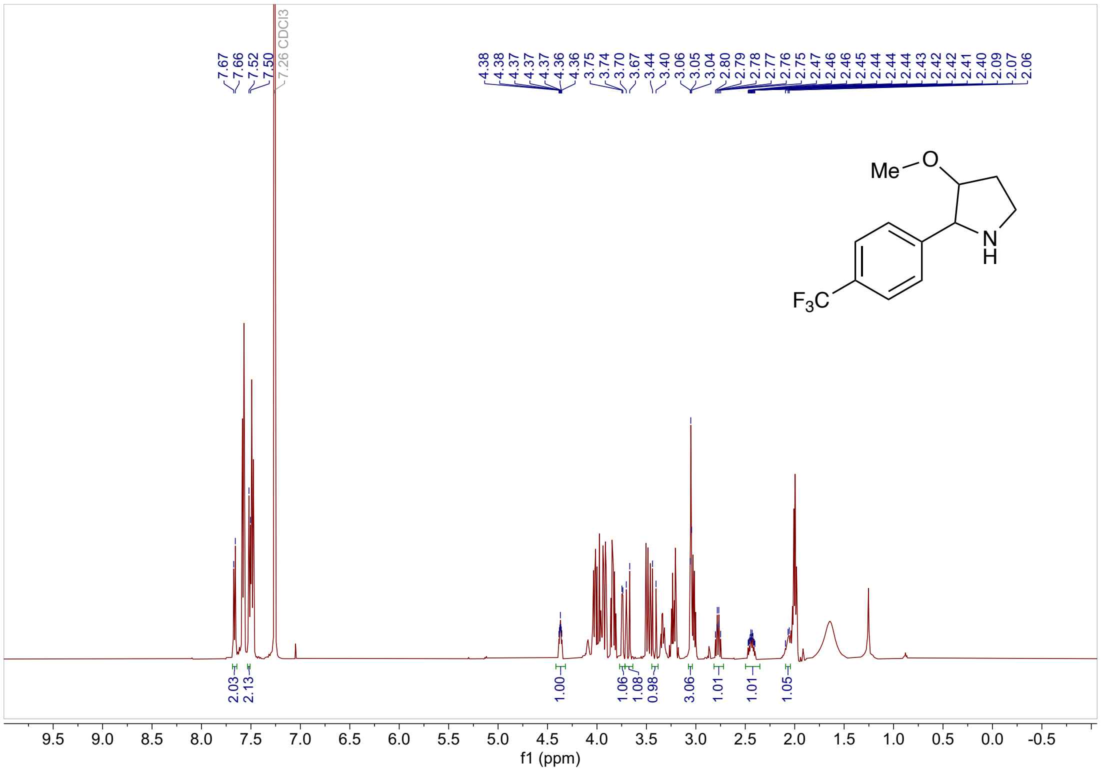

SI-sub – ^{1}H NMR (500 MHz, $CDCl_3$) [integration for minor product – pyrrolidine]

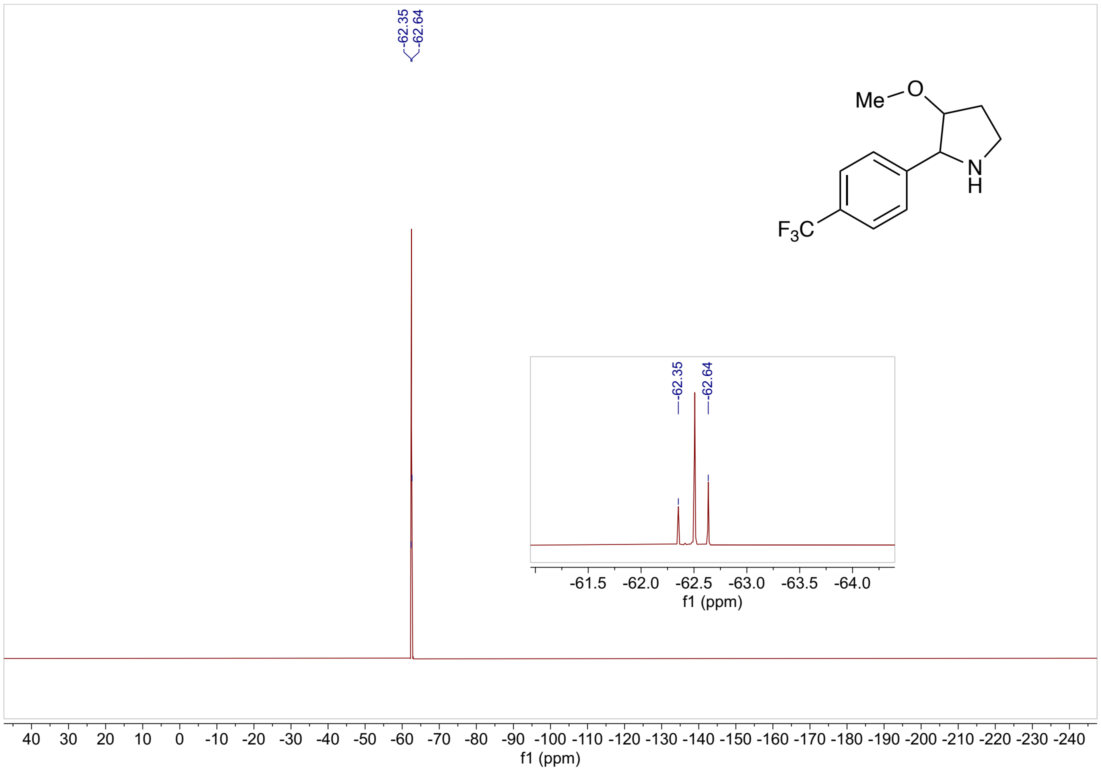

SI-sub – ^{19}F NMR (471 MHz, $CDCl_3$) [assignment for minor product – pyrrolidine]

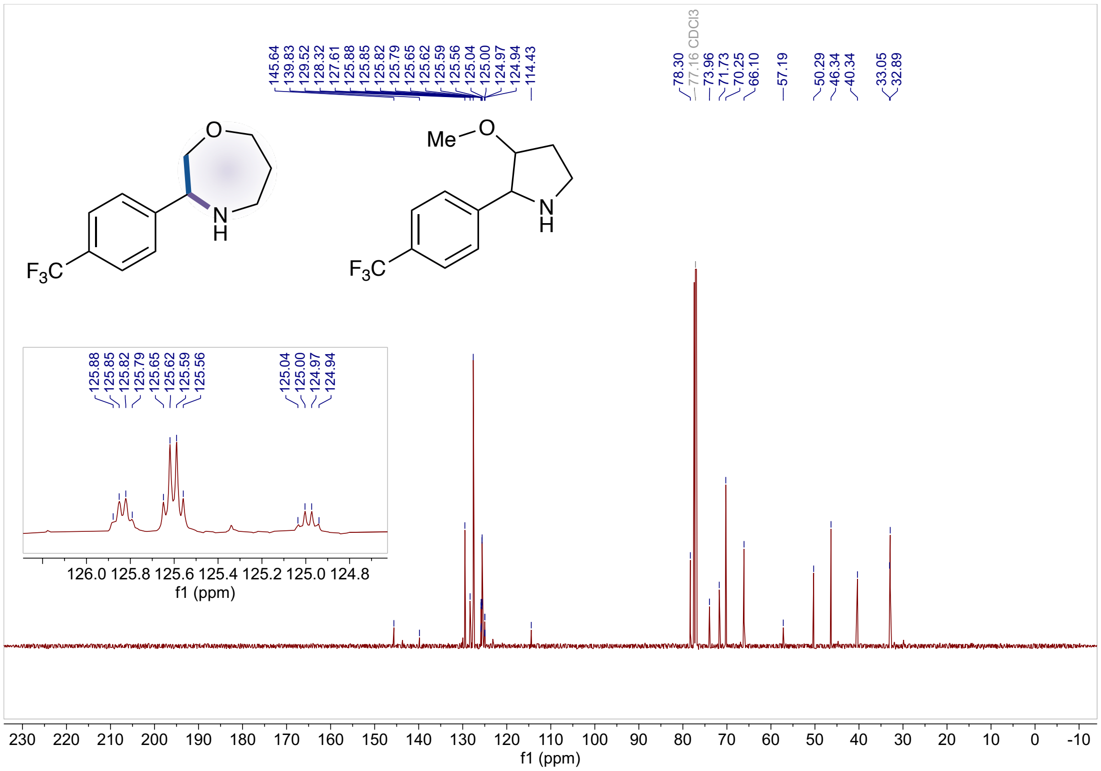

SI-sub – ^{13}C NMR (126 MHz, $CDCl_3$) [mixture of major and minor regioisomers]

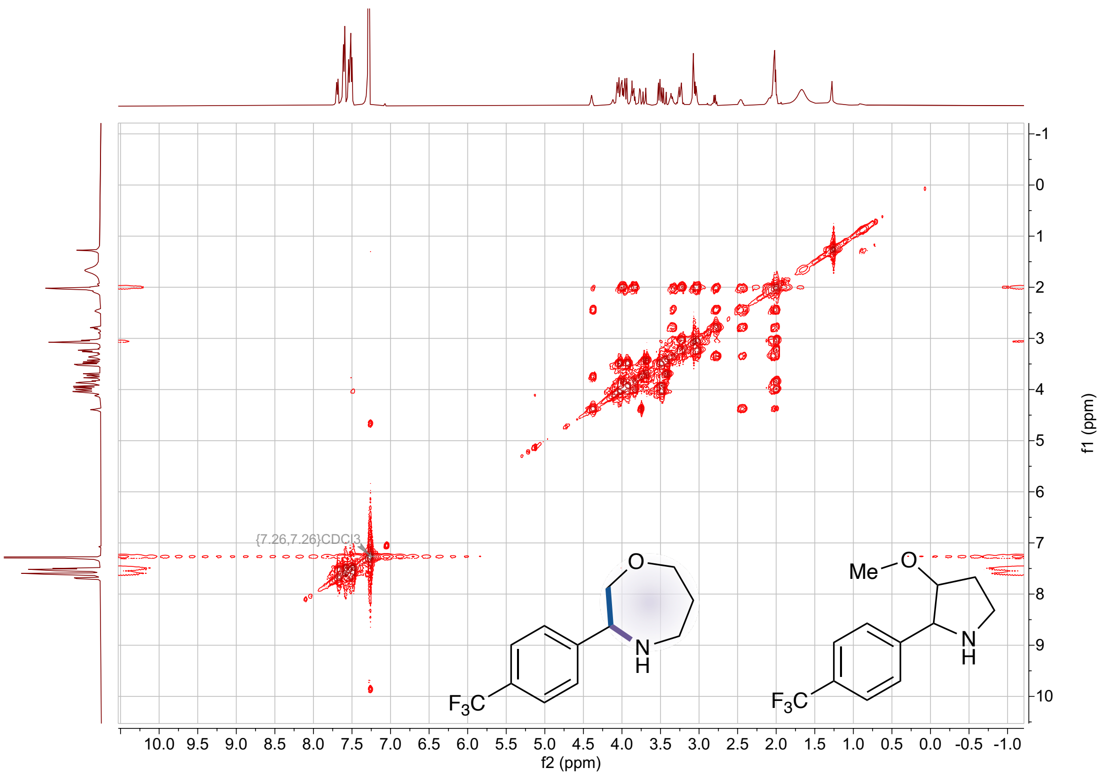

SI-sub – COSY

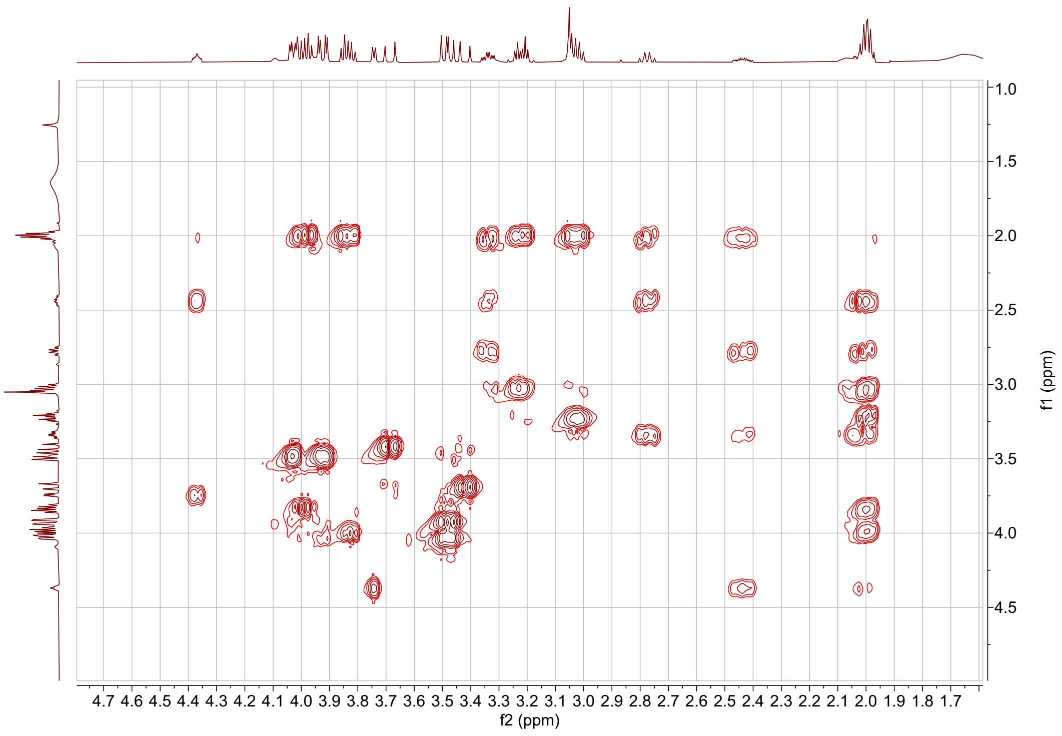

SI-sub – COSY (zoomed in from 1.0 to 5.0 ppm)
